# Enantioselective one-carbon expansion of aromatic rings by simultaneous formation and chromoselective irradiation of a transient coloured enolate†

**DOI:** 10.1039/d1sc06684f

**Published:** 2022-01-25

**Authors:** Rakesh K. Saunthwal, James Mortimer, Andrew J. Orr-Ewing, Jonathan Clayden

**Affiliations:** School of Chemistry, University of Bristol Cantock's Close Bristol BS8 1TS UK j.clayden@bristol.ac.uk

## Abstract

Enantioenriched seven-membered carbocycles are motifs in many molecules of structural and biological interest. We report a simple, practical, transition metal-free and mechanistically unusual method for the enantioselective synthesis of substituted cycloheptatrienes. By forming a coloured enolate with an appropriate absorption band and selectively irradiating *in situ*, we to initiate a tandem, asymmetric anionic and photochemical ring expansion of readily accessible *N*-benzylbenzamides. The cascade of reactions leading to the products entails enantioselective benzylic deprotonation with a chiral lithium amide, dearomatizing cyclization of the resulting configurationally defined organolithium to give an extended amide enolate, and photochemically induced formal [1,7]-sigmatropic rearrangement and 6π-electrocyclic ring-opening – the latter all evidently being stereospecific – to deliver enantioenriched cycloheptatrienes with embedded benzylic stereocentres.

## Abstract 2

Irradiation of a mixture of aromatic amide and chiral base leads to a tandem reaction sequence in which dearomatization forms a chromophore capable of photochemical rearrangement leading to overall asymmetric expansion of the aromatic ring.

## Graphical abstract

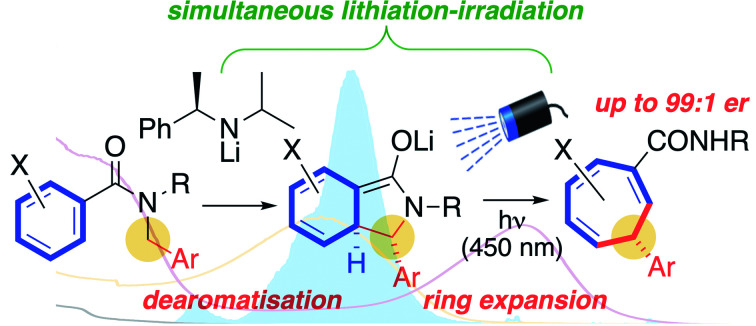

## Introduction

**Scheme 1:**
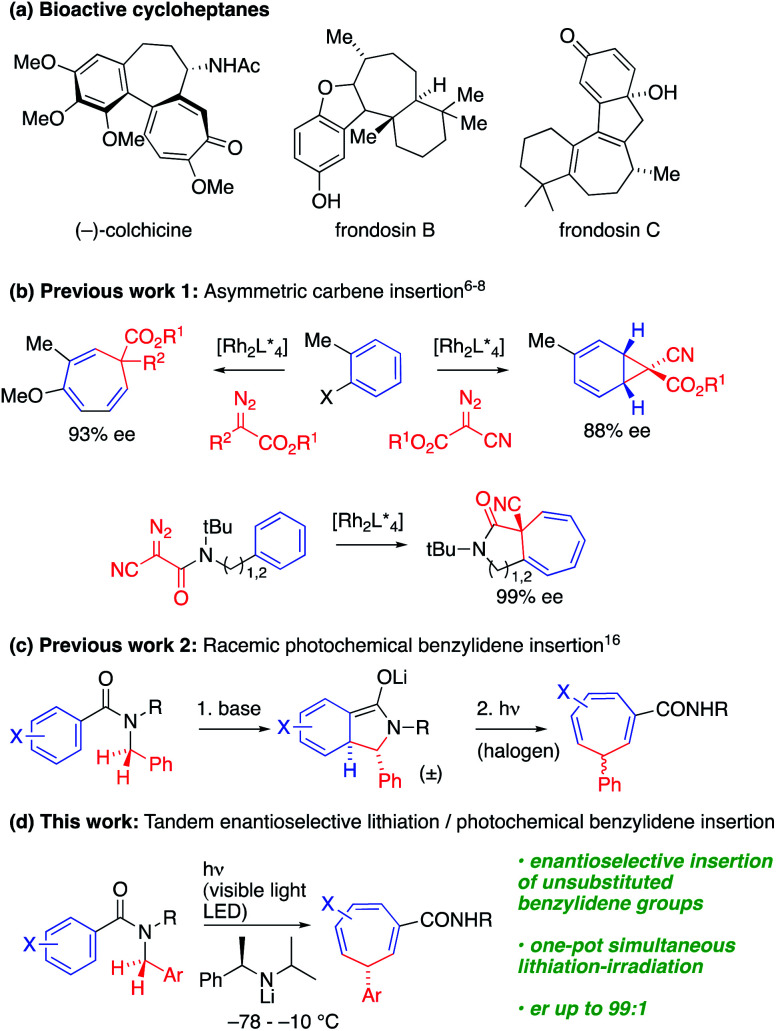
Cycloheptatrienes and synthetic approaches.

Saturated and partially saturated seven-membered carbocyclic derivatives are widely prevalent in bioactive natural products and pharmaceutical molecules.^1^ For example, (−)-colchicine, frondosin B and frondosin C show anti-inflammatory activities (Scheme 1a).^2^ There are many approaches to the synthesis of these compounds, but connective, enantioselective methods for preparing the parent unsaturated members of the class, cycloheptatrienes,^3^ with a single stereogenic sp^3^-hybridised carbon atom, are limited.

An appealingly simple approach to these compounds is the one-carbon ring expansion of aromatic systems. Early photochemical and thermal instances of these reactions^4,5^ had limited synthetic utility and poor selectivity due to rapid isomerization of the cycloheptatriene. However, recently, Beeler^6^ and Darses^7^ have reported enantioselective Rh-catalysed carbene insertions, allowing the synthesis of enantioenriched cycloheptatrienes (Scheme 1b). In related work, Johnson described a Rh-catalyzed asymmetric dearomative arene cyclopropanation using α-cyanodiazoacetates as carbene precursors,^8^ but enantioselective insertion reactions have so far been limited to fully substituted C atoms.^6^ Synthetic approaches to less substituted targets must negotiate the challenges presented by reversible hydride shifts or racemization by deprotonation.

The synthesis of a complex molecular framework in a single step from simple precursors is a characteristic feature of photochemical reactions.^9,10^ Photochemical reactions have enabled numerous approaches to the enantioselective synthesis of bioactive and other useful compounds,^11^ but control of enantioselectivity in the excited state continues to pose a challenge.^12^ Recent progress in asymmetric photochemical reactions has mainly focused on [2 + 2] cycloadditions and [1,3]-migration reactions;^13^ much rarer are enantioselective [1,7]-sigmatropic rearrangements.^14^

Dearomatizing reactions are an important way of introducing complexity into cyclic systems,^15^ and an alternative approach to the construction of cycloheptatrienes entails photochemical ring expansion of lithiated *N*-benzylbenzamides.^16^ We previously noted (Scheme 1c) that dearomatized enolates undergo photochemical rearrangement to cycloheptatrienes on irradiation with a 500 W halogen lamp, but enantioselective variants of this reaction were limited to stereospecific transformations of chiral precursors. We now show that the whole cascade of reactions from the simple achiral benzamide precursor to the chiral cycloheptatriene may be rendered enantioselective by using a chiral lithium amide base to perform an asymmetric deprotonation while simultaneously irradiating, with a visible light 40 W LED, the mixture of anions that is formed by rearrangement. Choice of wavelength induces chromoselective isomerization of only one of the three conjugated anions in the mixture. The operationally simple cascade reaction that results achieves a remarkable chemical transformation: the conversion of a benzylic amide into a benzylidene group that is enantioselectivity inserted into the σ-bond framework of the aromatic ring.

The cascade starts with the enantioselective deprotonation and dearomatizing cyclization of an *N*-benzylbenzamide,^17^ a reaction that provided a key partially saturated isoindolinone intermediate for the synthesis of the (−)-isodomoic acids and other kainoids.^17*a*–*d*^ We proposed that photochemical rearrangement of enolate anions resulting from widening this cyclisation to other substrates could provide an enantioselective version of Scheme 1c, and consequently a general enantioselective route to the less substituted cycloheptatrienes that are unavailable by other methods.

**Scheme 2:**
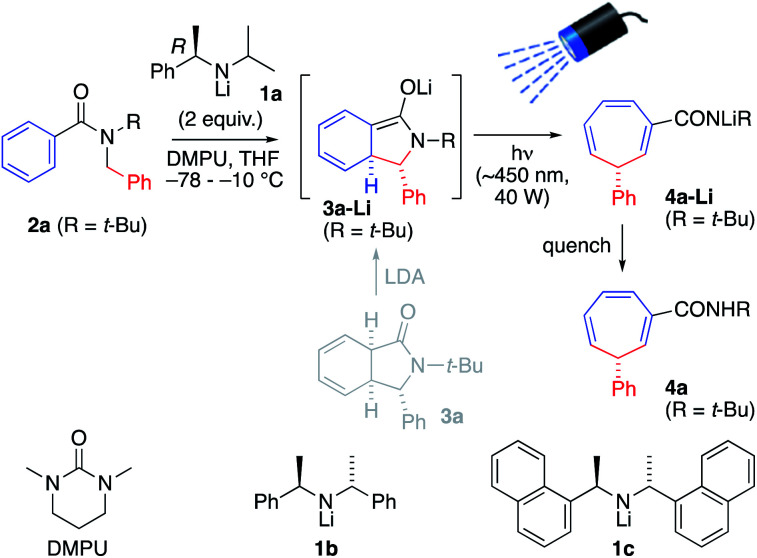
Simultaneous lithiation/irradiation.

To explore this possibility, *N-t*-butyl benzamide 2a was treated with 2.0 equiv. of the chiral lithium amide base (*R*)-1a and DMPU (6 equiv.) at −78 °C, warming to −10 °C for 4 h to induce dearomatizing cyclisation to 3-Li, completion of which was confirmed by TLC (Scheme 2 and Table 1, entry 1). Then, the mixture was irradiated from above for 3 h at −10 °C with a visible light LED (*λ*_max_ = ∼450 nm, 40 W ‘Kessil Blue’) to induce ring-expanding rearrangement.^18^ Under these conditions, the product cycloheptatriene 4a was obtained in 60% yield with 93 : 7 er, confirming that the ring expansion from 2a to 4a can proceed with stereoselectivity. However, there was significant decomposition during the reaction, presumably due to the instability of the dearomatized intermediate 3a-Li, so we decided to attempt simultaneous lithiation and irradiation, to allow the cyclized enolate anion 3a-Li to rearrange as soon as it formed (entry 2). This protocol gave a significantly higher yield (though slightly lower er), suggesting that of the components of the reaction mixture, only the enolate has a productive electronic transition in the requisite visible part of the spectrum.

**Table 1:** Development of the simultaneous lithiation/irradiation protocol^a^

Entry	R=	Base	DMPU equiv.	Time (h)	Yield 4^b^ (%)	er^c^
1^d^	*t*-Bu	1a	6	4 + 3	60	93 : 7
2	*t*-Bu	1a	6	5	80	86 : 14
3	*t*-Bu	1a	3	5	71	96 : 4
4	*t*-Bu	1a	1	4	60	97 : 3
5	*t*-Bu	1a	—	8	40	>99 : 1
6	*t*-Bu	1b	3	5	68	74 : 26
7	*t*-Bu	1c	3	5	65	53 : 47
8	i-Pr	1a	3	5	72	42 : 58
9	Me	1a	3	4	—e	—

a Reactions performed using 0.37 mmol of 2a in 0.1 M THF.

b Isolated yield.

c Determined by HPLC analysis of chiral stationary phase.

d Stepwise: dearomatizing cyclization at −78 °C to −10 °C for 4 h followed by 3 h irradiation at −10 °C.

e Starting material decomposed.

**Fig. 1:**
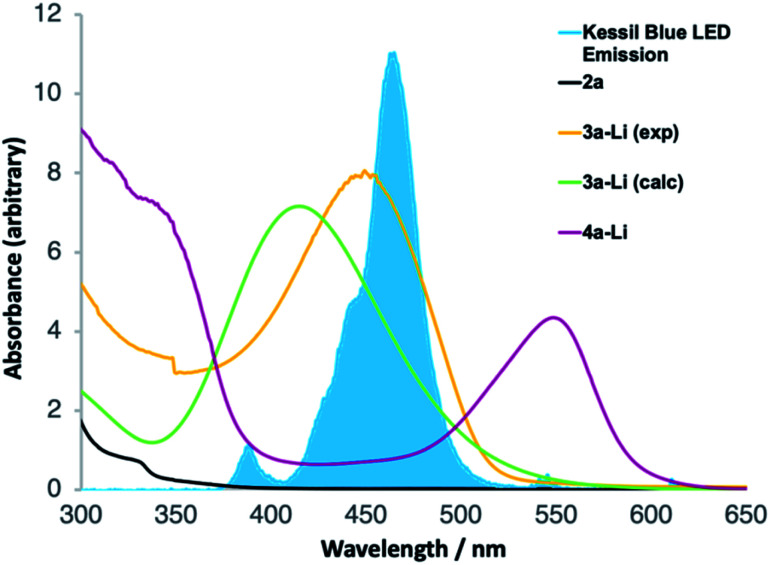
UV-Vis spectroscopy of reaction intermediates.

UV-Vis spectroscopy (Fig. 1) confirmed that this is indeed the case. Spectra were acquired, in the reaction solvent THF, of 2a, 3a-Li (by deprotonating an authentic sample of 3 with *n*-BuLi) and 4a-Li (by deprotonating the product 4a with *n*-BuLi). Fig. 1 superimposes these spectra upon the emission spectrum acquired from the visible light LED used for the reaction, and of these components of the reaction mixture (see further discussion below) only 3-Li has an absorption band in the range of the emission of the LED. Time-dependent DFT calculation of the UV-Vis spectrum of 3a-Li (Fig. 1, green line; for details see ESI†) provided further support for the proposal that the reaction proceeds because 3a-Li absorbs strongly in the region of emission of the blue LED. This versatile technique of simultaneous irradiation and deprotonation thus allows the *in situ* generation of a photoactive species that may be chromoselectively^19^ rearranged in the presence of other chromophores.

Further optimization of the reaction was carried out. In accordance with previous observations that DMPU increases both nucleophilicity and reaction rates of organolithiums,^20^ reducing the amount of DMPU raised the er but at the cost of yield (Table 1, entries 3–5). More hindered bases 1b and 1c gave poor er (entries 6 and 7), and it is worth noting that 1a offers greater practicality than 1b or 1c due to its higher polarity and ease of separation from the products. Reduction of the size of the *t*-butyl substituent to i-Pr (entry 8) gave poorer er, even when paired with a more hindered base; an *N*-methyl benzamide was unstable towards strong base (entry 9). Adding LiCl did not improve yield or selectivity.

**Scheme 3:**
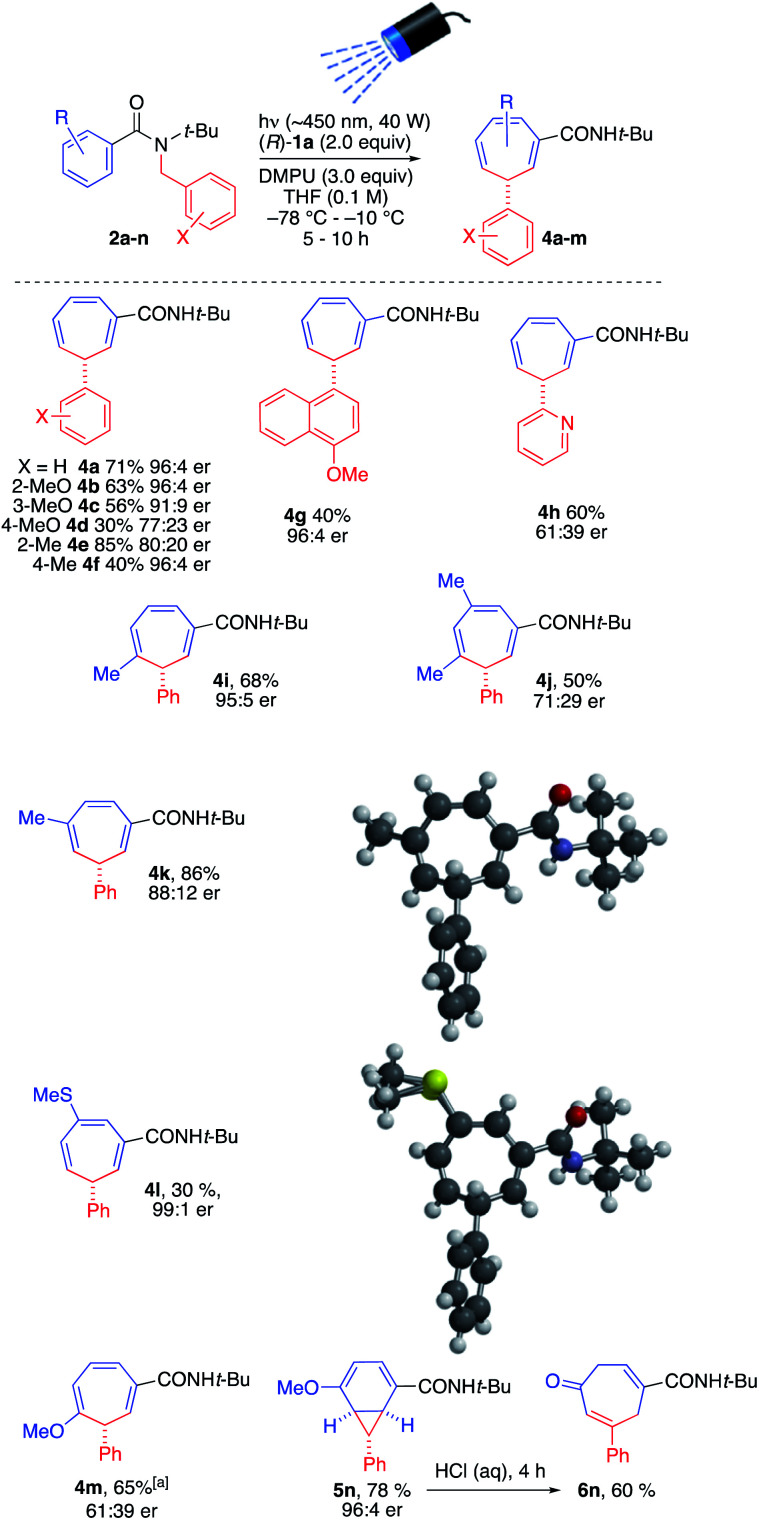
Scope of enantioselective photochemical ring expansion reaction. ^*a*^Carried out in two steps.

The conditions of entry 3 were selected as the optimal pairing of high enantioselectivity with good yield, and using this protocol we set out to investigate the scope of the reaction (Scheme 3), bearing in mind that asymmetric deprotonation of benzamides using chiral lithium amides had been demonstrated only for simple unsubstituted substrates.^17*a,c*^

We found that in general the tandem lithiation-ring expansion tolerated a range of substituents with differing steric and electronic properties in both the benzamide ring and *N*-benzyl group, in addition to delicate functional groups. In general, enantioselectivity was substantially compromised by substitution on the inserting benzyl group, with methoxy (4b–4d) and methyl (4e, 4f) substituents rearranging in moderate to good yield. The more hindered 4-methylnaphthamide substituent of 4g likewise inserted enantioselectively, if in lower yield. Even a 3-pyridyl substituent underwent the reaction, though the stabilized nature of its anion led to poor er in product 4h.^21^

Substitution on the expanding ring was tolerated to varying degrees, with er being high in many cases but dropping with some more electron-rich rings. 3-, 4- and 3,5-(Di)methylsubstituted rings expanded to methylcycloheptatrienes 4i, 4k and 4j in moderate to good yield and varying er. 3-Methylthio-substituted 2l gave 4l in excellent ee but poorer yield, while its 3-methoxy analogue 2m reacted with differing regiochemistry (presumably driven by inductive activation of the 2-position) to give 4m. The decreased reactivity of this electron-rich substrate lowered the er of the product and meant that this reaction was optimally carried out in two stages – allowing slow dearomatisation to reach completion before irradiating the enolate. With a 4-methoxy substituent, the isomeric norcaradiene 5n (in which conjugation between the methoxy group and the amide substituent is maintained) was obtained rather than a cycloheptatriene. Hydrolysis of the enol ether promoted electrocyclic ring-opening to cycloheptadienone 6n.

The absolute configuration of the products was established by X-ray crystallography of 4k and supported by X-ray crystallography of 4l.^22^ Circular dichroism spectra were obtained for 4a, 4c, 4d, 4k, 4l, and 4m and in every case a similar pattern was obtained (see ESI†): a significant positive band at ∼220 nm, a (usually) weaker negative band at ∼260 nm, and a weak or very weak positive band at ∼320 nm. We therefore assume all products represented in Scheme 3 are homochiral with one another.

**Scheme 4:**
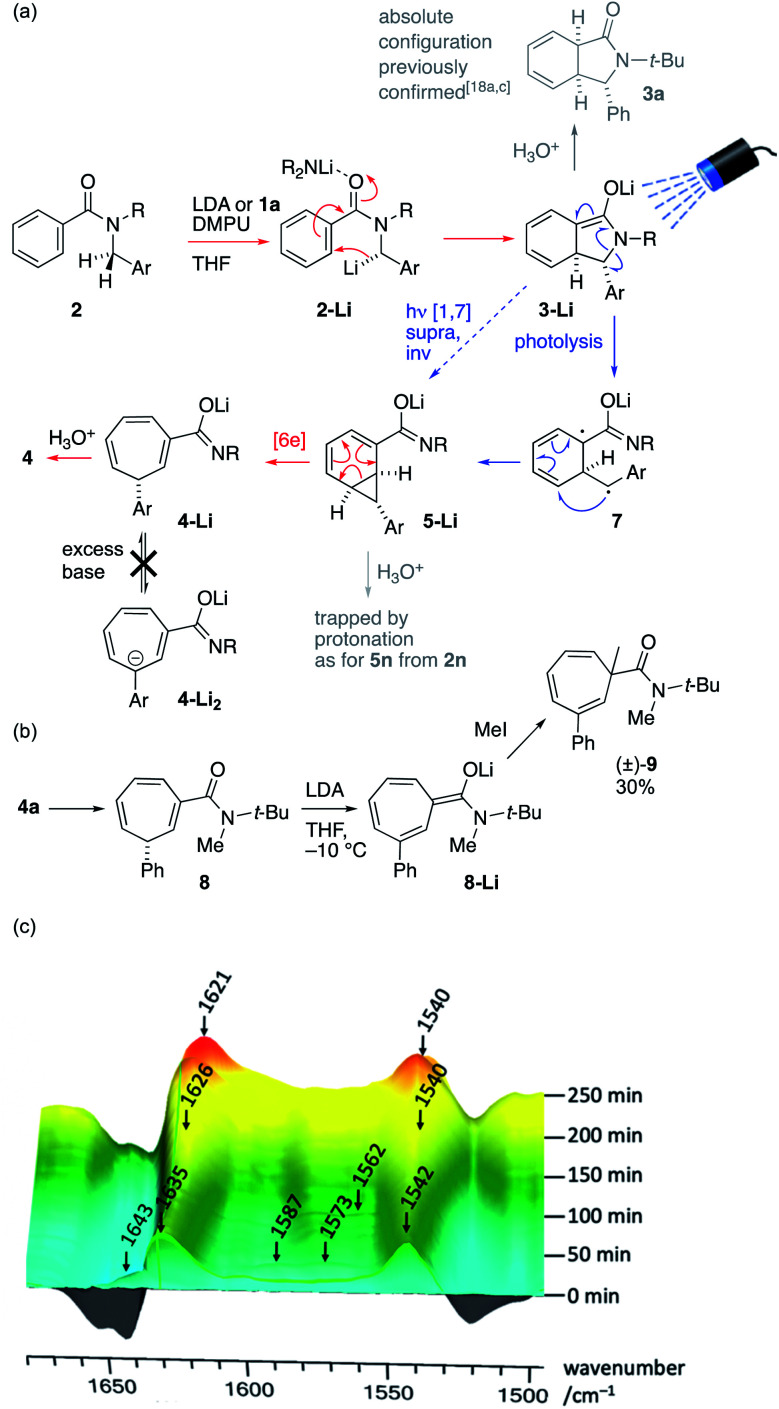
(a) Proposed mechanism; (b) exploration of ring deprotonation and (c) evolution of the reaction mixture studied by *in situ* infra-red spectroscopy (ReactIR).

The operational simplicity of the reaction belies its mechanistic complexity. Our hypothesis with regard to the sequence of events that lead to the ring-expanded products 4 is illustrated in Scheme 4a, in which thermal steps are indicated by red arrows and photochemical steps by blue arrows. Amide 2 is enantioselectively deprotonated by (*R*)-1a to yield benzylic organolithium 2-Li which undergoes precedented asymmetric dearomatizing cyclisation to the bicyclic extended enolate 3-Li, placing the Ar substituent on the *exo* face of the new bicyclic system. The relative and absolute configuration of 3-Li has previously been established unequivocally^17*a*,*c*^ and the possibility of the reaction being electrocyclic in nature have been explored experimentally^17*e*^ and computationally.^17*f*^ As shown in Fig. 1, extended enolate chromophore 3a-Li absorbs visible light, promoting rearrangement to the norcaradiene 5-Li either by a concerted photochemical [1,7]-rearrangement or by photolysis to a diradical 7a and recombination. The intermediacy of norcaradiene 5 is supported by the rearrangement of 2n, which initially yields isolable 5n on protonation. The electrocyclic ring-opening of 5-Li gives, after protonation, the product cycloheptatriene 4. Given the established enantioselectivity of benzylic deprotonation with (*R*)-1a,^18a,c^ the absolute configuration of the products is consistent with overall formal inversion of configuration at the benzyl group either as a result of a photochemically allowed suprafacial invertive (but apparently unprecedented) [1,7] migration of C, or with the transient formation of a new stereogenic centre in 3-Li, which, although lost in the final product, ensures stereoselective re-formation of the benzylic centre in the favoured, *exo*, diastereoisomer of the norcaradiene 5-Li (a stereochemical strategy reminiscent of the ‘self-regeneration of stereocentres’ concept^23^).

We ascribe the surprising stability of the product towards racemization by a deprotonation/reprotonation pathway to two features: firstly, the fact that deprotonation at the stereogenic centre is disfavoured by the antiaromaticity of the resulting anion, and secondly the anionic secondary amide substituent. The critical ‘protecting’ role of this anionic amide was demonstrated by *N*-methylation of 4a to give 8: treatment of this neutral tertiary amide with LDA gave an extended enolate 8-Li that was readily α-methylated to yield racemic 9 (Scheme 4b).

Evidence for the intermediacy of the species shown in Scheme 4a was initially gathered by quenching the reaction of 2a under the standard conditions after only 2 h at −10 °C (see ESI†). The crude ^1^H NMR spectrum of the mixture obtained exhibited signals characteristic of all of 2a, 3a, 5a and 4a in a ratio of 0.42 : 0.11 : 0.84 : 1.0. Similar experiments repeated after periods from 0.5–3 h showed complex behaviour in which a small amount of 4a is initially formed rapidly, with intermediates 3a and 5a then accumulating in varying proportions, before declining with time. This behaviour is consistent with a rapid photochemical step that becomes retarded by the formation of a turbid or coloured solution as the reaction progresses, and by the possible involvement of the conjugate acid of 1a generated by deprotonation.

*In situ* infra-red spectroscopy (React-IR, Scheme 4c, baseline-corrected for THF + DMPU) of the reaction of 2a with LDA at −10 °C revealed a similar story, in which the carbonyl signal of starting material 2a (1643 cm^−1^, partly obscured by peaks resulting from DMPU) is rapidly replaced by signals at 1635 and 1542 characteristic of a lithio derivative (hypothesized to be 2a-Li) that is likewise formed by lithiation without irradiation (see ESI†). During the following 4 hours, peaks corresponding to 3a-Li (1587 and 1573) and 5a-Li (1562 cm^−1^) grew and then declined, while peaks corresponding to the product 4a-Li (1626 and 1540 cm^−1^) eventually became dominant, before finally forming 4a (1621 and 1540 cm^−1^) on quench.

**Scheme 5:**
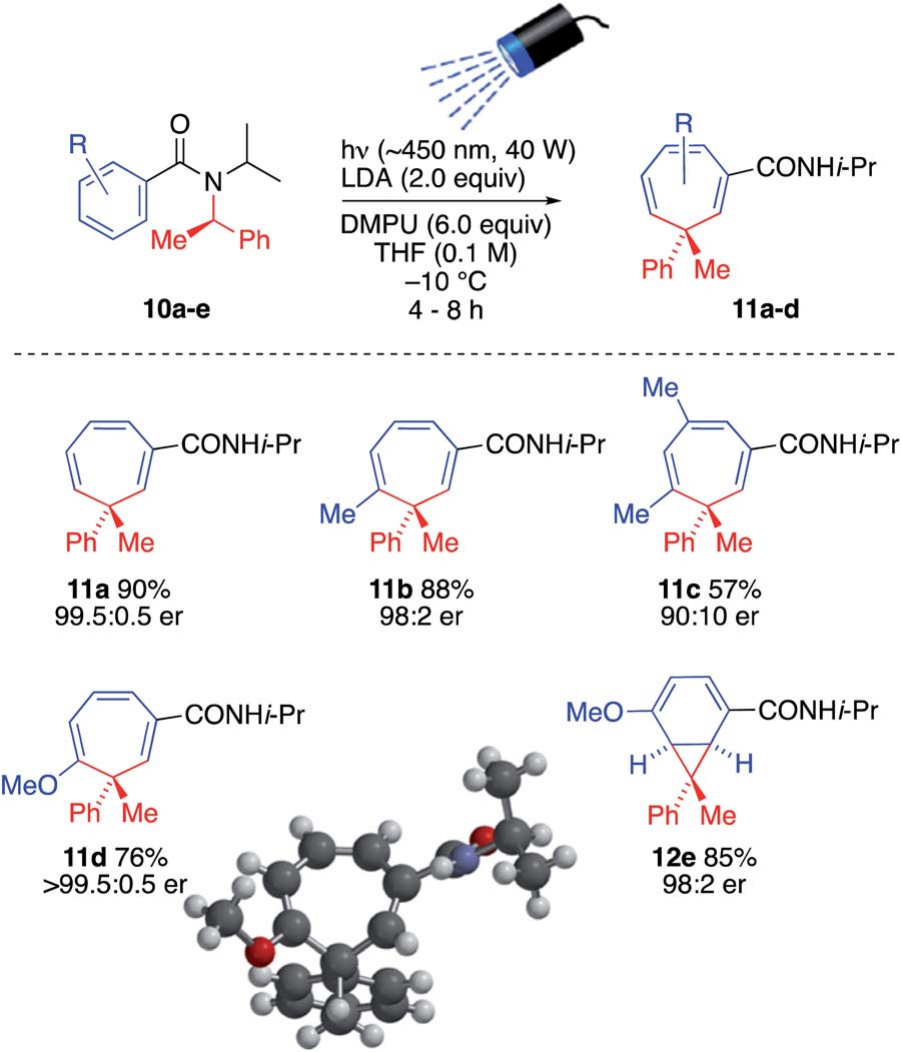
Simultaneous lithiation–irradiation for stereospecific insertion of a quaternary centre into a benzamide ring.

The ability to generate a thermally reactive but photochemically inert organolithium and induce rearrangements of its thermally stable but photochemically reactive reaction products by simultaneous lithiation and chromoselective irradiation is intriguing and valuable strategy that we found could be applied to other similar reactions. Thus, treatment of enantiopure precursors 10a–10e with LDA while irradiating with visible light (*λ*_max_ = ∼450 nm, 40 W Kessil blue LED) generated almost enantiopure 3-methyl-3-phenylcycloheptatriene derivatives 11a–11d and norcaradiene 12 with high levels of stereospecificity, presumably by retentive lithiation and overall retentive rearrangement by way of a configurationally stable organolithium (Scheme 5).^16^ The absolute configuration of 11d was established by X-ray crystallography and CD spectra of 11a, 11c and 11d were superimposable (see SI). Consistent with formation of 5l from 4-methoxybenzamide 2l, 4-methoxybenzamide 10e gave the norcoradiene isomer 12e.^21^

**Scheme 6:**
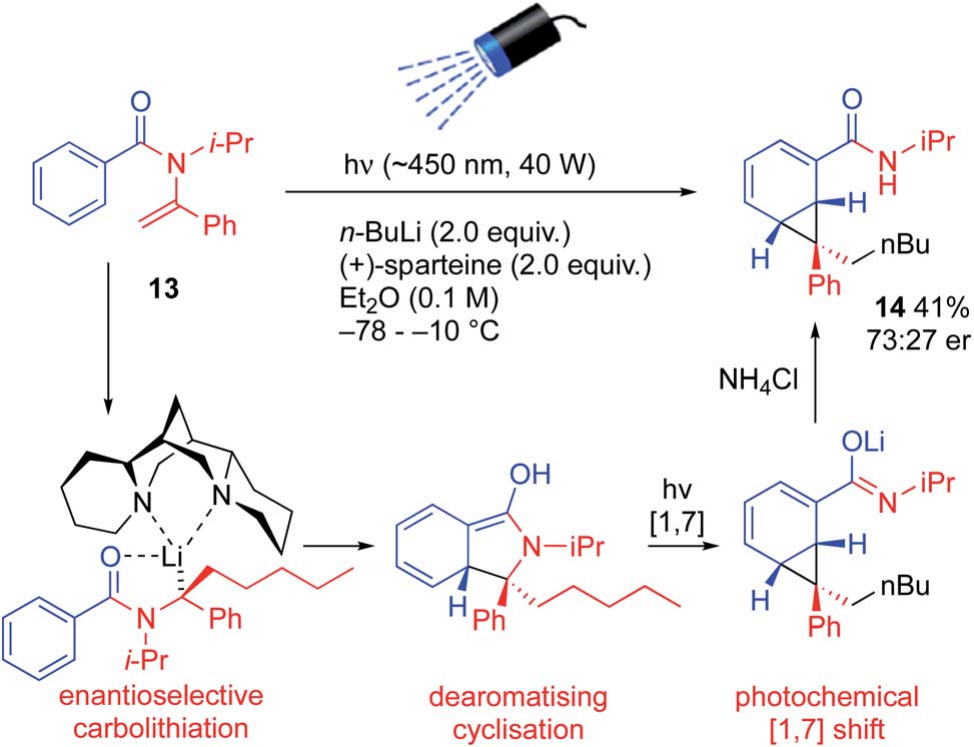
Asymmetric carbolithiation followed by photochemical rearrangement.

More substituted configurationally defined organolithiums may be generated by enantioselective carbolithiation of *N*-acylenamines.^24^ Treatment of the vinylic amide 13 with *n*-BuLi and (+)-sparteine in ether while irradiating the mixture initiated a remarkable cascade of reactions that led to the enantioenriched norcaradiene 14, albeit in moderate yield and er.^25^ Enantioselective carbolithiation of 13 (a reaction known for vinylic carbamates, thiocarbamates and ureas,^24^ but not previously reported for amides) initiated dearomatizing cyclisation and photochemical rearrangement of the resulting lithium enolate (Scheme 6).

## Conclusions

In summary, we show that *in situ* generation of an anionic chromophore by deprotonation or carbolithiation, while simultaneously irradiating with visible light from a 40 W LED, is a practical and effective way to carry out a photochemical transformation by activating, chromoselectively, an extended enolate intermediate. Specifically, we have for the first time been able to insert enantioselectively into the σ framework of a range of substituted benzene rings a series of α-unsubstituted benzylidene groups by a cascade of addition, rearrangement and ring-opening steps. The products are enantiomerically enriched cyloheptatrienes with the potential for use as versatile synthetic building blocks.

## Data availability

Experimental and spectroscopic data are provided in the ESI.†

## Author contributions

RKS and JM carried out the experimental and computational work. AJO-E provided advice on computation and spectroscopy. JC directed the project.

## Conflicts of interest

There are no conflicts of interest to declare.

## Supplementary Material

SC-013-D1SC06684F-s001

SC-013-D1SC06684F-s002
