# Supplementary material for: Enantioselective one-carbon expansion of aromatic rings by simultaneous formation and chromoselective irradiation of a transient coloured enolate
Source: Chem Sci. 2022 Jan 25;13(7):2079–85. doi: 10.1039/d1sc06684f (PMC8848985; doi:10.1039/d1sc06684f)
Supplement: SC-013-D1SC06684F-s001 [file SC-013-D1SC06684F-s001.pdf]

Electronic Supplementary Material (ESI) for Chemical Science.
This journal is © The Royal Society of Chemistry 2022

Enantioselective one-carbon ring expansion of aromatic rings by simultaneous formation and chromoselective irradiation of a transient coloured enolate

Rakesh K. Saunthwal, James Mortimer, Andrew J. Orr-Ewing and Jonathan Clayden*

Experimental

General Information	S2
Analytical Information	S2
UV-visible Spectroscopy Measurements	S3
Literature Known Starting Materials	S3
General Procedure 1: Synthesis of *rac*-*N*-(1-Phenylethyl)-1-methylethylamine	S3
General Procedure 2: Synthesis of *(R)*-*N*-(1-Phenylethyl)-1-methylethylamine	S4
General Procedure 3: Synthesis of *(S)*-*N*-(1-Phenylethyl)-1-methylethylamine	S4
General Procedure 4: Starting material synthesis	S5
General Procedure 5: General procedure for the synthesis of amides 4.	S5
General Procedure 6: Enantioselective Photochemical Ring Expansion	S5
General Procedure 7: *rac*-Photochemical Ring Expansion	S6
General Procedure 8: Stereospecific Photochemical Ring Expansion	S7
General Procedure 9: *rac*-Stereospecific Photochemical Ring Expansion	S7
Analytical data for amines	S8
Analytical data for amides	S8-S17
X-ray crystallography Data	S17-S22
Analytical data for enantioselective photochemical ring expansion	S22-S36
Analytical data for product modifications	S36-S38
Analytical data for stereospecific photochemical ring expansion	S38-S41
Analytical data for carbolithiation and photochemical rearrangements	S42
Mechanistic Study	S43-S53
Experimental setup for ReactIR	S43
React-IR study	S43-S46
NMR Study	S47-S49
VCD spectra	S50-S53
Time-Dependent DFT Calculation	S54-S56
Copies of 1H, 13C and HPLC spectra	S57-S184

1 Experimental

1.1 General Information: Reactions requiring anhydrous conditions (where specified) were executed under dry nitrogen or argon atmospheres in glassware that was dried using either a combination of vacuum and heat-gun, oven, or flame drying. Reaction mixtures were stirred magnetically. Air- and moisture-sensitive liquids and solutions were transferred via syringe into the reaction vessels through rubber septa. All reagents were purchased (unless specified) at highest commercial quality and used as received. Non-anhydrous solvents were purchased (unless specified) at the highest commercial quality and used as received. Anhydrous CH_2Cl_2 and THF were obtained from the University of Bristol's dry solvent system and were purified by filtration over a column of activated alumina. All temperatures described below −10 °C were achieved using a Julabo cryostat.

1.2 Analytical Information:

Rf: TLC was performed on aluminium-backed silica plates (0.2 mm, 60 F254) which were developed using standard visualising agents: UV fluorescence (254 & 366 nm), phosphomolybdic acid / Δ, vanillin / Δ, potassium permanganate / Δ and Seebach / Δ. Chromatography: Flash chromatography was performed on an automated Biotage Isolera TM Spectra Four using gradient elution on pre-packed silica gel Biotage® Sfar Silica D Duo columns.

MP: Melting points were measured on a Kofler hotstage melting point apparatus and are uncorrected.

IR: IR spectra were recorded on neat compounds using a Perkin Elmer (Spectrum One) FT-IR spectrometer (ATR sampling accessory). Only strong and selected absorbance's (ν_{max} expressed in cm^{-1}) are reported.

^{1}H NMR: Spectra were recorded on Jeol ECS (400 MHz) or Bruker NMR (400 MHz or 500 MHz) instruments. Chemical shifts (δ H) are quoted in parts per million (ppm) was used. Spin-spin coupling constants (J) are reported in Hertz (Hz). 2D NMR experiments NOSY, HSQC and HMBC where necessary.

13C NMR: Spectra were recorded on Jeol ECS (101 MHz) or Bruker NMR (101 MHz or 125 MHz) instruments. Chemical shifts (δ C) are quoted in parts per million (ppm) and referenced to the appropriate solvent peak(s). Spin-spin coupling constants (J) are reported in Hertz (Hz).

HRMS: High resolution mass spectra were recorded on a Bruker Daltronics MicrOTOF 2 mass spectrometer (ESI).

High Performance Liquid Chromatography: Enantiomeric ratios were determined by HPLC on Agilent 1100 series or Agilent Technologies 1260 Infinity with UV detection at 280 and 254. Chiral Regis Whelk O1 and CHIRALPAK® IA with hexane:2-propanol (IPA) as the eluent for all separations, unless otherwise stated. If the temperature could be set the separation was performed at 25 °C otherwise room temperature.

UV-Vis: UV-visible spectra were recorded on an Agilent Cary Series 300 UV-Vis Spectrophotometer.

1.3 UV-visible Spectroscopy Measurements

To an oven-dried 1 mm cuvette was attached a septum and secured with Parafilm®. The cuvette was swing-purged using a Schlenk line before being washed with *n*-butyl lithium (0.1 mL) and dry THF (0.1 mL) to remove any excess water. The cuvette was then placed in an ice bath before addition of a solution of enolate (~4x10^{-4} mmol/mL formed from the corresponding amide and deprotonation with 1 equivalent of *n*-butyl lithium) in dry THF by syringe. The cuvette was kept in an ice bath until running the spectrum.

A blank sample containing *n*-butyl lithium and dry THF was ran before UV-vis measurements.

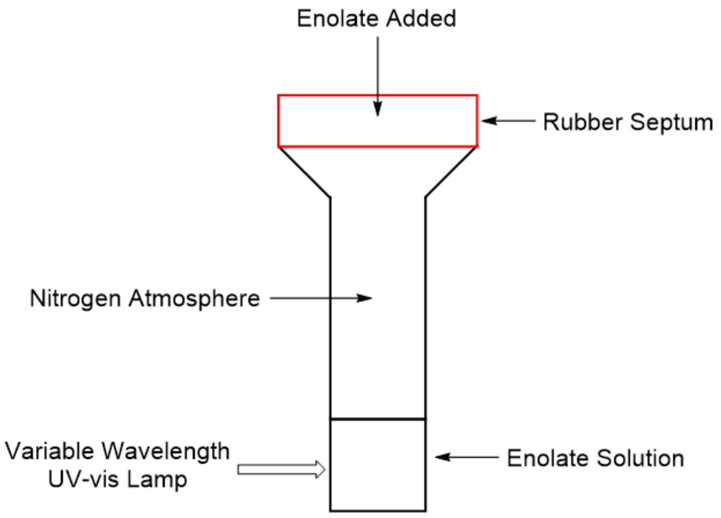

1.4 Known Starting Materials

Starting material amines[1] and *N*-benzyl-*N*-(alkyl)benzamides were prepared according to the procedures reported. Spectroscopic data for the materials prepared as described above were consistent with those reported in the literature.

Synthesis of N-(1-Phenylethyl)-1-methylethylamine

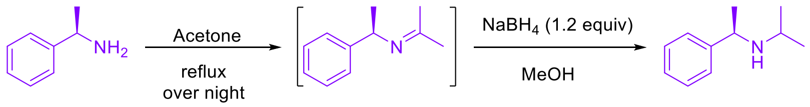

[1] S. Leleu, C. Papamicael, F. Marsais, G. Dupas, V. Levacher *Tetrahedron:Asymmetry* **2004**, 15 3919–3928

1.5 General Procedure 1: Synthesis of *rec-N*-(1-Phenylethyl)-1-methylethylamine. A solution of α-methylbenzylamine (10 g) in acetone (300 mL) was refluxed for 16 h. After evaporation of the solvent *in vacuo*, the residue was dissolved in methanol (350 mL) and sodium tetrahydroborate (3.2 g) was slowly added at 0 ºC. The solution was stirred for 2.5 h at 20 ºC. After evaporation of the solvent *in vacuo*, water (30 mL) was added, and the resulting aqueous phase was extracted with CH_2Cl_2 (3 × 30 mL). The combined organic layers were dried ($MgSO_4$) and concentrated in vacuo. Purification by flash silica chromatography (20% EtOAC/Hexane) afforded the title amine in 80% yield.

1.6 General Procedure 2: Synthesis of *(R)-N*-(1-Phenylethyl)-1-methylethylamine. A solution of *(R)*-α-methylbenzylamine in acetone (400 mL) was refluxed for 12 h. After evaporation of the solvent under vacuum, the residue was dissolved in methanol (300 mL) and sodium tetrahydroborate (1.5 g, 39.7 mmol) was slowly added at 0 °C. The solution was stirred for 2 h at 20 °C. After evaporation of the solvent *in vacuo*, water (30 mL) was added, and the resulting aqueous phase was extracted with CH_2Cl_2 (3 × 30 mL). The combined organic layers were dried ($MgSO_4$) and concentrated *in vacuo*. Purification by flash silica chromatography (20% EtOAC/Hexane) afforded the title amine in 80% yield.

1.7 General Procedure 3: Synthesis of *(S)-N*-(1-Phenylethyl)-1-methylethylamine.[2] A solution of *(S)*-α-methylbenzylamine in acetone (400 mL) was refluxed 12 h. After evaporation of the solvent under vacuum, the residue was dissolved in methanol (300 mL) and sodium tetrahydroborate (1.5 g, 39.7 mmol) was slowly added at 0 °C. The solution was stirred for 2 h at 20 °C. After evaporation of the solvent *in vacuo*, water (30 mL) was added and the resulting aqueous phase was extracted with CH_2Cl_2 (3 × 30 mL). The combined organic layers were dried ($MgSO_4$) and concentrated *in vacuo*. Purification by flash silica chromatography (20% EtOAC/Hexane) afforded the title amine in 80% yield.

[2] D. M. Mercea, M. G. Howlett, A. D. Piascik, D J. Scott, A Steven, A E. Ashley, M. J. Fuchter *Chem. Commun*., **2019**, 55, 7077

1.8 General Procedure 4: Starting material 2 synthesis[3].

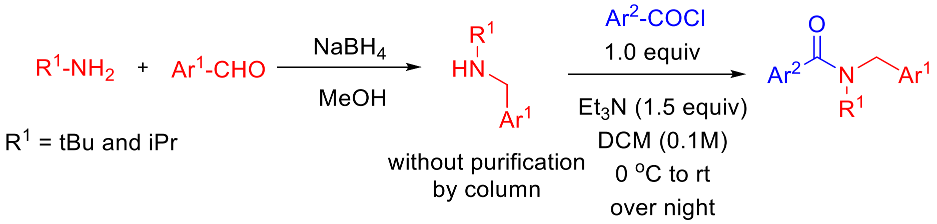

tert-Butylamine (20 mmol, 1.0 equiv.) and aldehyde (20 mmol, 1.0 equiv) were dissolved in MeOH (50 mL). The resulting mixture was stirred for 3 h at RT, and then $NaBH_4$ (1.2 equiv) was added portion-wise at 0 ºC and warmed to room temperature. After 2 h, water (30 mL) was added to the solution and then extracted with CH_2Cl_2. The combined organic layer was dried over Na_2SO_4 and then evaporated under reduced pressure to give the secondary amine. The product was used in the next step without further purification.

The acid chloride (1.0 equiv) was added dropwise to a solution of *N*-alkyl benzylamine (3.59 mmol, 1.0 equiv) and triethyl amine (1.5 equiv) in CH_2Cl_2 (0.1M) at 0 °C. The mixture was allowed to warm to room temperature overnight. The reaction was quenched with water and the aqueous layer was extracted with CH_2Cl_2 (3 × 10 mL). The combined organics were washed with brine (1 × 10 mL), dried over $MgSO_4$ and concentrated under reduced pressure to give a crude residue. Purification by flash silica chromatography (5% to 20% EtOAC/Hexane) afforded the title compound.

1.9 General Procedure 5: General procedure for the synthesis of amides 10.

The acid chloride (1.0 equiv) was added dropwise to a solution of *N*-(1-phenylethyl)propan-2-amine (2.31 mmol to 3.61 mmol) and triethyl amine (2.0 equiv) in CH_2Cl_2 (0.2M) at 0 °C. The mixture was allowed to warm to room temperature for 12 h. The reaction was quenched with water, and the aqueous layer was extracted with CH_2Cl_2 (3 × 10 mL). The combined organics were washed with brine (1 × 10 mL), dried over $MgSO_4$ and concentrated under reduced pressure to give a crude residue. Purification by flash silica chromatography (10% to 20% EtOAC/Hexane) afforded the title compound.

[3] Y. Nakagawa, S. Chanthamath, Y. Liang, K. Shibatomi, S. Iwasa *J. Org. Chem*. **2019**, 84, 2607−2618,

1.10 General Procedure 6: Enantioselective Photochemical Ring Expansion

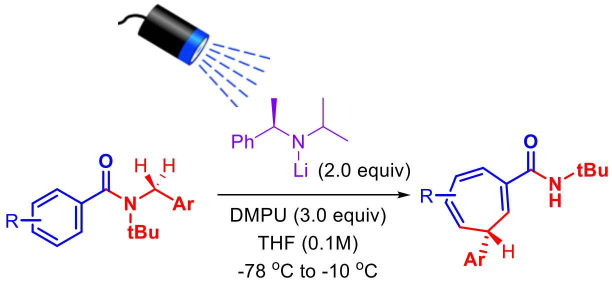

To a suspension of chiral amine (2.0 equiv.) in dry THF (0.1 M) at –78 °C was added *n*-butyl lithium (3.0 equiv, 2.5 M in hexanes), dropwise. The resulting mixture was allowed to warm to room temperature over 15 minutes, resulting in a clear yellow solution. This was then cooled down to -78 °C before a solution of amide (0.28 mmol to 0.39 mmol) in THF (0.5 mL) was added dropwise and the reaction mixture was stirred at 700 rpm and irradiated with a 40 W Kessil Tuna Blue lamp. The mixture was stirred at –78 °C for 30 minutes, then the temperature of the Cryostat was set to -10 °C and the reaction mixture was slowly allowed to warm. After a further 1 h, DMPU (3.0 equiv.) was added, and further irradiation was continued until consumption of the amide was observed by TLC. The reaction was quenched with sat. aq. NH_4Cl (15 mL), and the aqueous layer was extracted with EtOAc (3 × 10 mL). The combined organic fractions were washed with brine, dried (Na_2SO_4) and evaporated to yield a crude product, which was purified by flash chromatography, eluting with EtOAc/petrol to afford the title compound.

1.10 General Procedure 7: *rec*-Photochemical Ring Expansion

Freshly prepared LDA (2.0 equiv) in dry THF (0.1M) was cooled to –10 °C and a solution of amide (0.30 mmol) in THF (0.5 mL) and DMPU (3.0 equiv.) were sequentially added dropwise, and the reaction mixture was stirred at 700 rpm and irradiated with a 40 W Kessil Tuna Blue lamp until consumption of the amide was observed by TLC. Saturated ammonium chloride solution was added, the layers separated, and the aqueous layer was extracted with ethyl acetate. The combined organic fractions were washed with brine, dried (Na_2SO_4) and evaporated to yield a crude product, which was purified by flash chromatography, eluting with EtOAc/petrol, affording the title compound.

1.11 General Procedure 8: Stereospecific Photochemical Ring Expansion

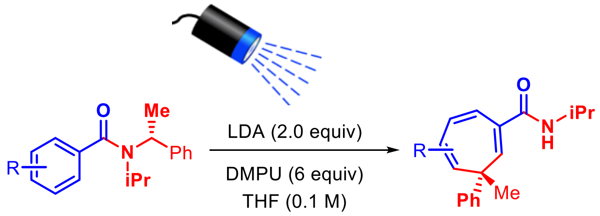

The chiral amide (0.33 mmol to 0.37 mmol) was dissolved in dry THF (0.1 M) under a nitrogen atmosphere. After cooling down to -10 °C, freshly prepared LDA (2.0 equiv) and DMPU (6.0 equiv) were sequentially added dropwise, and the reaction mixture was stirred at 700 rpm and irradiated with a 40 W Kessil Tuna Blue lamp until consumption of the amide was observed by TLC. The reaction was quenched with sat. aq. NH_4Cl (15 mL), and the aqueous layer was extracted with EtOAc (3 × 10 mL). The combined organics were washed with brine (1 × 10 mL), dried over MgSO4 and concentrated under reduced pressure to give a crude residue. Purification by flash silica chromatography (10 to 15% EtOAC/Hexane) afforded the title compound.

1.12 General Procedure 9: rec-Stereospecific Photochemical Ring Expansion

The chiral amide (0.3 mmol) was dissolved in dry THF (0.1 M) under a nitrogen atmosphere. After cooling down to -10 °C, freshly prepared LDA (2.0 equiv) and DMPU (6.0 equiv) were sequentially added dropwise, and the reaction mixture was stirred at 700 rpm and irradiated with a 40 W Kessil Tuna Blue lamp until consumption of the amide was observed by TLC. The reaction was quenched with sat. aq. NH_4Cl (15 mL), and the aqueous layer was extracted with EtOAc (3 × 10 mL). The combined organics were washed with brine (1 × 10 mL), dried over MgSO4 and concentrated under reduced pressure to give a crude residue. Purification by flash silica chromatography (10 to 15% EtOAC/Hexane) afforded the title compound.

1.13 Analytical data of amine

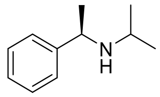

IR (neat, cm^{-1}) 3315, 2962, 1451, 1169, 759, 698, 585, 554, 517

***(R)*-*N*-(1-Phenylethyl)-1-methylethylamine**[1] **(1c).** The crude product was purified by flash silica chromatography (hexane/EtOAc = 80/20) to afford **1c** as colorless oil; $[\alpha]_D{}^{23}$ = 68 (c = 1.00 in $CHCl_3$); ^{1}**H NMR** (400 MHz, Chloroform-*d*) δ 7.39 – 7.22 (m, 5H), 3.92 (q, *J* = 6.6 Hz, 1H), 2.66 (hept, *J* = 6.3 Hz, 1H), 1.38 (d, *J* = 6.6 Hz, 3H), 1.06 (d, *J* = 6.2 Hz, 3H), 1.03 (d, *J* = 6.4 Hz, 3H). ^{13}**C NMR** (101 MHz, Chloroform-*d*) δ 146.09, 128.41, 126.75, 126.46, 55.09, 45.55, 24.88, 24.06, 22.19. **HRMS** (ESI^+) m/z calcd for $C_{11}H_{17}NNa$ $[M+Na]^+$ 186.1259, found 186.1262.

1.14 Analytical data of amides

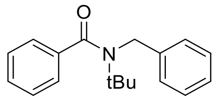

***N*-benzyl-*N*-(tert-butyl)benzamide**[4] (**2a**); By following general Procedure 4: The crude product was purified by flash silica chromatography (hexane/EtOAc = 90/10) to afford **2a** as colorless needles (720 mg, 75%); **MP**: 121-123 °C: ν_{max} /cm^{-1}(neat): 2971, 1636, 1391, 1361, 1203, 704; ^{1}**H NMR** (400 MHz, Chloroform-*d*) δ 7.39 – 7.34 (m, 2H), 7.31 – 7.23 (m, 5H), 7.22 – 7.15 (m, 3H), 4.57 (s, 2H), 1.47 (s, 9H). ^{13}**C NMR** (101 MHz, Chloroform-*d*) δ 173.95, 140.11, 139.39, 129.03, 128.59, 128.43, 127.06, 126.40, 126.29, 58.12, 51.68, 28.82. **HRMS** (ESI) calcd for [$C_{18}H_{21}NONa$] requires $[M+Na]^+$ 290.1521, found 290.1524.

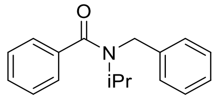

***N*-benzyl-*N*-isopropylbenzamide**[5] (**2a'**), By following general Procedure 4: The crude product was purified by flash silica chromatography (hexane/EtOAc = 90/10) to afford **2a'** as pale-yellow needles (750 mg, 83%); **MP**: 68-70 °C; ν_{max} /cm^{-1}(neat): 2976, 1626, 1411, 1341, 1175, 1060, 699; ^{1}**H NMR** (400 MHz, Chloroform-*d*) δ 7.50 – 7.11 (m, 10H), 4.67 (s, 2H), 1.33 – 0.96 (m, 6H). ^{13}**C**

---

[4] P.-L. Lagueux-Tremblay, A. Fabrikant, B. A. Arndtsen ACS Catal. **2018**, 8, 5350−5354

[5] D. Lu, H.-X. Wei, J. Zhang, Y. Gu, P. Osenkowski, W. Ye, D. J. Selkoe, M. S. Wolfe, C. E. Augelli-Szafran *Bioorg. Med. Chem. Lett.* **2016**, *26*, 2129–2132

NMR (101 MHz, Chloroform-*d*) δ 172.33, 139.52, 137.48, 129.31, 128.61, 128.52, 127.12, 126.91, 126.33, 50.84, 43.39, 21.49. **HRMS** (ESI) calcd for [$C_{17}H_{19}NONa$] requires [M+Na]+ 276.1359, found 276.1364.

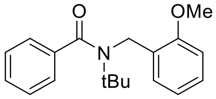

***N*-(tert-butyl)-*N*-(2-methoxybenzyl)benzamide** (**2b**), By following general Procedure 4: The crude product was purified by flash silica chromatography (hexane/EtOAc = 85/15) to afford **2b** as colorless needles (700 mg, 66%); **MP**: 122-124 ° C; ν_{max} /cm^{-1}(neat): 2976, 1628, 1490, 1461, 1391, 1239, 1198, 1025, 752, 705; **^{1}H NMR** (400 MHz, Chloroform-*d*) δ 7.48 – 7.36 (m, 3H), 7.32 – 7.21 (m, 4H), 7.02 (t, *J* = 7.3 Hz, 1H), 6.80 (d, *J* = 8.1 Hz, 1H), 4.59 (s, 2H), 3.74 (s, 3H), 1.53 (s, 9H). **^{13}C NMR** (101 MHz, Chloroform-*d*) δ 174.14, 155.96, 139.51, 128.80, 128.18, 128.11, 127.88, 127.70, 126.06, 120.25, 110.10, 57.88, 55.13, 46.57, 28.54. **HRMS** (ESI) calcd for [$C_{19}H_{23}NO_2Na$] requires [M+Na]+ 320.1621, found 320.1623.

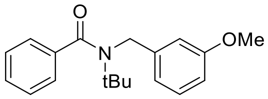

***N*-(*tert*-butyl)-*N*-(3-methoxybenzyl)benzamide** (**2c**), By following general Procedure 4: The crude product was purified by flash silica chromatography (hexane/EtOAc = 85/15) to afford **2c** as colorless needles (740 mg, 69%); **MP**: 82-84 ° C; ν_{max} /cm^{-1}(neat): 2971, 1632, 1488, 1388, 1252, 1198, 1047, 971, 750, 698, 655; **^{1}H NMR** (400 MHz, Chloroform-*d*) δ 7.45 – 7.39 (m, 2H), 7.30 (q, *J* = 3.7 Hz, 3H), 7.24 (dd, *J* = 8.9, 7.6 Hz, 1H), 6.83 (d, *J* = 7.7 Hz, 1H), 6.80 – 6.75 (m, 2H), 4.59 (s, 2H), 3.79 (s, 3H), 1.54 (s, 9H). **^{13}C NMR** (101 MHz, Chloroform-*d*) δ 173.80, 159.81, 141.79, 139.29, 129.59, 128.97, 128.38, 126.17, 118.59, 112.26, 112.05, 58.10, 55.18, 51.56, 28.74. **HRMS** (ESI) calcd for [$C_{19}H_{23}NO_2Na$] requires [M+Na]+ 320.1621, found 320.1628.

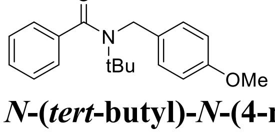

***N*-(*tert*-butyl)-*N*-(4-methoxybenzyl)benzamide** (**2d**), By following general Procedure 4: The crude product was purified by flash silica chromatography (hexane/EtOAc = 85/15) to afford **2d** as colorless needles (780 mg, 73%); **MP**: 122-124 ° C; ν_{max} /cm^{-1}(neat): 2971, 1630, 1511, 1388, 1243, 1032, 750, 699; **^{1}H NMR** (400 MHz, Chloroform-*d*) δ 7.44 – 7.37 (m, 2H), 7.31 – 7.26 (m, 3H), 7.11 (d, *J* = 8.8 Hz, 2H), 6.85 (d, *J* = 8.7 Hz, 2H), 4.54 (s, 2H), 3.78 (s, 3H), 1.49 (s, 9H). **^{13}C**

NMR (101 MHz, $CDCl_3$) δ 173.86, 158.61, 139.33, 131.84, 128.96, 128.35, 127.45, 126.28, 113.92, 57.97, 55.28, 51.01, 28.74. **HRMS** (ESI) calcd for [$C_{19}H_{23}NO_2Na$] requires [M+Na]+ 320.1626, found 320.1632.

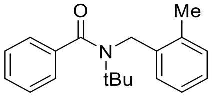

***N*-(*tert*-butyl)-*N*-(2-methylbenzyl)benzamide** (**2e**), By following general Procedure 4: The crude product was purified by flash silica chromatography (hexane/EtOAc = 90/10) to afford **2e** as colorless needles (630 mg, 62%); **MP**: 96-98 ° C; ν_{max} /cm^{-1}(neat): 2971, 1631, 1393, 1197, 1066, 699; ^{1}**H NMR** (400 MHz, Chloroform-*d*) δ 7.48 (d, *J* = 7.7 Hz, 1H), 7.32 (dd, *J* = 7.8, 1.5 Hz, 2H), 7.29 – 7.17 (m, 4H), 7.14 (t, *J* = 7.4 Hz, 1H), 7.05 (d, *J* = 7.4 Hz, 1H), 4.46 (s, 2H), 2.05 (s, 3H), 1.53 (s, 9H). ^{13}**C NMR** (101 MHz, CHLOROFORM-*D*) δ 174.04, 139.32, 137.96, 133.86, 130.38, 128.94, 128.29, 126.75, 126.37, 126.05, 125.87, 58.15, 49.12, 28.62, 19.00. **HRMS** (ESI) calcd for [$C_{19}H_{23}NONa$] requires [M+Na]+ 304.1672, found 304.1665.

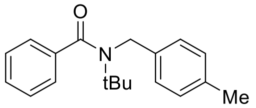

***N*-(*tert*-butyl)-*N*-(4-methylbenzyl)benzamide** (**2f**), By following general Procedure 4: The crude product was purified by flash silica chromatography (hexane/EtOAc = 90/10) to afford **2f** as colorless needles (780 mg, 77%); **MP**: 72-74 ° C; ν_{max} /cm^{-1}(neat): 2971, 1631, 1385, 1199, 1066, 790, 654, 698; ^{1}**H NMR** (400 MHz, Chloroform-*d*) δ 7.42 – 7.36 (m, 2H), 7.29 – 7.23 (m, 3H), 7.13 – 7.05 (m, 4H), 4.55 (s, 2H), 2.31 (s, 3H), 1.49 (s, 9H). ^{13}**C NMR** (101 MHz, CHLOROFORM-*D*) δ 173.93, 139.45, 137.02, 136.66, 129.27, 128.99, 128.42, 126.32, 58.08, 51.46, 28.82, 21.11. **HRMS** (ESI) calcd for [$C_{19}H_{24}NO$] requires [M+ H]+ 282.1852, found 282.1862.

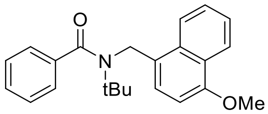

N-(tert-butyl)-N-((4-methoxynaphthalen-1-yl)methyl)benzamide (**2g**), By following general Procedure 4: The crude product was purified by flash silica chromatography (hexane/EtOAc = 80/20) to afford **2g** as colorless needles (805 mg, 64%); **MP**: 160-162 ° C; ν_{max} /cm^{-1}(neat): 2971, 1621, 1586, 1384, 1265, 1195, 1089, 697, 749; ^{1}**H NMR** (400 MHz, Chloroform-*d*) δ 8.35 – 8.27 (m, 1H), 7.67 – 7.62 (m, 1H), 7.60 (d, *J* = 8.0 Hz, 1H), 7.50 – 7.42 (m, 2H), 7.40 (dd, *J* = 7.9, 1.6

Hz, 2H), 7.24 – 7.13 (m, 3H), 6.90 (d, *J* = 8.0 Hz, 1H), 4.91 (s, 2H), 4.03 (s, 3H), 1.58 (s, 9H). **^{13}C NMR** (101 MHz, Chloroform-*d*) δ 174.07, 154.73, 139.25, 130.60, 128.87, 128.17, 126.90, 126.68, 125.72, 125.63, 125.13, 124.25, 122.91, 121.61, 103.04, 58.25, 55.52, 48.56, 28.59. **HRMS** (ESI) calcd for [$C_{23}H_{25}NO_2Na$] requires [M+Na]+ 370.1777, found 370.1781.

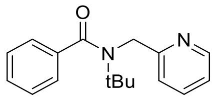

***N*-(tert-butyl)-*N*-(pyridin-2-ylmethyl)benzamide** (**2h**), By following general Procedure 4: The crude product was purified by flash silica chromatography (hexane/EtOAc = 80/20) to afford **2h** as colorless needles (630 mg, 65%); **MP**: 94-96 ° C; ν_{max} /cm^{-1}(neat): 2971, 1633, 1388, 1197, 974, 749, 699; **^{1}H NMR** (400 MHz, Chloroform-*d*) δ 8.48 (ddd, *J* = 4.9, 1.8, 0.9 Hz, 1H), 7.64 (td, *J* = 7.7, 1.8 Hz, 1H), 7.40 – 7.35 (m, 2H), 7.30 – 7.21 (m, 4H), 7.14 – 7.09 (m, 1H), 4.66 (s, 2H), 1.46 (s, 9H). **^{13}C NMR** (101 MHz, CHLOROFORM-*D*) δ 174.04, 159.66, 149.37, 139.37, 136.53, 129.03, 128.41, 126.24, 122.08, 121.11, 58.07, 53.46, 28.80. **HRMS** (ESI) calcd for [$C_{17}H_{20}N_2ONa$] requires [M+Na]+ 291.1473, found 291.1475.

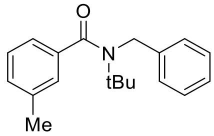

***N*-benzyl-*N*-(tert-butyl)-3-methylbenzamide** (**2i**), By following general Procedure 4: The crude product was purified by flash silica chromatography (hexane/EtOAc = 90/10) to afford **2i** as colorless needles (650 mg, 71%); **MP**: 93-95° C; ν_{max} /cm^{-1}(neat): 2967, 1633, 1386, 1358, 1195, 973, 739, 701; **^{1}H NMR** (400 MHz, Chloroform-*d*) δ 7.33 – 7.27 (m, 2H), 7.24 – 7.15 (m, 5H), 7.15 – 7.06 (m, 2H), 4.59 (s, 2H), 2.26 (s, 3H), 1.49 (s, 9H). **^{13}C NMR** (101 MHz, Chloroform-*d*) δ 174.16, 140.20, 139.33, 138.20, 129.74, 128.54, 128.27, 127.05, 127.02, 126.44, 123.19, 58.06, 51.72, 28.82, 21.39. **HRMS** (ESI) calcd for [$C_{19}H_{24}NO$] requires [M+H] $^{+}$ 282.1858, found 282.1864.

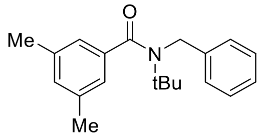

***N*-benzyl-*N*-(tert-butyl)-3,5-dimethylbenzamide** (**2j**), By following general Procedure 4: The crude product was purified by flash silica chromatography (hexane/EtOAc = 90/10) to afford **2j** as colorless needles (520 mg, 59%); **MP**: 92-94 ° C; ν_{max} /cm^{-1}(neat): 2969, 1633, 1390, 1358, 1196, 855, 746; **^{1}H NMR** (400 MHz, Chloroform-*d*) δ 7.34 – 7.28 (m, 2H), 7.22 (t, *J* = 7.3 Hz,

3H), 6.99 (s, 2H), 6.93 – 6.89 (m, 1H), 4.60 (s, 2H), 2.24 – 2.21 (m, 6H), 1.49 (s, 9H). **^{13}C NMR** (101 MHz, Chloroform-*d*) δ 174.34, 140.29, 139.31, 138.00, 130.55, 128.49, 126.97, 126.49, 123.99, 57.98, 51.76, 28.82, 21.26. **HRMS** (ESI) calcd for [$C_{20}H_{25}NONa$] requires $[M+Na]^+$ 318.1828, found 318.1843.

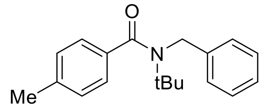

***N*-benzyl-*N*-(tert-butyl)-4-methylbenzamide** (**2k**), By following general Procedure 4: The crude product was purified by flash silica chromatography (hexane/EtOAc = 90/10) to afford **2k** as colorless needles (690 mg, 75%); **MP**: 123-125 ° C; ν_{max} /cm^{-1}(neat): 2971, 1631, 1384, 1358, 1199, 1074, 958. 829, 744, 701; **^{1}H NMR** (400 MHz, Chloroform-*d*) δ 7.34 – 7.28 (m, 4H), 7.25 – 7.19 (m, 3H), 7.10 – 7.05 (m, 2H), 4.61 (s, 2H), 2.29 (s, 3H), 1.49 (s, 9H). **^{13}C NMR** (101 MHz, Chloroform-*d*) δ 174.18, 140.26, 139.04, 136.50, 129.02, 128.56, 127.00, 126.42, 126.39, 58.03, 51.75, 28.83, 21.34. **HRMS** (ESI) calcd for [$C_{19}H_{23}NONa$] requires $[M + Na]^+$ 304.1672, found 304.1686.

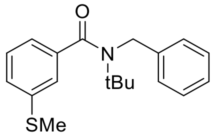

***N*-benzyl-*N*-(*tert*-butyl)-3-(methylthio)benzamide** (**2l**), By following general Procedure 4: The crude product was purified by flash silica chromatography (hexane/EtOAc = 85/15) to afford **2l** as pale yellow needles (500 mg, 59%); **MP**: 101-103 ° C; ν_{max} /cm^{-1}(neat): 2971, 1633, 1384, 1358, 1253, 1198, 977, 746; **^{1}H NMR** (400 MHz, Chloroform-*d*) δ 7.35 – 7.29 (m, 2H), 7.26 – 7.19 (m, 4H), 7.19 – 7.13 (m, 3H), 4.58 (s, 2H), 2.30 (s, 3H), 1.51 (s, 9H). **^{13}C NMR** (101 MHz, $CDCl_3$) δ 173.29, 140.03, 138.93, 128.70, 128.56, 127.22, 127.01, 126.22, 123.60, 122.79, 58.20, 51.56, 28.67, 15.46. **HRMS** (ESI) calcd for [$C_{19}H_{23}NOSNa$] requires [M+Na]+ 336.1393, found 336.1395.

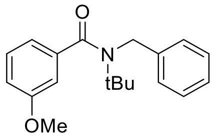

***N*-benzyl-*N*-(tert-butyl)-3-methoxybenzamide**[5] (**2m**), By following general Procedure 4: The crude product was purified by flash silica chromatography (hexane/EtOAc = 85/15) to afford **2m** as colorless needles (750 mg, 85%); **MP**: 92-93 ° C; ν_{max} /cm^{-1}(neat): 2971, 1633, 1452, 1391, 1358, 1197, 1046, 699, 744; **^{1}H NMR** (400 MHz, Chloroform-*d*) δ 7.35 – 7.28 (m, 2H), 7.25 –

7.16 (m, 4H), 6.98 (dt, J = 7.5, 1.1 Hz, 1H), 6.91 – 6.87 (m, 1H), 6.85 – 6.80 (m, 1H), 4.60 (s, 2H), 3.65 (s, 3H), 1.51 (s, 9H). **^{13}C NMR** (101 MHz, Chloroform-*d*) δ 173.58, 159.36, 140.47, 140.18, 129.50, 128.51, 126.93, 126.27, 118.43, 115.31, 111.18, 58.10, 55.17, 51.58, 28.69. **HRMS** (ESI) calcd for [$C_{19}H_{23}NO_2Na$] requires [M+Na]+ 297.1721, found 320.1625.

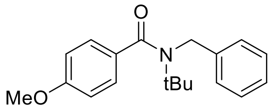

***N*-benzyl-*N*-(tert-butyl)-4-methoxybenzamide**[6] (**2n**), By following general Procedure 4: The crude product was purified by flash silica chromatography (hexane/EtOAc = 85/15) to afford **2n**as colorless needles (710 mg, 81%); **MP**: 130-132 ° C; ν_{max} /cm^{-1}(neat): 2970, 1630, 1382, 1246, 1027, 838, 745, 702; **^{1}H NMR** (400 MHz, Chloroform-*d*) δ 7.39 – 7.34 (m, 2H), 7.30 (t, J = 7.4 Hz, 2H), 7.21 (t, J = 7.3 Hz, 3H), 6.80 – 6.75 (m, 2H), 4.62 (s, 2H), 3.75 (s, 3H), 1.47 (s, 9H). **^{13}C NMR** (101 MHz, Chloroform-*d*) δ 173.96, 160.31, 140.37, 131.68, 128.57, 128.32, 127.01, 126.46, 113.66, 58.01, 55.34, 51.85, 28.83. **HRMS** (ESI) calcd for [$C_{19}H_{23}NO_2Na$] requires [M+Na]+ 320.1621, found 320.1609.

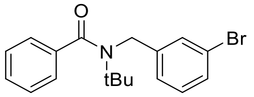

***N*-(3-bromobenzyl)-*N*-(*tert*-butyl)benzamide** (**2o**), By following general Procedure 4: The crude product was purified by flash silica chromatography (hexane/EtOAc = 90/10) to afford **2o** as colorless needles (600 mg, 48%); **MP**: 116-118 ° C; ν_{max} /cm^{-1}(neat): 2971, 1632, 1383, 1195, 1069, 968, 774; **^{1}H NMR** (400 MHz, Chloroform-*d*) δ 7.38 – 7.34 (m, 3H), 7.33 – 7.26 (m, 4H), 7.22 – 7.12 (m, 2H), 4.56 (s, 2H), 1.50 (s, 9H). **^{13}C NMR** (101 MHz, CHLOROFORM-*D*) δ 173.93, 142.58, 139.06, 130.24, 130.19, 129.43, 129.21, 128.54, 126.11, 124.95, 122.81, 58.28, 51.10, 28.86. **HRMS** (ESI) calcd for [$C_{18}H_{20}BrNONa$] requires [M+Na]+ 368.0620, found 368.0615.

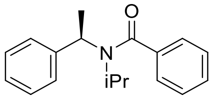

***(R)*-*N*-isopropyl-*N*-(1-phenylethyl)benzamide** (**10a**), By following general procedure 5: The acid chloride (1.0 equiv) was added dropwise to a solution of *N*-(1-phenylethyl)propan-2-amine

[6] J. Clayden, K. Tchabanenko, S. A. Yasin, M. D. Turnbull. *Synlett* **2001** (2), 302-304.

(3.61 mmol) and triethyl amine (2.0 equiv) in CH_2Cl_2 (0.2M) at 0 °C. The mixture was allowed to warm to room temperature for 12 h. The reaction was quenched with water, and the aqueous layer was extracted with CH_2Cl_2 (3 × 10 mL). The combined organics were washed with brine (1 × 10 mL), dried over $MgSO_4$ and concentrated under reduced pressure to give a crude residue. The crude product was purified by flash silica chromatography (hexane/EtOAc = 90/10) to afford **10a** as colorless oil (705 mg, 73%); ν_{max} /cm^{-1}(neat): 2976, 1630, 1439, 1333, 854, 756; **^{1}H NMR** (400 MHz, Chloroform-d) δ 7.47 – 7.37 (m, 5H), 7.35 (dd, J = 7.2, 3.2 Hz, 4H), 7.27 (d, J = 4.3 Hz, 1H), 4.91 (d, J = 6.2 Hz, 1H), 3.38 (brs s, 1H), 1.65 (brs s, 3H), 1.45 (brs s, 3H), 1.14 (s, 3H). **13C NMR** (101 MHz, Chloroform-d) δ 171.45, 138.81, 134.63, 130.67, 128.98, 128.89, 128.70, 128.38, 127.30, 125.88, 59.93 (br), 39.12 (br), 21.15 (br), 20.33 (br), 17.70 (br). **HRMS** (ESI) calcd for [$C_{18}H_{21}NONa$] requires [M+Na]+ 290.1521, found 290.1520.

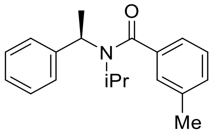

***(R)*-*N*-isopropyl-3-methyl-*N*-(1-phenylethyl)benzamide** (**10b**), By following general procedure 5: The acid chloride (1.0 equiv) was added dropwise to a solution of *N*-(1-phenylethyl)propan-2-amine (3.26 mmol) and triethyl amine (2.0 equiv) in CH_2Cl_2 (0.2M) at 0 °C. The mixture was allowed to warm to room temperature for 12 h. The reaction was quenched with water, and the aqueous layer was extracted with CH_2Cl_2 (3 × 10 mL). The combined organics were washed with brine (1 × 10 mL), dried over $MgSO_4$ and concentrated under reduced pressure to give a crude residue. The crude product was purified by flash silica chromatography (hexane/EtOAc = 90/10) to afford **10b** as colorless oil (690 mg, 75%); ν_{max} /cm^{-1}(neat): 2971, 1632, 1435, 1353, 756, 699; **^{1}H NMR** (400 MHz, Chloroform-*d*) δ 7.33 (d, *J* = 4.7 Hz, 4H), 7.28 – 7.19 (m, 4H), 7.19 – 7.15 (m, 1H), 4.90 (br s, 1H), 3.36 (br s, 1H), 2.37 (s, 3H), 1.64 (br s, 3H), 1.44 (br s, 3H), 1.14 (d, *J* = 6.7 Hz, 3H). **^{13}C NMR** (101 MHz, Chloroform-*d*) δ 171.65, 138.80, 138.54, 129.60, 128.53, 128.36, 127.32, 126.53, 122.77, 56.07 (br), 48.55 (br), 21.53, 21.14 (br), 20.33 (br), 17.69 (br). **HRMS** (ESI) calcd for [$C_{19}H_{23}NONa$] requires [M+Na]+ 304.1677, found 304.1683.

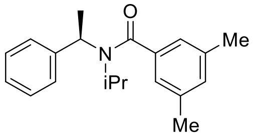

***(R)*-*N*-isopropyl-3,5-dimethyl-*N*-(1-phenylethyl)benzamide** (**10c**), By following general procedure 5: The acid chloride (1.0 equiv) was added dropwise to a solution of *N*-(1-phenylethyl)propan-2-amine (3.00 mmol) and triethyl amine (2.0 equiv) in CH_2Cl_2 (0.2M) at 0 °C. The mixture was allowed to warm to room temperature for 12 h. The reaction was quenched with water, and the aqueous layer was extracted with CH_2Cl_2 (3 × 10 mL). The combined organics were washed with brine (1 × 10 mL), dried over $MgSO_4$ and concentrated under reduced pressure to give a crude residue. The crude product was purified by flash silica chromatography (hexane/EtOAc = 90/10) to afford **4c** as colorless needles (630 mg, 71%); **MP:** 81-83 °C; ν_{max} /cm^{-1}(neat): 2976, 1629, 1435, 1333, 854, 756, 698; **^{1}H NMR** (400 MHz, Chloroform-*d*) δ 7.34 (d, *J* = 4.5 Hz, 4H), 7.29 – 7.22 (m, 1H), 7.02 (d, *J* = 11.7 Hz, 3H), 4.93 (br s, 1H), 3.39 (br s, 1H), 2.33 (s, 6H), 1.65 (br s, 3H), 1.45 (br s, 3H), 1.14 (d, *J* = 6.7 Hz, 3H). **^{13}C NMR** (101 MHz, $CDCl_3$) δ 171.74, 138.75, 138.23, 130.31, 128.25, 127.26, 123.36, 60.40 (br), 49.88 (br), 21.33, 21.05 (br), 20.27 (br), 14.21. **HRMS** (ESI) calcd for [$C_{20}H_{25}NONa$] requires [M+Na]$^+$ 318.1824, found 318.1828.

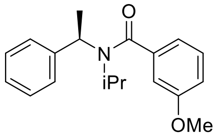

***(R)*-*N*-isopropyl-3-methoxy-*N*-(1-phenylethyl)benzamide** (**10d**), By following general procedure 5: The acid chloride (1.0 equiv) was added dropwise to a solution of *N*-(1-phenylethyl)propan-2-amine (2.95 mmol) and triethyl amine (2.0 equiv) in CH_2Cl_2 (0.2M) at 0 °C. The mixture was allowed to warm to room temperature for 12 h. The reaction was quenched with water, and the aqueous layer was extracted with CH_2Cl_2 (3 × 10 mL). The combined organics were washed with brine (1 × 10 mL), dried over $MgSO_4$ and concentrated under reduced pressure to give a crude residue. The crude product was purified by flash silica chromatography (hexane/EtOAc = 90/10) to afford **10d** as colorless needles (685 mg, 78%); **MP:** 78-80 °C; ν_{max} /cm^{-1}(neat): 2971, 1629, 1433, 1351, 1236, 749; **^{1}H NMR** (400 MHz, Chloroform-*d*) δ 7.37 – 7.29 (m, 5H), 7.27 – 7.20 (m, 1H), 7.00 (d, *J* = 7.5 Hz, 1H), 6.96 (s, 1H), 6.90 (ddd, *J* = 8.3, 2.6, 0.9 Hz, 1H), 4.89 (br s, 1H), 3.81 (s, 3H), 3.37 (br s, 1H), 1.63 (br s, 3H), 1.44 (br s, 3H), 1.14 (d, *J* = 6.7 Hz, 3H). **^{13}C NMR** (101 MHz, Chloroform-*d*) δ 171.14, 159.81, 140.03, 129.87, 128.38, 127.31, 118.02, 114.72, 111.45, 56.97 (br), 55.45, 48.08 (br), 21.13 (br), 20.25 (br), 17.64 (br). **HRMS** (ESI) calcd for [$C_{19}H_{23}NO_2Na$] requires [M+Na]+ 320.1621, found 320.1626.

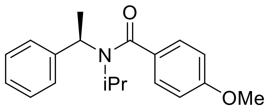

***(R)*-*N*-isopropyl-4-methoxy-*N*-(1-phenylethyl)benzamide** (**10e**), By following general procedure 5: The acid chloride (1.0 equiv) was added dropwise to a solution of *N*-(1-phenylethyl)propan-2-amine (2.95 mmol) and triethyl amine (2.0 equiv) in CH_2Cl_2 (0.2M) at 0 °C. The mixture was allowed to warm to room temperature for 12 h. The reaction was quenched with water, and the aqueous layer was extracted with CH_2Cl_2 (3 × 10 mL). The combined organics were washed with brine (1 × 10 mL), dried over $MgSO_4$ and concentrated under reduced pressure to give a crude residue. The crude product was purified by flash silica chromatography (hexane/EtOAc = 90/10) to afford **10e** as colorless needles (640 mg, 73%); **MP:** 84-86 °C; ν_{max} /cm^{-1}(neat): 2976, 1625, 1511, 1432, 1360, 1246, 1174, 1024; **^{1}H NMR** (400 MHz, Chloroform-*d*) δ 7.39 (d, *J* = 8.8 Hz, 2H), 7.31 (d, *J* = 4.4 Hz, 4H), 7.24 (q, *J* = 4.6 Hz, 1H), 6.90 (d, *J* = 8.8 Hz, 2H), 4.96 (q, *J* = 6.7 Hz, 1H), 3.80 (s, 3H), 3.46 (s, 1H), 1.65 (d, *J* = 6.8 Hz, 3H), 1.42 (d, *J* = 6.6 Hz, 3H), 1.13 (d, *J* = 6.7 Hz, 3H). **^{13}C NMR** (101 MHz, Chloroform-*d*) δ 171.45, 160.12, 131.13, 128.36, 127.73, 127.36, 127.26, 113.96, 60.48 (br), 55.39, 48.51 (br), 21.21 (br), 20.48 (br), 17.88 (br). **HRMS** (ESI) calcd for [$C_{19}H_{23}NO_2Na$] requires [M+Na]+ 320.1621, found 320.1619.

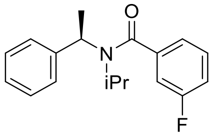

***(R)*-3-fluoro-*N*-isopropyl-*N*-(1-phenylethyl)benzamide (10f)** By following general procedure 5: The acid chloride (1.0 equiv) was added dropwise to a solution of *N*-(1-phenylethyl)propan-2-amine (3.20 mmol) and triethyl amine (2.0 equiv) in CH_2Cl_2 (0.2M) at 0 °C. The mixture was allowed to warm to room temperature for 12 h. The reaction was quenched with water, and the aqueous layer was extracted with CH_2Cl_2 (3 × 10 mL). The combined organics were washed with brine (1 × 10 mL), dried over $MgSO_4$ and concentrated under reduced pressure to give a crude residue. The crude product was purified by flash silica chromatography (hexane/EtOAc = 90/10) to afford **10f** as colorless oil (540 mg, 59%); ν_{max} /cm^{-1}(neat): 2972, 1629, 1583, 1441, 1327, 1055, 785, 749, 697; **^{1}H NMR** (400 MHz, Chloroform-*d*) δ 7.45 – 7.33 (m, 5H), 7.33 – 7.27 (m, 1H), 7.25 (d, *J* = 7.6 Hz, 1H), 7.18 (d, *J* = 8.8 Hz, 1H), 7.11 (tdd, *J* = 8.6, 2.6, 1.0 Hz, 1H), 4.90 (d, *J* = 6.3 Hz, 1H), 3.44 (br s, 1H), 1.69 (br s, 3H), 1.48 (br s, 3H), 1.16 (s, 3H). **^{13}C NMR** (101 MHz,

Chloroform-*d*) δ 169.76, 162.69(d $^1J_{CF}$ = 248.46 Hz), 140.67 (d, $^3J_{CF}$ = 7.49 Hz), 130.50 (d, $^3J_{CF}$ = 7.07 Hz), 128.39, 127.50, 127.18, 121.50 (d, $^4J_{CF}$ = 3.03 Hz), 115.85 (d, $^2J_{CF}$ = 21.21 Hz), 113.33 (d, $^2J_{CF}$ = 23.23 Hz), 56.27 (br), 48.79 (br), 21.02 (br), 20.17 (br), 17.69 (br). **HRMS** (ESI) calcd for [$C_{18}H_{20}FNONa$] requires [M+Na]+ 308.1427, found 308.1431.

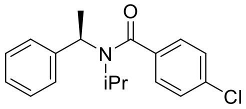

***(R)*-4-chloro-*N*-isopropyl-*N*-(1-phenylethyl)benzamide (10g)** By following general procedure 5: The acid chloride (1.0 equiv) was added dropwise to a solution of *N*-(1-phenylethyl)propan-2-amine (2.92 mmol) and triethyl amine (2.0 equiv) in CH_2Cl_2 (0.2M) at 0 °C. The mixture was allowed to warm to room temperature for 12 h. The reaction was quenched with water, and the aqueous layer was extracted with CH_2Cl_2 (3 × 10 mL). The combined organics were washed with brine (1 × 10 mL), dried over $MgSO_4$ and concentrated under reduced pressure to give a crude residue. The crude product was purified by flash silica chromatography (hexane/EtOAc = 90/10) to afford **10g** as colorless oil (590 mg, 67%); ν_{max} /cm^{-1}(neat): 2971, 1630, 1565, 1443, 784, 749, 699; **^{1}H NMR** (400 MHz, Chloroform-*d*) δ 8.71 – 8.61 (m, 2H), 7.37 – 7.20 (m, 7H), 4.77 (q, *J* = 6.9 Hz, 1H), 3.31 (s, 1H), 1.78 – 1.26 (m, 6H), 1.13 (d, *J* = 6.8 Hz, 3H). **^{13}C NMR** (101 MHz, Chloroform-*d*) δ 168.72, 150.46, 145.99, 128.55, 127.22, 120.38, 57.15 (br), 48.34 (br), 21.01 (br), 19.87 (br), 17.48 (br). **HRMS** (ESI) calcd for [$C_{18}H_{20}ClNONa$] requires [M+Na]+ 324.1131, found 324.1136.

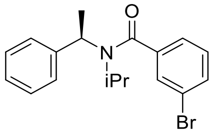

***(R)*-3-bromo-*N*-isopropyl-*N*-(1-phenylethyl)benzamide (10h)** By following general procedure 5: The acid chloride (1.0 equiv) was added dropwise to a solution of *N*-(1-phenylethyl)propan-2-amine (2.31 mmol) and triethyl amine (2.0 equiv) in CH_2Cl_2 (0.2M) at 0 °C. The mixture was allowed to warm to room temperature for 12 h. The reaction was quenched with water, and the aqueous layer was extracted with CH_2Cl_2 (3 × 10 mL). The combined organics were washed with brine (1 × 10 mL), dried over $MgSO_4$ and concentrated under reduced pressure to give a crude residue. The crude product was purified by flash silica chromatography (hexane/EtOAc = 90/10) to afford **10h** as colorless oil (554 mg, 69%); ν_{max} /cm^{-1}(neat): 2972, 1628, 1435, 1361, 1322,

1065, 796, 723, 695, 657; **^{1}H NMR** (400 MHz, Chloroform-*d*) δ 7.60 (s, 1H), 7.54 (ddd, *J* = 7.9, 2.0, 1.2 Hz, 1H), 7.43 – 7.26 (m, 7H), 4.95 – 4.78 (m, 1H), 3.45 (br s, 1H), 1.69 (br s, 3H), 1.47 (br s, 3H), 1.17 (d, *J* = 6.7 Hz, 3H). **^{13}C NMR** (101 MHz, $CDCl_3$) δ 169.52, 140.54, 131.96, 130.27, 128.97, 128.40, 127.18, 124.35, 122.79, 65.86 (br), 48.48 (br), 20.99 (br), 20.23 (br), 17.78 (br). **HRMS** (ESI) calcd for [$C_{18}H_{20}BrNONa$] requires $[M+Na]^+$ 368.0626, found 368.0629.

1.15 X-ray crystallography Data

X-ray Structure of 4k

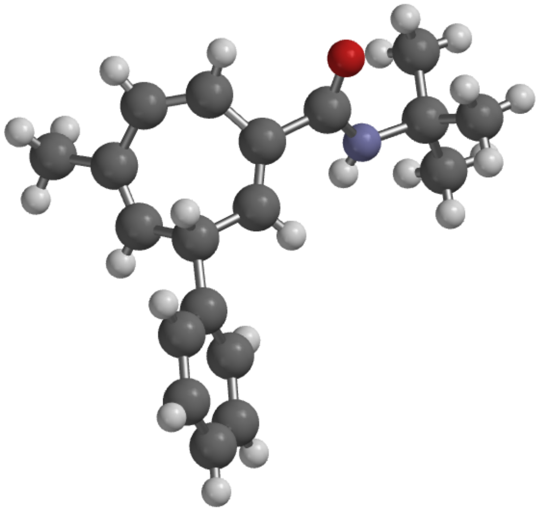

Colorless crystals of **4k** were grown from a hexanes/Aectone solution by slow volatilization. CCDC: 2083306

Bond precision:	C-C = 0.0020 A		Wavelength=1.54178
Cell:	a=5.8270(3)	b=16.2460(7)	c=16.8144(8)
	alpha=90	beta=90	gamma=90
Temperature:	100 K		
	Calculated		Reported
Volume	1591.74(13)		1591.74(13)
Space group	P 21 21 21		P 21 21 21
Hall group	P 2ac 2ab		P 2ac 2ab
Moiety formula	C19 H23 N O		C19 H23 N O
Sum formula	C19 H23 N O		C19 H23 N O
Mr	281.38		281.38
Dx,g cm-3	1.174		1.174

Z	4		4
Mu (mm-1)	0.553		0.553
F000	608.0		608.0
F000’	609.61		
h,k,lmax	7,19,20		7,19,20
Nref	2907[1707]		2906
Tmin,Tmax	0.923,0.967		0.511,0.754
Tmin’	0.802		
Correction method= # Reported T Limits:			Tmin=0.511 Tmax=0.754
AbsCorr =	MULTI-SCAN		
Data completeness=		1.70/1.00	
heta(max)=	68.236		
R(reflections)= 0.0261(2895)		wR2(reflections)= 0.0656(2906)	
S = 1.098		Npar= 198	

X-ray Structure of 4l

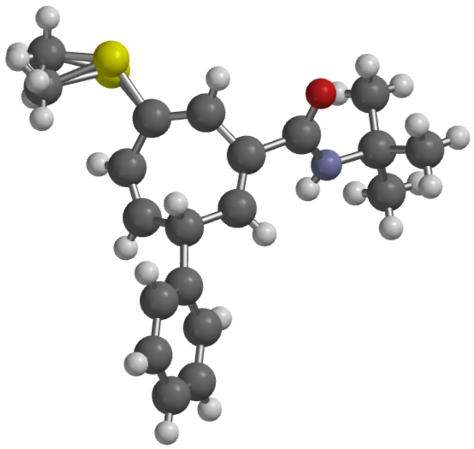

Colorless crystals of **4l** were grown from a hexanes/EtOAc solution by slow volatilization. CCDC: 2083307

Bond precision:	C-C = 0.0022 A		Wavelength=1.54178
Cell:	a=5.8465(1)	b=16.2467(4)	c=17.6742(4)
	alpha=90	beta=90	gamma=90
Temperature:	100 K		

	Calculated	Reported
Volume	1678.81(6)	1678.81(6)
Space group	P 21 21 21	P 21 21 21
Hall group	P 2ac 2ab	P 2ac 2ab
Moiety formula	C19 H23 N O S	C19 H23 N O S
Sum formula	C19 H23 N O S	C19 H23 N O S
Mr	313.44	313.44
Dx,g cm-3	1.240	1.240
Z	4	4
Mu (mm-1)	1.708	1.708
F000	672.0	672.0
F000’	674.95	674.95
h,k,lmax	7,19,21	7,19,21
Nref	3180 [1862]	3179
Tmin,Tmax	0.782,0.872	0.284,0.754
Tmin’	0.537	

Correction method= # Reported T Limits: Tmin=0.284 Tmax=0.754

AbsCorr = MULTI-SCAN

Data completeness=1.71/1.00 Theta(max)= 70.060

R(reflections)= 0.0242(3167) wR2(reflections)= 0.0624(3179)

S = 1.084 Npar= 227

X-ray Structure of 11d

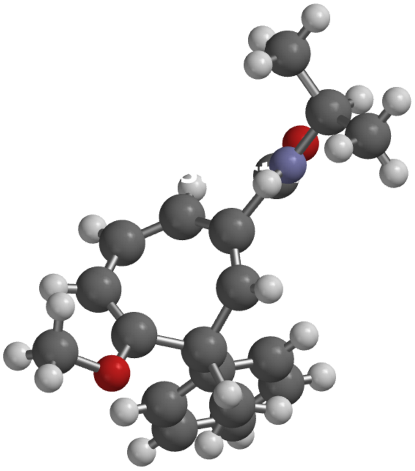

Colorless crystals of **11d** were grown from a hexanes/Aectone solution by slow volatilization. CCDC: 2083305

Bond precision:	C-C = 0.0022 A		Wavelength=1.54178
Cell:	a=9.4746(3)	b=12.0701(4)	c=30.0751(11)
	alpha=90	beta=90	gamma=90
Temperature:	100 K		
	Calculated		Reported
Volume	3439.4(2)		3439.4(2)
Space group	P 21 21 21		P 21 21 21
Hall group	P 2ac 2ab		P 2ac 2ab
Moiety formula	C19 H23 N O2		C19 H23 N O2
Sum formula	C19 H23 N O2		C19 H23 N O2
Mr	297.38		297.38
Dx,g cm-3	1.149		1.149
Z	8		8
Mu (mm-1)	0.582		0.582
F000	1280.0		1280.0
F000’	1283.61		
h,k,lmax	11,14,36		11,14,36
Nref	6342[3593]		6333
Tmin,Tmax	0.876,0.933		0.434,0.753
Tmin’	0.711		

Correction method= # Reported T Limits: Tmin=0.434 Tmax=0.753

AbsCorr = MULTI-SCAN

Data completeness=1.76/1.00 Theta(max)= 68.552

R(reflections)= 0.0266(6290) wR2(reflections)= 0.0674(6333)

S = 1.051 Npar= 452

1.16 Analytical data of enantioselective photochemical ring expansion

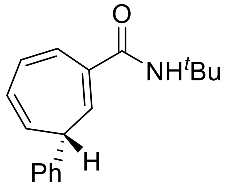

***(R)*-*N*-(tert-butyl)-3-phenylcyclohepta-1,4,6-triene-1-carboxamide (4a):** **General Procedure 6:** To a suspension of chiral amine (2.0 equiv) in dry THF (0.1 M) at –78 °C was added *n*-butyl lithium (3.0 equiv, 2.5 M in hexanes) dropwise. The resulting mixture was allowed to warm to room temperature over 15 minutes, resulting in a clear yellow solution. This was then cooled down to -78 °C and a solution of amide **2a** (100 mg, 0.37 mmol) in THF (0.5 mL) was added dropwise and the reaction mixture was stirred at 700 rpm and irradiated with a 40 W Kessil Tuna Blue lamp. The mixture was stirred at –78 °C for 30 minutes, then the temperature of the Cryostat was set to -10 °C and the reaction mixture was slowly allowed to warm. After a further 1 h, DMPU (3.0 equiv.) was added and further irradiation was continued for 2.5 h. The reaction was quenched with sat. aq. NH_4Cl (15 mL), and the aqueous layer was extracted with EtOAc (3 × 10 mL). The combined organic fractions were washed with brine, dried (Na_2SO_4) and evaporated to yield a crude product, which was purified by flash chromatography, eluting with 10:90 EtOAc:Petrol (Rf = 0.4), afforded the title compound (71 mg, 71%) as a colourless oil. R_f = 0.40; $[\alpha]_D{}^{23}$ = 16 (c = 1.00 in $CHCl_3$); ν_{max} /cm^{-1}(neat): 3322, 2970, 1642, 1452, 1392, 1218, 1075, 753, 698; **^{1}H NMR** (400 MHz, Chloroform-*d*) δ 7.38 – 7.36 (m, 4H), 7.31 – 7.28 (m, 1H), 7.07 (d, *J* = 11.2 Hz, 1H), 6.83 (dd, *J* = 11.2, 5.6 Hz, 1H), 6.26 (ddd, *J* = 9.2, 5.6, 1.5 Hz, 1H), 5.93 (d, *J* = 6.1 Hz, 1H), 5.55 (s, 1H), 5.49 (ddq, *J* = 9.2, 5.4, 0.9 Hz, 1H), 2.72 (td, *J* = 5.9, 1.5 Hz, 1H), 1.37 (s, 9H). **^{13}C NMR** (101 MHz, Chloroform-*d*) δ 167.10, 142.91, 133.64, 132.32, 128.93, 128.19, 127.70, 127.06, 126.47, 125.32, 51.61, 44.65, 28.86. **HRMS** (ESI+): m/z calcd for $C_{18}H_{21}NONa$ [M+Na]+ 290.1515, found 290.1517. **HPLC**: *er* 3.798: 96.202; Chiral Regis Whelk O1,

Hexane:IPA = 85:15 flow = 1.0 mL/min, λ = 280 nm, tR = 20.10 mins (minor), and 25.99 mins (major).

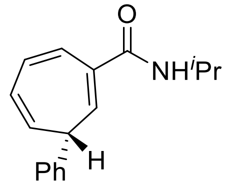

***(R)-N*-isopropyl-3-phenylcyclohepta-1,4,6-triene-1-carboxamide (4a'):**

General Procedure 6: To a suspension of chiral amine (2.0 equiv) in dry THF (0.1 M) at –78 °C, was added *n*-butyl lithium (3.0 equiv, 2.5 M in hexanes), dropwise. The resulting mixture was allowed to warm to room temperature over 15 minutes, resulting in a clear yellow solution. This was then cooled down to -78 °C and a solution of amide **2a'** (100 mg, 0.39 mmol) in THF (0.5 mL) was added dropwise and the reaction mixture was stirred at 700 rpm and irradiated with a 40 W Kessil Tuna Blue lamp. The mixture was stirred at –78 °C for 30 minutes before the temperature of the Cryostat was set to -10 ºC and the reaction mixture was slowly allowed to warm. After a further 1 h, DMPU (3.0 equiv.) was added and further irradiation was continued for 2.5 h. The reaction was quenched with sat. aq. NH_4Cl (15 mL), and the aqueous layer was extracted with EtOAc (3 × 10 mL). The combined organic fractions were washed with brine, dried (Na_2SO_4) and evaporated to yield a crude product, which was purified by flash chromatography, eluting with 85:15 petrol-EtOAc (Rf = 0.40), afforded the title compound (72 mg, 72%) as a pale-yellow oil. R_f = 0.40; $[\alpha]_D^{23}$ = 8 (c = 1.00 in $CHCl_3$); ν_{max} /cm^{-1}(neat): 3295, 2976, 1632, 1531, 1447, 697; **^{1}H NMR** (400 MHz, Chloroform-*d*) δ 7.31 – 7.26 (m, 4H), 7.22 – 7.19 (m, 1H), 7.00 (d, *J* = 11.2 Hz, 1H), 6.74 (dd, *J* = 11.2, 5.6 Hz, 1H), 6.16 (ddd, *J* = 9.2, 5.6, 1.5 Hz, 1H), 5.89 (d, *J* = 6.0 Hz, 1H), 5.58 (d, *J* = 6.5 Hz, 1H), 5.41 – 5.33 (m, 1H), 4.10 – 3.94 (m, 1H), 2.64 (td, *J* = 6.0, 1.4 Hz, 1H), 1.07 (d, *J* = 1.4 Hz, 3H), 1.05 (d, *J* = 1.3 Hz, 3H). **^{13}C NMR** (101 MHz, Chloroform-*d*) δ 166.73, 142.78, 132.66, 132.30, 128.85, 128.43, 128.03, 127.61, 127.52, 126.98, 126.23, 125.28, 44.61, 41.83, 22.77, 22.75. **HRMS** (ESI+): m/z calcd for $C_{17}H_{20}NO$ [M+H]+ 254.1539, found 254.1547; **HPLC**: *er* 42:58; CHIRALPAK® IA column, Hexane: PA = 98:02 flow = 1.0 mL/min, λ = 280 nm, tR = 18.22 mins (minor) and 24.32 mins (major) respectively.

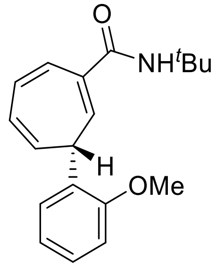

***(R)-N*-(tert-butyl)-3-(2-methoxyphenyl)cyclohepta-1,4,6-triene-1-carboxamide (4b): General Procedure 6:** To a suspension of chiral amine (2.0 equiv) in dry THF (0.1) at –78 °C (Cryostat), was added n-butyl lithium (3.0 equiv, 2.5 M in hexanes), dropwise. The resulting mixture was allowed to warm to room temperature over 15 minutes, resulting in a clear yellow solution. This was then cooled down to -78 °C, a solution of amide **2b** (100 mg, 0.33 mmol) in THF (0.5 mL) was added dropwise and the reaction mixture was stirred at 700 rpm and irradiated with a 40 W Kessil lamp. The mixture was stirred at –78 °C for 30 minutes, then the temperature of the Cryostat was set to -10 °C and the reaction mixture was slowly allowed to warm. After a further 1 h, DMPU (3.0 equiv.) was added and further irradiation was continued for 6.5 h. The reaction was quenched with sat. aq. NH_4Cl (15 mL), and the aqueous layer was extracted with EtOAc (3 × 10 mL). The combined organic fractions were washed with brine, dried (Na_2SO_4) and evaporated to yield a crude product, which was purified by flash chromatography, eluting with 85:15 petrol-EtOAc (Rf = 0.35), afforded the title compound (63 mg, 63%) as a colorless needles (**MP**: 120-122 °C). R_f = 0.38; $[\alpha]_D{}^{23}$ = 16 (c = 1.00 in $CHCl_3$); ν_{max} /cm^{-1}(neat): 3327, 2967, 1644, 1494, 1454, 1242, 1027, 751, 716; **^{1}H NMR** (400 MHz, Chloroform-*d*) δ 7.31 – 7.27 (m, 2H), 7.03 (d, *J* = 11.2 Hz, 1H), 6.97 (td, *J* = 7.5, 1.1 Hz, 1H), 6.94 – 6.90 (m, 1H), 6.82 – 6.75 (m, 1H), 6.21 (ddd, *J* = 9.2, 5.6, 1.5 Hz, 1H), 5.90 (d, *J* = 6.0 Hz, 1H), 5.56 (s, 1H), 5.49 – 5.41 (m, 1H), 3.79 (s, 3H), 3.07 (td, *J* = 5.8, 1.5 Hz, 1H), 1.36 (s, 9H). **^{13}C NMR** (101 MHz, Chloroform-*d*) δ 167.28, 157.43, 133.36, 132.08, 130.71, 129.18, 128.61, 128.27, 128.04, 127.93, 127.34, 126.80, 124.89, 120.89, 111.01, 55.45, 51.50, 40.01, 28.87. **HRMS** (ESI+): m/z calcd for $C_{19}H_{23}NO_2Na$ [M+Na]+ 320.1621, found 320.1622; **HPLC**: er 8:92; CHIRALPAK® IA column, Hexane:IPA = 97:3 flow = 1.0 mL/min, λ = 280 nm, tR = 8.9 mins (minor) and 10.11 mins (major).

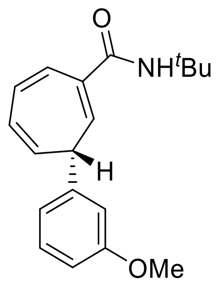

***(R)-N*-(tert-butyl)-3-(3-methoxyphenyl)cyclohepta-1,4,6-triene-1-carboxamide (4c): General Procedure 6:** To a suspension of chiral amine (2.0 equiv) in dry THF (0.1) at –78 °C (Cryostat), was added n-butyl lithium (3.0 equiv, 2.5 M in hexanes), dropwise. The resulting mixture was allowed to warm to room temperature over 15 minutes, resulting in a clear yellow solution. This was then cooled down to -78 °C, a solution of amide **2c** (100 mg, 0.33 mmol) in THF (0.5 mL) was added dropwise and the reaction mixture was stirred at 700 rpm and irradiated with a 40 W Kessil lamp. The mixture was stirred at –78 °C for 30 minutes, then the temperature of the Cryostat was set to -10 °C and the reaction mixture was slowly allowed to warm. After a further 1 h, DMPU (3.0 equiv.) was added and further irradiation was continued for 22.5 h. The reaction was quenched with sat. aq. NH_4Cl (15 mL), and the aqueous layer was extracted with EtOAc (3 × 10 mL). The combined organic fractions were washed with brine, dried (Na_2SO_4) and evaporated to yield a crude product, which was purified by flash chromatography, eluting with 85:15 petrol-EtOAc (Rf = 0.35), afforded the title compound (56 mg, 56%) as a colorless needles (**MP**: 107-109 °C). R_f = 0.40; $[\alpha]_D^{24}$ = 20 (c = 1.00 in $CHCl_3$); ν_{max} /cm^{-1}(neat): 3327, 2968, 1642, 1453, 1391, 1220, 1156, 1049, 752, 716, 699; **^{1}H NMR** (400 MHz, Chloroform-*d*) δ 7.32 – 7.27 (m, 1H), 7.08 (d, *J* = 11.2 Hz, 1H), 6.98 – 6.93 (m, 1H), 6.91 – 6.89 (m, 1H), 6.87 – 6.81 (m, 2H), 6.26 (ddd, *J* = 9.2, 5.6, 1.5 Hz, 1H), 5.93 (d, *J* = 5.3 Hz, 1H), 5.58 (s, 1H), 5.53 – 5.47 (m, 1H), 2.71 (td, *J* = 5.9, 1.4 Hz, 1H), 1.38 (s, 9H). **^{13}C NMR** (101 MHz, Chloroform-*d*) δ 167.11, 160.06, 144.56, 133.68, 132.30, 129.95, 128.24, 127.25, 126.35, 125.31, 119.99, 113.55, 112.33, 55.35, 51.60, 44.72, 28.86. **HRMS** (ESI+): m/z calcd for $C_{19}H_{23}NO_2Na$ [M+Na]+ 320.1621, found 320.1626; **HPLC**: er 9:91; CHIRALPAK® IA column, Hexane:IPA = 99:1 flow = 1.0 mL/min, λ = 280 nm, tR = 14.11 mins (minor) and 19.42 mins (major).

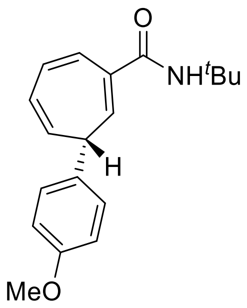

***(R)*-*N*-(tert-butyl)-3-(4-methoxyphenyl)cyclohepta-1,4,6-triene-1-carboxamide (4d):**

To a suspension of chiral amine (2.0 equiv) in dry THF (0.1) at –78 °C (Cryostat), was added n-butyl lithium (3.0 equiv, 2.5 M in hexanes), dropwise. The resulting mixture was allowed to warm to room temperature over 15 minutes, resulting in a clear yellow solution. This was then cooled down to -78 °C, a solution of amide **2d** (100 mg, 0.33 mmol) in THF (0.5 mL) and 3.0 equiv DMPU were sequentially added dropwise. The mixture was stirred at –78 °C for 30 minutes before being slowly warmed to room temperature over 5 hours. After 5h reaction mixture was re-cool to -10 °C and placed under kessil LED. Irradiation was continued at -10 °C for 10 h. The reaction was quenched with sat. aq. NH_4Cl (15 mL), and the aqueous layer was extracted with EtOAc (3 × 10 mL). The combined organic fractions were washed with brine, dried (Na_2SO_4) and evaporated to yield a crude product, which was purified by flash chromatography, eluting with 85:15 petrol-EtOAc, afforded the title compound (30 mg, 30%) as a colorless needles (**MP**: 99-101 °C). R_f = 0.40; $[\alpha]_D^{23.2}$ = 8 (c = 1.00 in $CHCl_3$); ν_{max} /cm^{-1}(neat): 3295, 2971, 1633, 1512, 1392, 1247; **^{1}H NMR** (400 MHz, Chloroform-*d*) δ 7.29 – 7.27 (m, 2H), 7.07 (d, *J* = 11.2 Hz, 1H), 6.94 – 6.91 (m, 2H), 6.84 (dd, *J* = 11.4, 5.5 Hz, 1H), 6.25 (ddd, *J* = 9.2, 5.6, 1.5 Hz, 1H), 5.93 (d, *J* = 6.0 Hz, 1H), 5.54 (s, 1H), 5.52 – 5.45 (m, 1H), 3.83 (s, 3H), 2.71 – 2.66 (m, 1H), 1.38 (s, 9H). **^{13}C NMR** (101 MHz, Chloroform-*d*) δ 167.06, 158.59, 132.27, 128.82, 128.57, 128.11, 127.42, 127.16, 125.05, 114.21, 55.35, 51.51, 43.86, 28.79. **HRMS** (ESI+): m/z calcd for $C_{19}H_{23}NO_2Na$ [M+Na]+ 320.1621, found 320.1621; **HPLC**: er 23:77; Chiral Regis Whelk O1, Hexane:IPA = 80:20 flow = 1.0 mL/min, λ = 280 nm, tR = 31.285 mins(minor) and 40:568 mins (major).

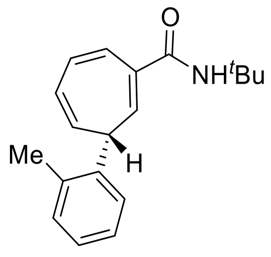

***(R)-N*-(tert-butyl)-3-(o-tolyl)cyclohepta-1,4,6-triene-1-carboxamide (4e): General Procedure 6:** To a suspension of chiral amine (2.0 equiv) in dry THF (0.1) at –78 °C (Cryostat), was added n-butyl lithium (3.0 equiv, 2.5 M in hexanes), dropwise. The resulting mixture was allowed to warm to room temperature over 15 minutes, resulting in a clear yellow solution. This was then cooled down to -78 °C, a solution of amide **2e** (100 mg, 0.35 mmol) in THF (0.5 mL) was added dropwise and the reaction mixture was stirred at 700 rpm and irradiated with a 40 W Kessil lamp. The mixture was stirred at –78 °C for 30 minutes, then the temperature of the Cryostat was set to -10 °C and the reaction mixture was slowly allowed to warm. After a further 1 h, DMPU (3.0 equiv.) was added and further irradiation was continued for 3.5 h. The reaction was quenched with sat. aq. NH_4Cl (15 mL), and the aqueous layer was extracted with EtOAc (3 × 10 mL). The combined organic fractions were washed with brine, dried (Na_2SO_4) and evaporated to yield a crude product, which was purified by flash chromatography, eluting with 90:10 petrol-EtOAc (Rf = 0.35), afforded the title compound (85 mg, 85%) as a pale-yellow oil. R_f = 0.40; $[\alpha]_D^{23.2}$ = 20 (c = 1.00 in $CHCl_3$); ν_{max} /cm^{-1}(neat): 3322, 2970, 1640, 1525, 1452, 1392, 1219, 1065, 752; **^{1}H NMR** (400 MHz, Chloroform-*d*) δ 7.47 (d, *J* = 7.4 Hz, 1H), 7.31 – 7.25 (m, 1H), 7.20 – 7.17 (m, 2H), 7.06 (d, *J* = 11.1 Hz, 1H), 6.81 (dd, *J* = 11.3, 5.5 Hz, 1H), 6.27 (ddd, *J* = 9.1, 5.7, 1.5 Hz, 1H), 5.86 – 5.78 (m, 1H), 5.54 (s, 1H), 5.42 – 5.32 (m, 1H), 2.90 (td, *J* = 5.8, 1.5 Hz, 1H), 2.19 (s, 3H), 1.37 (s, 9H). **^{13}C NMR** (101 MHz, Chloroform-*d*) δ 167.13, 140.90, 136.34, 133.88, 131.95, 130.70, 128.05, 126.86, 126.79, 126.61, 125.61, 125.36, 124.57, 51.59, 40.57, 28.86, 19.64; **HRMS** (ESI+): m/z calcd for $C_{19}H_{23}NONa$ [M+Na]+ 304.1675, found 304.1672; **HPLC**: *er* 4:96; Chiral Regis Whelk O1, Hexane:IPA = 90:10, flow = 1.0 mL/min, λ = 280 nm, tR = 30.722 mins(minor) and 44.404 mins (major).

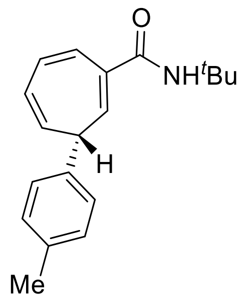

***(R)*-*N*-(tert-butyl)-3-(*p*-tolyl)cyclohepta-1,4,6-triene-1-carboxamide (4f): General Procedure 6:** To a suspension of chiral amine (2.0 equiv) in dry THF (0.1) at –78 °C (Cryostat), was added n-butyl lithium (3.0 equiv, 2.5 M in hexanes), dropwise. The resulting mixture was allowed to warm to room temperature over 15 minutes, resulting in a clear yellow solution. This was then cooled down to -78 °C, a solution of amide **2f** (100 mg, 0.35 mmol) in THF (0.5 mL) was added dropwise and the reaction mixture was stirred at 700 rpm and irradiated with a 40 W Kessil lamp. The mixture was stirred at –78 °C for 30 minutes, then the temperature of the Cryostat was set to -10 °C and the reaction mixture was slowly allowed to warm. After a further 1 h, DMPU (3.0 equiv.) was added and further irradiation was continued for 8.5 h. The reaction was quenched with sat. aq. NH_4Cl (15 mL), and the aqueous layer was extracted with EtOAc (3 × 10 mL). The combined organic fractions were washed with brine, dried (Na_2SO_4) and evaporated to yield a crude product, which was purified by flash chromatography, eluting with 90:10 petrol-EtOAc (Rf = 0.35), afforded the title compound (40 mg, 40%) as a pale-yellow oil. R_f = 0.40; $[\alpha]_D^{23.2}$ = 12 (c = 1.00 in $CHCl_3$); ν_{max} /cm^{-1}(neat): 3327, 2959, 1644, 1512, 1451, 1363, 1217, 750, 697; **^{1}H NMR** (500 MHz, Chloroform-*d*) δ 7.30 – 7.26 (m, 2H), 7.23 (t, *J* = 6.2 Hz, 2H), 7.11 (d, *J* = 11.2 Hz, 1H), 6.87 (dd, *J* = 11.2, 5.6 Hz, 1H), 6.31 – 6.25 (m, 1H), 5.94 (d, *J* = 6.0 Hz, 1H), 5.57 (s, 1H), 5.52 (dd, *J* = 9.1, 5.4 Hz, 1H), 2.73 (t, *J* = 5.5 Hz, 1H), 2.39 (s, 3H), 1.40 (s, 9H). **^{13}C NMR** (126 MHz Chloroform-*d*) δ 167.09, 139.84, 136.58, 133.51, 132.24, 129.51, 128.16, 127.75, 126.86, 125.13, 51.51, 44.24, 28.78, 21.07. **HRMS** (ESI+): m/z calcd for $C_{19}H_{23}NONa$ [M+Na]+ 304.1675, found 304.1671; **HPLC**: *er* 4:96; CHIRALPAK® IA column, Hexane:IPA = 99:01, flow = 1.0 mL/min, λ = 280 nm, tR = 10.271 mins(minor) and 12.86 mins (major).

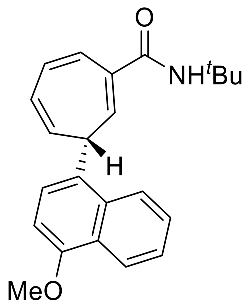

***(R)-N*-(tert-butyl)-3-(4-methoxynaphthalen-1-yl)cyclohepta-1,4,6-triene-1-carboxamide (4g): General Procedure 6:** To a suspension of chiral amine (2.0 equiv) in dry THF (0.1) at –78 ºC (Cryostat), was added n-butyl lithium (3.0 equiv, 2.5 M in hexanes), dropwise. The resulting mixture was allowed to warm to room temperature over 15 minutes, resulting in a clear yellow solution. This was then cooled down to -78 °C, a solution of amide **2g** (100 mg, 0.28 mmol) in THF (0.5 mL) was added dropwise and the reaction mixture was stirred at 700 rpm and irradiated with a 40 W Kessil lamp. The mixture was stirred at –78 ºC for 30 minutes, then the temperature of the Cryostat was set to -10 ºC and the reaction mixture was slowly allowed to warm. After a further 1 h, DMPU (3.0 equiv.) was added and further irradiation was continued for 14.5 h. The reaction was quenched with sat. aq. NH_4Cl (15 mL), and the aqueous layer was extracted with EtOAc (3 × 10 mL). The combined organic fractions were washed with brine, dried (Na_2SO_4) and evaporated to yield a crude product, which was purified by flash chromatography, eluting with 80:20 petrol-EtOAc (Rf = 0.35), afforded the title compound (40 mg, 40%) as a yellow oil. R_f = 0.40; $[\alpha]_D^{22.6}$ = 12 (c = 1.00 in $CHCl_3$); ν_{max} /cm^{-1}(neat): 3327, 2970, 1649, 1586, 1518, 1392, 1225, 1055, 759; **^{1}H NMR** (400 MHz, Chloroform-*d*) δ 8.37 – 8.31 (m, 1H), 7.89 – 7.81 (m, 1H), 7.52 – 7.45 (m, 3H), 7.11 (d, *J* = 11.0 Hz, 1H), 6.88 – 6.83 (m, 1H), 6.82 (d, *J* = 8.1 Hz, 1H), 6.30 (ddd, *J* = 9.0, 5.7, 1.4 Hz, 1H), 5.95 (d, *J* = 6.0 Hz, 1H), 5.60 – 5.47 (m, 2H), 4.01 (s, 3H), 3.28 (t, *J* = 5.7 Hz, 1H), 1.36 (s, 9H). **^{13}C NMR** (101 MHz, Chloroform-*d*) δ 167.19, 154.98, 133.63, 132.39, 131.90, 129.84, 128.20, 126.62, 126.40, 125.42, 125.18, 124.72, 124.02, 123.58, 122.97, 103.29, 55.64, 51.59, 40.63, 28.86. **HRMS** (ESI+): m/z calcd for $C_{23}H_{25}NO_2Na$ [M+Na]+ 370.1777, found 370.1788; **HPLC**: er 6:94 Chiral Regis Whelk O1, Hexane:IPA = 70:30 flow = 1.0 mL/min, λ = 280 nm, tR = 33.68 (minor), and 41.3 (major).

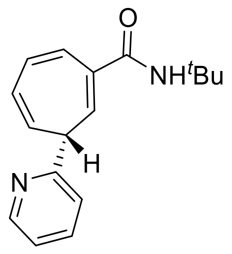

***(R)-N*-(tert-butyl)-3-(pyridin-2-yl)cyclohepta-1,4,6-triene-1-carboxamide (4h): General Procedure 6:** To a suspension of chiral *(R)*-amine (2.0 equiv) in dry THF (0.1) at –78 °C (Cryostat), was added n-butyl lithium (3.0 equiv, 2.5 M in hexanes), dropwise. The resulting mixture was allowed to warm to room temperature over 15 minutes, resulting in a clear yellow solution. This was then cooled down to -78 °C, a solution of amide **2h** (100 mg, 0.28 mmol) in THF (0.5 mL) was added dropwise and the reaction mixture was stirred at 700 rpm and irradiated with a 40 W Kessil lamp. The mixture was stirred at –78 °C for 30 minutes, then the temperature of the Cryostat was set to -10 °C and the reaction mixture was slowly allowed to warm. After a further 1 h, DMPU (3.0 equiv.) was added and further irradiation was continued for 5.5 h. The reaction was quenched with sat. aq. NH_4Cl (15 mL), and the aqueous layer was extracted with EtOAc (3 × 10 mL). The combined organic fractions were washed with brine, dried (Na_2SO_4) and evaporated to yield a crude product, which was purified by flash chromatography, eluting with 80:20 petrol-EtOAc (Rf = 0.35), afforded the title compound (60 mg, 60%) as a yellow oil. R_f = 0.35 $[\alpha]_D^{23.2}$ = 12 (c = 1.00 in $CHCl_3$); ν_{max} /cm^{-1}(neat): 3313, 2970, 1650, 1543, 1363, 1223, 1052, 749; **^{1}H NMR** (400 MHz, Chloroform-*d*) δ 8.59 (ddd, *J* = 4.8, 1.8, 0.9 Hz, 1H), 7.65 (ddd, *J* = 7.5, 1.9 Hz, 1H), 7.51 (dt, *J* = 8.0, 1.0 Hz, 1H), 7.25 – 7.21 (m, 1H), 7.16 (ddd, *J* = 7.5, 4.8, 1.1 Hz, 1H), 6.73 (dd, *J* = 10.4, 6.3 Hz, 1H), 6.32 (dd, *J* = 8.4, 6.3 Hz, 1H), 5.59 – 5.51 (m, 1H), 5.48 (s, 1H), 5.01 (ddt, *J* = 7.8, 5.3, 2.5 Hz, 1H), 2.11 – 2.06 (m, 1H), 1.39 (s, 9H). **^{13}C NMR** (101 MHz, Chloroform-*d*) δ 171.86, 157.47, 149.29, 136.84, 136.57, 130.12, 128.80, 126.57, 122.00, 121.08, 103.02, 101.69, 51.62, 41.91, 28.96. **HRMS** (ESI+): m/z calcd for $C_{17}H_{20}N_2ONa$ [M+Na]+ 291.1467, found 291.1472; **HPLC**: er 39:61 Chiral Regis Whelk O1, Hexane:IPA = 85:15 flow = 1.0 mL/min, λ = 280 nm, tR = 20.987 mins(minor) and 22.505 mins (major).

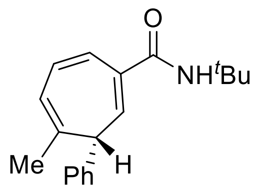

***(S)-N*-(tert-butyl)-4-methyl-3-phenylcyclohepta-1,4,6-triene-1-carboxamide (4i): General Procedure 6:** To a suspension of chiral amine (2.0 equiv) in dry THF (0.1) at –78 ºC (Cryostat), was added n-butyl lithium (3.0 equiv, 2.5 M in hexanes), dropwise. The resulting mixture was allowed to warm to room temperature over 15 minutes, resulting in a clear yellow solution. This was then cooling down to -78 °C, a solution of amide **4i** (100 mg, 0.35 mmol) in THF (0.5 mL) was added dropwise and the reaction mixture was stirred at 700 rpm and irradiated with a 40 W Kessil lamp. The mixture was stirred at –78 ºC for 30 minutes, then the temperature of the Cryostat was set to -10 ºC and the reaction mixture was slowly allowed to warm. After a further 1 h, DMPU (3.0 equiv.) was added and further irradiation was continued for 4.5 h. The reaction was quenched with sat. aq. NH_4Cl (15 mL), and the aqueous layer was extracted with EtOAc (3 × 10 mL). The combined organic fractions were washed with brine, dried (Na_2SO_4) and evaporated to yield a crude product, which was purified by flash chromatography, eluting with 90:10 petrol-EtOAc (Rf = 0.40), afforded the title compound (68 mg, 68%) as a colorless oil. R_f = 0.40; $[\alpha]_D{}^{23}$ = 20 (c = 1.00 in $CHCl_3$); ν_{max} /cm^{-1}(neat): 3354, 2971, 1644, 1532, 1451, 1393, 1055; **^{1}H NMR** (400 MHz, Chloroform-*d*) δ 7.27 – 7.18 (m, 3H), 7.17 – 7.14 (m, 2H), 6.70 (d, *J* = 11.2 Hz, 1H), 6.55 (dd, *J* = 11.2, 5.7 Hz, 1H), 6.27 (d, *J* = 7.4 Hz, 1H), 6.00 (d, *J* = 6.6 Hz, 1H), 5.52 (s, 1H), 3.04 (d, *J* = 7.4 Hz, 1H), 1.61 (s, 3H), 1.28 (s, 9H). **^{13}C NMR** (101 MHz, Chloroform-*d*) δ 167.02, 139.94, 137.31, 133.88, 132.12, 128.67, 128.28, 127.39, 126.77, 125.56, 122.28, 51.48, 48.29, 28.81, 22.11. **HRMS** (ESI+): m/z calcd for $C_{19}H_{23}NONa$ [M+Na]+ 304.1672, found 304.1674; **HPLC**: *er* 5.3: 94:6; Chiral Regis Whelk O1, Hexane:IPA = 70:30 flow = 1.0 mL/min, λ = 280 nm, tR = 21.53 mins (minor) and 29.20 mins (major) respectively.

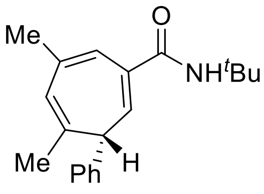

***(S)-N*-(tert-butyl)-4,6-dimethyl-3-phenylcyclohepta-1,4,6-triene-1-carboxamide (4j): General Procedure 6:** To a suspension of chiral amine (2.0 equiv) in dry THF (0.1) at –78 ºC (Cryostat), was added n-butyl lithium (3.0 equiv, 2.5 M in hexanes), dropwise. The resulting mixture was allowed to warm to room temperature over 15 minutes, resulting in a clear

yellow solution. This was then cooled down to -78 °C, a solution of amide **2j** (100 mg, 0.33 mmol) in THF (0.5 mL) was added dropwise and the reaction mixture was stirred at 700 rpm and irradiated with a 40 W Kessil lamp. The mixture was stirred at –78 °C for 30 minutes, then the temperature of the Cryostat was set to -5 °C and the reaction mixture was slowly allowed to warm. After a further 1 h, DMPU (3.0 equiv.) was added and further irradiation was continued for 8.5 h. The reaction was quenched with sat. aq. NH_4Cl (15 mL), and the aqueous layer was extracted with EtOAc (3 × 10 mL). The combined organic fractions were washed with brine, dried (Na_2SO_4) and evaporated to yield a crude product, which was purified by flash chromatography, eluting with 90:10 petrol-EtOAc (Rf = 0.40), afforded the title compound (50 mg, 50%) as a colorless oil. R_f = 0.39; $[\alpha]_D^{22.8}$ = 16 (c = 1.00 in $CHCl_3$); ν_{max} /cm^{-1}(neat): 3320, 2971, 1662, 1529, 1455, 1398, 1065; **^{1}H NMR** (400 MHz, Chloroform-*d*) δ 7.29 – 7.17 (m, 5H), 6.53 (s, 1H), 6.25 (d, *J* = 7.2 Hz, 1H), 5.87 (s, 1H), 5.55 (s, 1H), 3.13 (d, *J* = 7.2 Hz, 1H), 2.01 (s, 3H), 1.61 (s, 3H), 1.32 (s, 9H). **^{13}C NMR** (101 MHz, $CDCl_3$) δ 167.46, 141.46, 140.22, 136.06, 134.16, 128.66, 128.26, 126.69, 126.09, 125.40, 122.98, 51.41, 48.19, 28.82, 24.69, 21.95. **HRMS** (ESI+): m/z calcd for $C_{20}H_{25}NONa$ $[M+Na]^+$ 318.1834, found 318.1831; **HPLC**: *er* 29:71; Chiral Regis Whelk O1, Hexane:IPA = 70:30 flow = 1.0 mL/min, λ = 280 nm, tR = 10.41 mins (minor) and 11.99 mins (major).

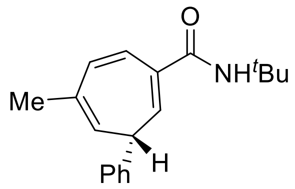

***(R)*-*N*-(tert-butyl)-5-methyl-3-phenylcyclohepta-1,4,6-triene-1-carboxamide (4k):**

Condition 1: General Procedure 6: To a suspension of chiral amine (2.0 equiv) in dry THF (0.1) at –78 °C (Cryostat), was added *n*-butyl lithium (3.0 equiv, 2.5 M in hexanes), dropwise. The resulting mixture was allowed to warm to room temperature over 15 minutes, resulting in a clear yellow solution. This was then cooled down to -78 °C, a solution of amide **2k** (100 mg, 0.35 mmol) in THF (0.5 mL) was added dropwise and the reaction mixture was stirred at 700 rpm and irradiated with a 40 W Kessil lamp. The mixture was stirred at –78 °C for 30 minutes, then the temperature of the Cryostat was set to -10 °C and the reaction mixture was slowly allowed to warm. After a further 1 h, DMPU (3.0 equiv.) was added and further irradiation was continued for 8.5 h. The reaction was quenched with sat. aq. NH_4Cl (15 mL), and the aqueous layer was extracted with

EtOAc (3 × 10 mL). The combined organic fractions were washed with brine, dried (Na_2SO_4) and evaporated to yield a crude product, which was purified by flash chromatography, eluting with 9:1 petrol-EtOAc (Rf = 0.40), afforded the title compound (84 mg, 84%) as a colorless oil. **HPLC**: Chiral Regis Whelk O1, eluting with hexane-IPA (70:30), showed it to consist of a 20:80 mixture of two enantiomers with retention times 9.88 mins (minor) and 16.17 min (major) respectively.

Condition 2: General Procedure 6: To a suspension of chiral amine (2.0 equiv) in dry THF (0.1) at –78 °C (Cryostat), was added n-butyl lithium (3.0 equiv, 2.5 M in hexanes), dropwise. The resulting mixture was allowed to warm to room temperature over 15 minutes, resulting in a clear yellow solution. This was then cooled down to -78 °C, a solution of amide **2k** (100 mg, 0.35 mmol) in THF (0.5 mL) was added dropwise and the reaction mixture was stirred at 700 rpm and irradiated with a 40 W Kessil lamp. The mixture was stirred at –78 °C for 30 minutes, then the temperature of the Cryostat was set to -20 °C and the reaction mixture was slowly allowed to warm. After a further 1 h, DMPU (3.0 equiv.) was added and further irradiation was continued for 14.5 h. The reaction was quenched with sat. aq. NH_4Cl (15 mL), and the aqueous layer was extracted with EtOAc (3 × 10 mL). The combined organic fractions were washed with brine, dried (Na_2SO_4) and evaporated to yield a crude product, which was purified by flash chromatography, eluting with 90:10 petrol-EtOAc (Rf = 0.40), afforded the title compound (86 mg, 86%) as a colorless needles (**MP**: 97-99 °C). R_f = 0.40; $[\alpha]_D^{22.5}$ = 48 (c = 1.00 in $CHCl_3$); ν_{max} /cm^{-1}(neat): 3327, 2967, 1641, 1529, 1451, 1218, 753, 699; **^{1}H NMR** (400 MHz, Chloroform-*d*) δ 7.38 – 7.32 (m, 2H), 7.29 (dt, *J* = 7.7, 1.7 Hz, 3H), 7.27 – 7.23 (m, 1H), 6.90 (d, *J* = 10.7 Hz, 1H), 6.58 (d, *J* = 10.7 Hz, 1H), 5.58 (s, 1H), 5.52 (dt, *J* = 5.8, 1.3 Hz, 1H), 4.81 – 4.74 (m, 1H), 2.52 (t, *J* = 5.5 Hz, 1H), 1.94 (s, 3H), 1.37 (s, 9H). **^{13}C NMR** (101 MHz, Chloroform-*d*) δ 167.01, 143.20, 134.79, 132.66, 132.48, 128.81, 127.40, 126.76, 114.33, 110.58, 51.51, 41.40, 28.88, 21.56. **HRMS** (ESI+): m/z calcd for $C_{19}H_{23}NONa$ [M+Na]+ 304.1677, found 304.1674; **HPLC**: *er* 12:88; Chiral Regis Whelk O1, Hexane:IPA = 70:30 flow = 1.0 mL/min, λ = 280 nm, tR = 10.41 mins (minor) and 16.29 mins (major).

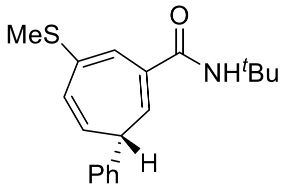

***(R)*-*N*-(tert-butyl)-6-(methylthio)-3-phenylcyclohepta-1,4,6-triene-1-carboxamide (4l): General Procedure 6:** To a suspension of chiral amine (2.0 equiv) in dry THF

(0.1) at –78 °C (Cryostat), was added n-butyl lithium (3.0 equiv, 2.5 M in hexanes), dropwise. The resulting mixture was allowed to warm to room temperature over 15 minutes, resulting in a clear yellow solution. This was then cooled down to -78 °C, a solution of amide **2l** (100 mg, 0.31 mmol) in THF (0.5 mL) was added dropwise and the reaction mixture was stirred at 700 rpm and irradiated with a 40 W Kessil lamp. The mixture was stirred at –78 °C for 30 minutes, then the temperature of the Cryostat was set to -10 °C and the reaction mixture was slowly allowed to warm. After a further 1 h, DMPU (3.0 equiv.) was added and further irradiation was continued for 4.5 h. The reaction was quenched with sat. aq. NH_4Cl (15 mL), and the aqueous layer was extracted with EtOAc (3 × 10 mL). The combined organic fractions were washed with brine, dried (Na_2SO_4) and evaporated to yield a crude product, which was purified by flash chromatography, eluting with 90:10 petrol-EtOAc (Rf = 0.35), afforded the title compound (30 mg, 30%) as a yellow needle (**MP**: 110-112 °C). R_f = 0.42; $[\alpha]_D^{23.2}$ = 16 (c = 1.00 in $CHCl_3$); ν_{max} /cm^{-1}(neat): 3322, 2962, 1638, 1523, 752, 700; **^{1}H NMR** (400 MHz, Chloroform-*d*) δ 7.42 – 7.34 (m, 4H), 7.33 – 7.28 (m, 1H), 6.92 (s, 1H), 6.19 (dt, *J* = 9.5, 1.4 Hz, 1H), 5.90 (d, *J* = 5.8 Hz, 1H), 5.61 (ddt, *J* = 9.6, 5.9, 0.7 Hz, 1H), 5.54 (s, 1H), 2.98 (td, *J* = 5.8, 1.4 Hz, 1H), 2.43 (s, 3H), 1.38 (s, 9H). **^{13}C NMR** (101 MHz, Chloroform-*d*) δ 167.05, 142.95, 142.47, 133.88, 128.97, 128.52, 127.68, 127.53, 127.15, 125.75, 121.77, 100.01, 51.65, 44.84, 28.86, 15.88. **HRMS** (ESI+): m/z calcd for $C_{19}H_{23}NOSNa$ [M+Na]+ 336.1396, found 336.1397; **HPLC**: er 1:99; CHIRALPAK® IA column, Hexane:IPA = 99:05, flow = 1.0 mL/min, λ = 280 nm, tR = 7.68 mins(minor) and 8.982 mins (major).

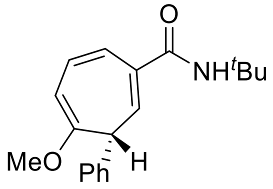

***(R)*-*N*-(tert-butyl)-4-methoxy-3-phenylcyclohepta-1,4,6-triene-1-carboxamide (4m):** To a suspension of chiral amine (2.0 equiv) in dry THF (0.1) at –78 °C (Cryostat), was added n-butyl lithium (3.0 equiv, 2.5 M in hexanes), dropwise. The resulting mixture was allowed to warm to room temperature over 15 minutes, resulting in a clear yellow solution. This was then cooled down to -78 °C, a solution of amide **2m** (100 mg, 0.33 mmol) in THF (0.5 mL) and 3.0 equiv DMPU were sequentially added dropwise. The mixture was stirred at –78 °C for 30 minutes before being slowly warmed to room temperature over 5 hours. After 5h reaction mixture was re-cool to -10 °C and placed under kessil LED. Irradiation was continued at -10 °C for 4 h. The reaction was quenched with sat. aq. NH_4Cl (15 mL), and the aqueous layer

was extracted with EtOAc (3 × 10 mL). The combined organic fractions were washed with brine, dried (Na_2SO_4) and evaporated to yield a crude product, which was purified by flash chromatography, eluting with 85:15 petrol-EtOAc, afforded the title compound (65 mg, 65%) as a colorless needles (**MP**: 123-125 °C); R_f = 0.40; $[\alpha]_D^{22.8}$ = 8 (c = 1.00 in $CHCl_3$); ν_{max} /cm^{-1}(neat): 3322, 2969, 1649, 1532, 1410, 1229, 700; **^{1}H NMR** (400 MHz, Chloroform-*d*) δ 7.23 – 7.12 (m, 6H), 6.48 – 6.43 (m, 2H), 6.17 (d, *J* = 8.1 Hz, 1H), 5.57 (s, 1H), 5.41 (dd, *J* = 4.5, 2.5 Hz, 1H), 3.60 (d, *J* = 8.1 Hz, 1H), 3.56 (s, 3H), 1.32 (s, 9H). **^{13}C NMR** (101 MHz, $CDCl_3$) δ 167.25, 155.14, 137.97, 135.57, 130.06, 128.50, 127.89, 126.75, 125.53, 121.08, 97.05, 56.65, 51.46, 47.81, 28.81. **HRMS** (ESI+): m/z calcd for $C_{19}H_{23}NO_2Na$ [M+Na]+ 320.1621, found 320.1633; **HPLC**: er 39:61; CHIRALPAK® IA column, Hexane:IPA = 90:10 flow = 1.0 mL/min, λ = 280 nm, tR = 6.025 mins (minor) and 7.58 mins (major).

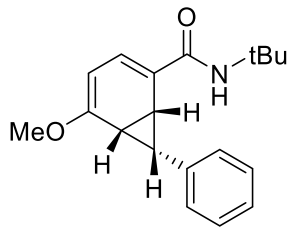

(1*R*,6*S*,7*S*)-*N*-(tert-butyl)-5-methoxy-7-phenylbicyclo[4.1.0]hepta-2,4-diene-2-carboxamide (5n): General Procedure 6: To a suspension of chiral amine (2.0 equiv) in dry THF (0.1) at –78 °C (Cryostat), was added n-butyl lithium (3.0 equiv, 2.5 M in hexanes), dropwise. The resulting mixture was allowed to warm to room temperature over 15 minutes, resulting in a clear yellow solution. This was then cooled down to -78 °C, a solution of amide **2n** (100 mg, 0.33 mmol) in THF (0.5 mL) was added dropwise and the reaction mixture was stirred at 700 rpm and irradiated with a 40 W Kessil lamp. The mixture was stirred at –78 °C for 30 minutes, then the temperature of the Cryostat was set to -10 °C and the reaction mixture was slowly allowed to warm. After a further 1 h, DMPU (3.0 equiv.) was added and further irradiation was continued for 4.5 h. Saturated aq. NH_4Cl was added, the layers separated, and the aqueous layer was extracted with ethyl acetate (10 x 3). The combined organic fractions were washed with brine, dried (Na_2SO_4) and evaporated to yield a crude product, which was purified by flash chromatography, eluting with 85:15 petrol-EtOAc (Rf = 0.35), afforded the title compound (78 mg, 78%) as a yellow oil. R_f = 0.40; $[\alpha]_D^{22}$ = 41 (c = 1.00 in $CHCl_3$); ν_{max} /cm^{-1}(neat): 3327, 2969, 1646, 1553, 1452, 1231, 749, 697; **^{1}H NMR** (400 MHz, Chloroform-*d*) δ 7.20 – 7.11 (m, 2H), 7.11 – 7.03 (m, 1H), 7.02 – 6.94 (m, 2H), 6.61 (dd, *J* = 8.3, 1.1 Hz, 1H), 5.45 (s, 1H), 5.26 (dd, *J* = 8.3,

1.4 Hz, 1H), 3.52 (s, 3H), 3.32 (t, $J = 5.9$ Hz, 1H), 2.71 (ddd, $J = 6.7, 4.8, 1.4$ Hz, 1H), 1.57 (t, $J = 4.9$ Hz, 1H), 1.24 (s, 9H). **^{13}C NMR** (101 MHz, Chloroform-*d*) δ 166.65, 161.35, 141.55, 128.58, 126.73, 126.23, 126.16, 113.59, 98.82, 55.84, 51.27, 28.92, 28.76, 25.79. **HRMS** (ESI+): m/z calcd for $C_{19}H_{23}NO_2Na$ [M+Na]+ 320.1621, found 320.1617; **HPLC**: *er* 6:94 CHIRALPAK® IA column, Hexane:IPA = 95:5 flow = 1.0 mL/min, λ = 280 nm, tR = 18.05 mins (minor), and 23.82 mins (major).

1.17 Analytical data of Product modifications

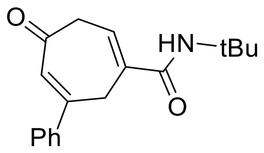

tert-butyl 4-oxo-6-phenylcyclohepta-1,5-diene-1-carboxylate (6n): To a suspension of chiral amine (2.0 equiv) in dry THF (0.1) at –78 °C (Cryostat), was added n-butyl lithium (3.0 equiv, 2.5 M in hexanes), dropwise. The resulting mixture was allowed to warm to room temperature over 15 minutes, resulting in a clear yellow solution. This was then cooling down to -78 °C, a solution of amide **2f** (100 mg, 0.33 mmol) in THF (0.5 mL) was added dropwise and the reaction mixture was stirred at 700 rpm and irradiated with a 40 W Kessil lamp. The mixture was stirred at –78 °C for 30 minutes, then the temperature of the Cryostat was set to -10 °C and the reaction mixture was slowly allowed to warm. After a further 1 h, DMPU (3.0 equiv.) was added and further irradiation was continued for 4.5 h. Saturated aq. NH_4Cl was added, the layers separated, and the aqueous layer was extracted with ethyl acetate (10 x 3). The combined organic fractions were washed with brine, dried (Na_2SO_4) and evaporated to yield a crude product, and conc HCl (1 ml) was added to a solution of the crude cycoheptadiene in THF (10 ml) at 0 °C and warm to room temperature for 4h, water was added to the reaction, and the layers were separated. The standard work-up gave a crude product which was purified by flash chromatography, eluting with 1:1 petrol-EtOAc to afford the title product as a yellow oil (60 mg, 60%); $R_f = 0.35$; ν_{max} /cm^{-1}(neat): 3330, 2977, 1643, 1525, 1216, 752, 697; **^{1}H NMR** (400 MHz, Chloroform-*d*) δ 7.46 – 7.41 (m, 2H), 7.40 – 7.29 (m, 3H), 6.77 (t, $J = 6.2$ Hz, 1H), 6.73 (s, 1H), 5.63 (s, 1H), 3.52 (s, 2H), 3.17 (d, $J = 6.2$ Hz, 2H), 1.41 (s, 9H).. **^{13}C NMR** (101 MHz, Chloroform-*D*) δ 209.71, 165.86, 140.85, 139.11, 138.24, 129.19, 128.78, 128.45, 126.36, 122.78, 51.79, 49.07, 44.41, 28.87. **HRMS** (ESI+): m/z calcd for $C_{18}H_{21}NO_2Na$ [M+Na]+ 306.1464, found 306.1473.

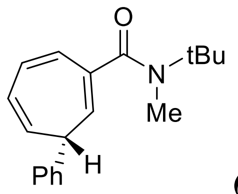

***(R)*-*N*-(*tert*-butyl)-*N*-methyl-3-phenylcyclohepta-1,4,6-triene-1-carboxamide (8):** To a suspension of chiral amine (2.0 equiv) in dry THF (0.1) at –78 ºC (Cryostat), was added n-butyl lithium (3.0 equiv, 2.5 M in hexanes), dropwise. The resulting mixture was allowed to warm to room temperature over 15 minutes, resulting in a clear yellow solution. This was then cooling down to -78 °C, a solution of amide **2a** (100 mg, 0.37 mmol) in THF (0.5 mL) was added dropwise and the reaction mixture was stirred at 700 rpm and irradiated with a 40 W Kessil lamp. The mixture was stirred at –78 ºC for 30 minutes, then the temperature of the Cryostat was set to -10 ºC and the reaction mixture was slowly allowed to warm. After a further 1 h, DMPU (3.0 equiv.) was added and further irradiation was continued for 2.5 h. Stop the irradiation and 5.0 equiv iodomethane was added to reaction mixture and reaction further run for 2 h at -10 ºC. The reaction was quenched with sat. aq. NH_4Cl (15 mL), and the aqueous layer was extracted with EtOAc (3 × 10 mL). The combined organic fractions were washed with brine, dried (Na_2SO_4) and evaporated to yield a crude product, which was purified by flash chromatography, eluting with 90:10 petrol-EtOAc afforded the title compound (71 mg, 68%) as a colorless needles (**MP**: 107-109 ºC). R_f = 0.40; $[\alpha]_D^{22.6}$ = 92 (c = 1.00 in $CHCl_3$); ν_{max} /cm^{-1}(neat): 2972, 1636, 1365, 1066, 699; **^{1}H NMR** (400 MHz, Chloroform-*d*) δ 7.37 – 7.34 (m, 4H), 7.27 (d, *J* = 1.4 Hz, 1H), 6.91 – 6.85 (m, 1H), 6.80 (dd, *J* = 11.1, 5.6 Hz, 1H), 6.29 – 6.23 (m, 1H), 5.51 (ddq, *J* = 9.2, 5.6, 0.8 Hz, 1H), 5.43 – 5.38 (m, 1H), 2.71 (td, *J* = 5.7, 1.5 Hz, 1H), 2.66 (s, 3H), 1.40 (s, 9H). **^{13}C NMR** (101 MHz, Chloroform-*d*) δ 172.65, 143.19, 136.00, 131.73, 129.67, 128.84, 127.70, 126.91, 126.38, 124.87, 124.13, 56.48, 44.62, 34.38, 27.78. **HRMS** (ESI+): m/z calcd for $C_{19}H_{23}NONa$ [M+Na]+ 304.1671, found 304.1671; **HPLC**: *er* 9:91; CHIRALPAK® IA, Hexane:IPA = 95:5 flow = 1.0 mL/min, λ = 280 nm, tR = 5.79 mins (minor), and 6.65 mins (major).

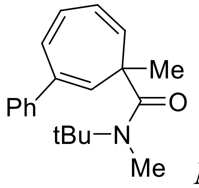

***N*-(tert-butyl)-N,1-dimethyl-3-phenylcyclohepta-2,4,6-triene-1-carboxamide (9):** The *(R)*-*N*-(*tert*-butyl)-*N*-methyl-3-phenylcyclohepta-1,4,6-triene-1-carboxamide (**8**, 100 mg,

0.35 mmol) was dissolved in dry THF (0.1 M) under a nitrogen atmosphere. After cooling down to -10 °C (Cryostat), freshly prepared LDA (1.5 equiv) was sequentially added dropwise and the reaction mixture was stirred at 700 rpm. After 15 mins, iodomethane (4.0 equiv.) was added and the reaction was continued for 2 h. The reaction was quenched with sat. aq. NH_4Cl (5 mL), and the aqueous layer was extracted with EtOAc (3 × 10 mL). The combined organics were washed with brine (1 × 10 mL), dried over MgSO4 and concentrated under reduced pressure to give a crude residue. Purification by flash silica chromatography (10:90 EtOAC/Hexane) afforded the title compound as yellow oil. R_f = 0.40; ν_{max} /cm^{-1}(neat): 2971, 1634, 1363, 1217, 1143, 1074, 752, 698; **^{1}H NMR** (400 MHz, Chloroform-*d*) δ 7.22 – 7.17 (m, 2H), 7.17 – 7.11 (m, 2H), 7.10 – 7.05 (m, 1H), 6.38 – 6.35 (m, 2H), 6.29 (ddd, *J* = 8.9, 5.1, 2.1 Hz, 1H), 5.33 – 5.27 (m, 2H), 2.69 (s, 3H), 1.53 (s, 3H), 1.45 (s, 9H). **^{13}C NMR** (101 MHz, Chloroform-*d*) δ 173.07, 146.63, 136.76, 130.12, 128.91, 128.12, 127.39, 126.77, 125.88, 125.73, 123.41, 120.59, 56.45, 40.74, 34.16, 30.83, 27.87. **HRMS** (ESI+): m/z calcd for $C_{20}H_{25}NONa$ [M+Na]+ 318.1834, found 318.1834. **HPLC**: *er* 53:47; CHIRALPAK® IA, Hexane:IPA = 99:1 flow = 1.0 mL/min, λ = 280 nm, tR = 6.70 mins, and 7.39 mins.

1.18 Analytical data of stereospecific photochemical ring expansion

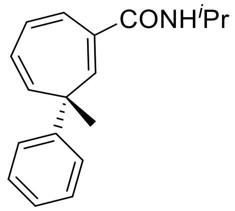

(*R*)-*N*-isopropyl-3-methyl-3-phenylcyclohepta-1,4,6-triene-1-carboxamide (11a): The *(R)-N*-isopropyl-*N*-(1-phenylethyl)benzamide **10a** (100 mg, 0.37 mmol) was dissolved in dry THF (0.1 M) under a nitrogen atmosphere. After cooling down to -10 °C (Cryostat), freshly prepared LDA (2.0 equiv) and DMPU (6.0 equiv) were sequentially added dropwise and the reaction mixture was stirred at 700 rpm and irradiated with a 40 W Kessil lamp for 8 h at -10 °C. The reaction was quenched with sat. aq. NH_4Cl (15 mL), and the aqueous layer was extracted with EtOAc (3 × 10 mL). The combined organics were washed with brine (1 × 10 mL), dried over MgSO4 and concentrated under reduced pressure to give a crude residue. Purification by flash silica chromatography (20% EtOAC/Hexane) afforded the title compound (90 mg, 90%) as pale yellow needles (**MP**: 104-106 ºC); R_f = 0.17; $[\alpha]_D^{23}$ = -92 (c = 1.00 in $CHCl_3$); ν_{max} /cm^{-1} (neat): 3304, 2971, 1634, 1527, 1445, 1055, 749, 698; **^{1}H NMR** (400 MHz, Chloroform-*d*) δ 7.20 – 7.13 (m,

4H), 7.13 – 7.08 (m, 1H), 6.63 – 6.56 (m, 1H), 6.36 – 6.27 (m, 2H), 5.63 (d, *J* = 7.8 Hz, 1H), 5.18 (dd, *J* = 3.4, 1.2 Hz, 1H), 4.71 – 4.64 (m, 1H), 4.18 (dp, *J* = 7.8, 6.5 Hz, 1H), 1.48 (s, 3H), 1.21 (d, *J* = 6.5 Hz, 6H). **^{13}C NMR** (101 MHz, Chloroform-*d*) δ 167.05, 145.39, 131.69, 128.84, 127.30, 126.99, 126.88, 125.95, 125.77, 104.70, 41.63, 35.21, 28.40, 22.63. **HRMS** (ESI+): m/z calcd for $C_{18}H_{21}NONa$ [M+Na]+ 290.1515, found 290.1525; **HPLC**: er 0.42:99.57, Chiral Whelk O1, Hexane-IPA (80:20), flow = 1.0 mL/min, λ = 280 nm tR = 15.472 mins(minor) and 20.112 mins (major).

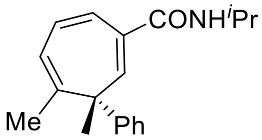

***(R)-N-isopropyl-3,4-dimethyl-3-phenylcyclohepta-1,4,6-triene-1-carboxamide* (11b)**: The *(R)-N*-isopropyl-3-methyl-*N*-(1-phenylethyl)benzamide **10b** (100 mg, 0.35 mmol) was dissolved in dry THF (0.1 M) under a nitrogen atmosphere. After cooling down to -10 °C (Cryostat), freshly prepared LDA (2.0 equiv) and DMPU (6.0 equiv) were sequentially added dropwise and the reaction mixture was stirred at 700 rpm and irradiated with a 40 W Kessil lamp for 4 h at -10 °C. The reaction was quenched with sat. aq. NH_4Cl (15 mL), and the aqueous layer was extracted with EtOAc (3 × 10 mL). The combined organics were washed with brine (1 × 10 mL), dried over MgSO4 and concentrated under reduced pressure to give a crude residue. Purification by flash silica chromatography (25% EtOAC/Hexane) afforded the title compound (80 mg, 80%) as pale-yellow oil. R_f = 0 40; $[\alpha]_D^{22.7}$ = -80 (c = 1.00 in $CHCl_3$); ν_{max} /cm^{-1}(neat): 3318, 2971, 1635, 1527, 1445, 1066, 749, 699; **^{1}H NMR** (400 MHz, Chloroform-*d*) δ 4.41 – 4.32 (m, 5H), 3.73 (dq, *J* = 7.8, 3.4 Hz, 1H), 3.56 – 3.46 (m, 2H), 3.08 (s, 1H), 2.96 – 2.80 (m, 1H), 1.47 (dp, *J* = 13.5, 6.8 Hz, 1H), -0.62 (s, 3H), -1.11 (s, 3H), -1.49 (d, *J* = 2.5 Hz, 3H), -1.50 (d, *J* = 2.5 Hz, 3H). **^{13}C NMR** (101 MHz, Chloroform-*d*) δ 167.21, 146.00, 132.08, 130.59, 127.08, 126.78, 125.70, 125.36, 124.31, 122.73, 43.83, 41.88, 29.84, 22.87. **HRMS** (ESI+): m/z calcd for $C_{19}H_{23}NONa$ [M+Na]+ 304.1672, found 304.1682; **HPLC**: er 2: 98 Chiral Whelk O1, Hexane:IPA = 85:15, flow = 1.0 mL/min, λ = 280 nm, tR = 25.838 mins (major) and 28.485 mins (minor).

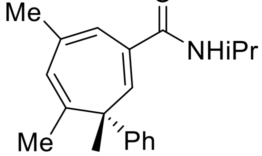

***(S)-N*-isopropyl-3,4,6-trimethyl-3-phenylcyclohepta-1,4,6-triene-1-carboxamide (11c):** The *(R)-N*-isopropyl-3,5-dimethyl-*N*-(1-phenylethyl)benzamide **10c** (100 mg, 0.33 mmol) was dissolved in dry THF (0.1 M) under a nitrogen atmosphere. After cooling down to -10 °C (Cryostat), freshly prepared LDA (2.0 eq.) and DMPU (6.0 equiv) were sequentially added dropwise and the reaction mixture was stirred at 700 rpm and irradiated with a 40 W Kessil lamp for 4 h at -10 °C (Cryostat). The reaction was quenched with sat. aq. NH_4Cl (15 mL), and the aqueous layer was extracted with EtOAc (3 × 10 mL). The combined organics were washed with brine (1 × 10 mL), dried over MgSO4 and concentrated under reduced pressure to give a crude residue. Purification by flash silica chromatography (25% EtOAC/Hexane) afforded the title compound (57 mg, 57%) as colorless oil. R_f = 0.30; $[\alpha]_D^{21}$ = -124 (c = 1.00 in $CHCl_3$); ν_{max} /cm^{-1} (neat): 3294, 2971, 1638, 1533, 1450,; **^{1}H NMR** (400 MHz, Chloroform-*d*) δ 7.08 – 7.01 (m, 5H), 6.16 (s, 1H), 6.00 (s, 1H), 5.76 (s, 1H), 5.57 (d, *J* = 9.4 Hz, 1H), 4.16 (dq, *J* = 13.1, 6.5 Hz, 1H), 2.02 (s, 3H), 1.69 (s, 3H), 1.54 (s, 3H), 1.20 (d, *J* = 2.6 Hz, 3H), 1.18 (d, *J* = 2.6 Hz, 3H). **^{13}C NMR** (101 MHz, Chloroform-*d*) δ 167.55, 146.90, 140.13, 132.17, 127.16, 126.59, 125.69, 122.65, 43.84, 41.87, 29.62, 23.53, 22.96, 22.91, 22.58. **HRMS** (ESI+): m/z calcd for $C_{20}H_{25}NONa$ [M+Na]+ 318.34, found 318.48; **HPLC**: er 90:10 Chiral Whelk O1, Hexane:IPA = 85:15 flow = 1.0 mL/min, λ = 280 nm, tR = 14.81 (major), and 16.01 (minor).

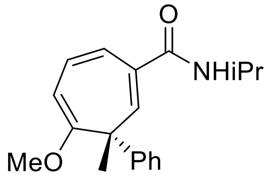

***(R)-N*-isopropyl-4-methoxy-3-methyl-3-phenylcyclohepta-1,4,6-triene-1-carboxamide (11d):** The *(R)-N*-isopropyl-3-methoxy-*N*-(1-phenylethyl)benzamide **10d** (100 mg, 0.33 mmol) was dissolved in dry THF (0.1 M) under a nitrogen atmosphere. After cooling down to -10 °C (Cryostat), freshly prepared LDA (2.0 eq.) and DMPU (6.0 equiv) were sequentially added dropwise and the reaction mixture was stirred at 700 rpm and irradiated with a 40 W Kessil lamp for 8 h at -10 °C. The reaction was quenched with sat. aq. NH_4Cl (15 mL), and the aqueous layer was extracted with EtOAc (3 × 10 mL). The combined organics were washed with brine (1 × 10 mL), dried over MgSO4 and concentrated under reduced pressure to give a crude residue. Purification by flash silica chromatography (30% EtOAC/Hexane) afforded the title compound (76 mg, 76%) as colorless needles. R_f = 0.40; **MP:** 192-194 ^{o}C; $[\alpha]_D^{21}$ = -124 (c = 1.00 in $CHCl_3$);

ν_{max} /cm^{-1}(neat): 3333, 2971, 1637, 1542, 1415, **^{1}H NMR** (400 MHz, Chloroform-*d*) δ 7.04 – 6.93 (m, 5H), 6.17 (d, *J* = 6.6 Hz, 2H), 6.10 (s, 1H), 5.56 (d, *J* = 7.1 Hz, 1H), 5.48 (d, *J* = 6.0 Hz, 1H), 4.16 – 4.05 (m, 1H), 3.69 (s, 3H), 1.57 (s, 3H), 1.14 (dd, *J* = 6.6, 3.3 Hz, 7H). **^{13}C NMR** (101 MHz, Chloroform-*d*) δ 167.27, 158.86, 145.02, 133.66, 131.79, 129.57, 128.27, 126.97, 126.07, 125.83, 120.06, 98.05, 56.94, 48.43, 41.88, 29.53, 22.82. **HRMS** (ESI+): m/z calcd for $C_{19}H_{23}NO_2Na$ [M+Na]+ 320.1621, found 320.1632; **HPLC**: er 100:00 Chiral Whelk O1, Hexane:IPA = 85:15, flow = 1.0 mL/min, λ = 280 nm, tR = 17.575.

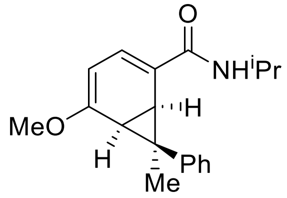

(1*R*,6*S*,7*R*)-*N*-isopropyl-5-methoxy-7-methyl-7-phenylbicyclo[4.1.0]hepta-2,4-diene-2-carboxamide (11e): The *(R)*-*N*-isopropyl-4-methoxy-*N*-(1-phenylethyl)benzamide **10e** (100 mg, 0.33 mmol) was dissolved in dry THF (0.1 M) under a nitrogen atmosphere. After cooling down to -10 °C (Cryostat), freshly prepared LDA (2.0 equiv) and DMPU (6.0 equiv) were sequentially added dropwise and the reaction mixture was stirred at 700 rpm and irradiated with a 40 W Kessil lamp for 5 h at -10 °C (Cryostat). The reaction was quenched with sat. aq. NH_4Cl (15 mL), and the aqueous layer was extracted with EtOAc (3 × 10 mL). The combined organics were washed with brine (1 × 10 mL), dried over $MgSO_4$ and concentrated under reduced pressure to give a crude residue. Purification by flash silica chromatography (30% EtOAC/Hexane) afforded the title compound (85 mg, 85%) as colorless needles (**MP**: 114-116 ºC). R_f = 0.17; $[\alpha]_D^{21}$ = -36 (c = 1.00 in $CHCl_3$); ν_{max} /cm^{-1}(neat): 3336, 2971, 1555, 1219, 1174, 1027, 748, 699; **^{1}H NMR** (400 MHz, Chloroform-*d*) δ 7.26 – 7.16 (m, 4H), 7.11 (ddd, *J* = 8.6, 5.4, 2.6 Hz, 1H), 6.57 (d, *J* = 7.1 Hz, 1H), 5.49 (d, *J* = 6.3 Hz, 1H), 4.97 (d, *J* = 7.1 Hz, 1H), 4.14 (dq, *J* = 13.3, 6.6 Hz, 1H), 3.63 (s, 3H), 2.58 (dd, *J* = 9.1, 0.9 Hz, 1H), 2.31 (dd, *J* = 9.1, 1.2 Hz, 1H), 1.17 – 1.11 (m, 9H). **^{13}C NMR** (101 MHz, $CDCl_3$) δ 166.84, 161.24, 128.52, 128.36, 128.01, 126.29, 121.22, 93.11, 55.77, 41.52, 34.03, 33.31, 23.02, 16.14. **HRMS** (ESI+): m/z calcd for $C_{19}H_{23}NO_2Na$ [M+Na]+ 320.1621, found 320.1632; **HPLC**: *er* 2:98; Chiral Whelk O1, Hexane:IPA = 70:30, flow = 1.0 mL/min, λ = 280 nm, tR = 14.25 (minor), and 22.63 (major).

1.19 Analytical data of carbolithiation and photochemical rearrangements

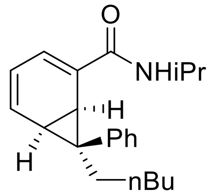

(1*R*,6*S*,7*S*)-*N*-isopropyl-7-pentyl-7-phenylbicyclo[4.1.0]hepta-2,4-diene-2-carboxamide (14): To a stirred solution of freshly distilled (−)-sparteine (2 equiv.) in dry diethyl ether (3.7 mL) at −78 °C, RLi (2 equiv.) was added resulting in a pale-yellow solution. The reaction was left to stir at the same temperature for 15 min. before the *N*-isopropyl-*N*-(1-phenylvinyl)benzamide (0.37 mmol, 1 equiv.) in solution in dry diethyl ether (1 mL) was slowly added dropwise resulting in a red-orange solution and irradiated with a 40 W Kessil lamp. The reaction was warm up to -10 ºC and stirred for 5 h. The reaction was quenched with sat. aq. NH_4Cl (15 mL), and the aqueous layer was extracted with EtOAc (3 × 10 mL). The combined organics were washed with brine (1 × 10 mL), dried over MgSO4 and concentrated under reduced pressure to give a crude residue. Purification by flash silica chromatography (10% EtOAC/Hexane) afforded the title compound (50 mg, 41%) as yellow oil. R_f = 0.17; $[\alpha]_D^{25}$ = -68 (c = 1.00 in $CHCl_3$); ν_{max} /cm^{-1}(neat): 3334, 2971, 1560, 1230, 1174,; **^{1}H NMR** (400 MHz, Chloroform-*d*) δ 7.18 – 7.05 (m, 5H), 6.47 (d, *J* = 8.6 Hz, 1H), 6.29 (t, *J* = 7.4 Hz, 1H), 6.16 – 6.07 (m, 1H), 5.61 (d, *J* = 7.7 Hz, 1H), 4.32 (d, *J* = 5.6 Hz, 1H), 4.25 – 4.14 (m, 1H), 3.89 (dd, *J* = 7.4, 5.0 Hz, 1H), 1.67 – 1.59 (m, 2H), 1.19 (dqd, *J* = 13.5, 7.5, 6.7, 2.8 Hz, 12H), 0.82 – 0.75 (m, 3H). **^{13}C NMR** (101 MHz, Chloroform-*d*) δ 167.36, 142.45, 131.09, 129.34, 128.08, 127.37, 126.87, 126.08, 125.50, 80.21, 79.76, 41.83, 39.89, 33.97, 32.15, 25.52, 22.99, 22.60, 14.09. **HRMS** (ESI+): m/z calcd for $C_{22}H_{29}NONa$ $[M+Na]^+$ 346.2141, found 346.2137; **HPLC**: er 27:73, Chiral Whelk O1, Hexane:IPA = 92:08, flow = 1.0 mL/min, λ = 254 nm, tR = 28.04 (minor) and 29.3 (major) min.

2 Mechanistic Study

2.1 experimental setup of ReactIR

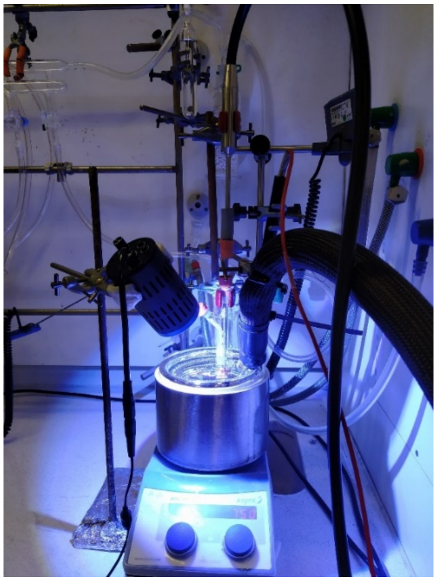

2.1.1 React-IR study

Starting material peaks with THF+DMPU in background

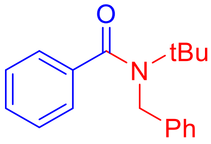

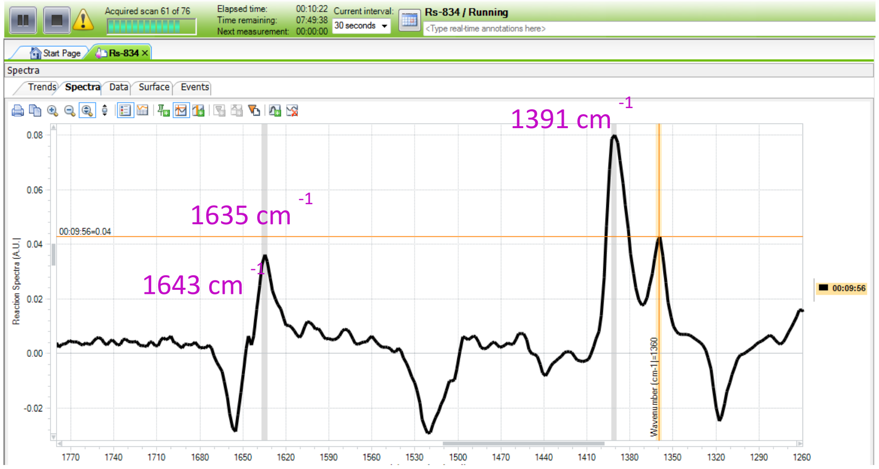

LDA addition

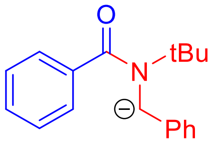

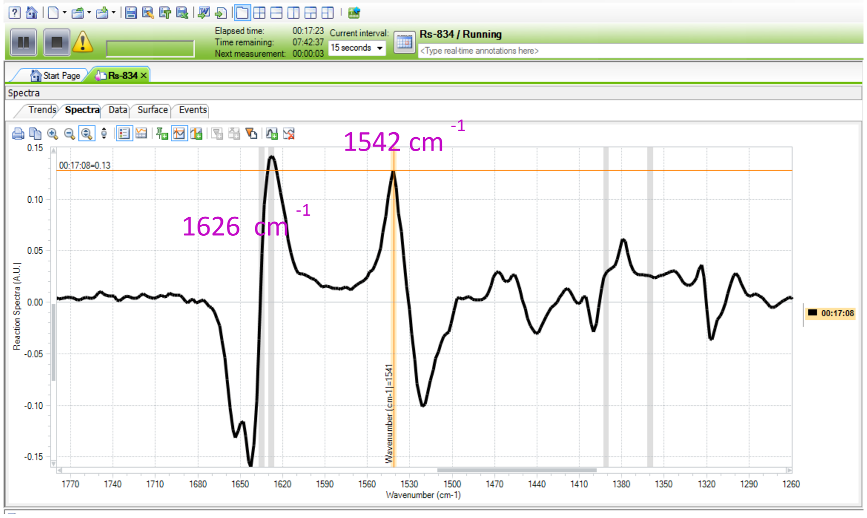

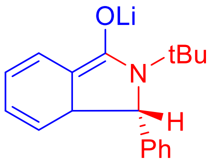

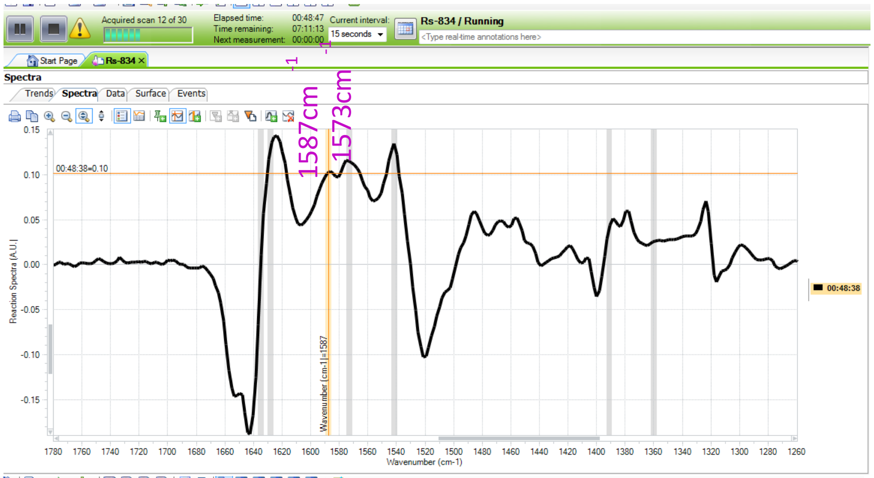

After (1.14 h)

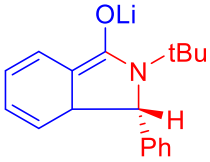

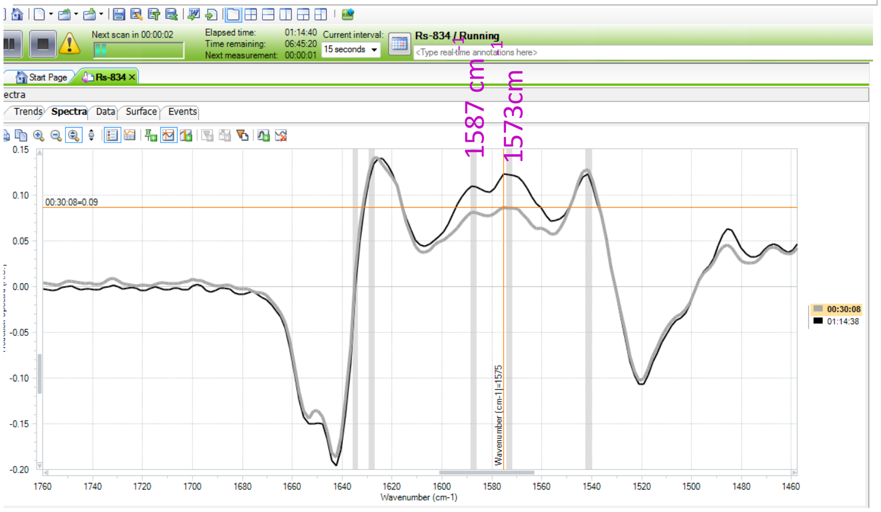

dearomatizing cyclization done Confirmed by TLC

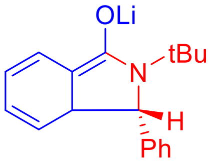

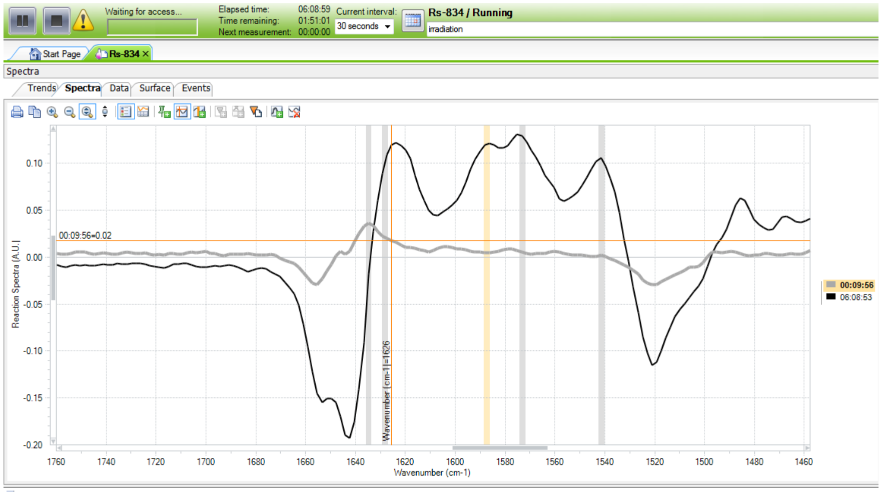

Irradiation start (after 10 mins of irradiation)

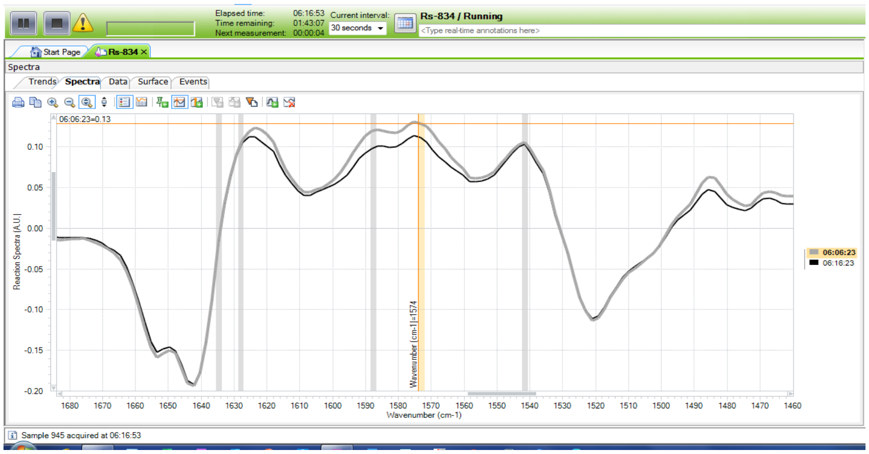

After 50 mins of Irradiation

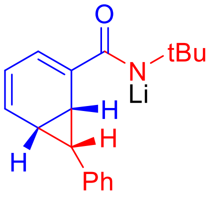

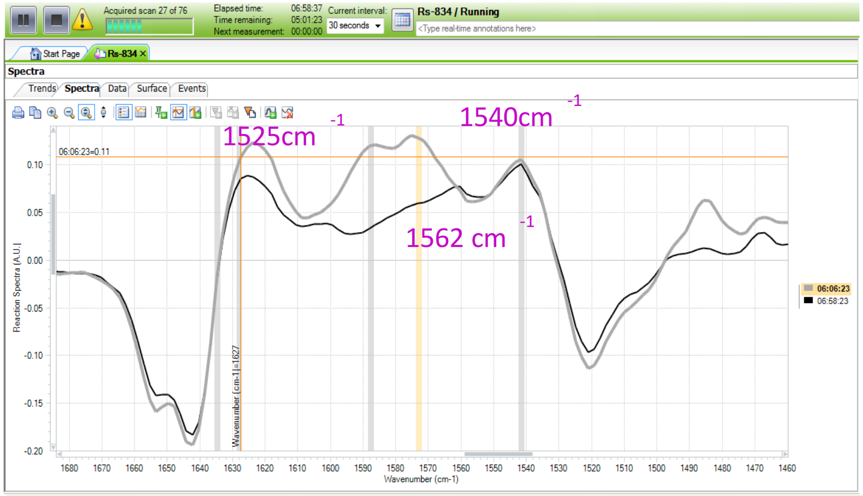

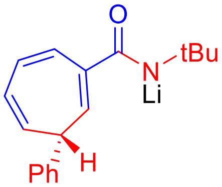

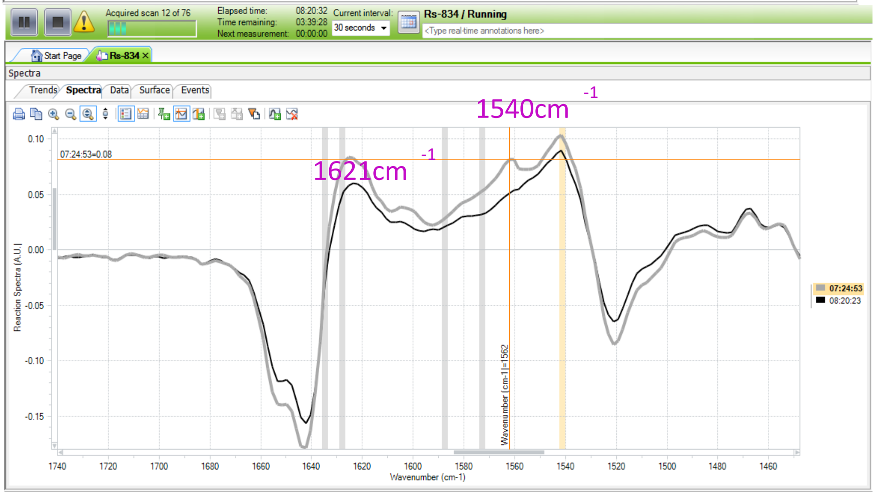

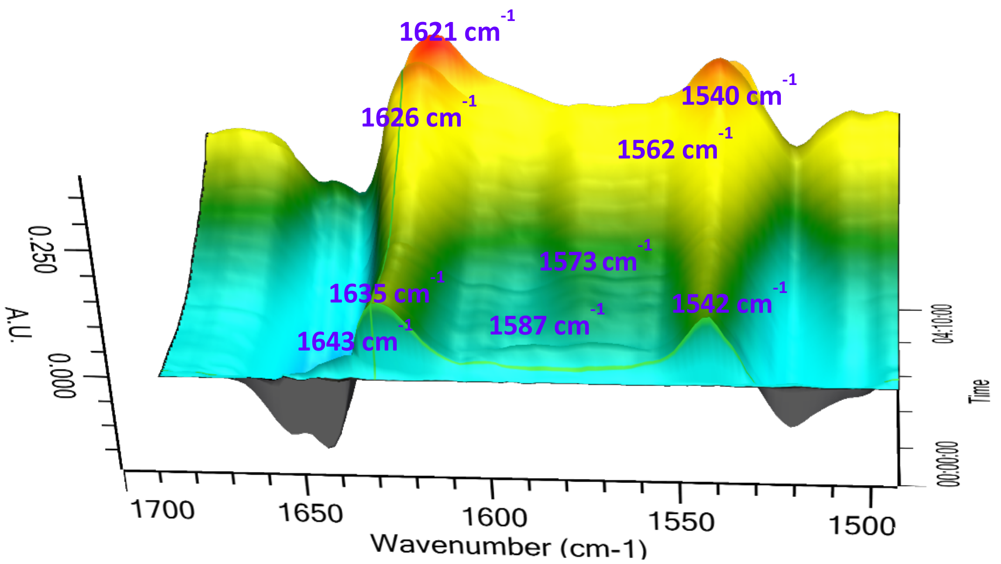

1621 cm-1
1626 cm-1
1540 cm-1
1562 cm-1
1573 cm-1
1635 cm-1
1542 cm-1
1587 cm-1
1643 cm-1
0.250
0.000
A.U.
04:10:00
00:00:00
Time
1700
1650
1600
1550
1500
Wavenumber (cm-1)

2.2 NMR Study

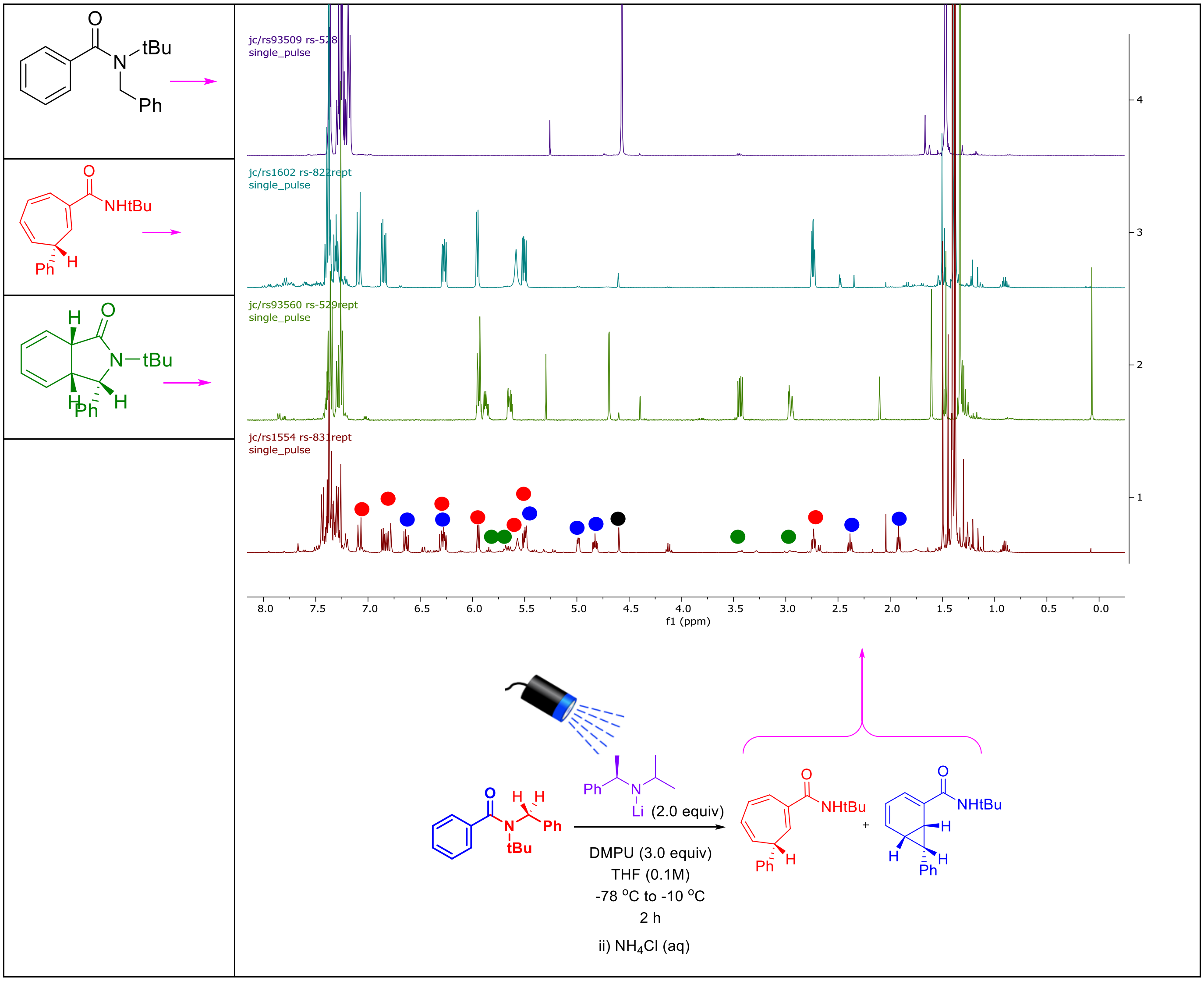

jc/rs93509 rs-528
single_pulse
jc/rs1602 rs-822rept
single_pulse
jc/rs93560 rs-529rept
single_pulse
jc/rs1554 rs-831rept
single_pulse
f1 (ppm)
NHtBu
N-tBu
(2.0 equiv)
DMPU (3.0 equiv)
THF (0.1M)
-78 °C to -10 °C
2 h
ii) NH4Cl (aq)

Reaction monitoring by NMR

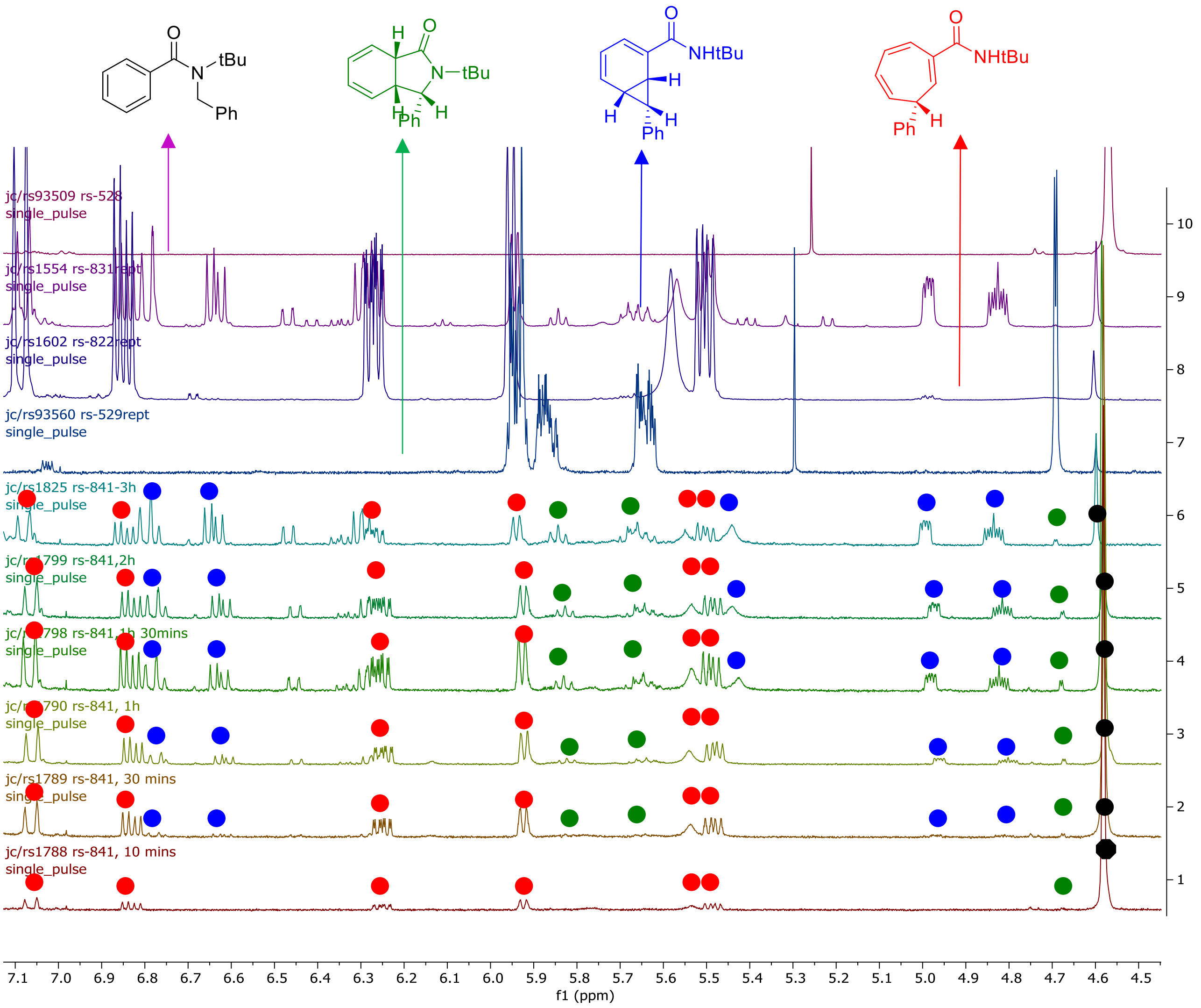

NMR ratio after 3 h and Final ratio

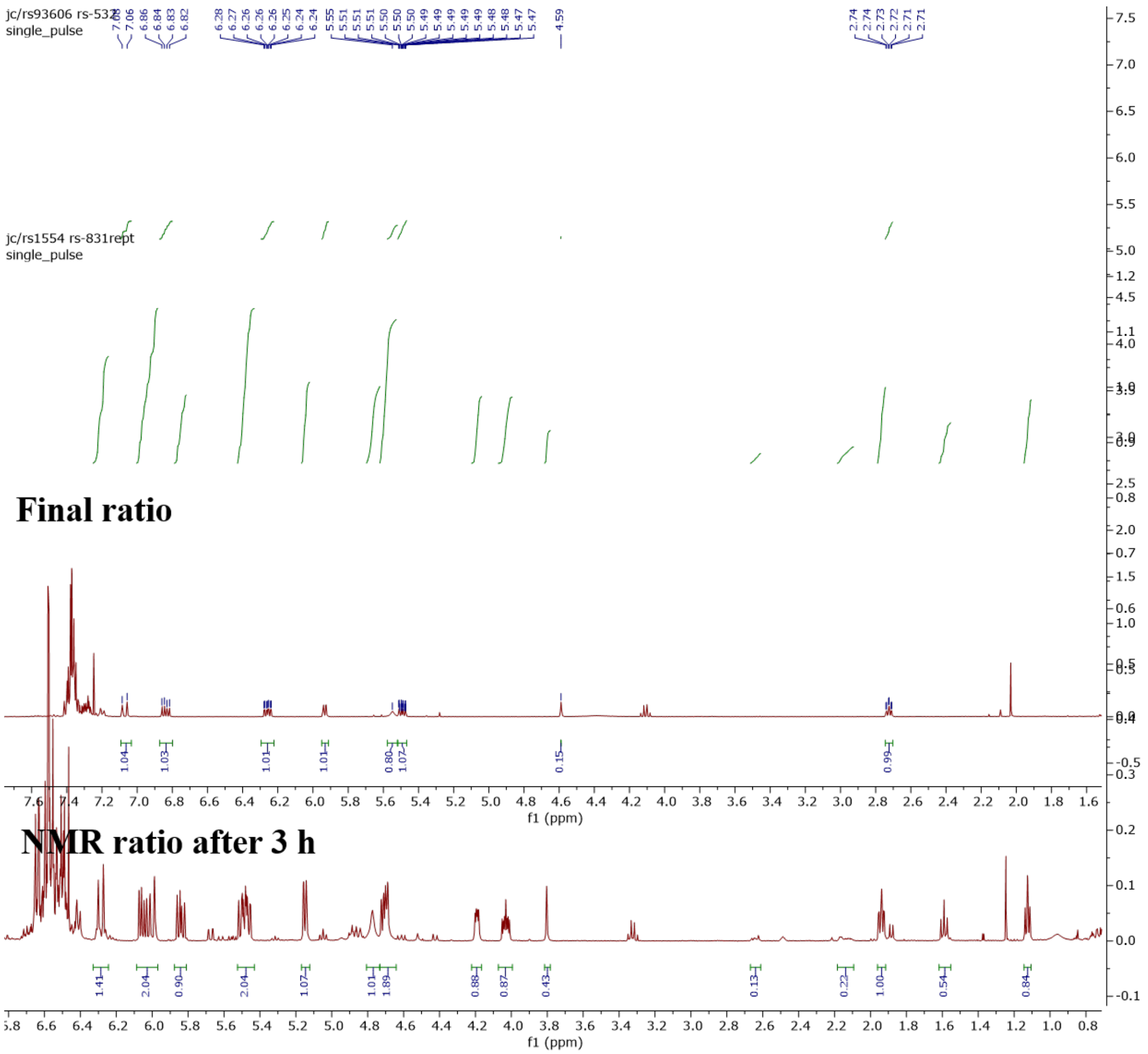

2.2 VCD spectra

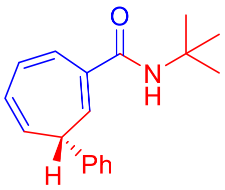

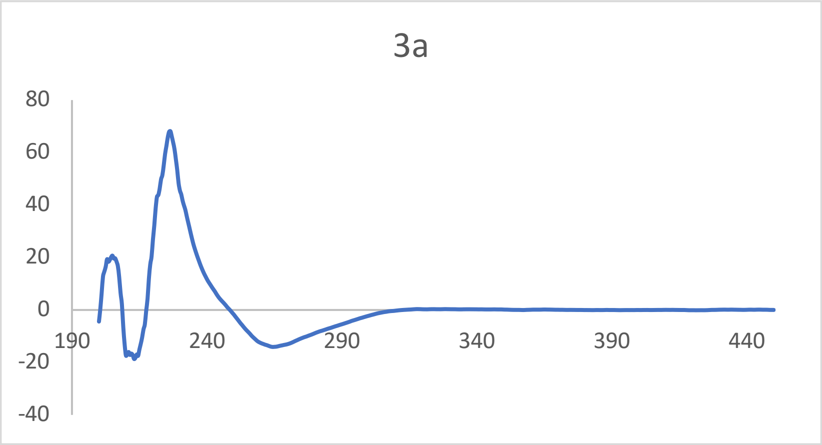

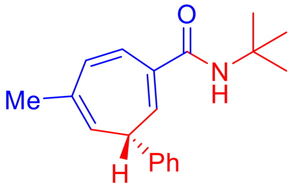

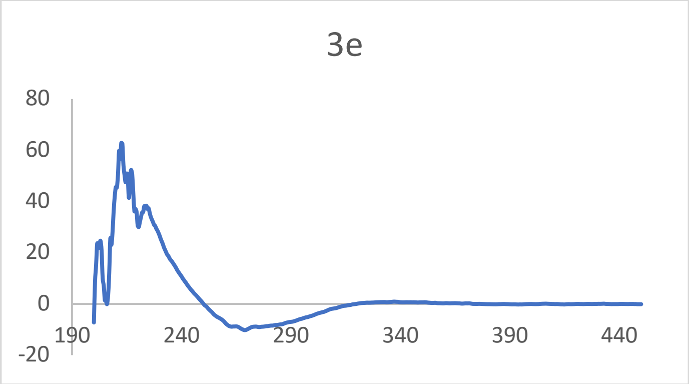

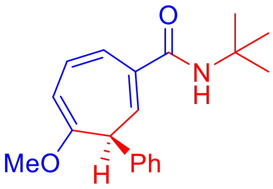

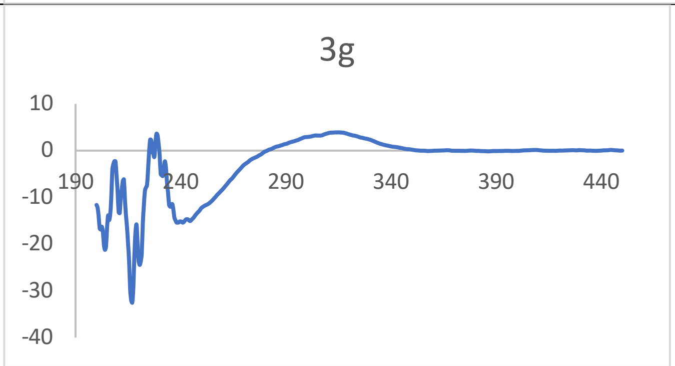

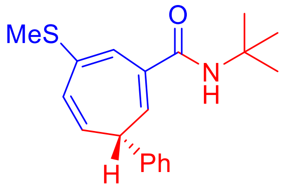
MeS
O
N
H
H
Ph

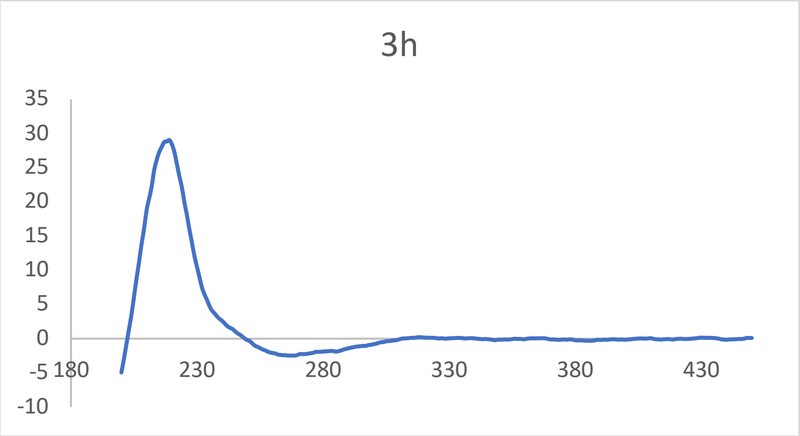
3h
35
30
25
20
15
10
5
0
-5
-10
180
230
280
330
380
430

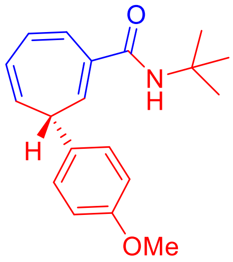
O
N
H
H
OMe

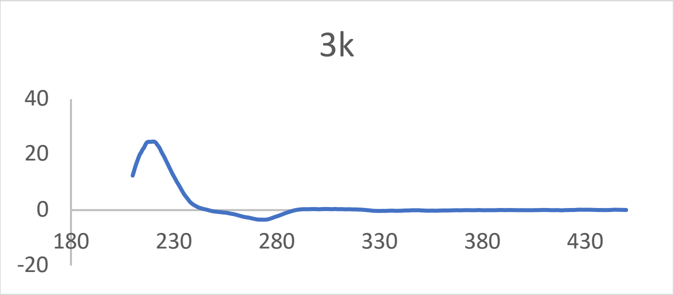
3k
40
20
0
-20
180
230
280
330
380
430

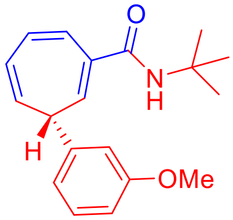
O
N
H
H
OMe

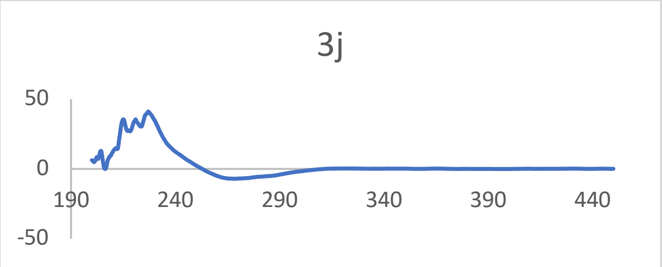
3j
50
0
-50
190
240
290
340
390
440

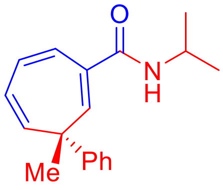
O
N
H
Me
Ph

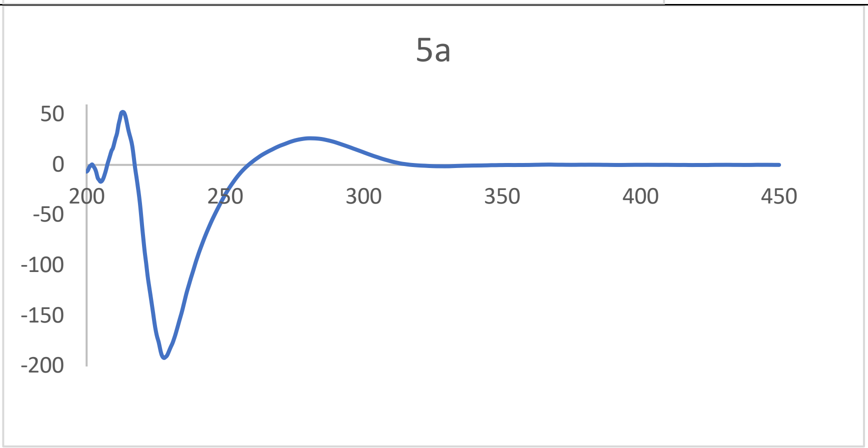
5a
50
0
-50
-100
-150
-200
200
250
300
350
400
450

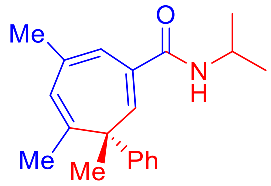
Me
O
N
H
Me
Me
Ph

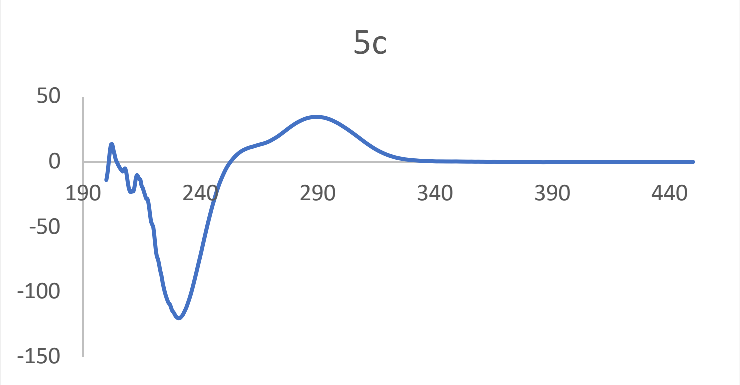
5c
50
0
-50
-100
-150
190
240
290
340
390
440

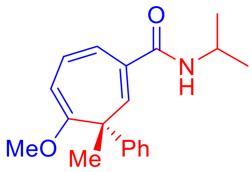
O
N
H
MeO
Me
Ph

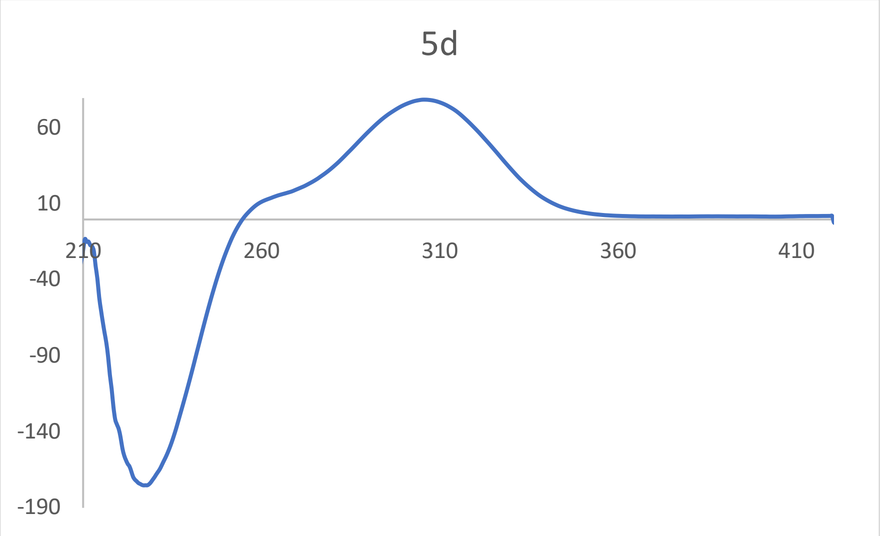
5d
60
10
-40
-90
-140
-190
210
260
310
360
410

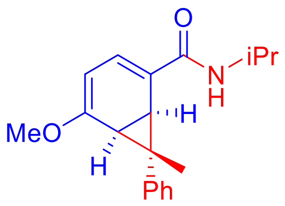
O
N
H
iPr
MeO
H
H
Ph

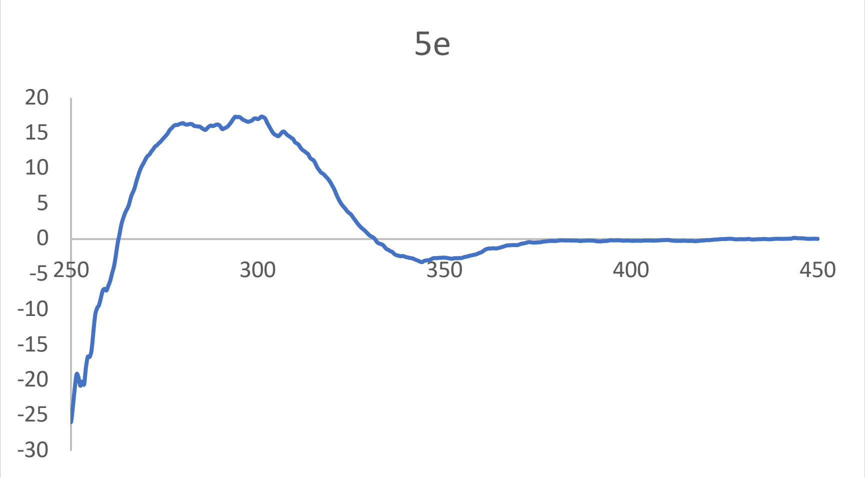
5e
20
15
10
5
0
-5
-10
-15
-20
-25
-30
250
300
350
400
450

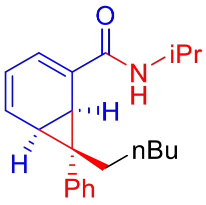 O<br>N<br>H<br>iPr<br>H<br>H<br>nBu<br>Ph	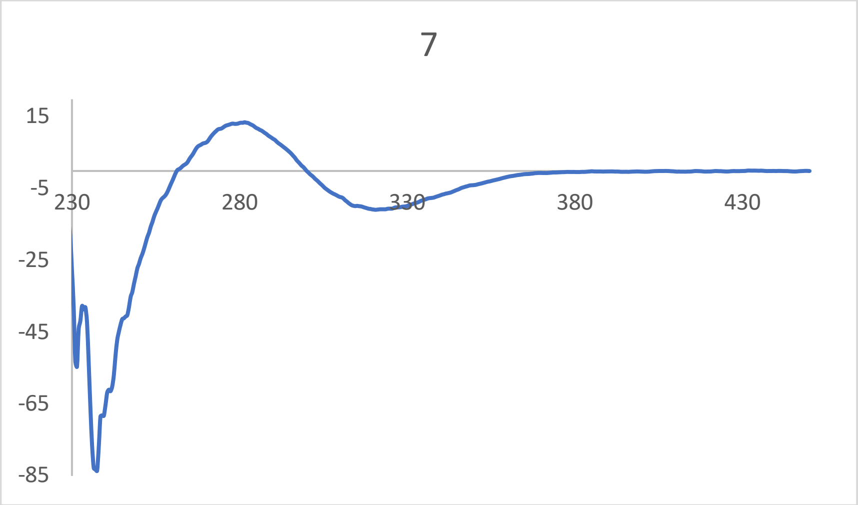 7<br>15<br>-5<br>-25<br>-45<br>-65<br>-85<br>230<br>280<br>330<br>380<br>430

Time-Dependent DFT Calculations

Computational characterizations of the ground- and excited-state properties of the enolate were performed using Gaussian 16 software[7]. The ground electronic state geometry of the enolate was computed using density functional theory (DFT). The B3LYP functional was used with the 6-31+G(d,p) basis set. Solvent effects for THF were included by implicit simulation of solvation as a conductor-like polarizable continuum medium. Vertical transition energies, oscillator strengths and excitation amplitudes were computed using time-dependent density functional theory (TD-DFT) using the Coulomb attenuated variant of the B3LYP functional (CAM-B3LYP). CAM-B3LYP was used with the 6-31+G(d,p) basis set and conductor-like continuum simulation of THF as solvent.

Cartesian Coordinates of Optimised Structure

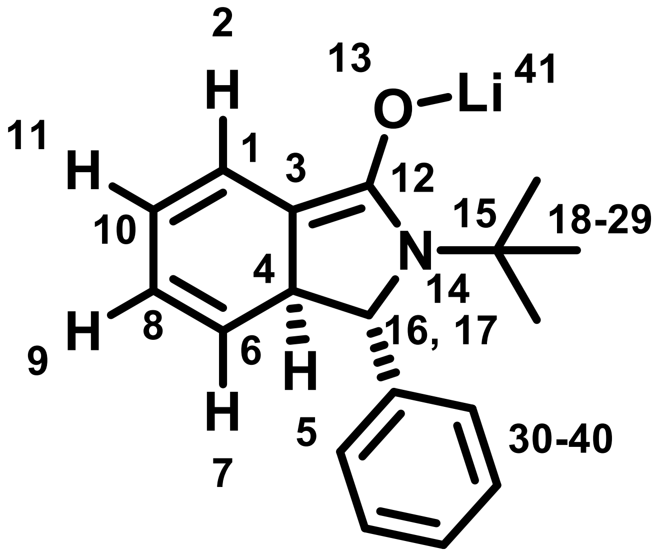

7

Tag	Symbol	X	Y	Z
1	C	-3.1034110	-1.5153440	-0.5790170
2	H	-4.0000200	-1.0658490	-1.0067870
3	C	-1.9312920	-0.7477870	-0.4228190
4	C	-0.6286430	-1.4240440	-0.0889220
5	H	-0.1441670	-1.7987030	-1.0162150
6	C	-0.8702700	-2.5858350	0.8455920

[7]Frisch, M. J.; Trucks, G. W.; Schlegel, H. B.; Scuseria, G. E.; Robb, M. A.; Cheeseman, J. R.; Scalmani, G.; Barone, V.; Petersson, G. A.; Nakatsuji, H.; Li, X.; Caricato, M.; Marenich, A. V.; Bloino, J.; Janesko, B. G.; Gomperts, R.; Mennucci, B.; Hratchian, H. P.; Ortiz, J. V.; Izmaylov, A. F.; Sonnenberg, J. L.; Williams; Ding, F.; Lipparini, F.; Egidi, F.; Goings, J.; Peng, B.; Petrone, A.; Henderson, T.; Ranasinghe, D.; Zakrzewski, V. G.; Gao, J.; Rega, N.; Zheng, G.; Liang, W.; Hada, M.; Ehara, M.; Toyota, K.; Fukuda, R.; Hasegawa, J.; Ishida, M.; Nakajima, T.; Honda, Y.; Kitao, O.; Nakai, H.; Vreven, T.; Throssell, K.; Montgomery Jr., J. A.; Peralta, J. E.; Ogliaro, F.; Bearpark, M. J.; Heyd, J. J.; Brothers, E. N.; Kudin, K. N.; Staroverov, V. N.; Keith, T. A.; Kobayashi, R.; Normand, J.; Raghavachari, K.; Rendell, A. P.; Burant, J. C.; Iyengar, S. S.; Tomasi, J.; Cossi, M.; Millam, J. M.; Klene, M.; Adamo, C.; Cammi, R.; Ochterski, J. W.; Martin, R. L.; Morokuma, K.; Farkas, O.; Foresman, J. B.; Fox, D. J. *Gaussian 16 Rev. C.01*, Wallingford, CT, 2016.

7	H	-0.0838380	-2.9158470	1.5220180
8	C	-2.0437770	-3.2703660	0.7399130
9	H	-2.1958230	-4.1656800	1.3419480
10	C	-3.1416040	-2.8111160	-0.0885310
11	H	-4.0427030	-3.4120360	-0.1685380
12	C	-1.7318110	0.6348070	-0.5121380
13	O	-2.5684210	1.5307190	-0.8866810
14	N	-0.4061030	0.9681140	-0.1299630
15	C	-0.1430150	2.3175950	0.4874950
16	C	0.2496590	-0.2435130	0.4315210
17	H	0.1798910	-0.2230750	1.5318700
18	C	-1.1216400	2.5745380	1.6560800
19	H	-0.9014510	3.5385080	2.1278760
20	H	-2.1561920	2.5889470	1.3067590
21	H	-1.0225560	1.7971530	2.4223970
22	C	-0.2826450	3.4120140	-0.5910410
23	H	-1.2906710	3.4341030	-1.0031190
24	H	-0.0532330	4.3903720	-0.1531590
25	H	0.4255930	3.2299410	-1.4068880
26	C	1.2930380	2.4040470	1.0380430
27	H	1.4397010	3.4094540	1.4454500
28	H	1.4791910	1.6927900	1.8467380
29	H	2.0397360	2.2433700	0.2571560
30	C	1.7055120	-0.4388590	0.0404400
31	C	2.6159170	-0.9560080	0.9721610
32	C	2.1525170	-0.2015160	-1.2683780
33	C	3.9402070	-1.2284650	0.6115060
34	H	2.2893300	-1.1405580	1.9930570
35	C	3.4748430	-0.4666820	-1.6326300
36	H	1.4598100	0.2050160	-1.9992900
37	C	4.3760140	-0.9825440	-0.6935730
38	H	4.6293460	-1.6269960	1.3510510
39	H	3.8030410	-0.2700330	-2.6497990
40	H	5.4046990	-1.1874560	-0.9760870
41	Li	-4.2423990	1.8094970	-1.4791560

TDDFT UV-Vis Prediction Data

Transition	Wavelength (nm)	Energy (eV)	Oscillator Strength
$S_0 \rightarrow S_1$	415.00	2.9875	0.2465
$S_0 \rightarrow S_2$	357.99	3.4634	0.0048
$S_0 \rightarrow S_3$	315.33	3.9319	0.0160
$S_0 \rightarrow S_4$	306.60	4.0438	0.0045

Copies of ^{1}H, ^{13}C NMR spectra (all in $CDCl_3$) and HPLC traces

^{1}H NMR

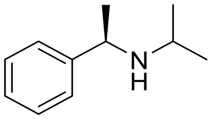

(R)-*N*-(1-Phenylethyl)-1-methylethylamine (1c)

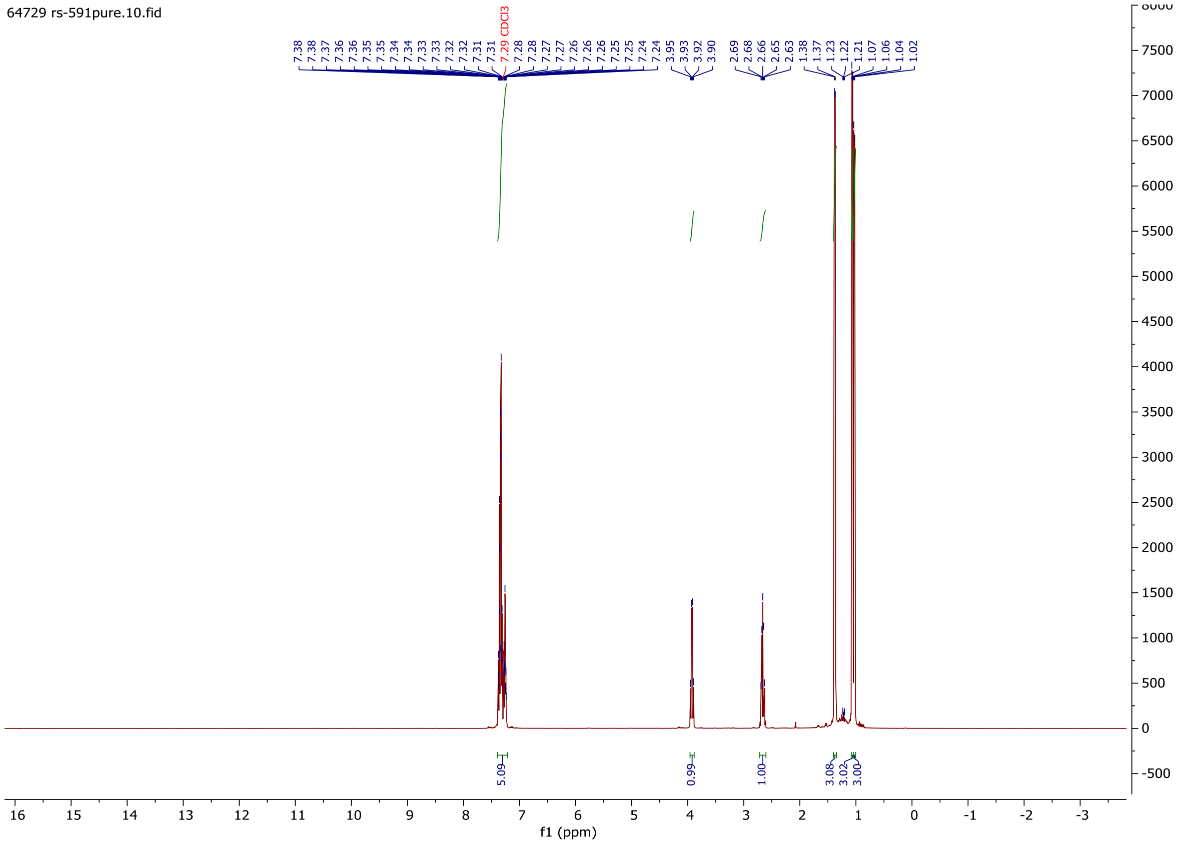

^{13}C NMR

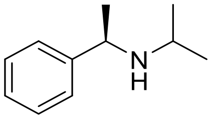

***(R)*-*N*-(1-Phenylethyl)-1-methylethylamine (1c)**

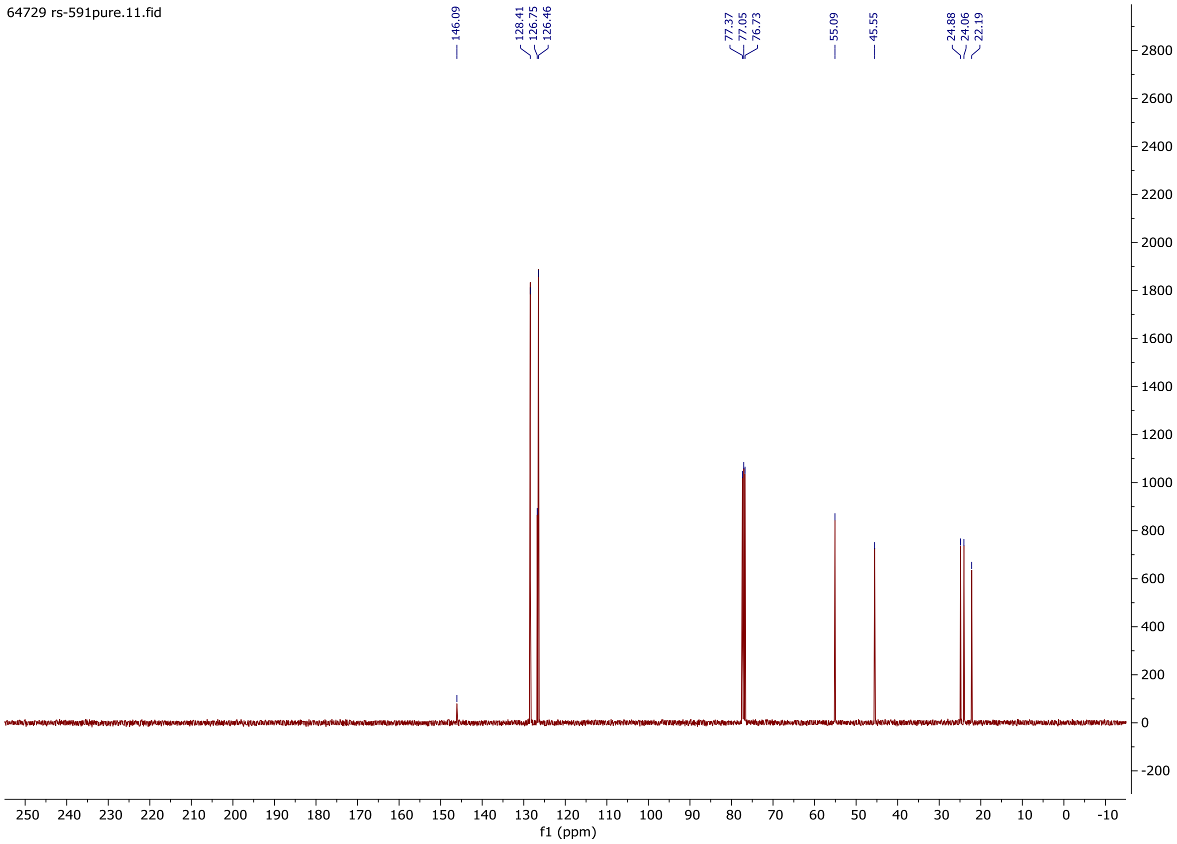

^{1}H NMR

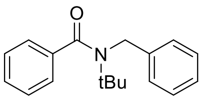

N-benzyl-*N*-(tert-butyl)benzamide (2a)

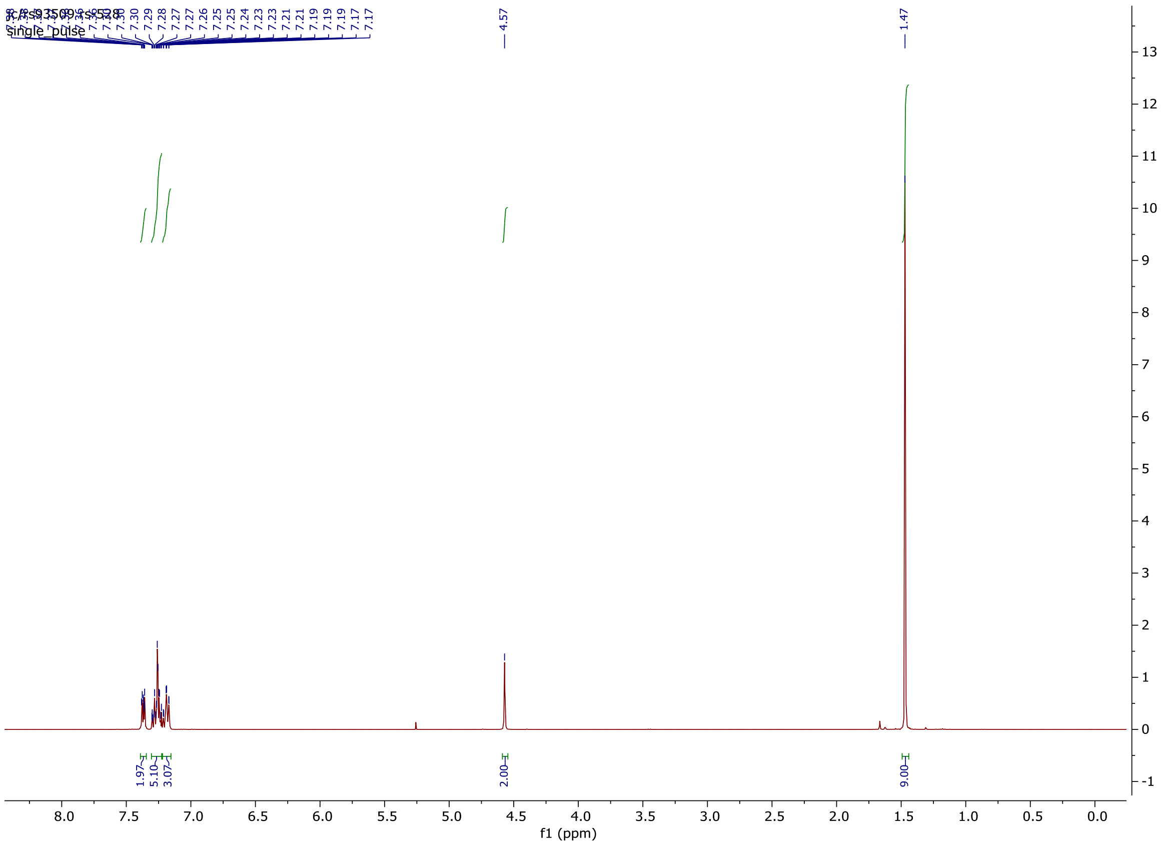

^{13}C NMR

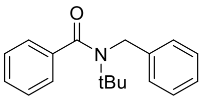

N-benzyl-*N*-(tert-butyl)benzamide (2a)

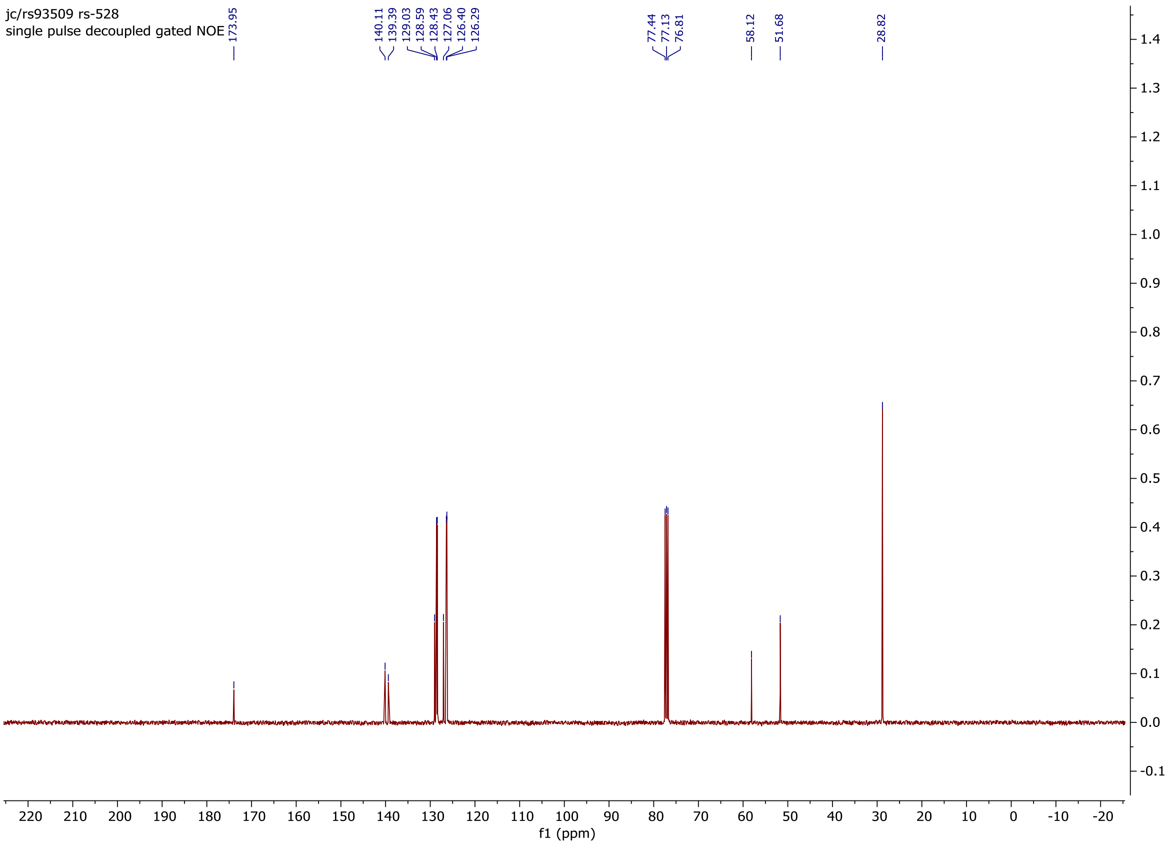

^{1}H NMR

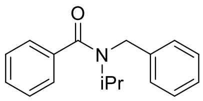

N-benzyl-*N*-isopropylbenzamide (2a’)

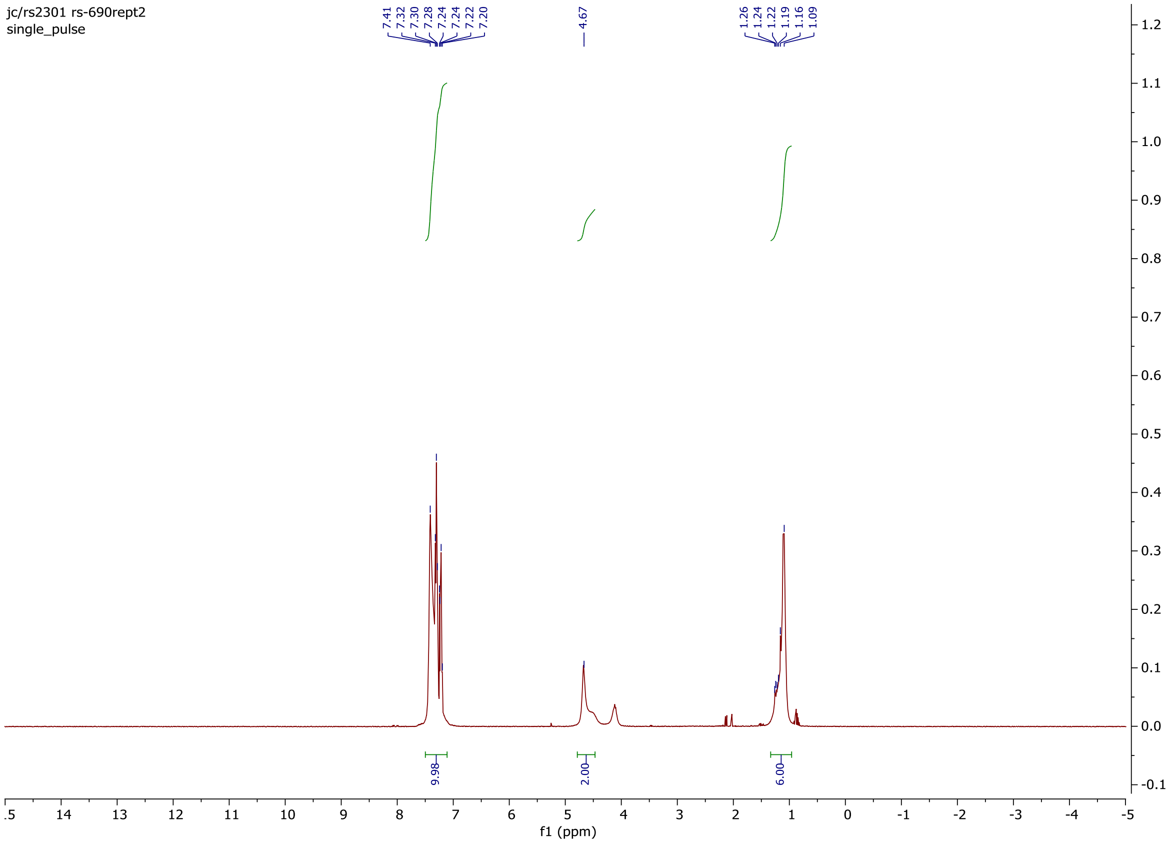

^{13}C NMR

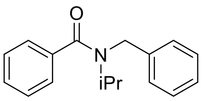

N-benzyl-*N*-isopropylbenzamide (2a')

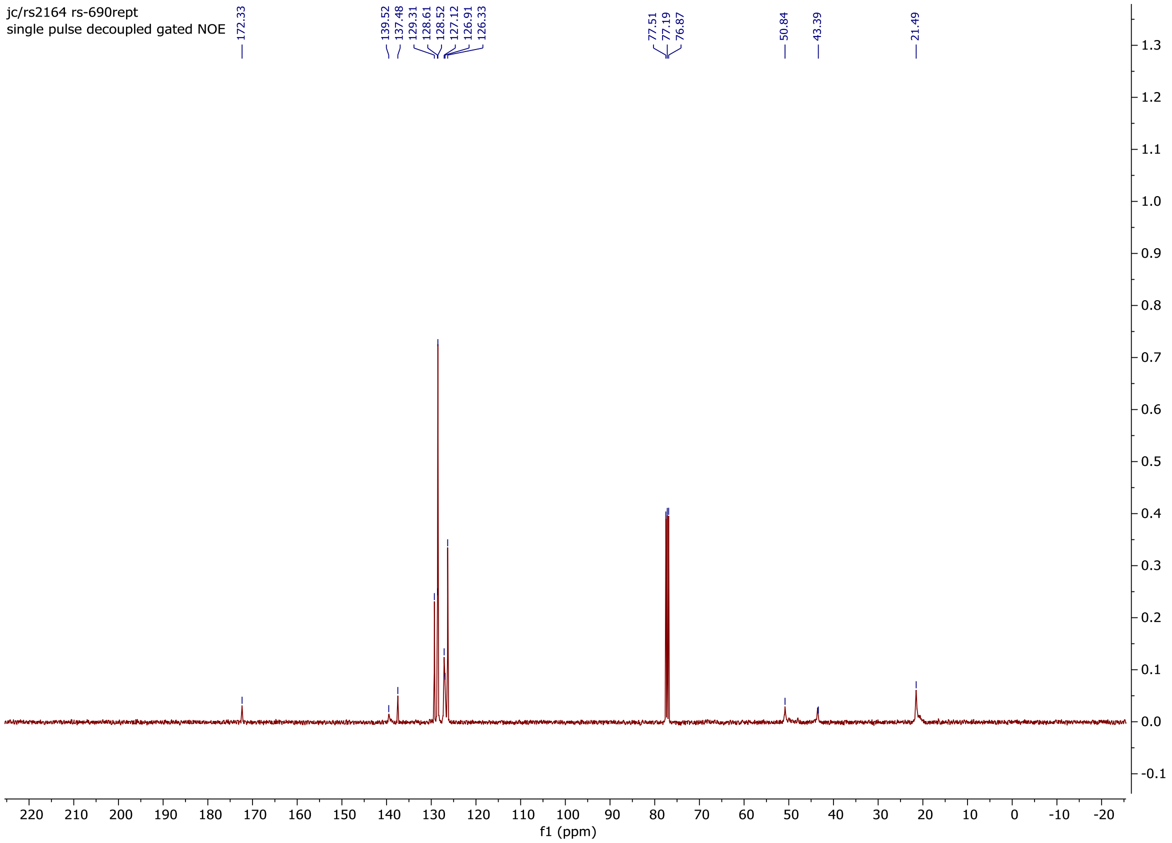

^{1}H NMR

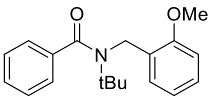

N-(tert-butyl)-*N*-(2-methoxybenzyl)benzamide (2b)

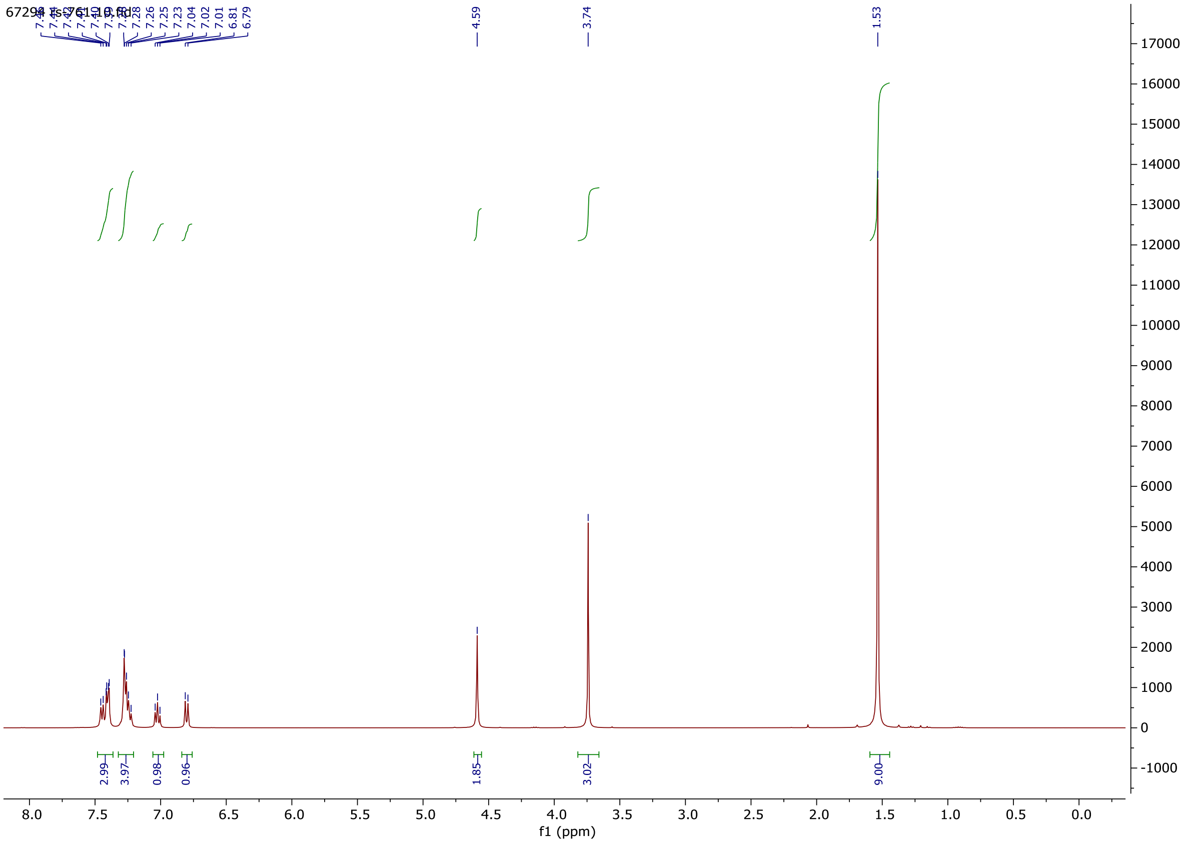

^{13}C NMR

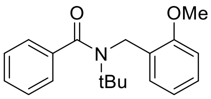

N-(tert-butyl)-*N*-(2-methoxybenzyl)benzamide (2b)

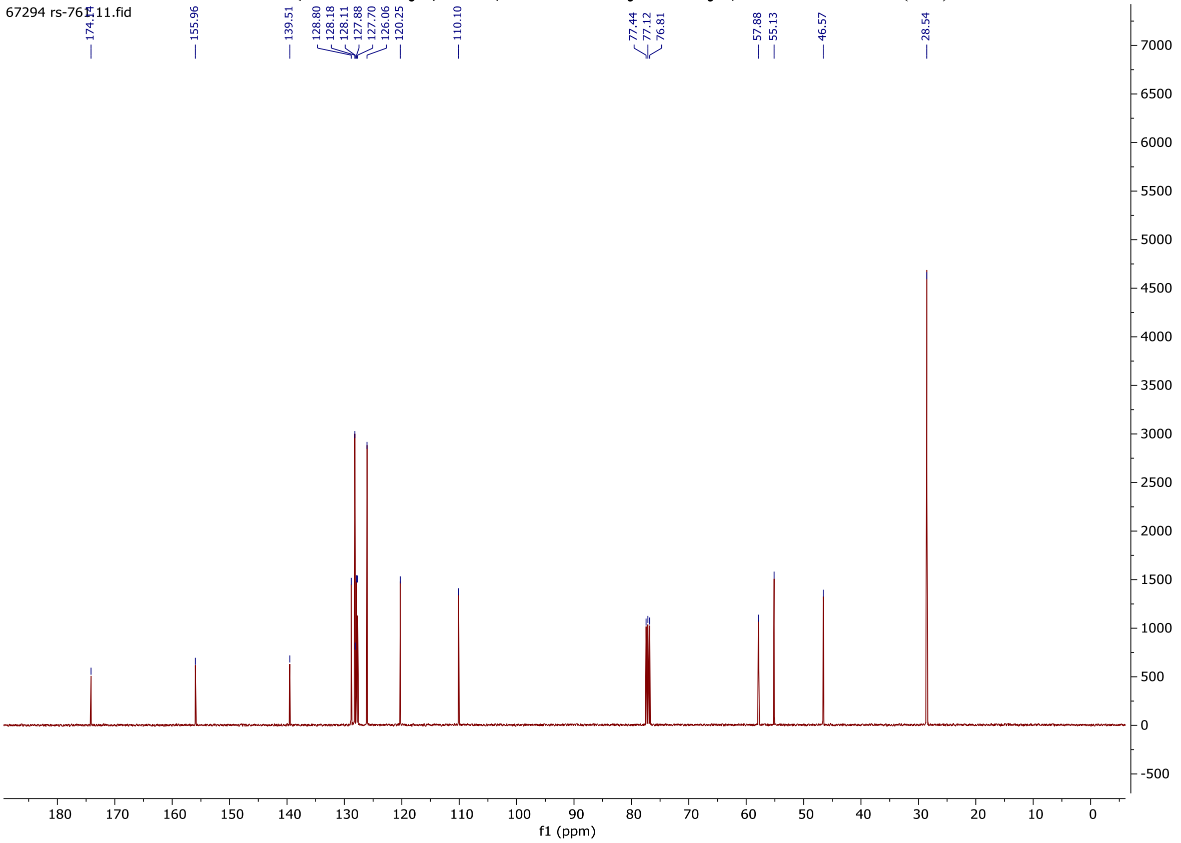

^{1}H NMR

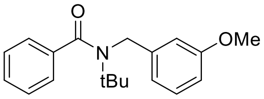

N-(tert-butyl)-*N*-(3-methoxybenzyl)benzamide (2c)

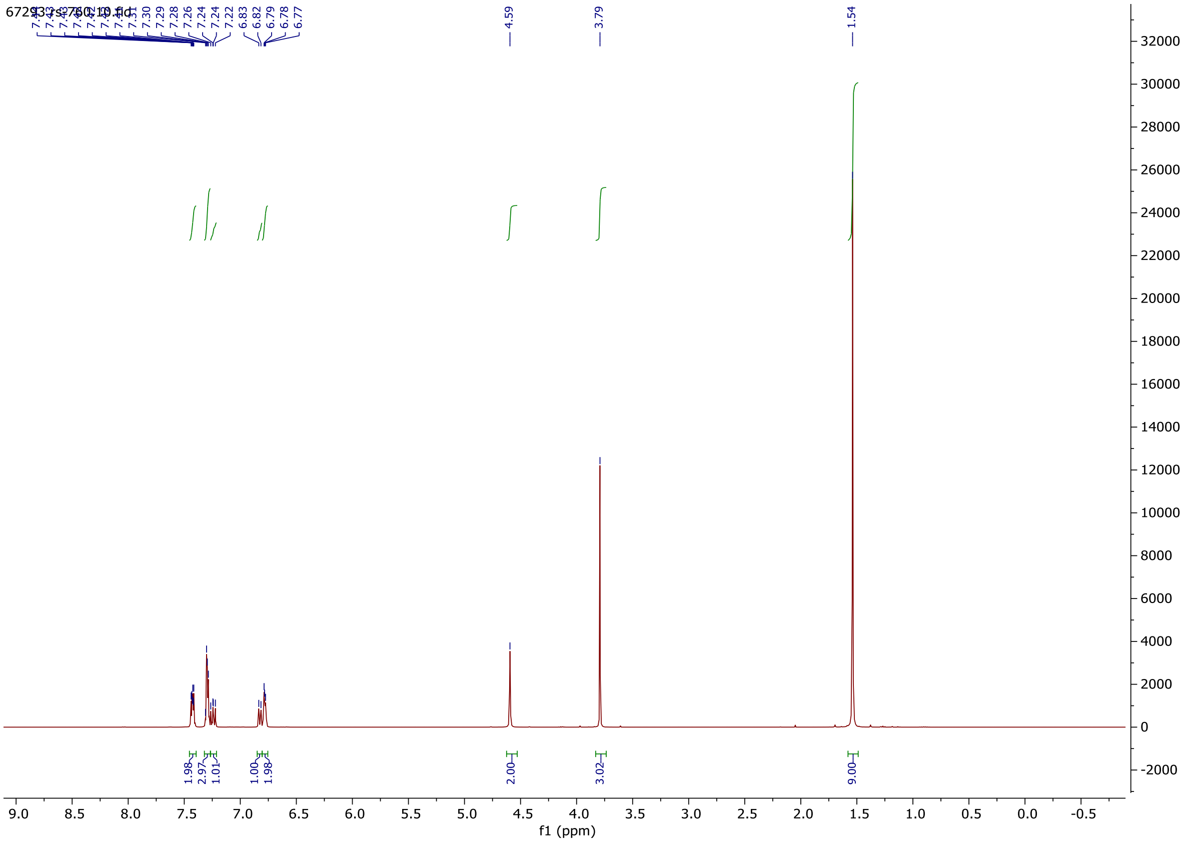

^{13}C NMR

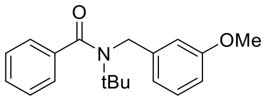

N-(tert-butyl)-N-(3-methoxybenzyl)benzamide (2c)

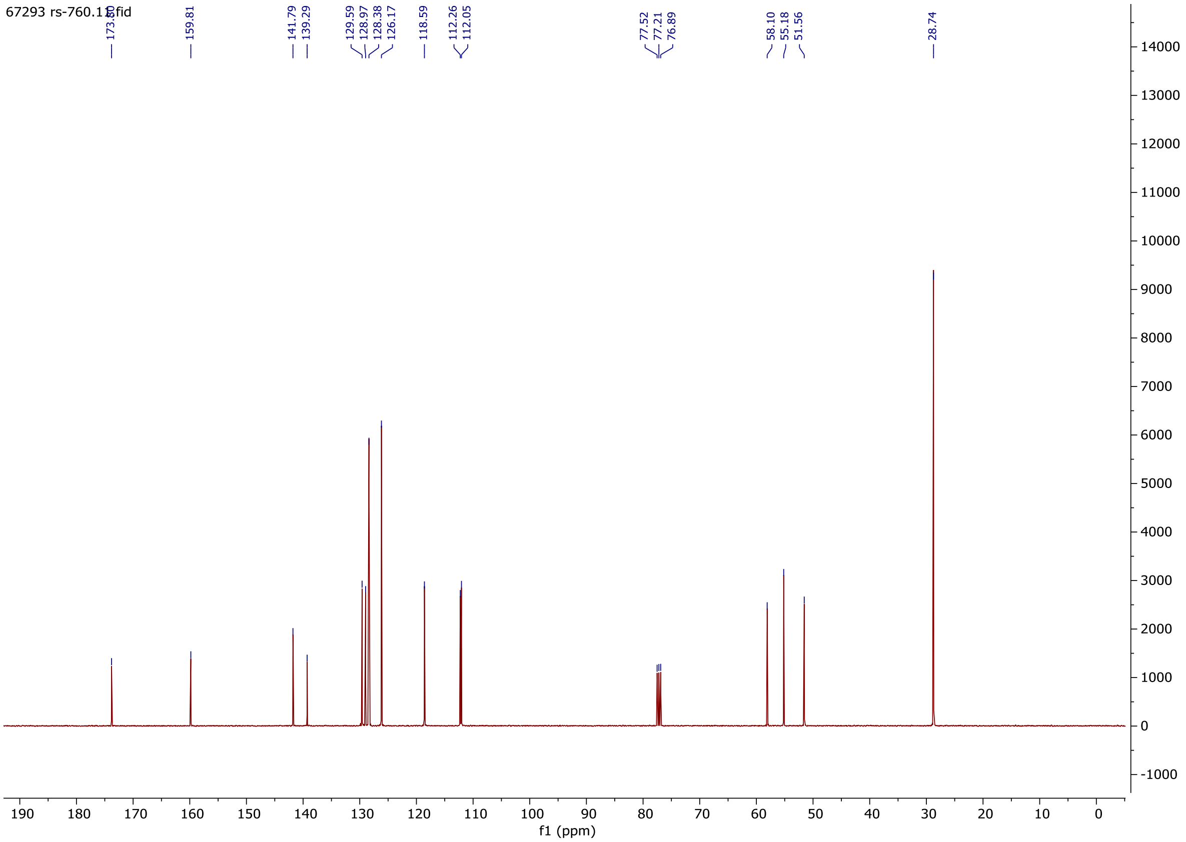

^{1}H NMR

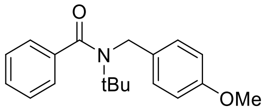

N-(tert-butyl)-N-(4-methoxybenzyl)benzamide (2d)

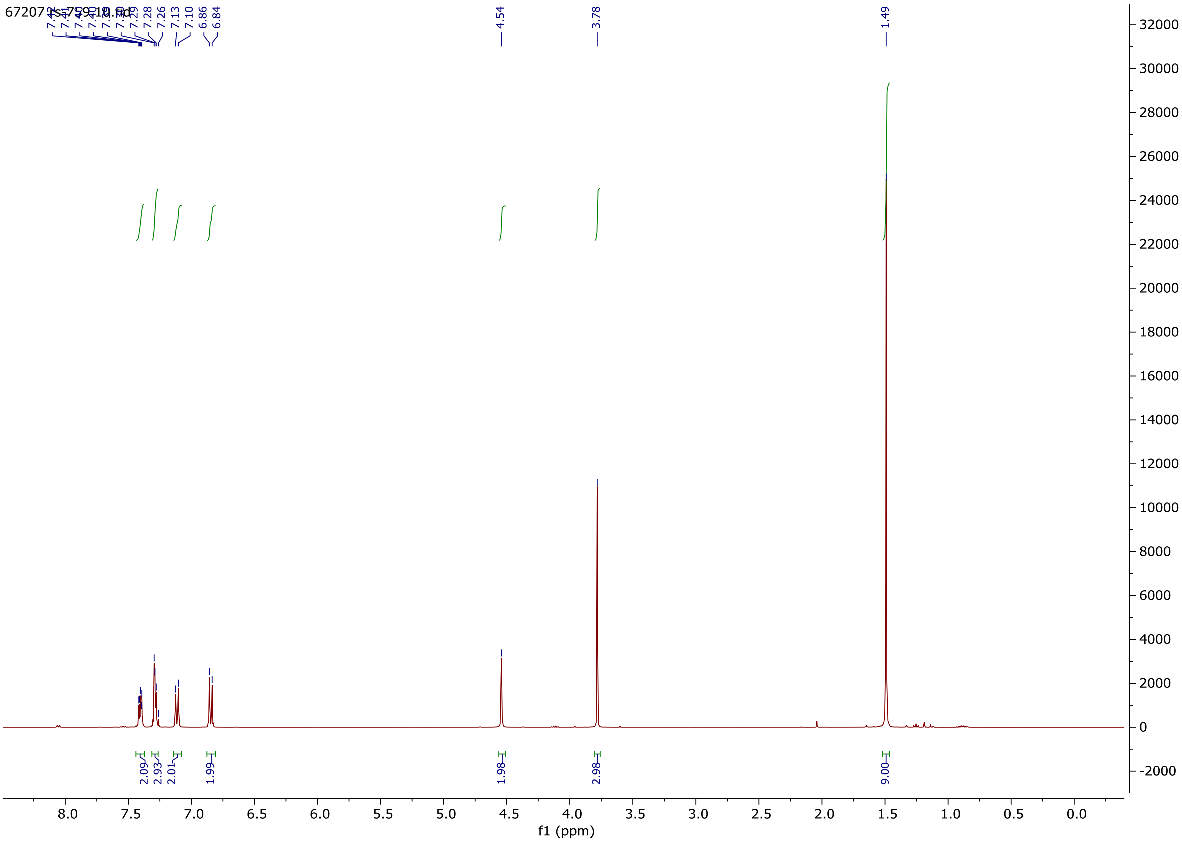

^{13}C NMR

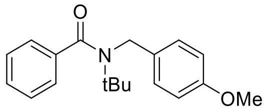

N-(*tert*-butyl)-N-(4-methoxybenzyl)benzamide (2d)

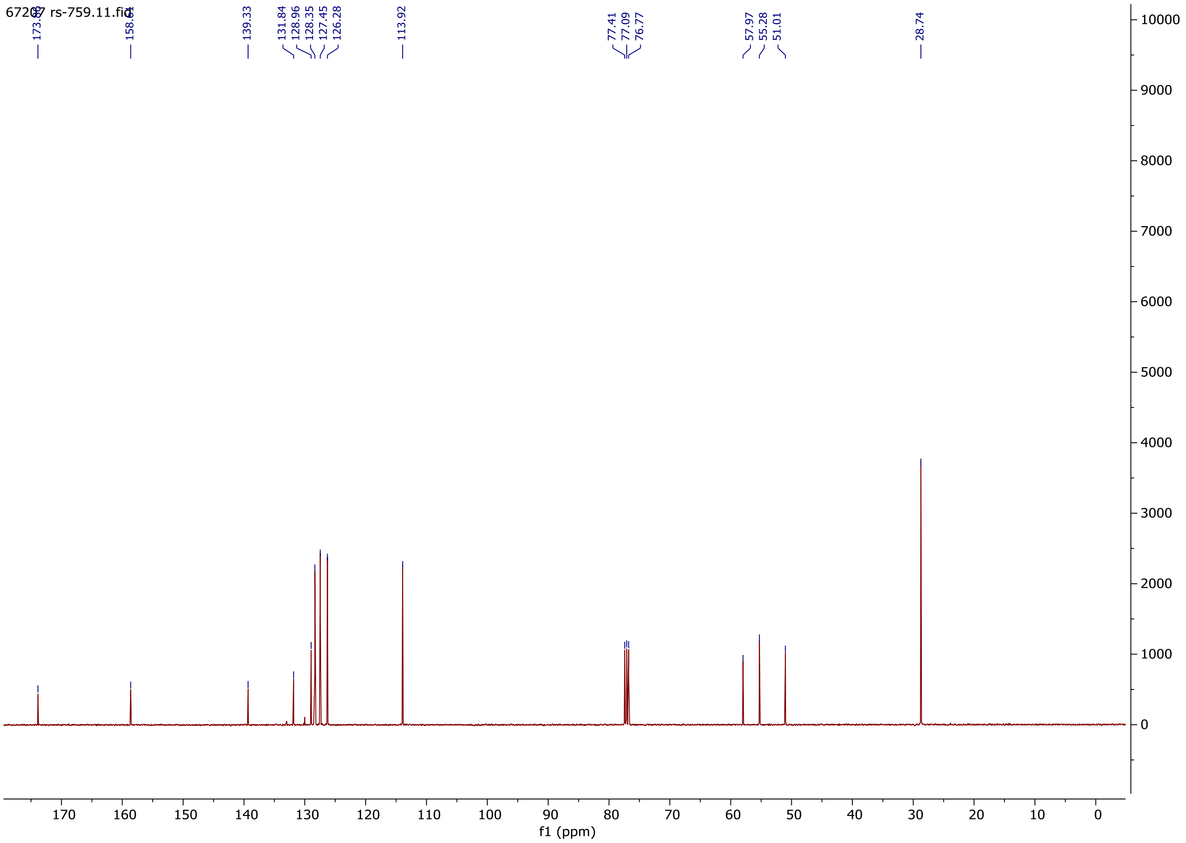

^{1}H NMR

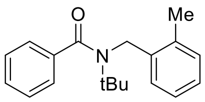

N-(*tert*-butyl)-*N*-(2-methylbenzyl)benzamide (2e)

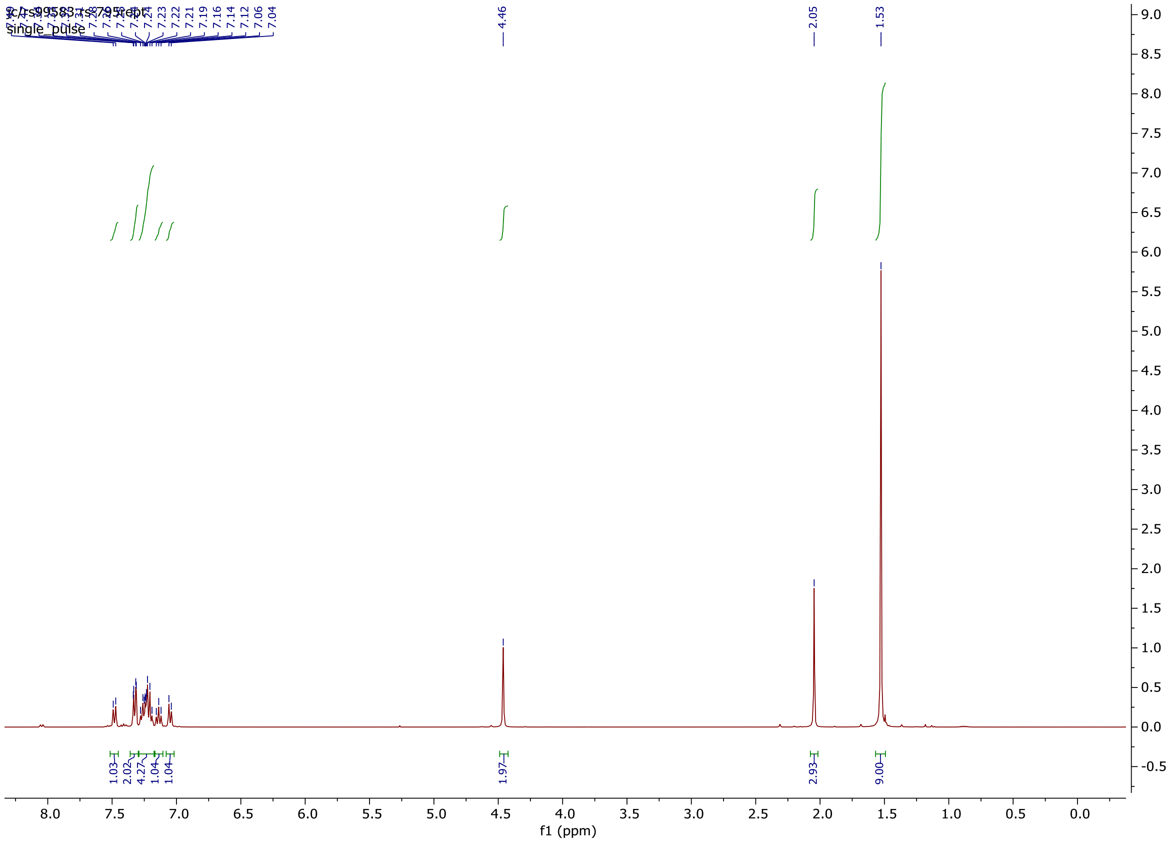

^{13}C NMR

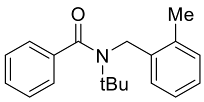

_N_-(_tert_-butyl)-_N_-(2-methylbenzyl)benzamide (2e)

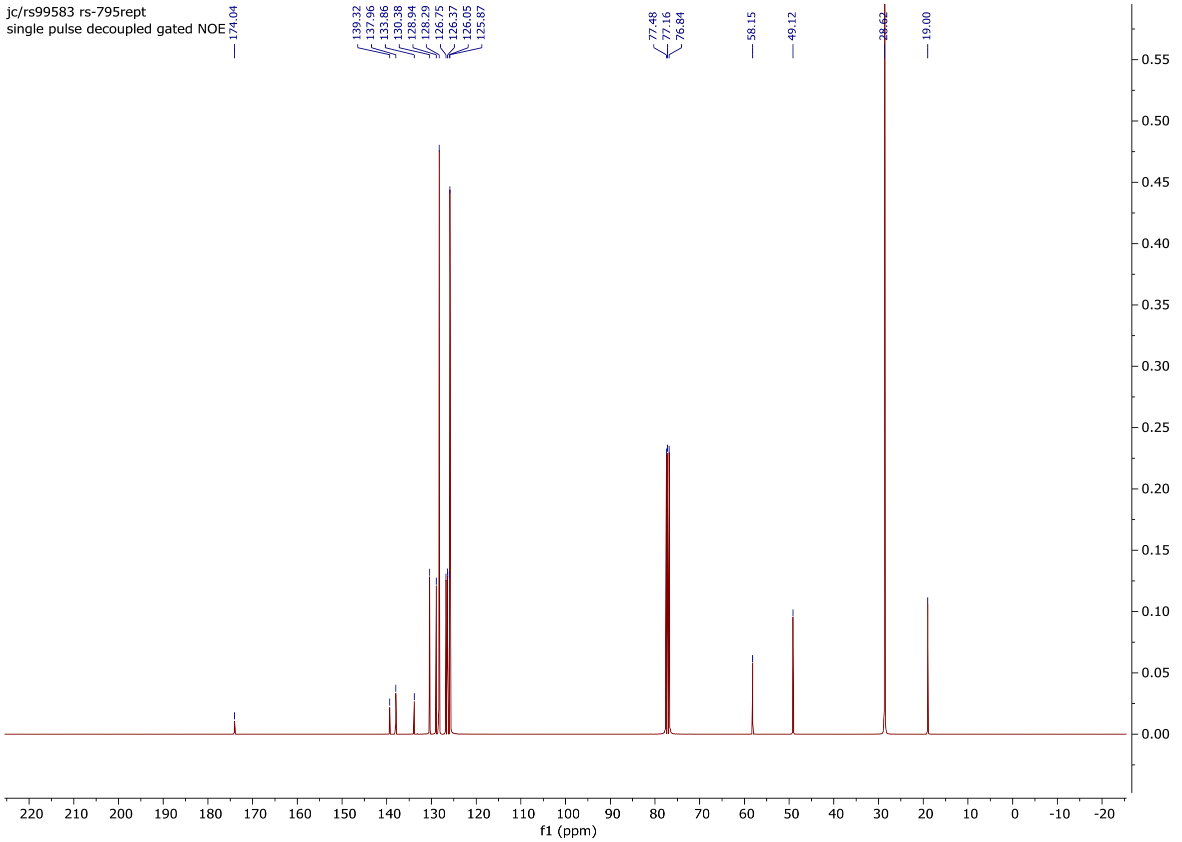

^{1}H NMR

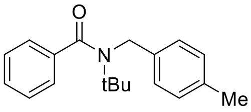

N-(tert-butyl)-N-(4-methylbenzyl)benzamide (2f)

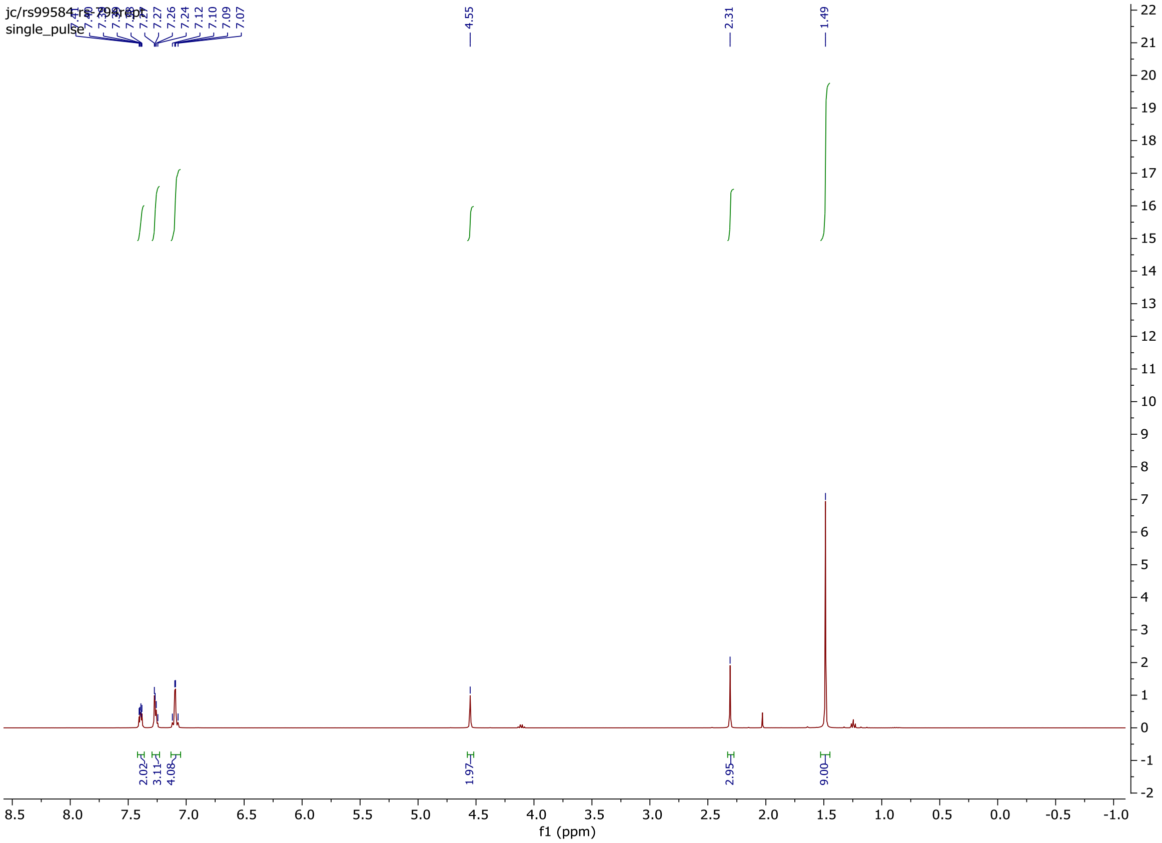

^{13}C NMR

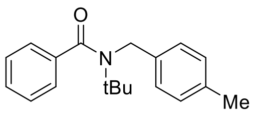

N-(tert-butyl)-*N*-(4-methylbenzyl)benzamide (2f)

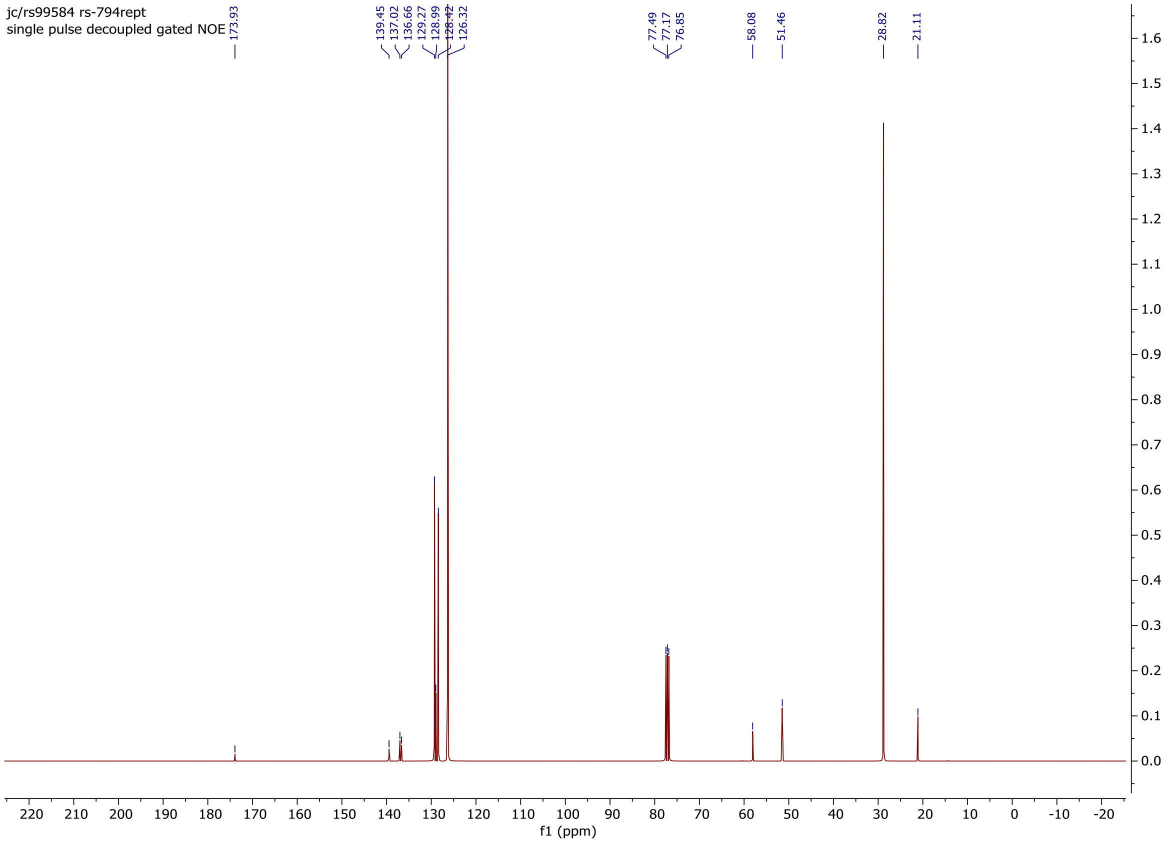

^{1}H NMR

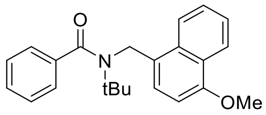

***N*-(tert-butyl)-*N*-((4-methoxynaphthalen-1-yl)methyl)benzamide (2g)**

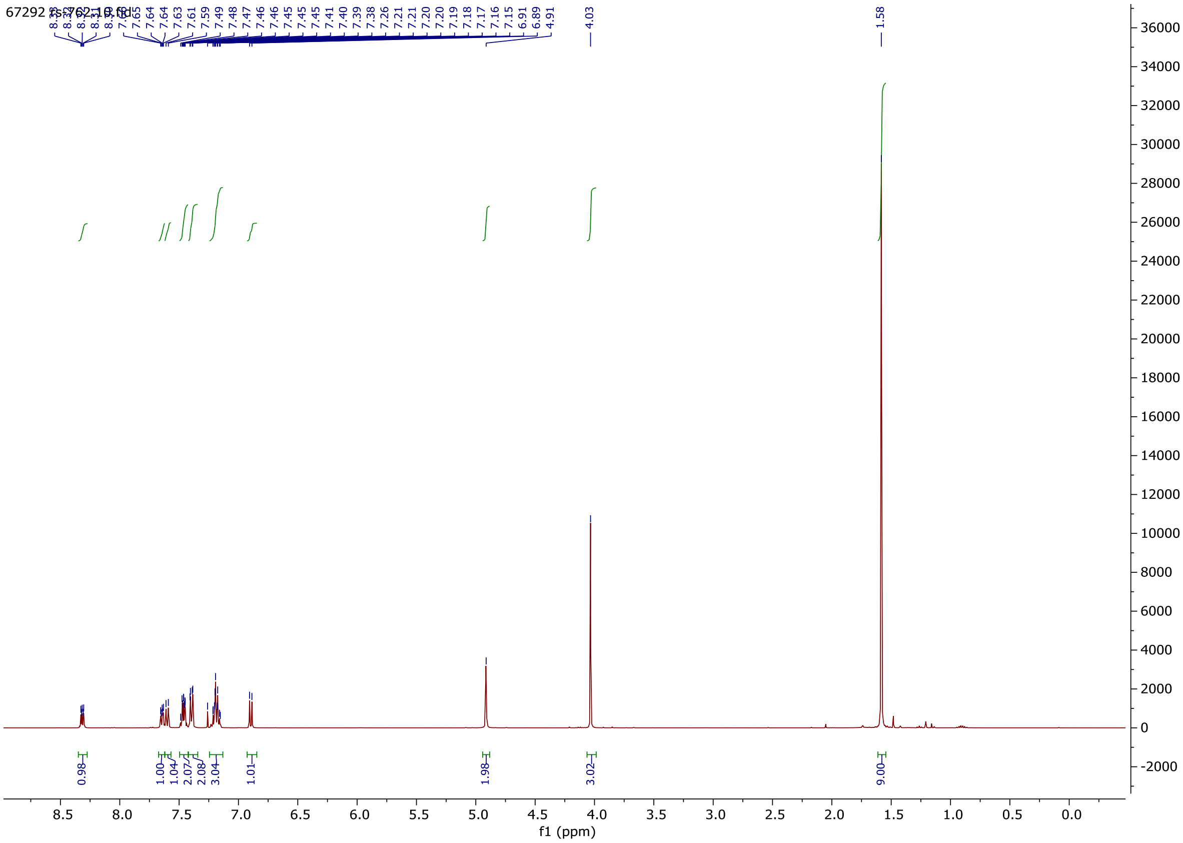

^{13}C NMR

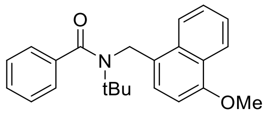

***N*-(tert-butyl)-*N*-((4-methoxynaphthalen-1-yl)methyl)benzamide (2g)**

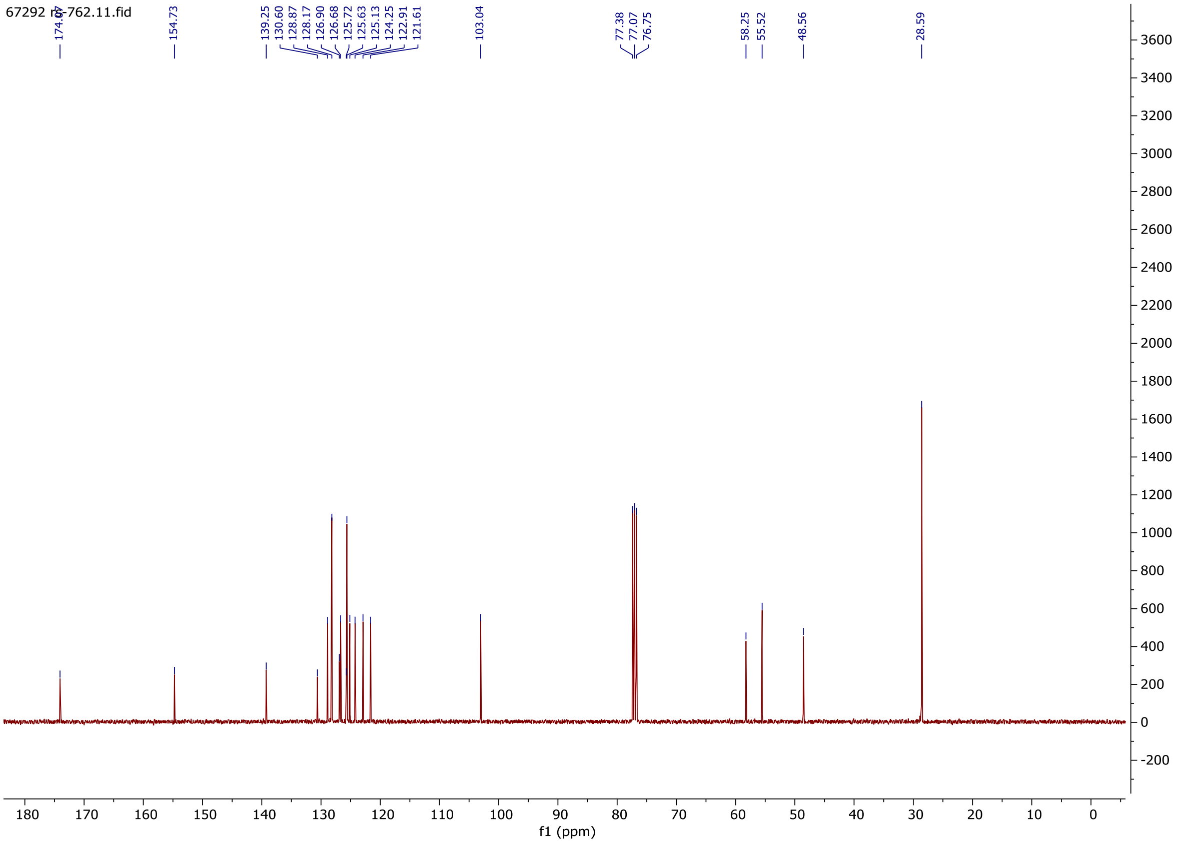

^{1}H NMR

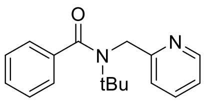

***N*-(tert-butyl)-*N*-(pyridin-2-ylmethyl)benzamide (2h)**

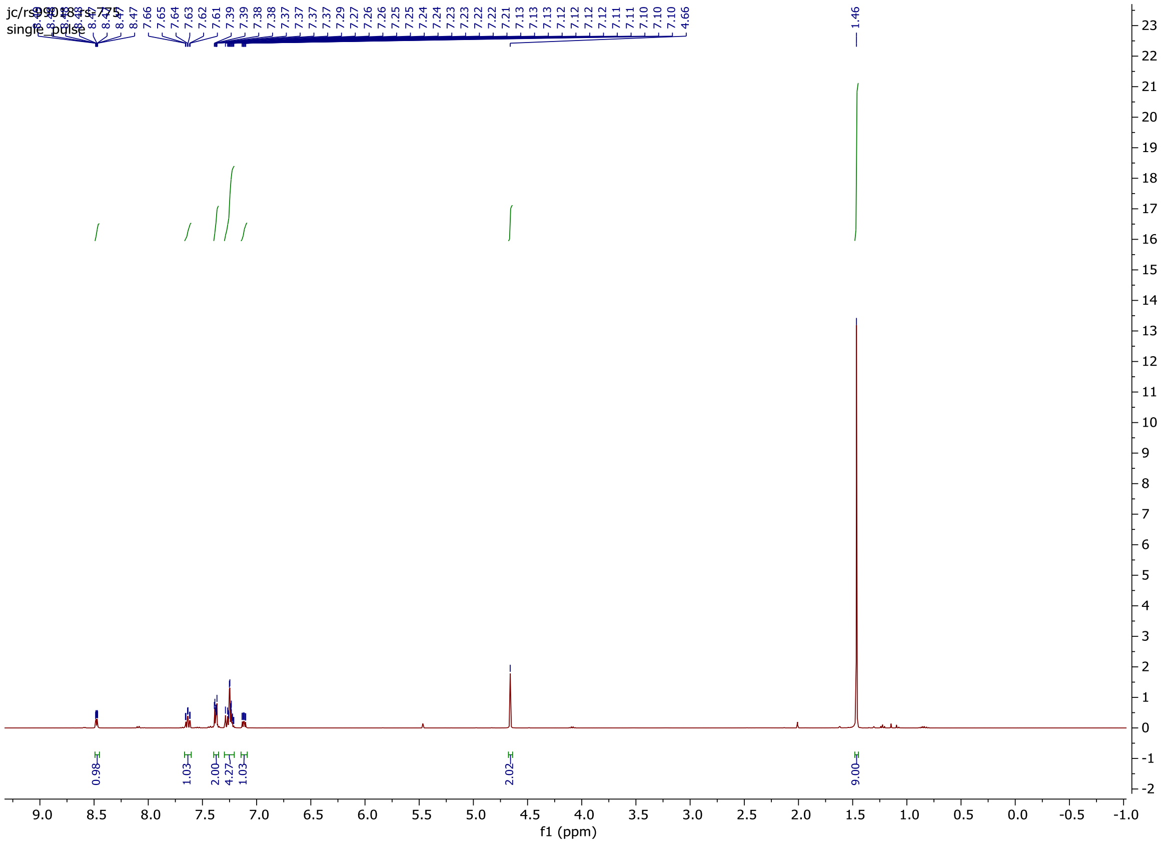

^{13}C NMR

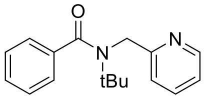

N-(tert-butyl)-*N*-(pyridin-2-ylmethyl)benzamide (2h)

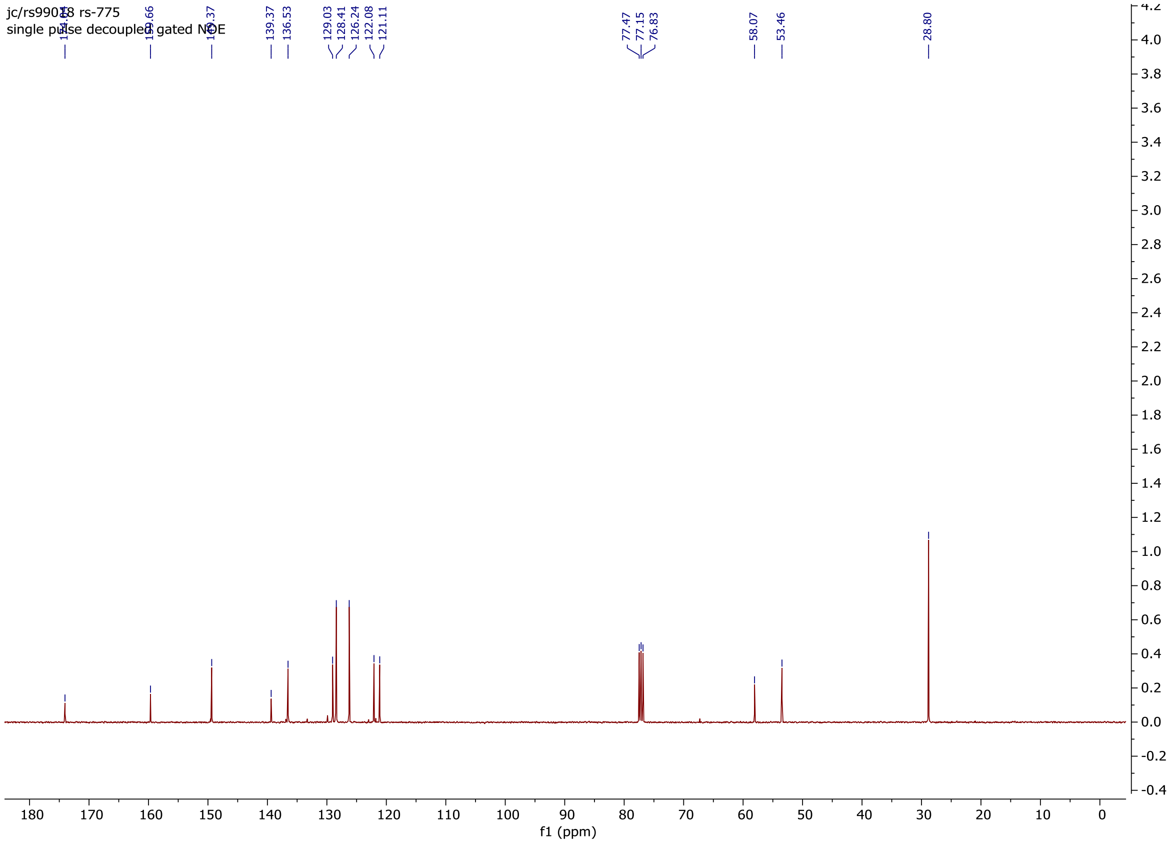

^1H NMR

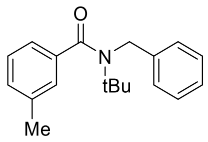

N-benzyl-*N*-(tert-butyl)-3-methylbenzamide (2i)

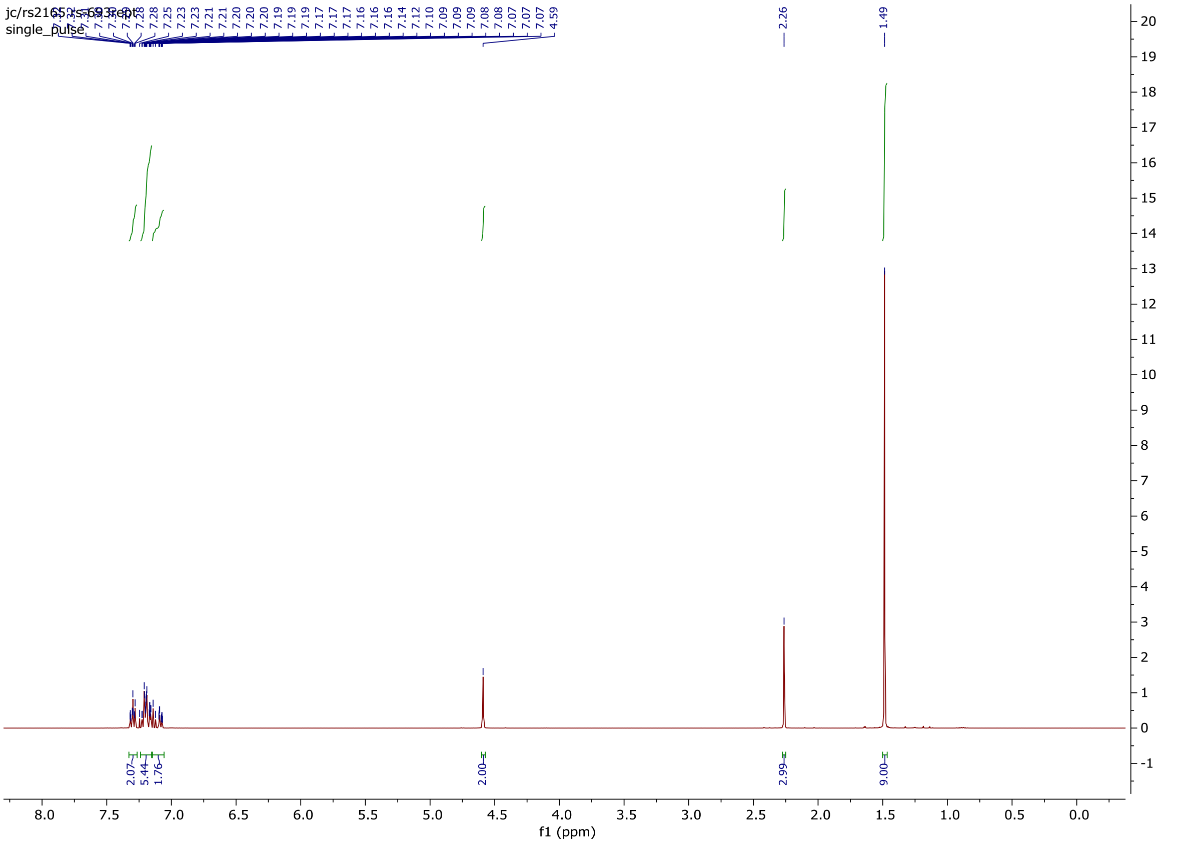

^{13}C NMR

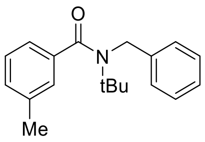

N-benzyl-*N*-(tert-butyl)-3-methylbenzamide (2i)

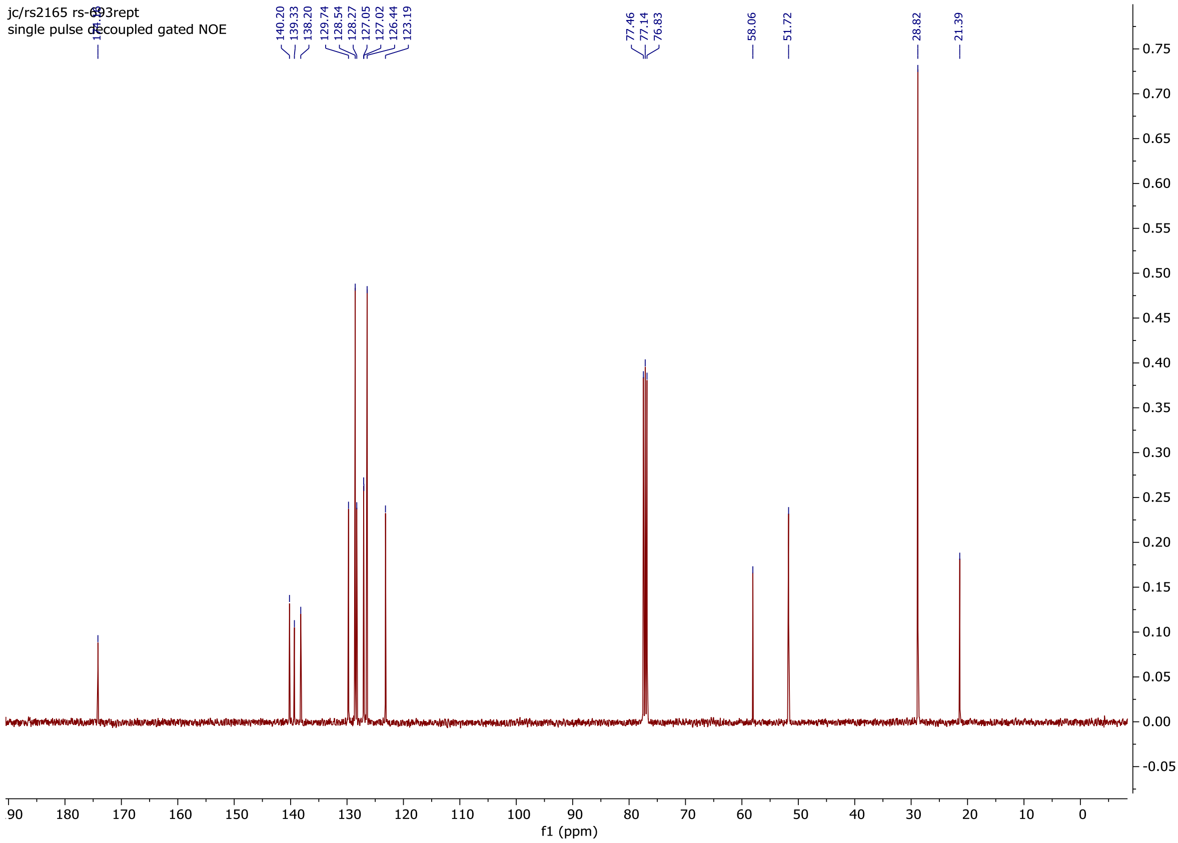

^{1}H NMR

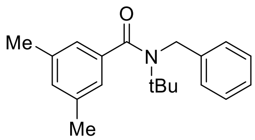

***N*-benzyl-*N*-(tert-butyl)-3,5-dimethylbenzamide (2j)**

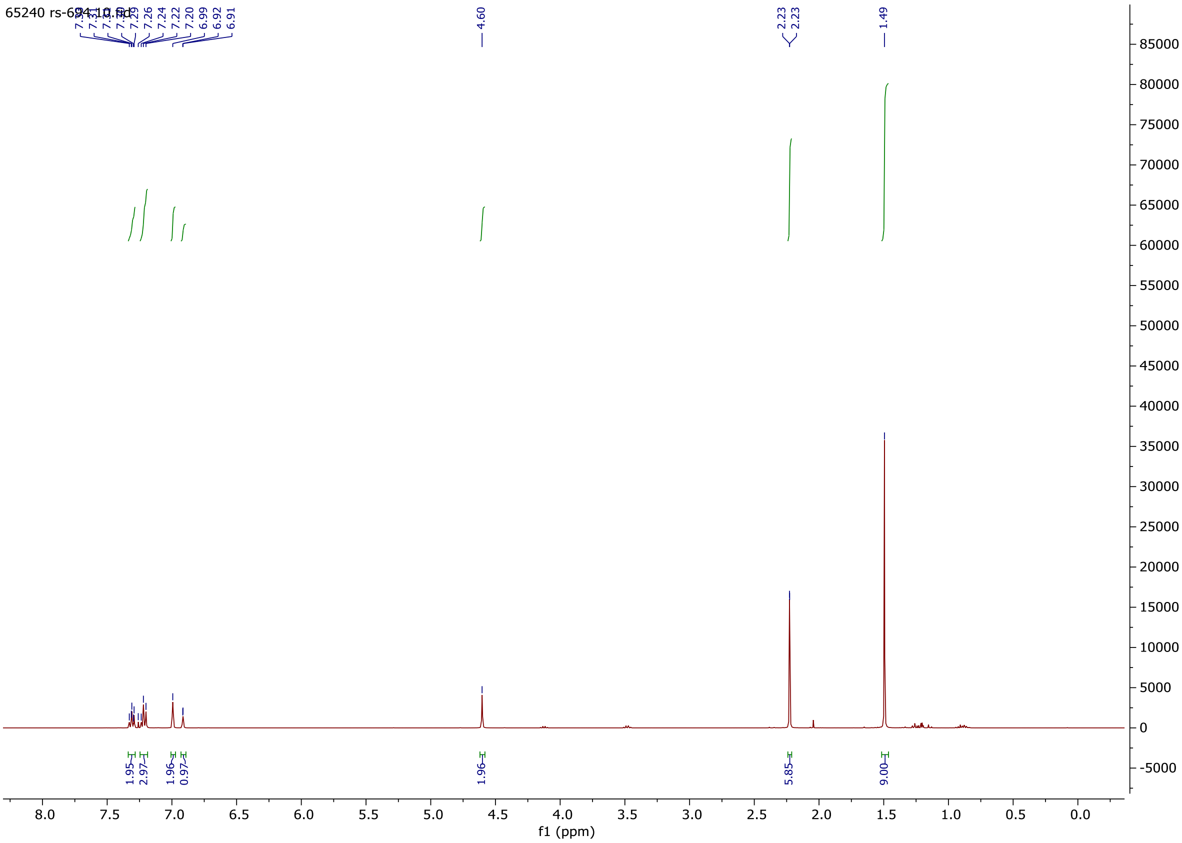

^{13}C NMR

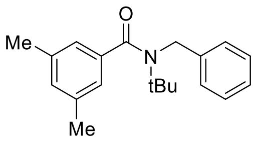

N-benzyl-*N*-(tert-butyl)-3,5-dimethylbenzamide (2j)

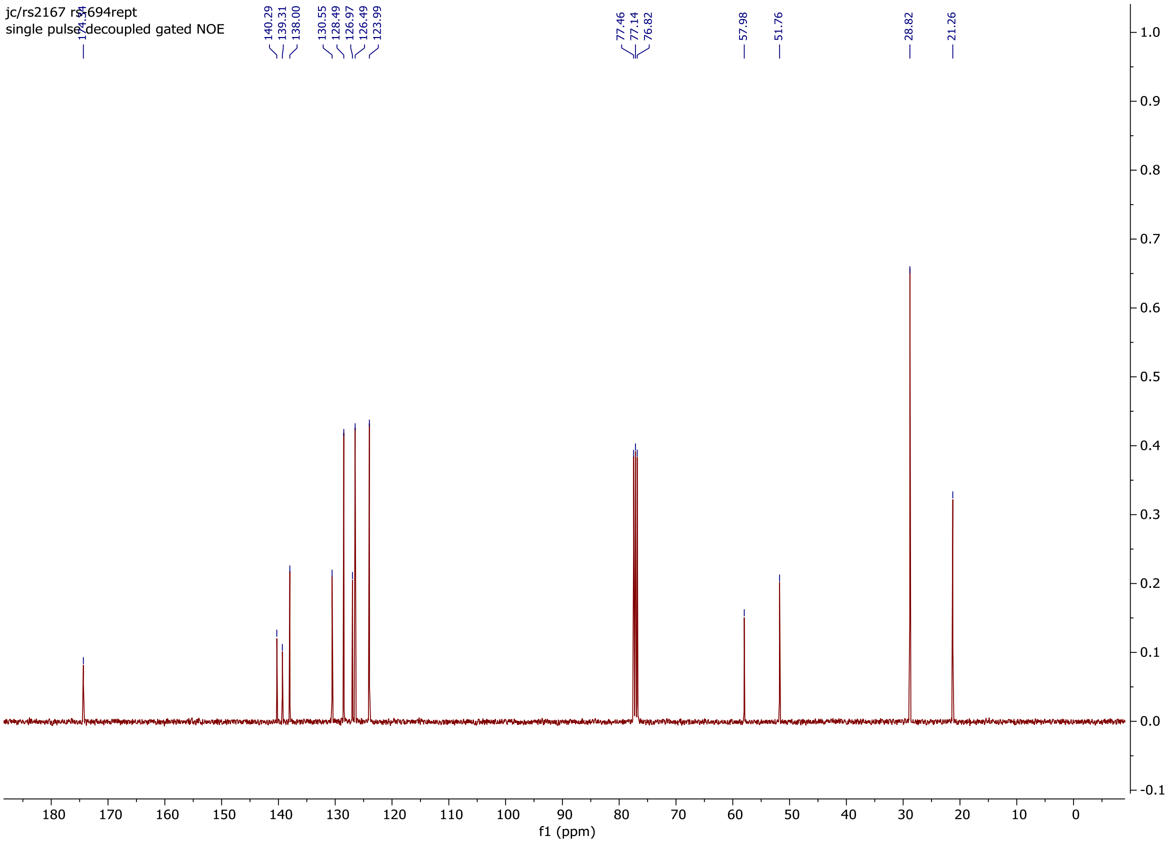

^{1}H NMR

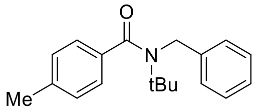

***N*-benzyl-*N*-(tert-butyl)-4-methylbenzamide (2k)**

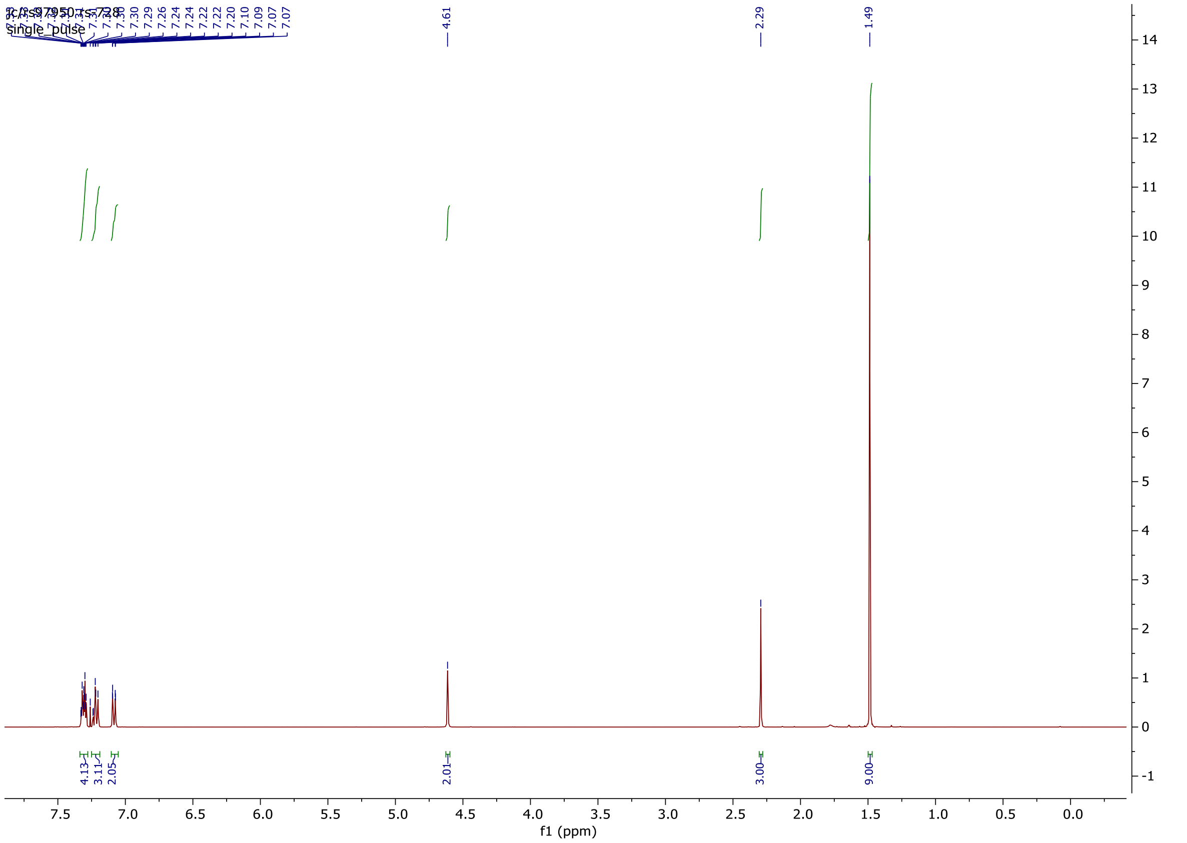

^{13}C NMR

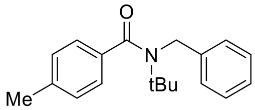

N-benzyl-*N*-(tert-butyl)-4-methylbenzamide (2k)

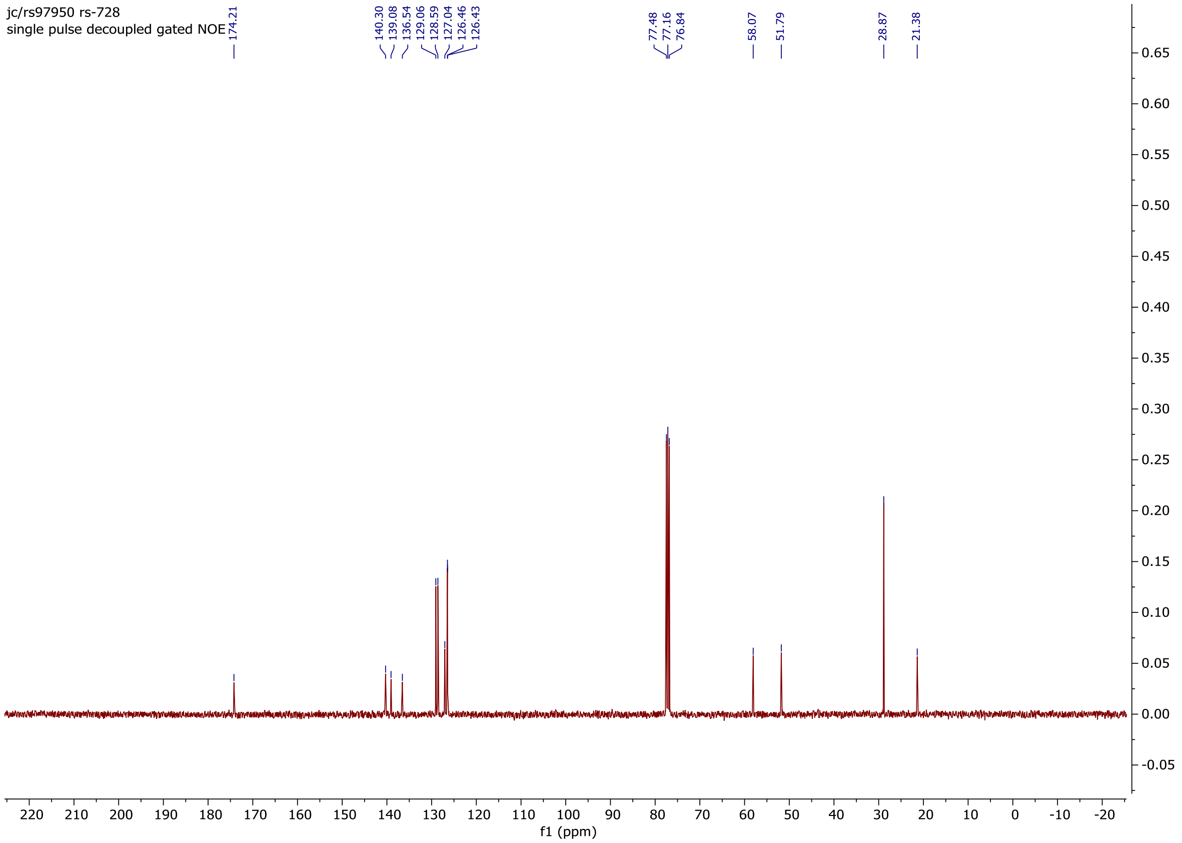

^{1}H NMR

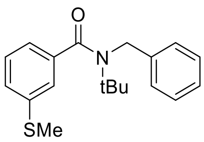

***N*-benzyl-*N*-(*tert*-butyl)-3-(methylthio)benzamide (2l)**

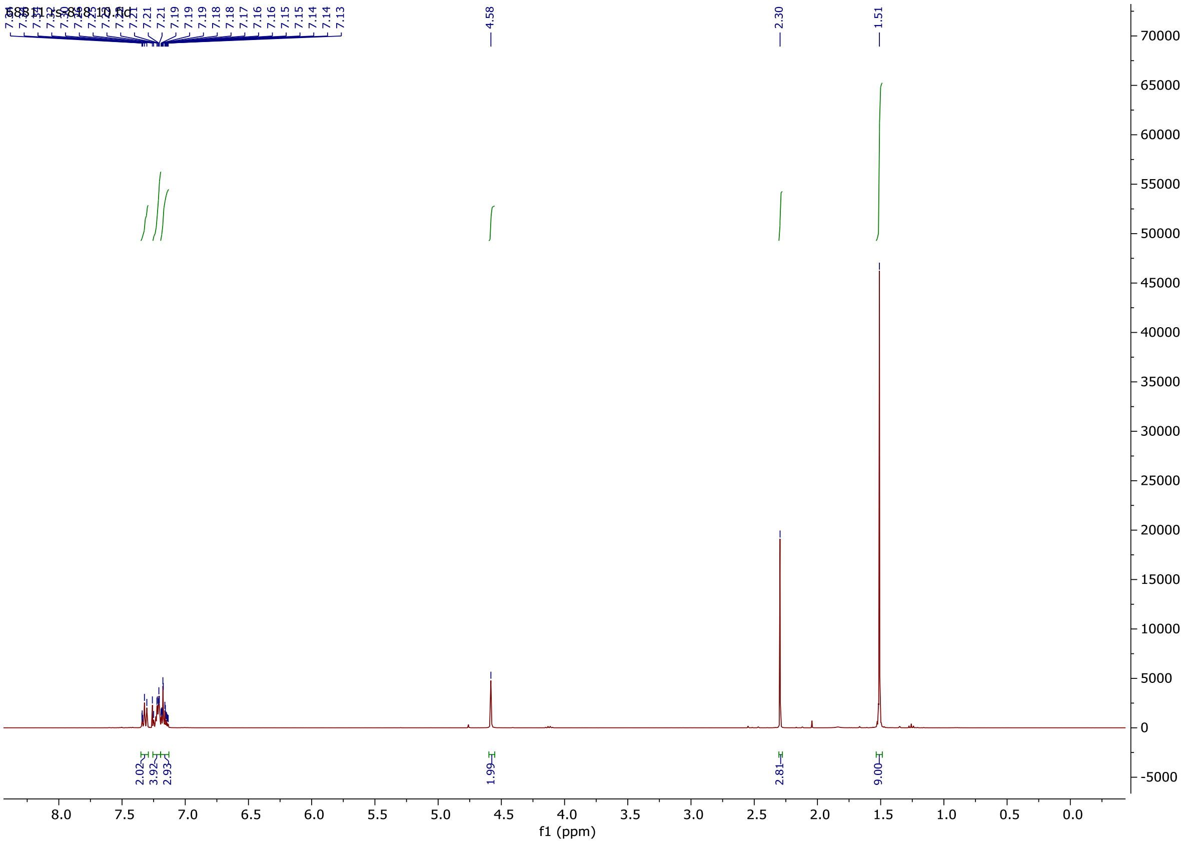

^{13}C NMR

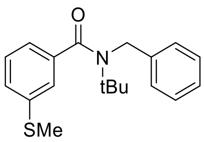

_N_-benzyl-_N_-(_tert_-butyl)-3-(methylthio)benzamide (2l)

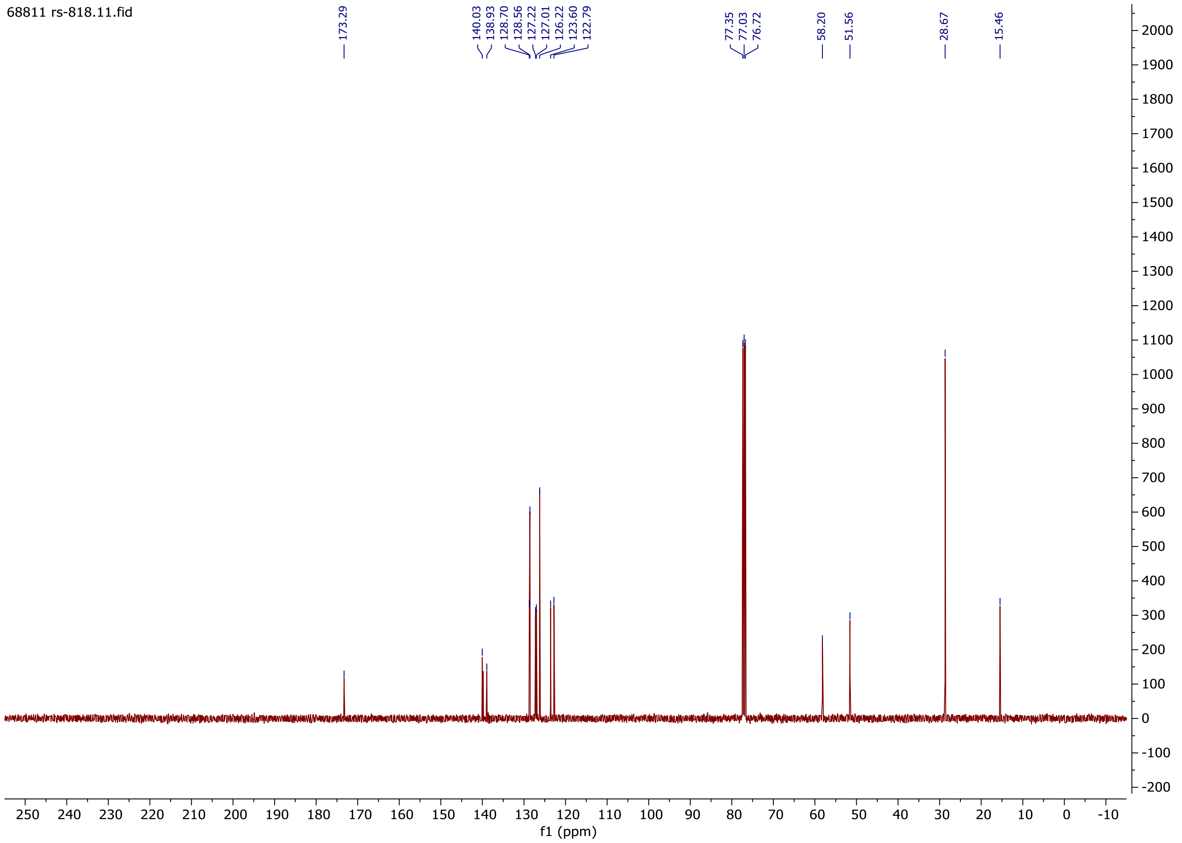

^{1}H NMR

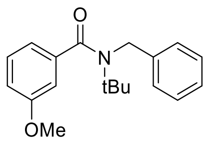

***N*-benzyl-*N*-(tert-butyl)-3-methoxybenzamide (2m)**

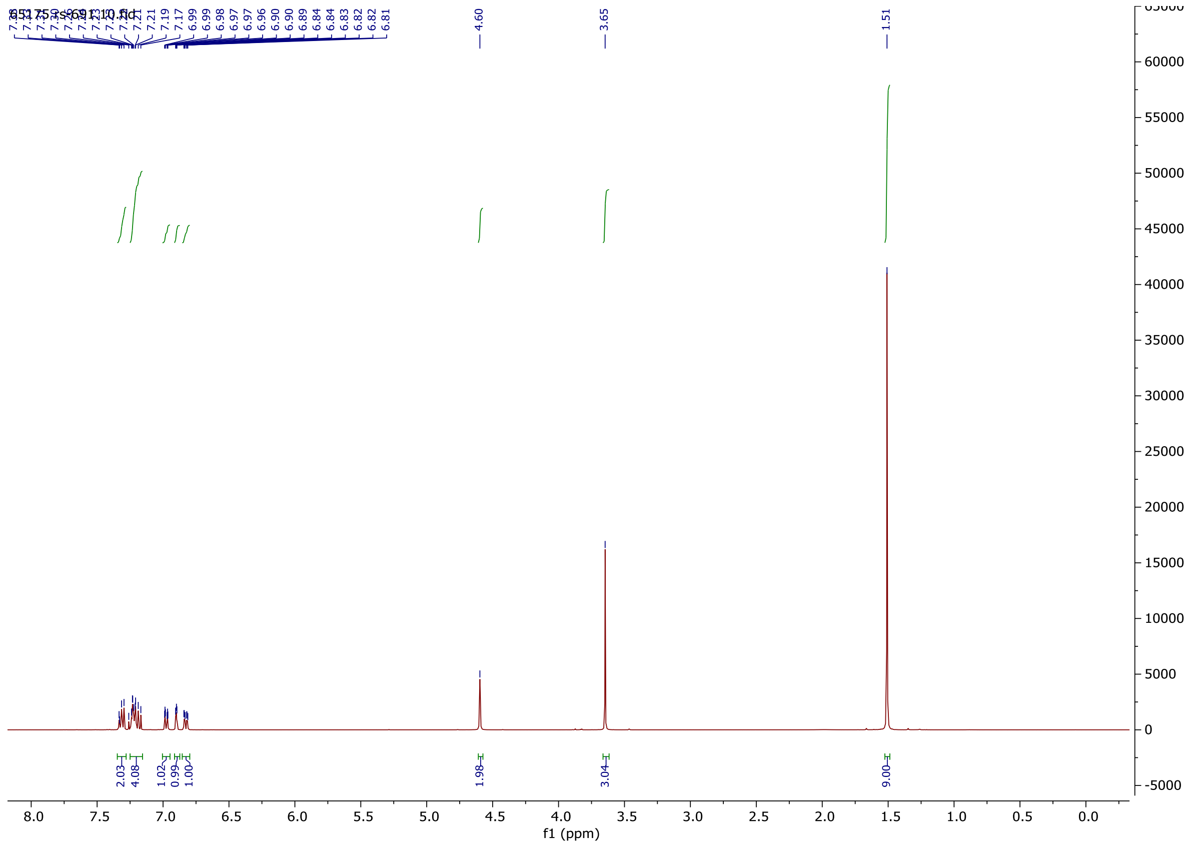

^{13}C NMR

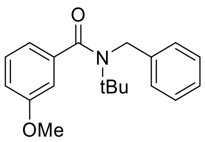

N-benzyl-*N*-(tert-butyl)-3-methoxybenzamide (2m)

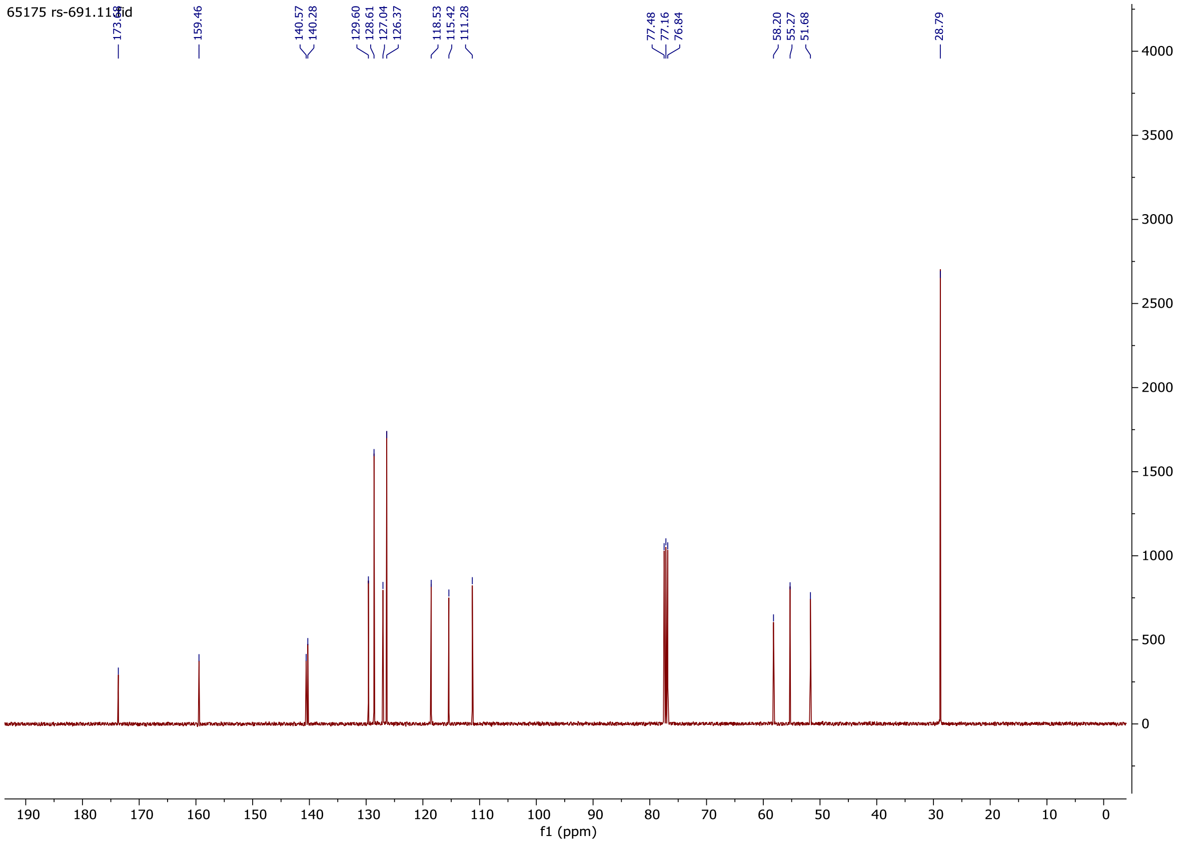

^{1}H NMR

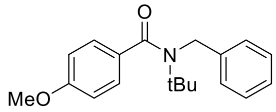

***N*-benzyl-*N*-(tert-butyl)-4-methoxybenzamide (2n)**

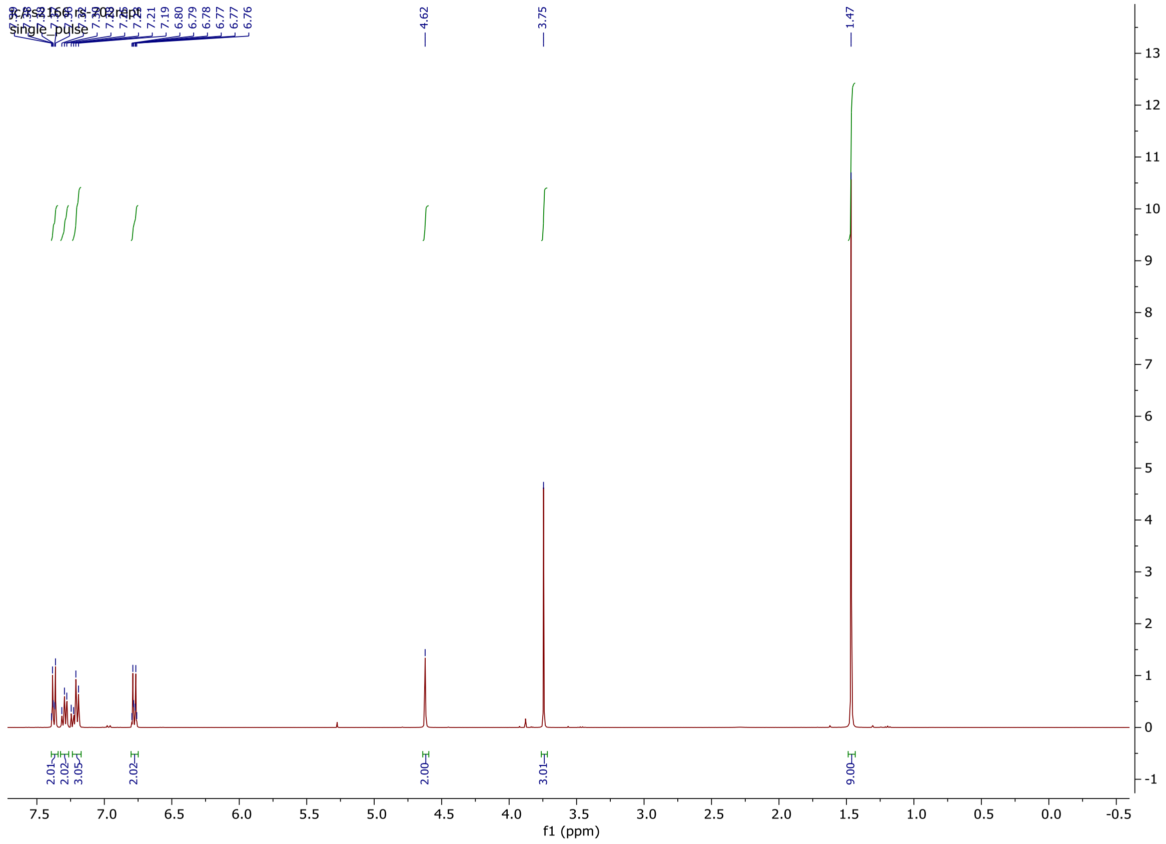

^{13}C NMR

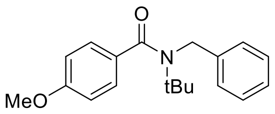

***N*-benzyl-*N*-(tert-butyl)-4-methoxybenzamide (2n)**

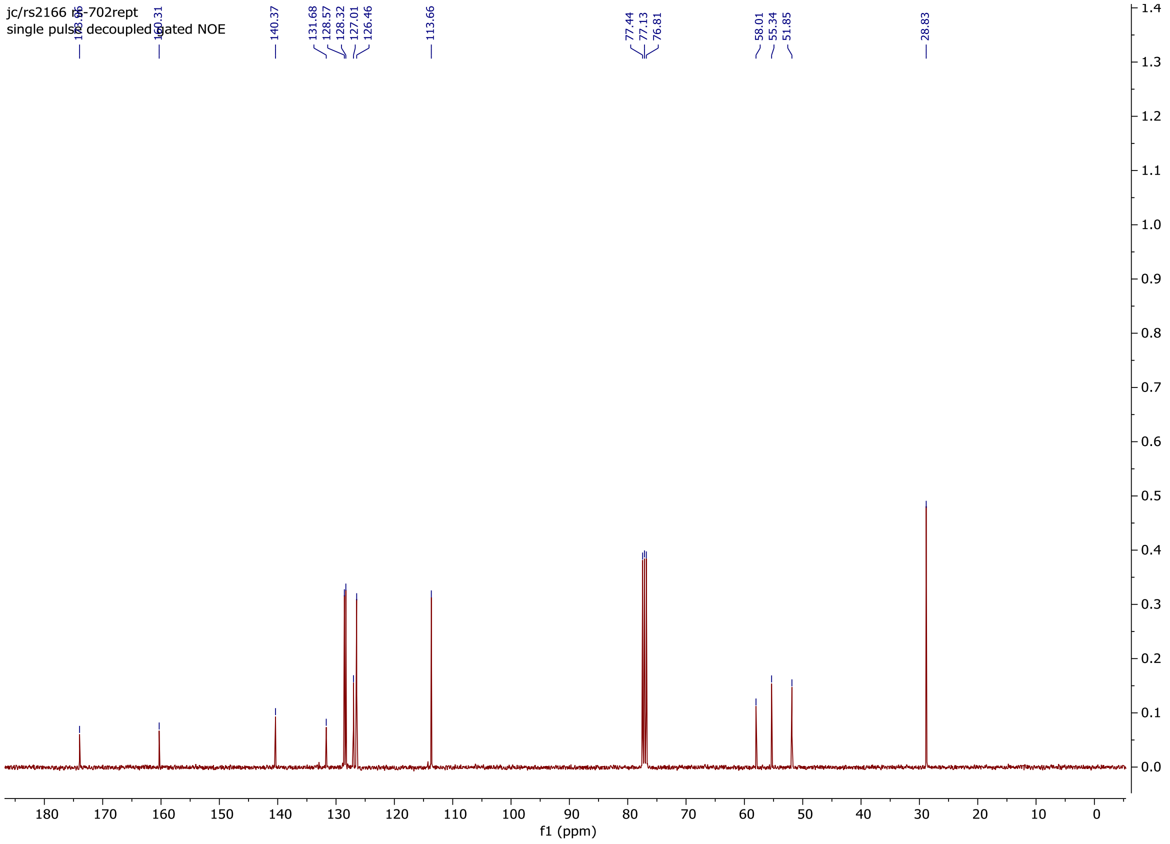

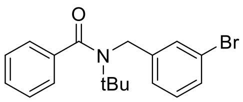

N-(3-bromobenzyl)-*N*-(tert-butyl)benzamide (2o)

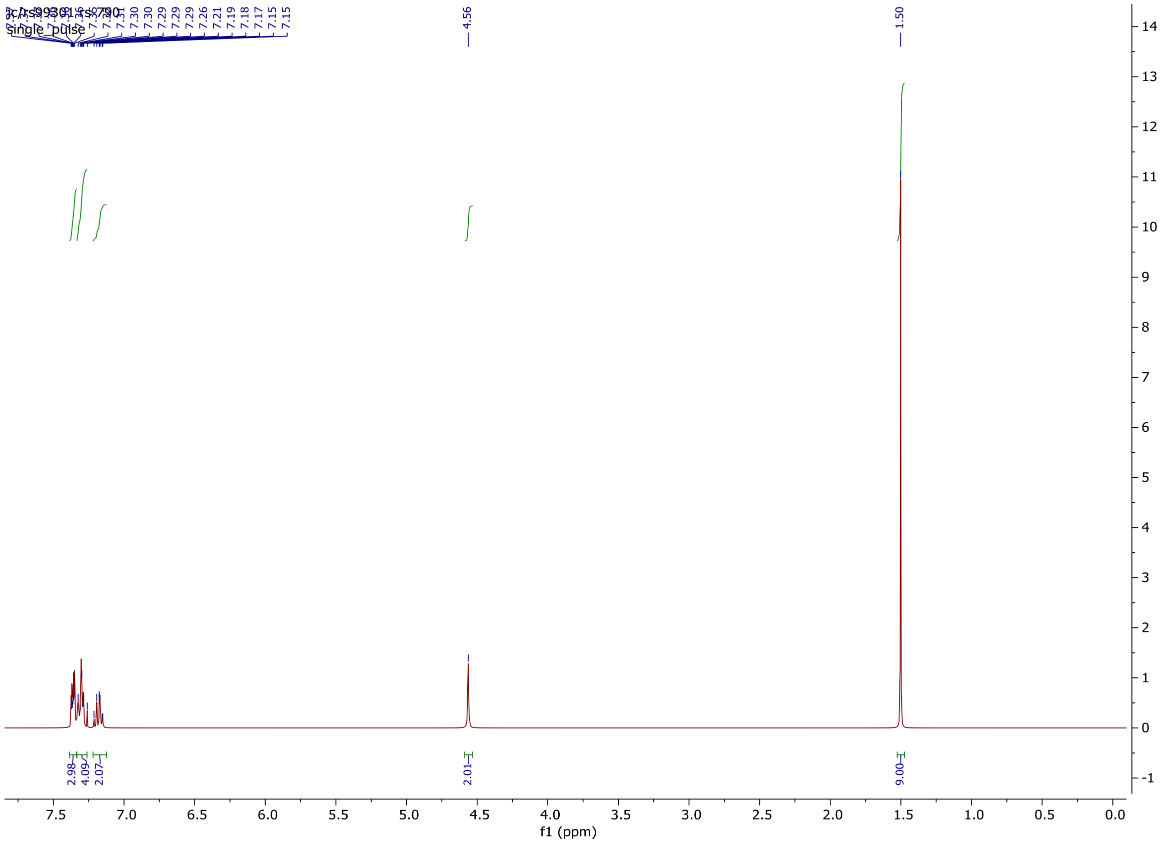

^{13}C NMR

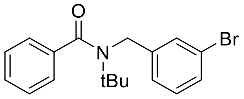

N-(3-bromobenzyl)-*N*-(tert-butyl)benzamide (2o)

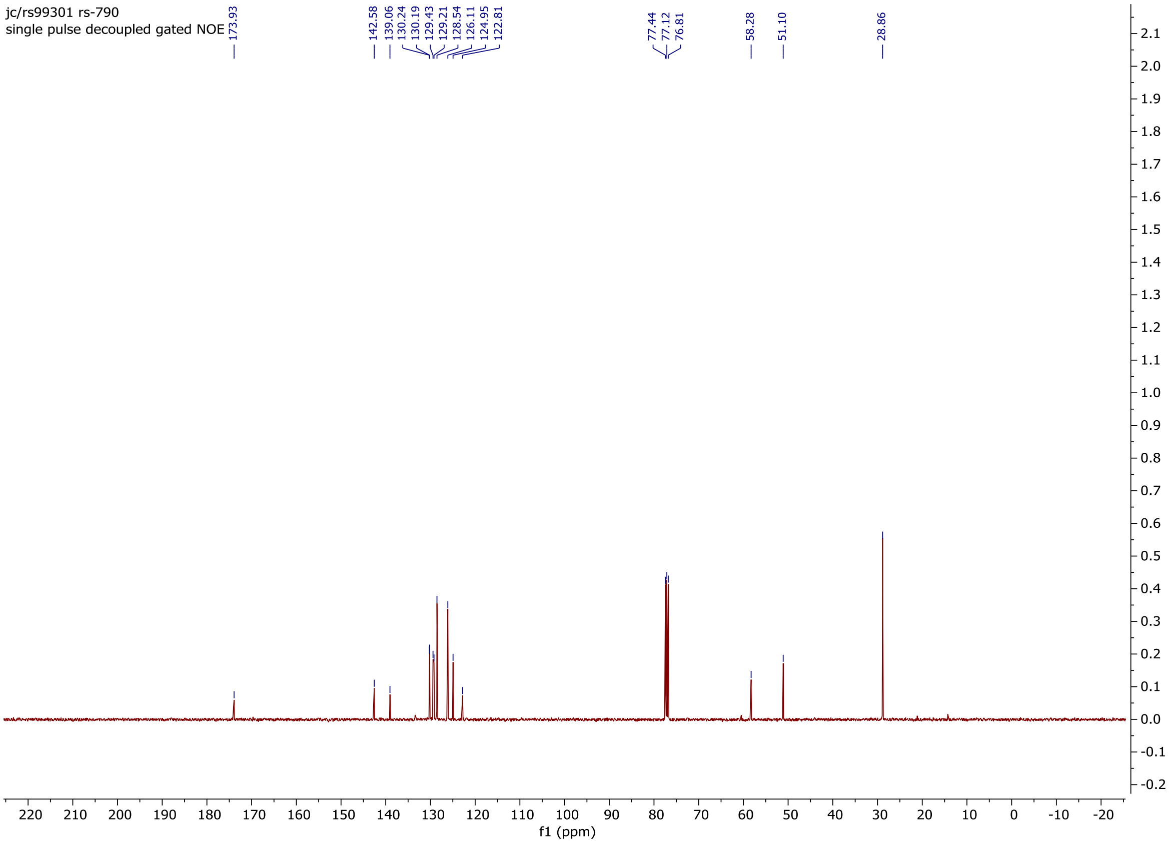

^{1}H NMR

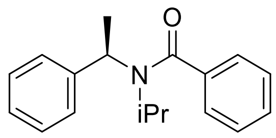

(*R*)-*N*-isopropyl-*N*-(1-phenylethyl)benzamide (10a)

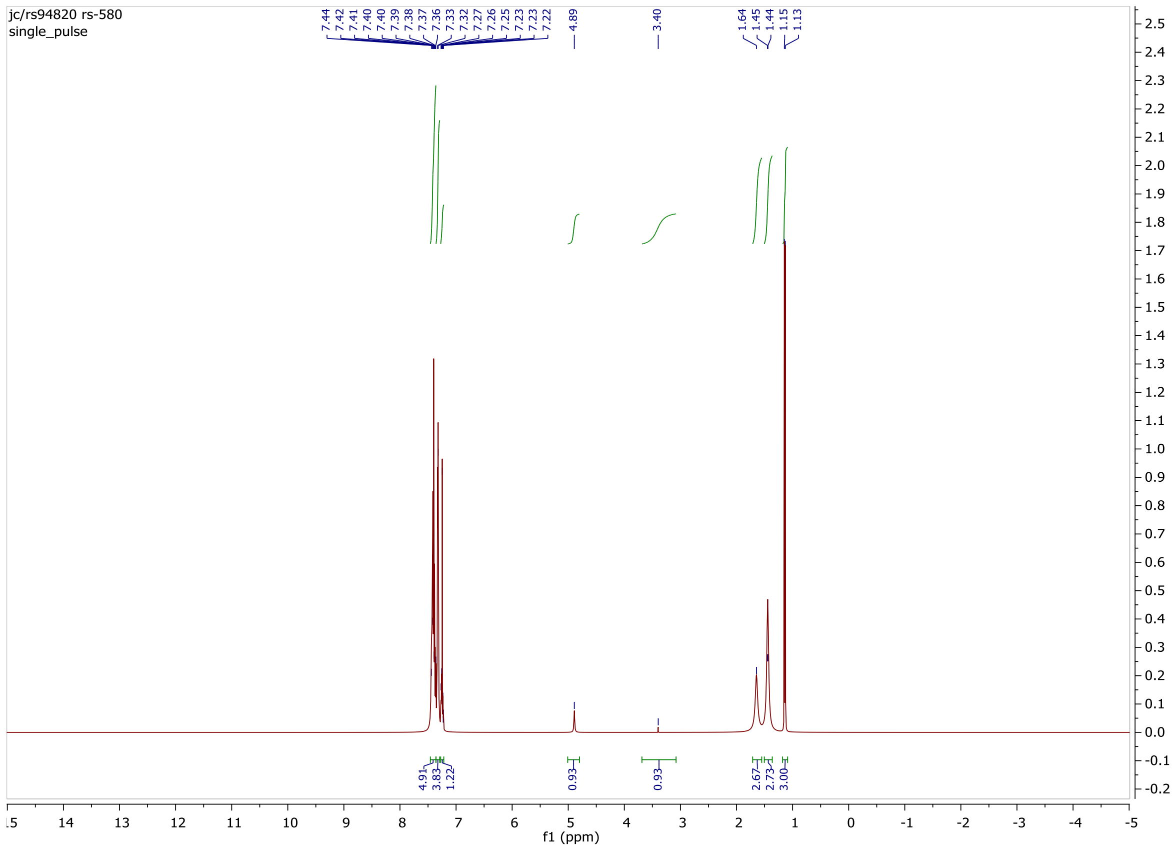

^{13}C NMR

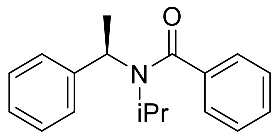

***(R)*-*N*-isopropyl-*N*-(1-phenylethyl)benzamide (10a)**

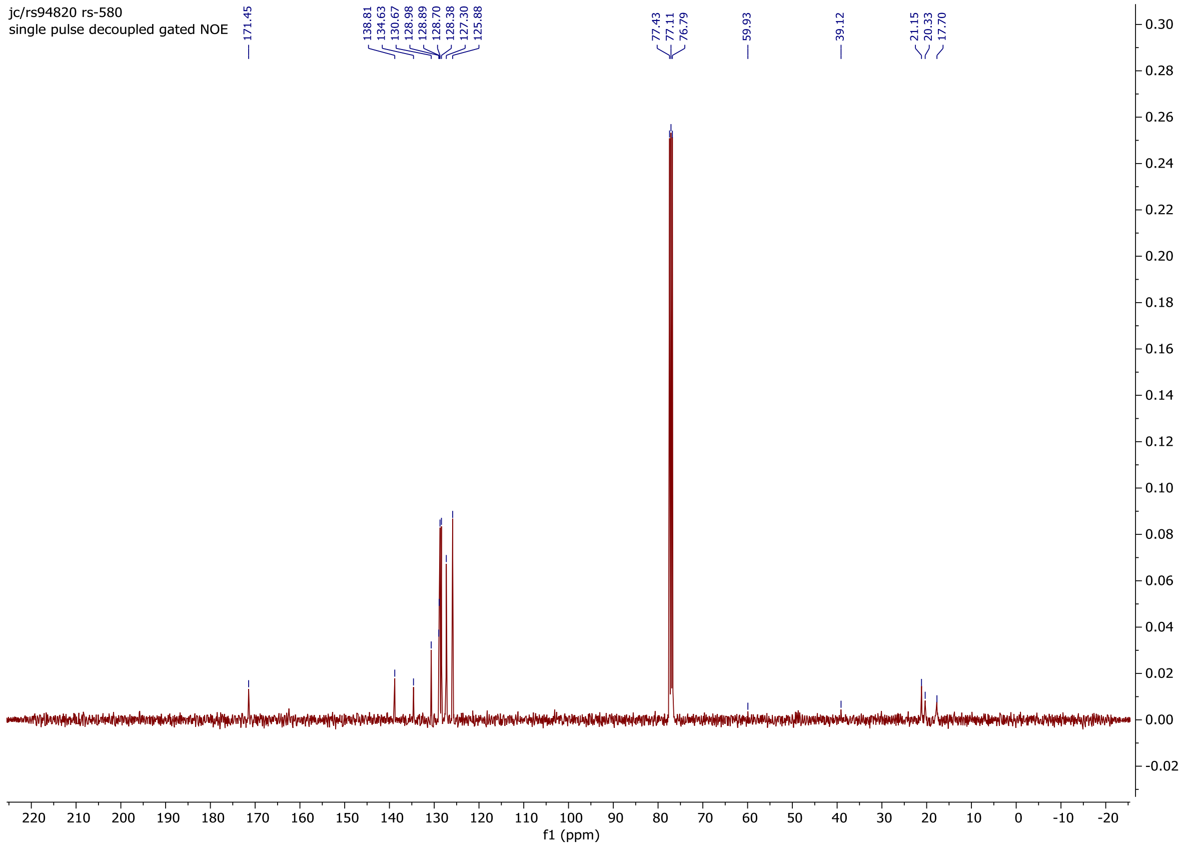

^{1}H NMR

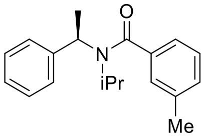

***(R)*-*N*-isopropyl-3-methyl-*N*-(1-phenylethyl)benzamide (10b)**

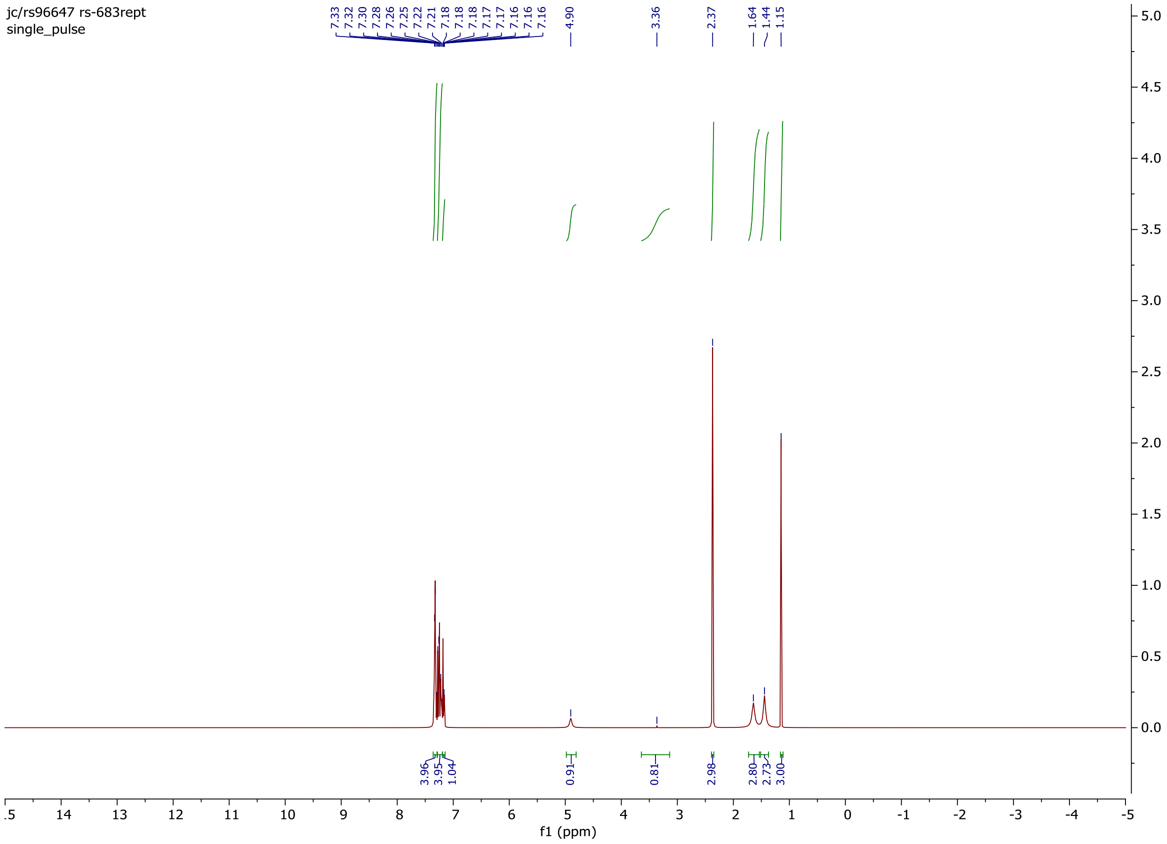

^{13}C NMR

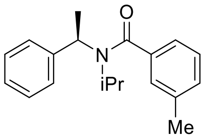

(R)-*N*-isopropyl-3-methyl-*N*-(1-phenylethyl)benzamide (10b)

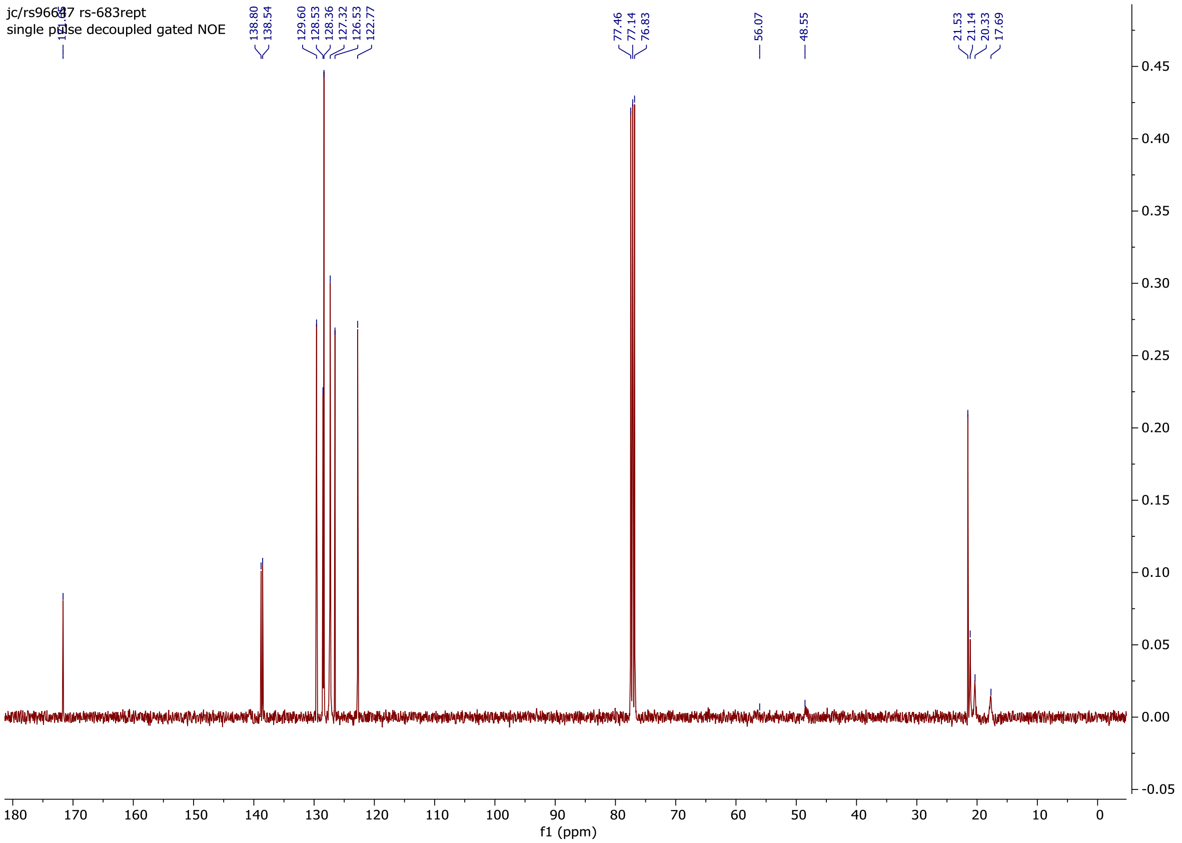

^{1}H NMR

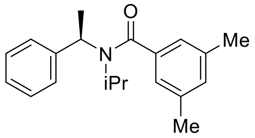

(R)-*N*-isopropyl-3,5-dimethyl-*N*-(1-phenylethyl)benzamide (10c)

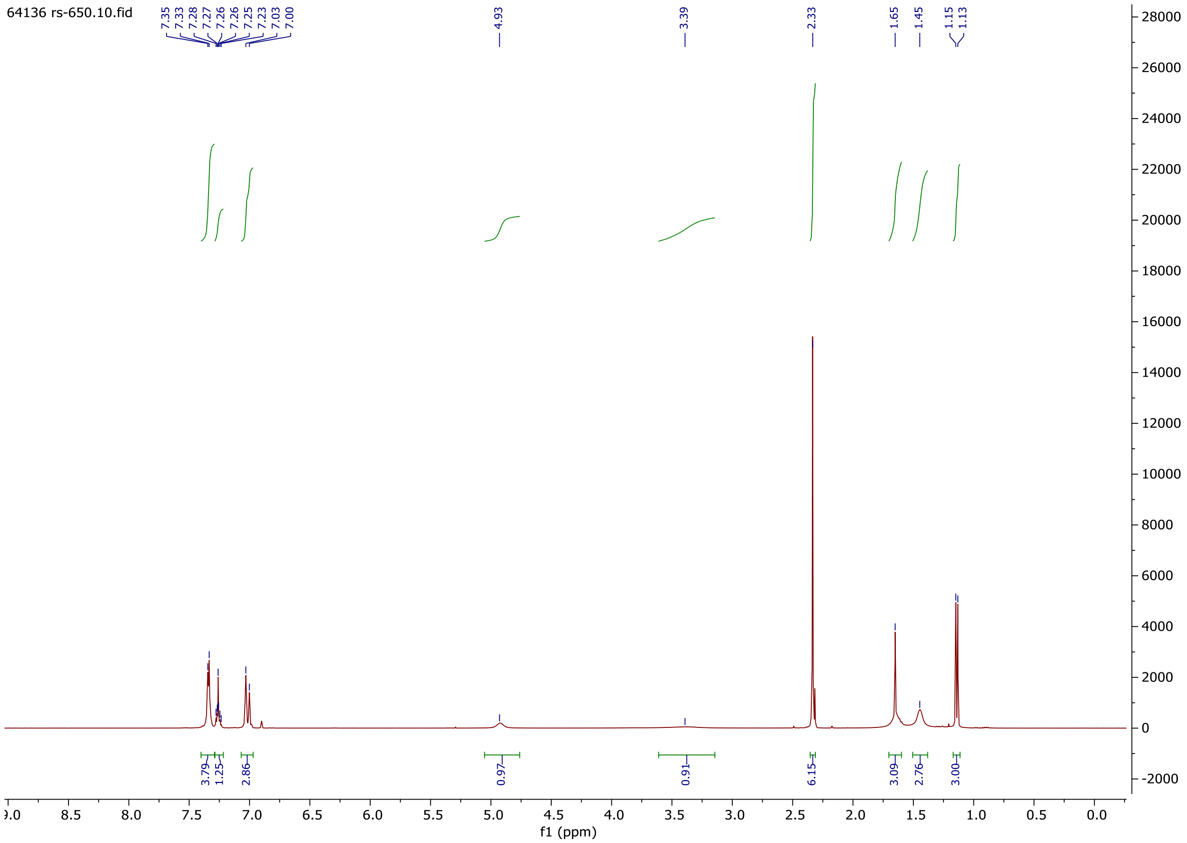

^{13}C NMR

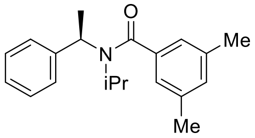

***(R)*-*N*-isopropyl-3,5-dimethyl-*N*-(1-phenylethyl)benzamide (10c)**

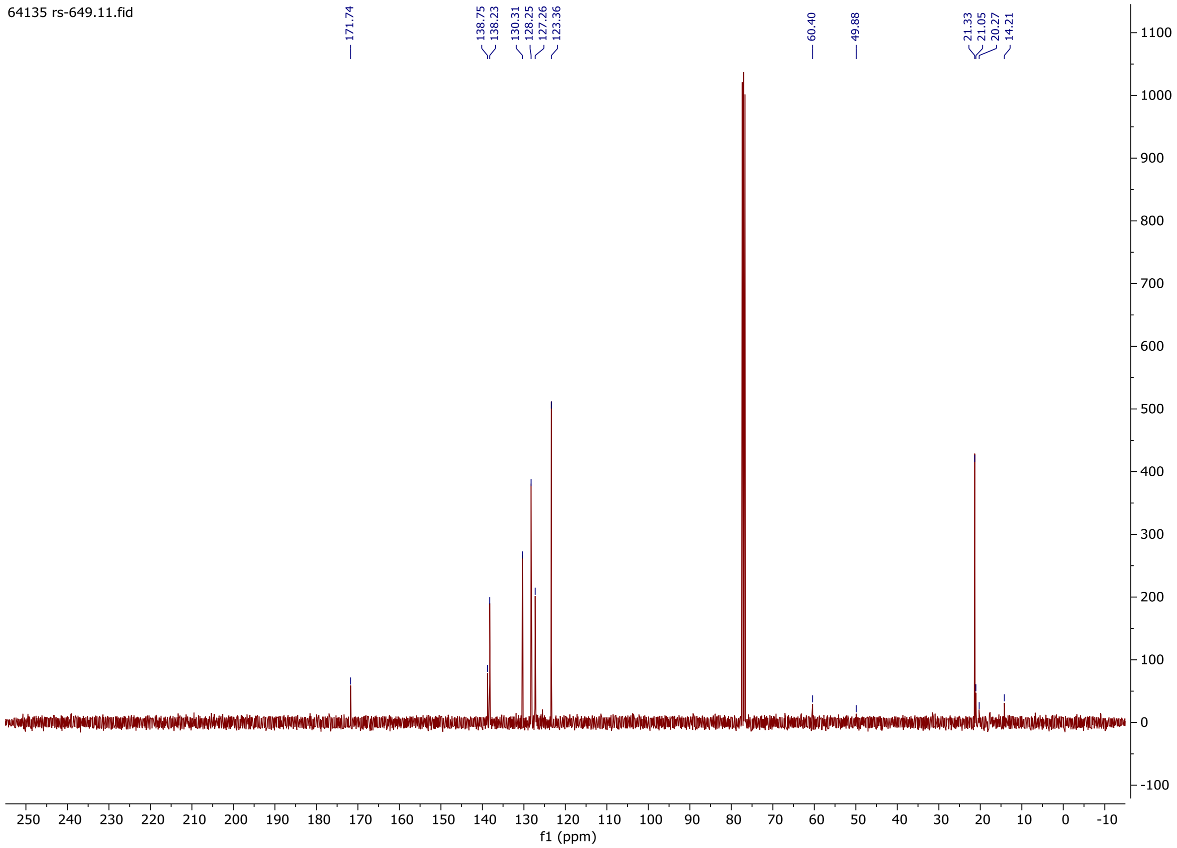

^{1}H NMR

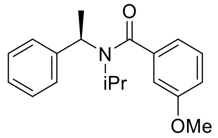

(R)-N-isopropyl-3-methoxy-N-(1-phenylethyl)benzamide (10d)

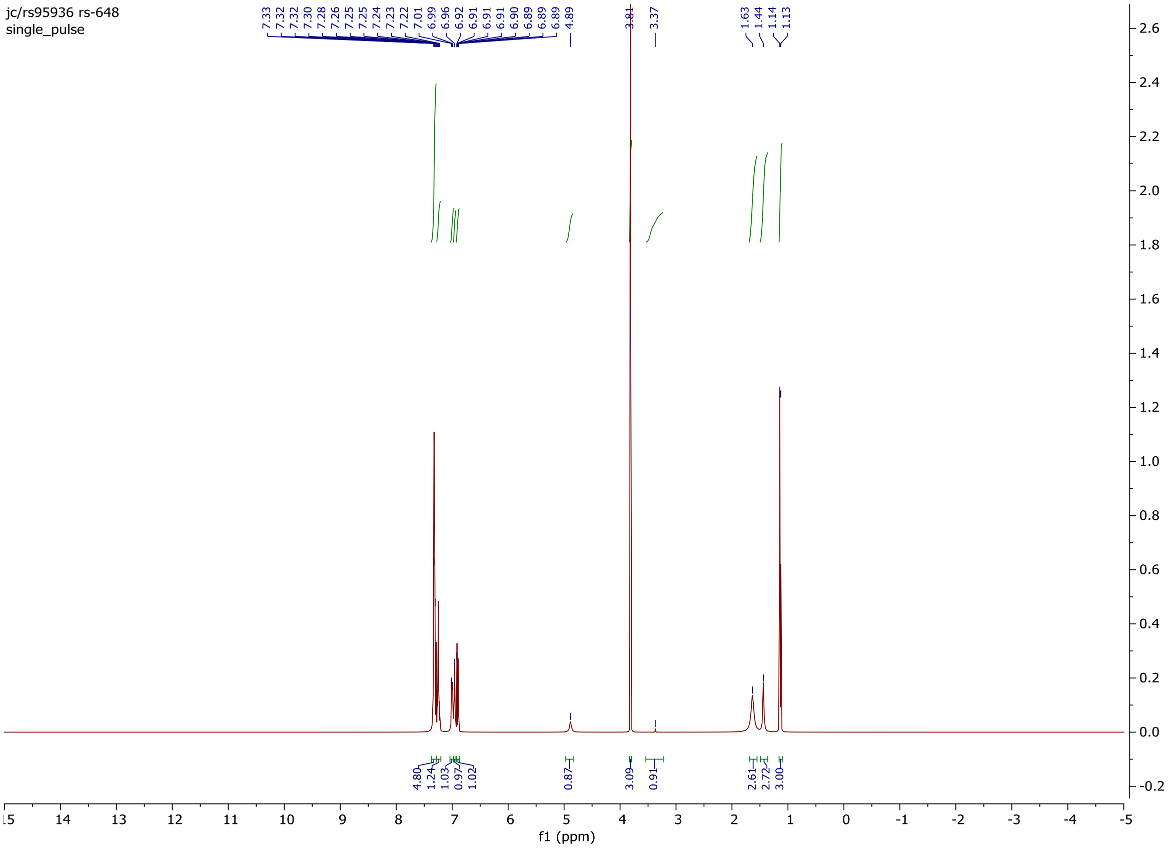

^{13}C NMR

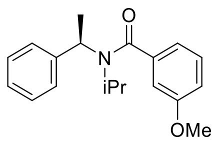

(R)-*N*-isopropyl-3-methoxy-*N*-(1-phenylethyl)benzamide (10d)

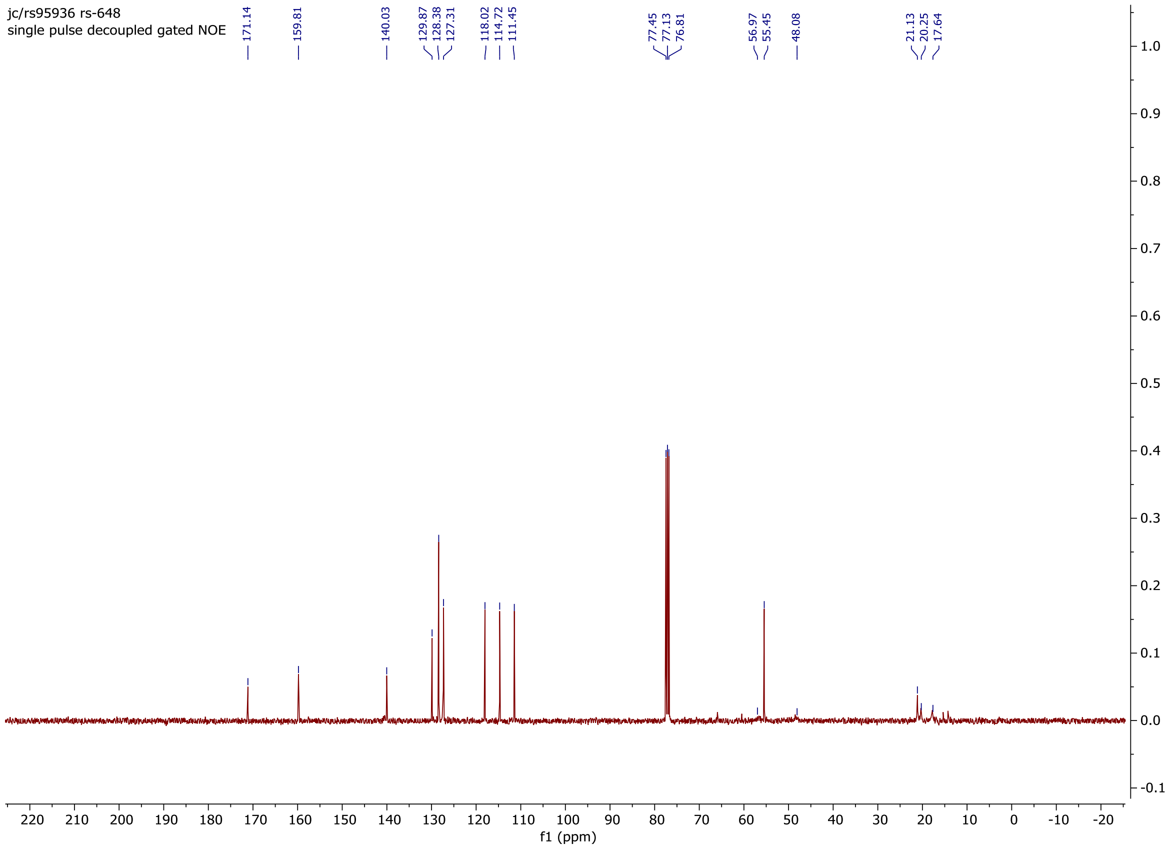

^{1}H NMR

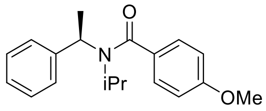

(R)-*N*-isopropyl-4-methoxy-*N*-(1-phenylethyl)benzamide (10e)

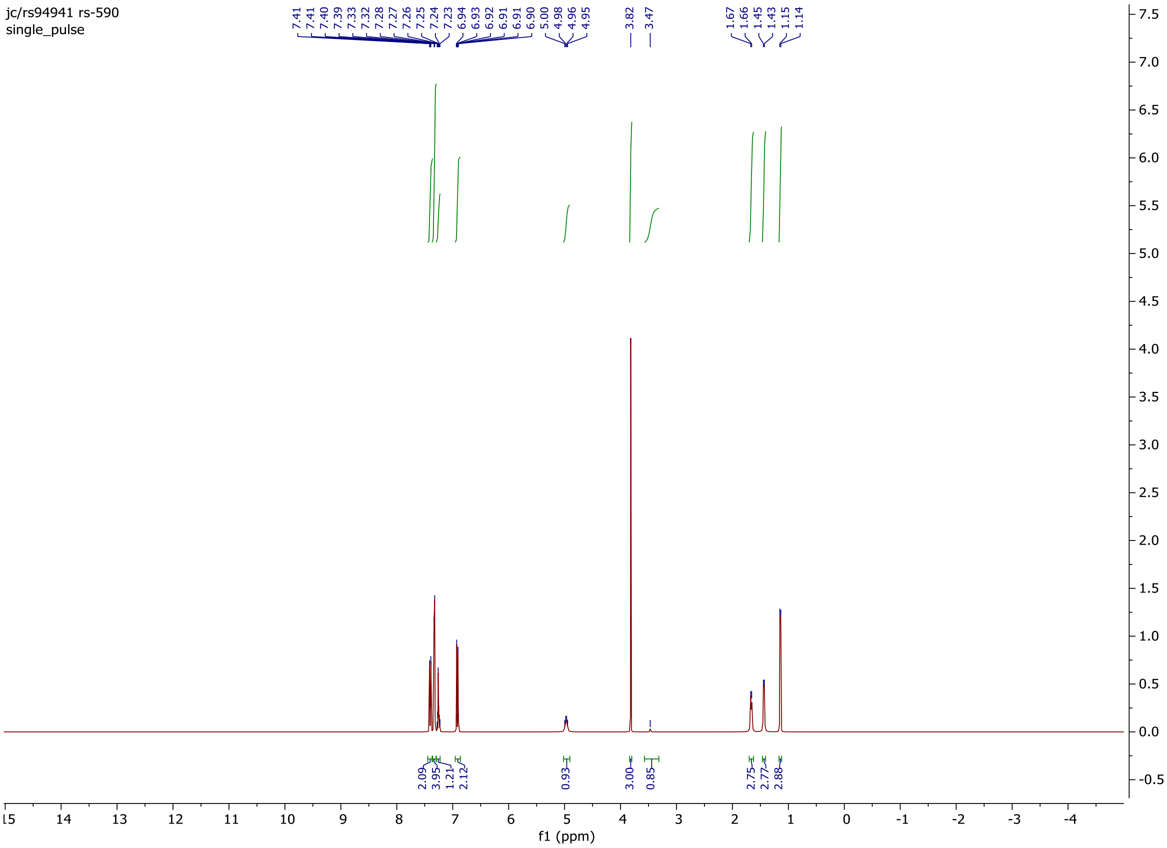

^{13}CNMR

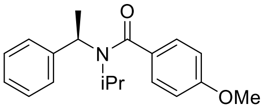

(R)-*N*-isopropyl-4-methoxy-*N*-(1-phenylethyl)benzamide (10e)

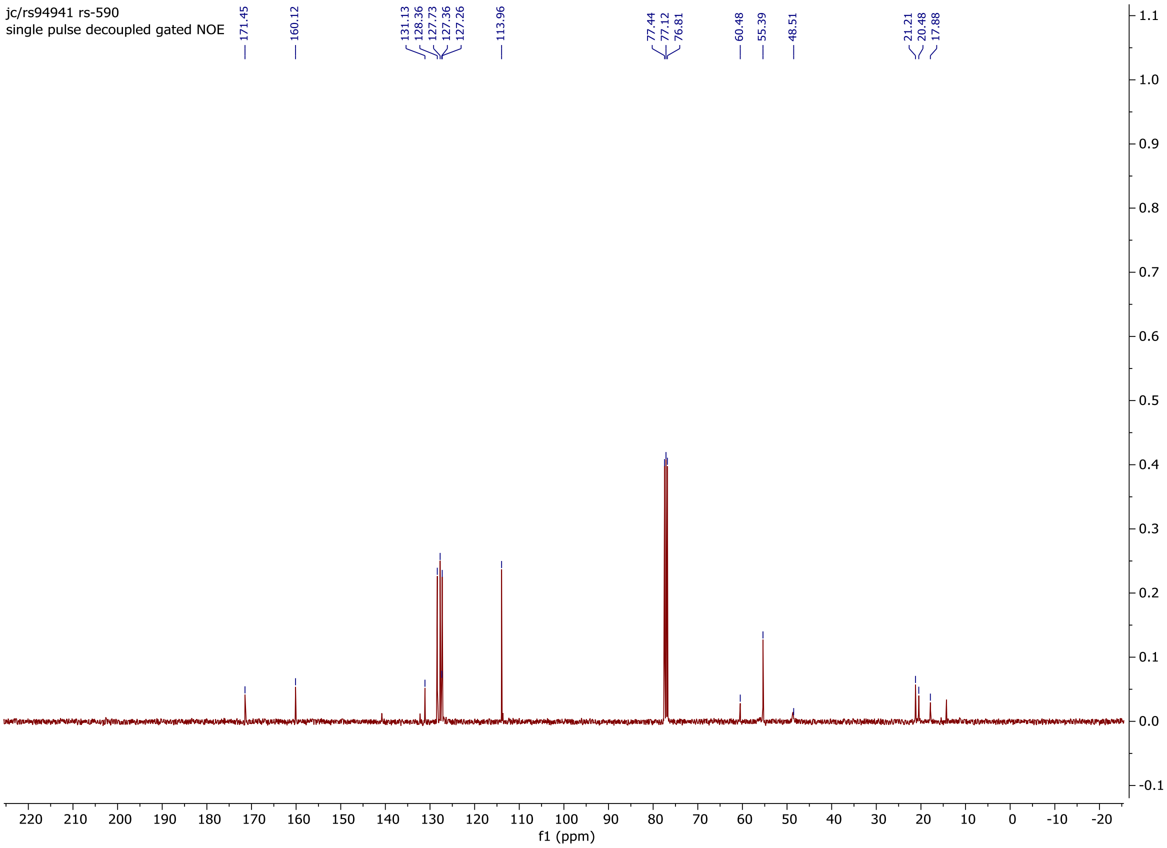

^{1}H NMR

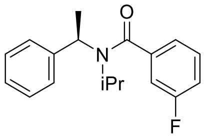

(R)-3-fluoro-*N*-isopropyl-*N*-(1-phenylethyl)benzamide (10f)

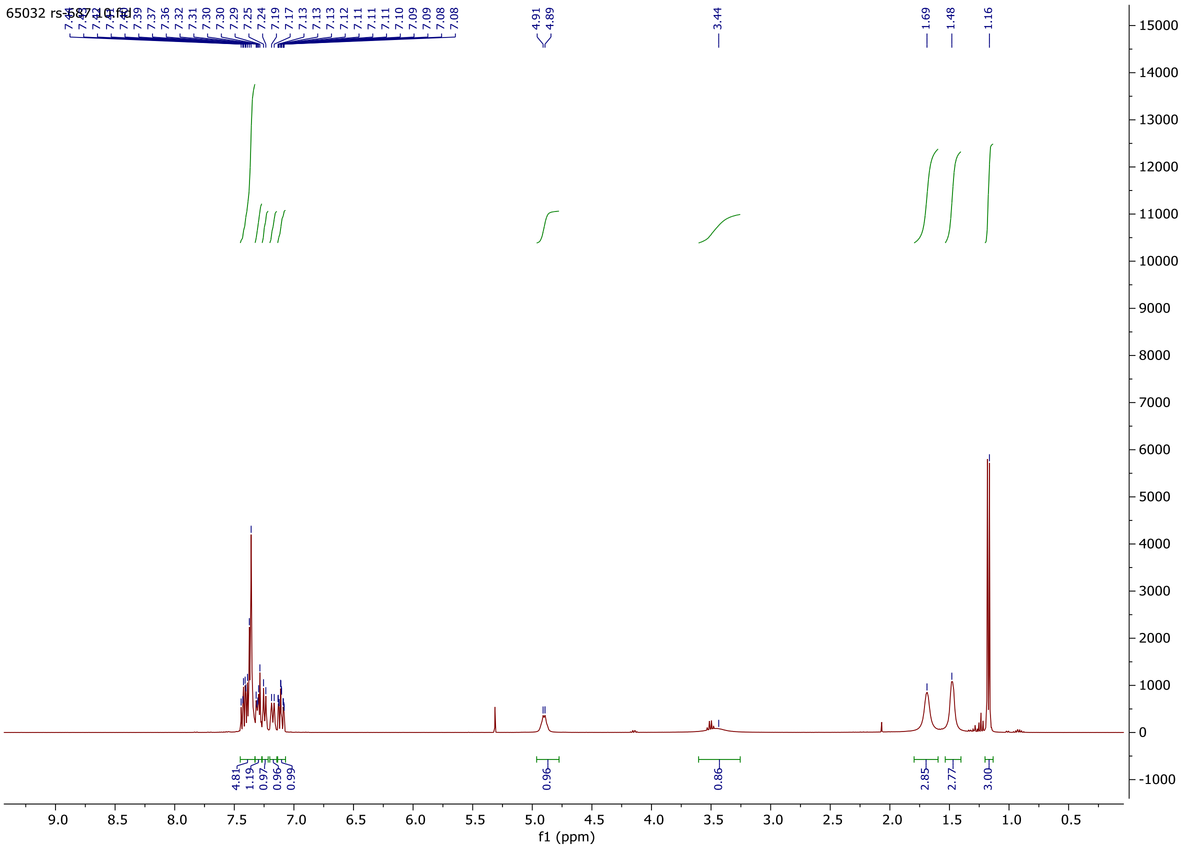

^{13}C NMR

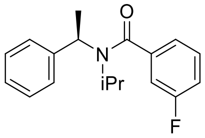

(R)-3-fluoro-N-isopropyl-N-(1-phenylethyl)benzamide (10f)

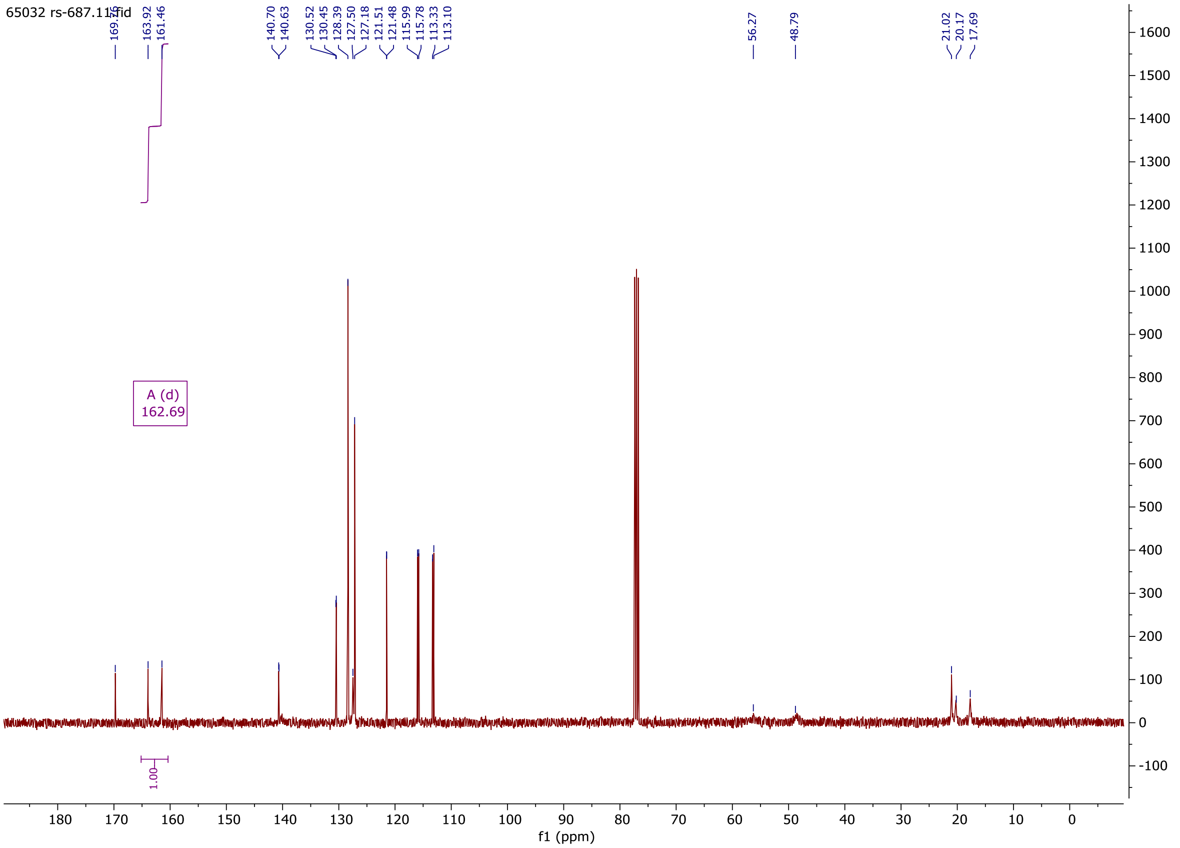

^{1}H NMR

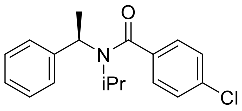

(*R*)-4-chloro-*N*-isopropyl-*N*-(1-phenylethyl)benzamide (10g)

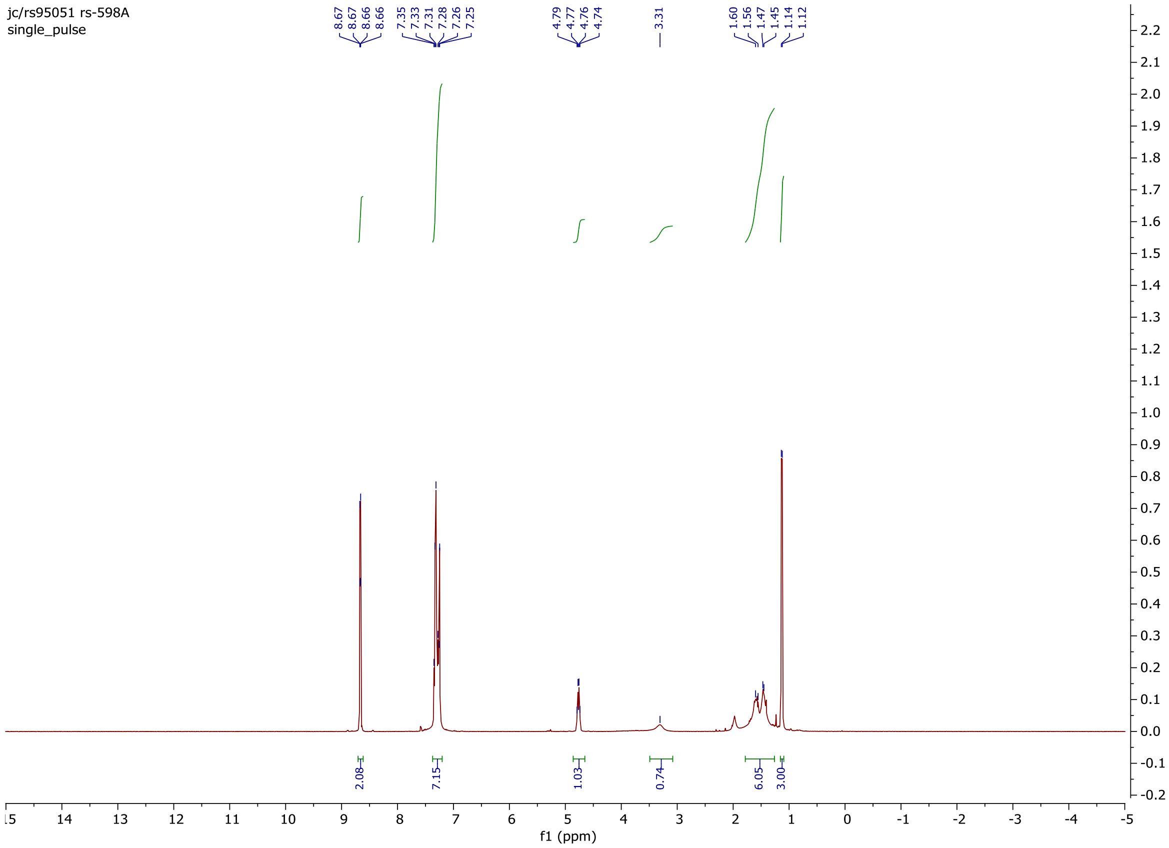

^{13}C NMR

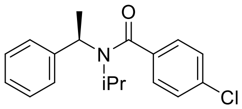

(R)-4-chloro-*N*-isopropyl-*N*-(1-phenylethyl)benzamide (10g)

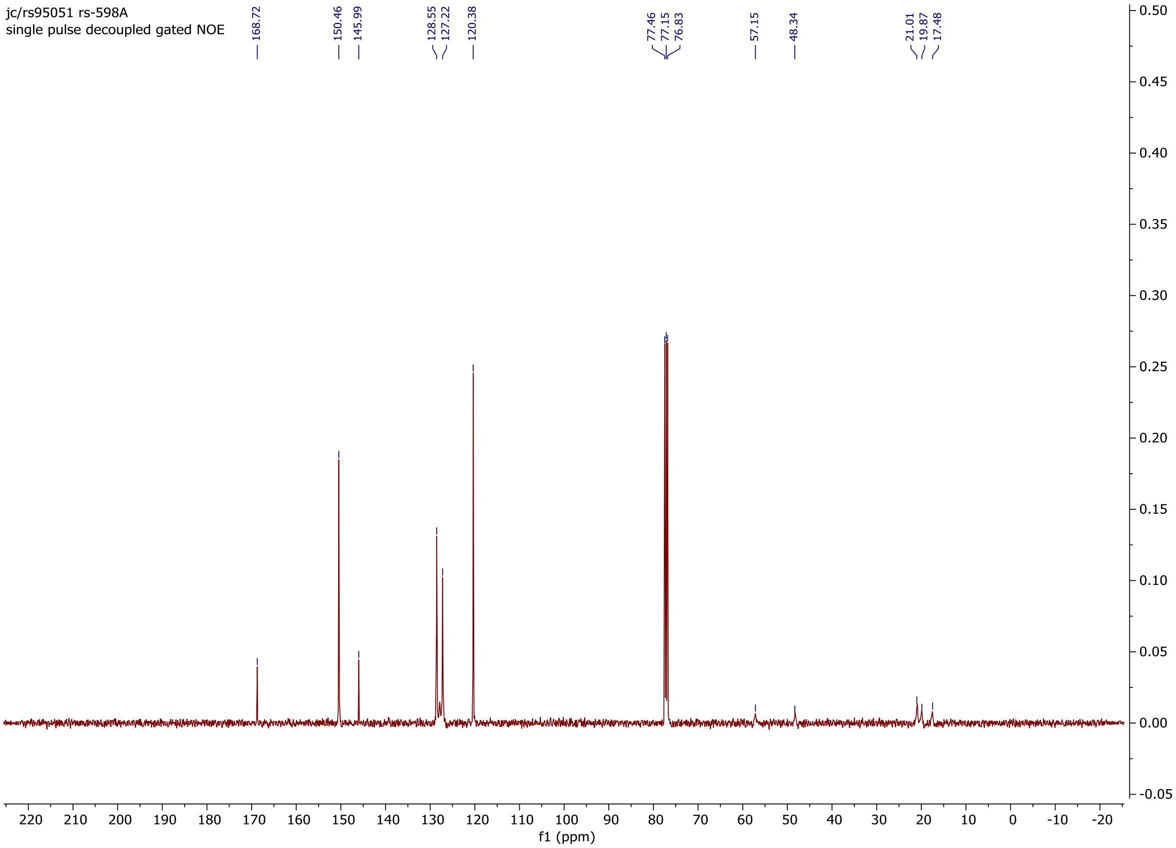

^{1}H NMR

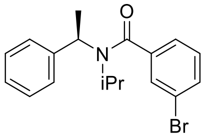

(R)-3-bromo-N-isopropyl-N-(1-phenylethyl)benzamide (10h)

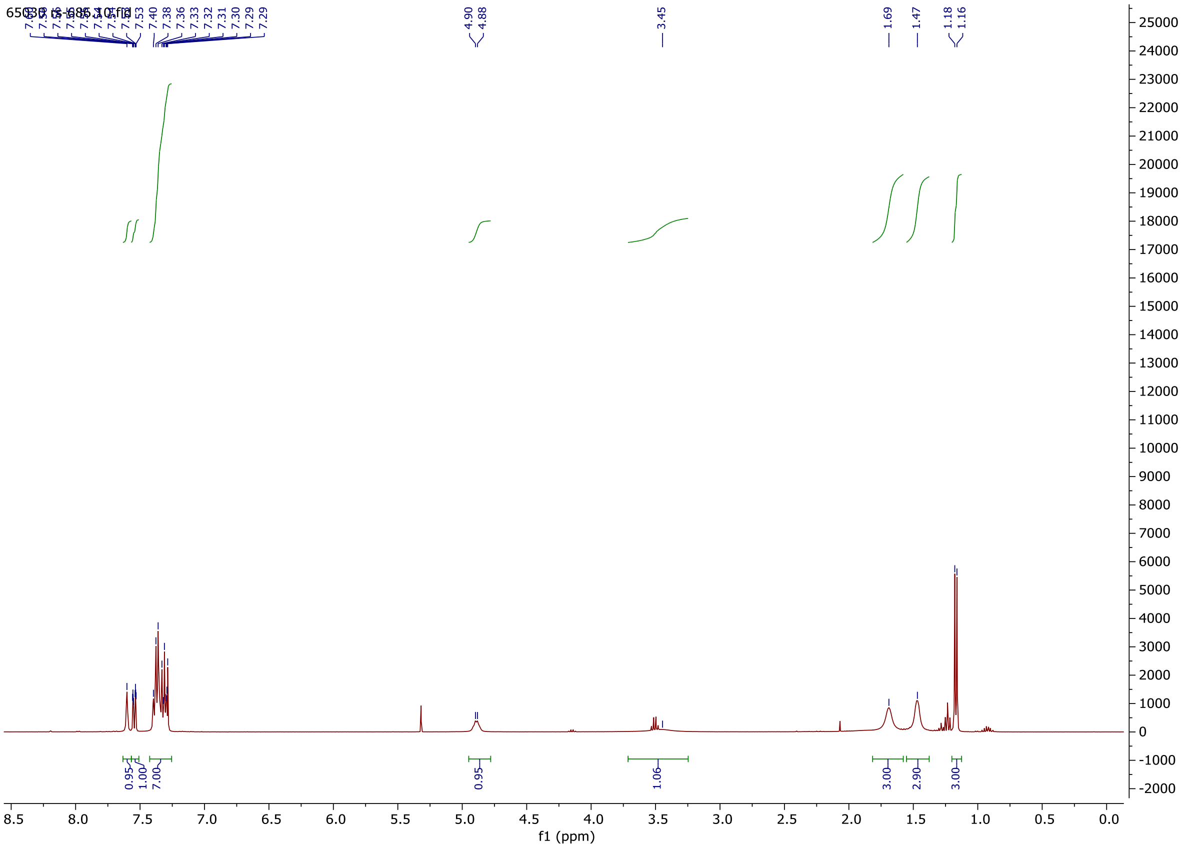

^{13}C NMR

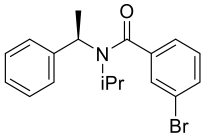

(R)-3-bromo-*N*-isopropyl-*N*-(1-phenylethyl)benzamide (10h)

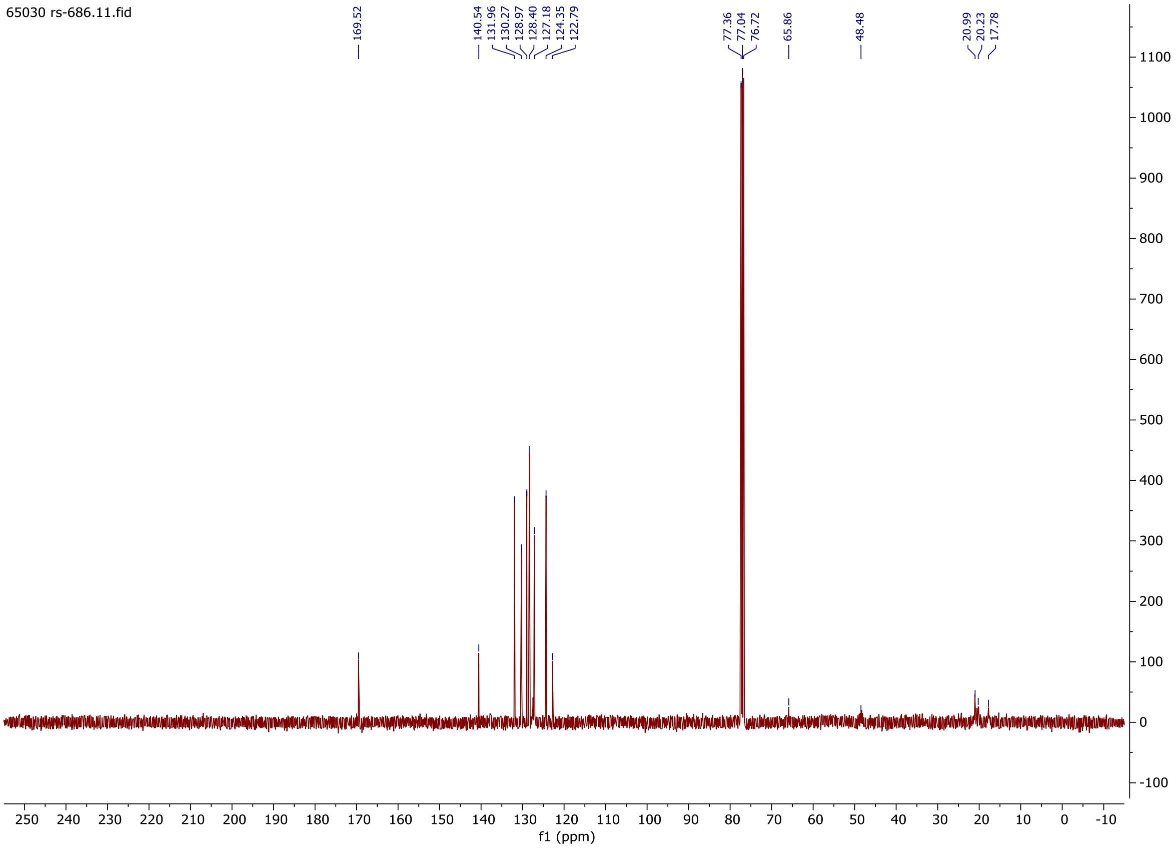

^{1}H NMR

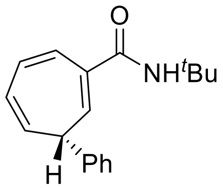

(*R*)-*N*-(tert-butyl)-3-phenylcyclohepta-1,4,6-triene-1-carboxamide (4a)

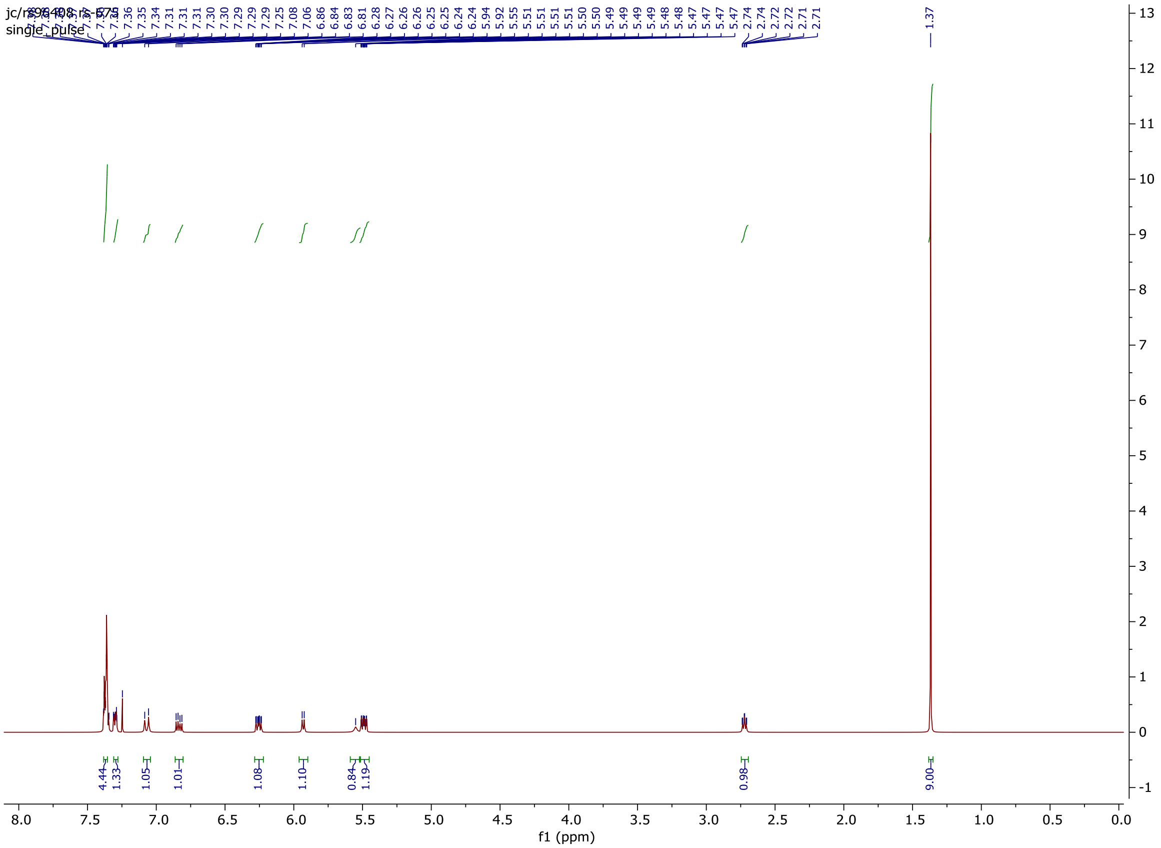

^{13}C NMR

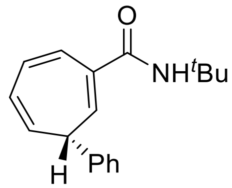

(R)-*N*-(tert-butyl)-3-phenylcyclohepta-1,4,6-triene-1-carboxamide (4a)

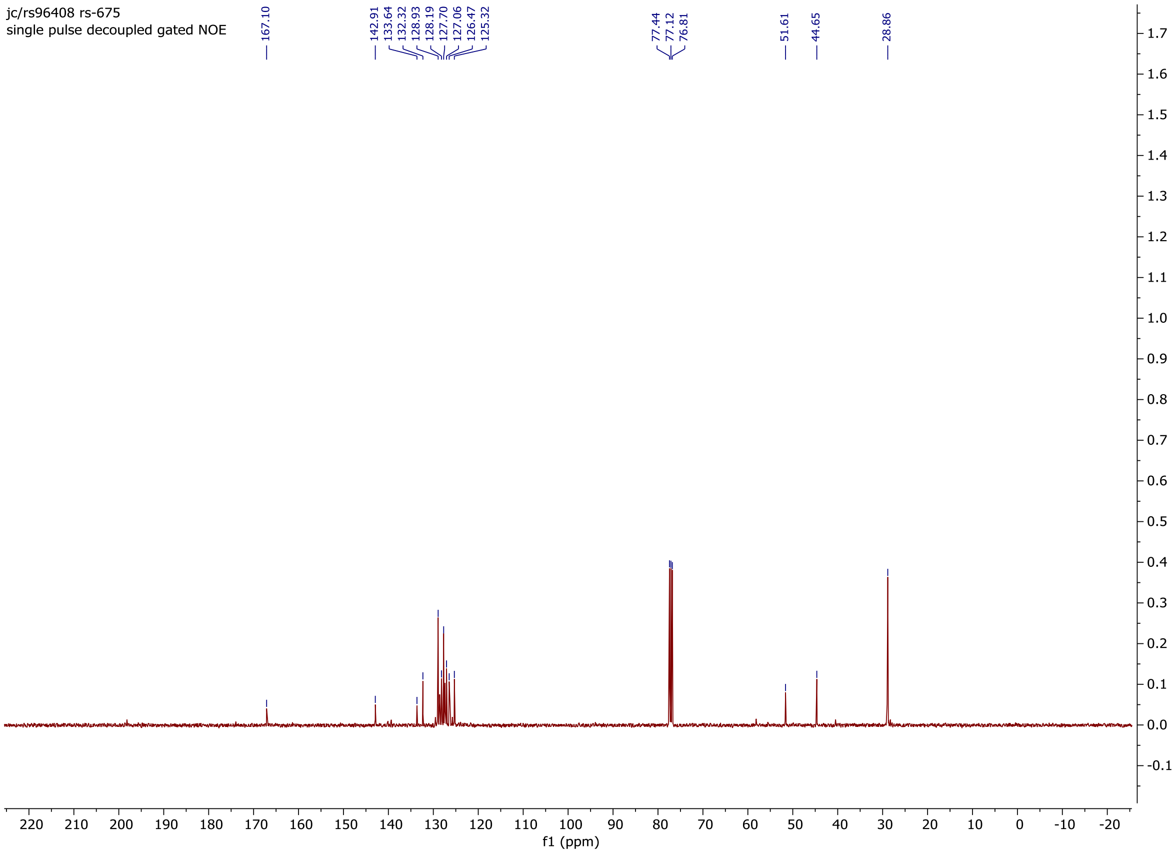

HPLC

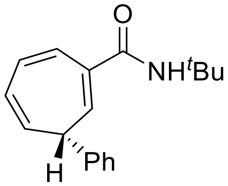

***(R)*-*N*-(tert-butyl)-3-phenylcyclohepta-1,4,6-triene-1-carboxamide (4a)**

Analytical HPLC (Chiral Regis Whelk O1), eluting with IPA–hexane (15:85), showed it to consist of a 3.798:96.202 mixture of two enantiomers with retention times 20.100 and 25.999 min respectively (ee = 92 %).

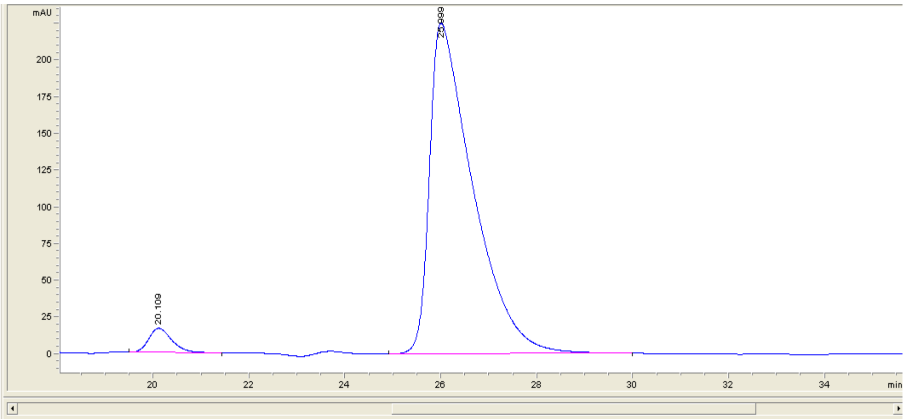

File Information

LC-File	RS-703.D
File Path	E:\CHEM32\1\DATA\RAKESH\RAKESH S 2020-09-29 16-17-39\
Date	29-Sep-20, 16:18:20
Sample	RS-703
Sample Info	85:15 hex:IPA IA
Barcode	
Operator	SYSTEM
Method	RAKESH 85-15 HEXANE-IPA1ML.M
Analysis Time	42.247 min
Sampling Rate	0.0067 min (0.402 sec), 6338 datapoints

#	Time	Area	Height	Width	Area%	Symmetry
1	20.109	559.9	16.5	0.5142	3.798	0.699
2	25.999	14182.4	225.1	0.9051	96.202	0.341

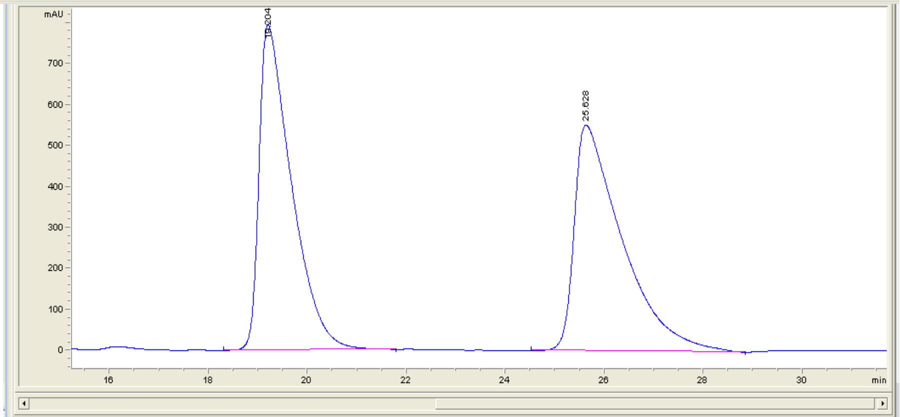

File Information

LC-File	RS-688.D
File Path	E:\CHEM32\1\DATA\RAKESH\RAKESH S 2020-09-23 10-46-48\
Date	23-Sep-20, 10:47:29
Sample	RS-688
Sample Info	85:15 hex:IPA IA
Barcode	
Operator	SYSTEM
Method	RAKESH 85-15 HEXANE-IPA1ML.M
Analysis Time	31.813 min
Sampling Rate	0.0067 min (0.402 sec), 4773 datapoints

#	Time	Area	Height	Width	Area%	Symmetry
1	19.204	36107.8	794.2	0.6584	49.289	0.36
2	25.628	37149.4	551.7	0.9435	50.711	0.304

^{1}H NMR

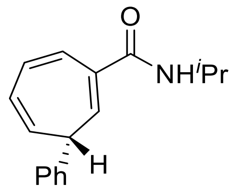

***(R)*-*N*-isopropyl-3-phenylcyclohepta-1,4,6-triene-1-carboxamide (4a')**

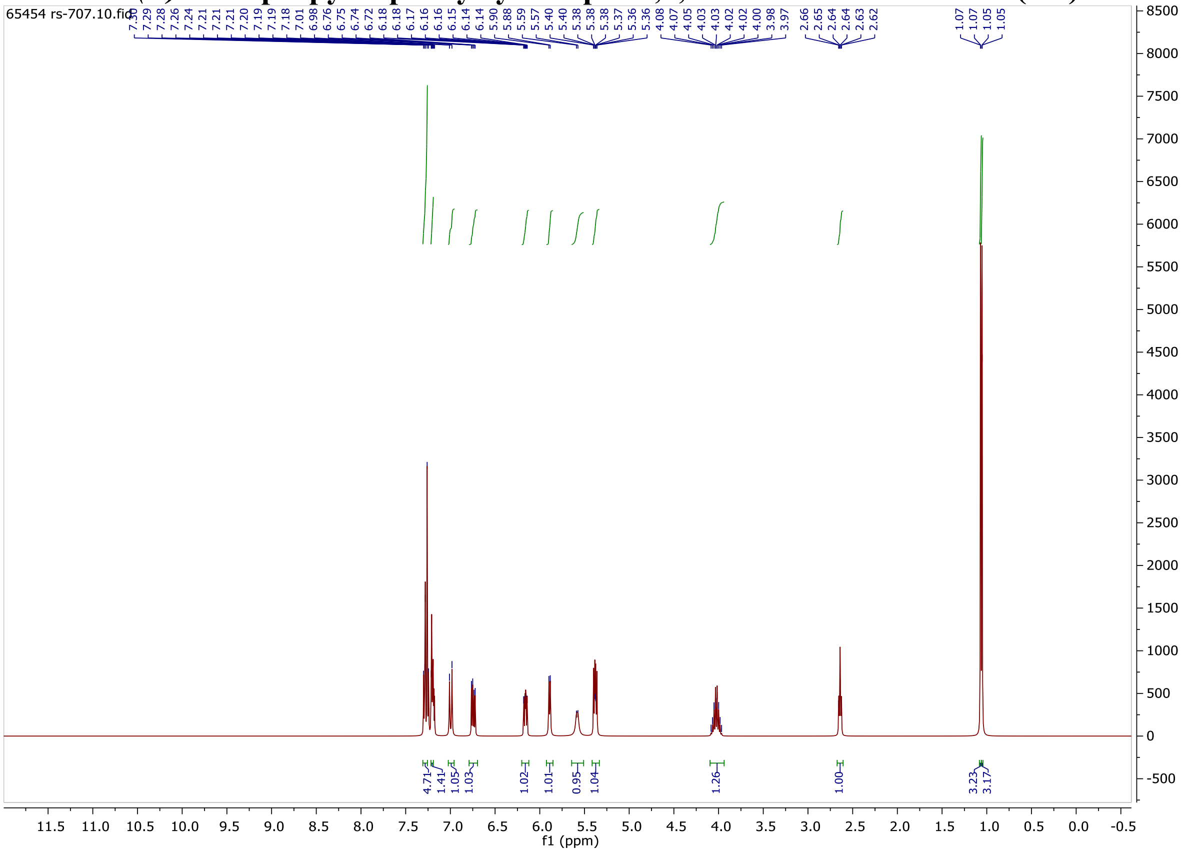

^{13}C NMR

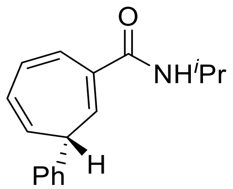

***(R)*-*N*-isopropyl-3-phenylcyclohepta-1,4,6-triene-1-carboxamide (4a')**

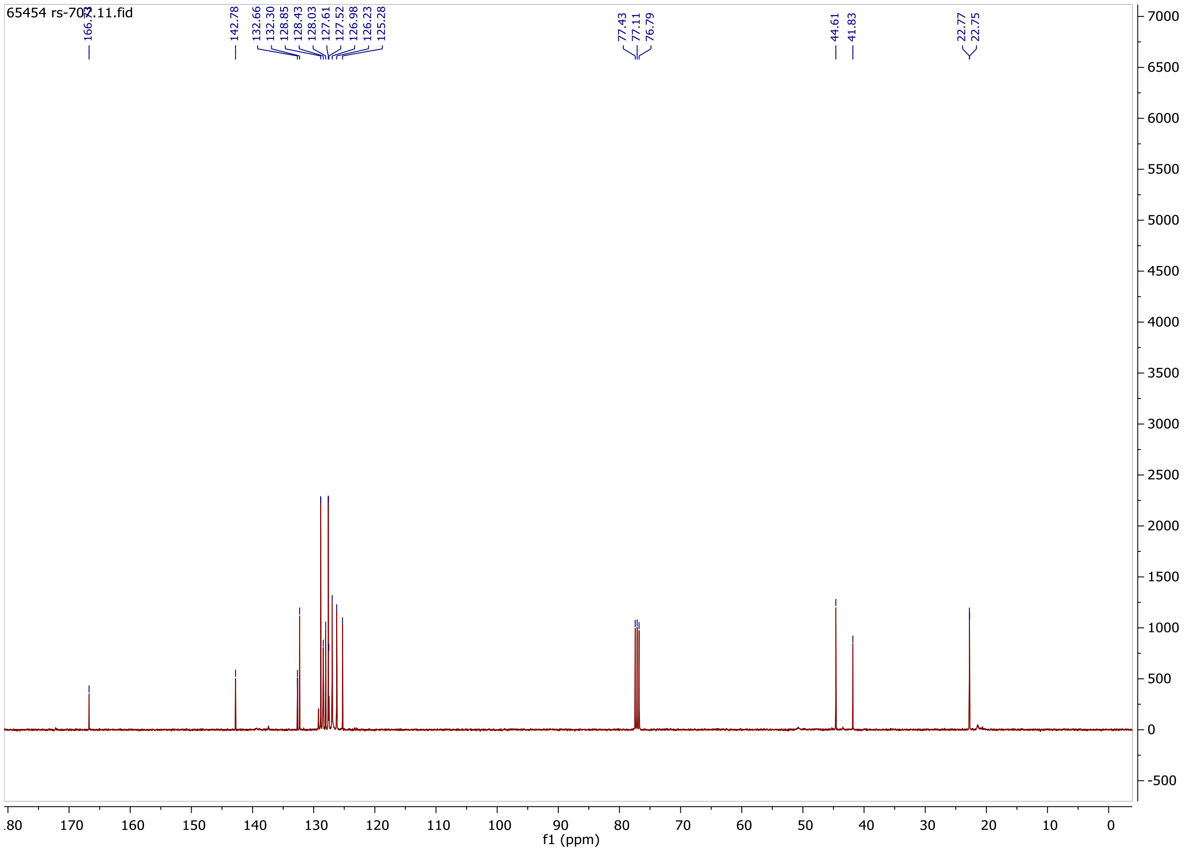

HPLC

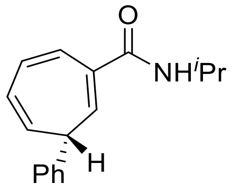

***(R)*-*N*-isopropyl-3-phenylcyclohepta-1,4,6-triene-1-carboxamide (4a')**

Analytical HPLC (IA column), eluting with IPA–hexane (02:98), showed it to consist of a 42:58 mixture of two enantiomers with retention times 18.22 and 24.32 min respectively.

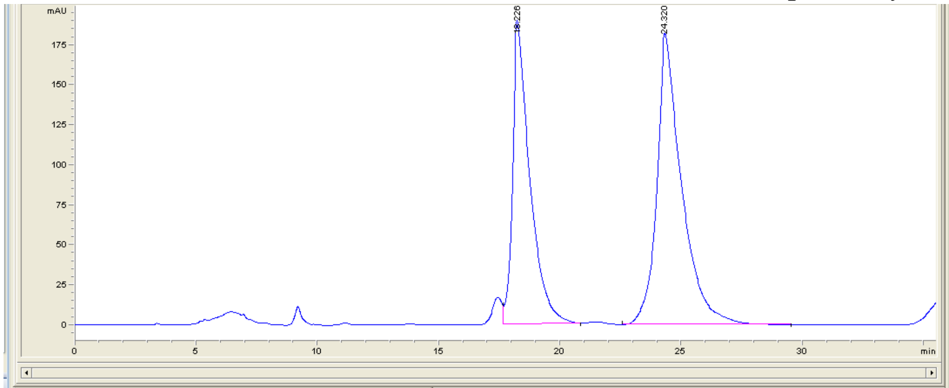

File Information

LC-File	RS-711(1) IA.D
File Path	E:\CHEM32\1\DATA\RAKESH\RAKESH S 2021-02-26 17-29-00\
Date	26-Feb-21, 17:29:48
Sample	RS-711(1) IA
Sample Info	98:02 hex:IPA IA
Barcode	
Operator	SYSTEM
Method	RAKESH 98-02 HEXANE-IPA1ML.M
Analysis Time	35.513 min
Sampling Rate	0.0067 min (0.402 sec), 5328 datapoints

#	Time	Area	Height	Width	Area%	Symmetry
1	18.226	9352.3	189.8	0.6769	41.996	0.362
2	24.32	12917	182.1	0.9374	58.004	0.485

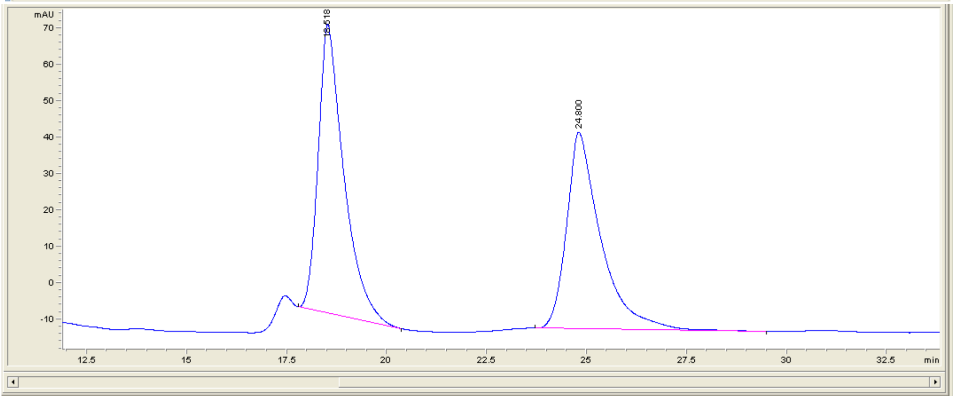

File Information

LC-File	RS-707 IA.D
File Path	E:\CHEM32\1\DATA\RAKESH\RAKESH S 2021-02-26 18-42-28\
Date	26-Feb-21, 18:43:15
Sample	RS-707 IA
Sample Info	98:02 hex:IPA IA
Barcode	
Operator	SYSTEM
Method	RAKESH 98-02 HEXANE-IPA1ML.M
Analysis Time	33.833 min
Sampling Rate	0.0067 min (0.402 sec), 5076 datapoints

#	Time	Area	Height	Width	Area%	Symmetry
1	18.518	3536.5	79.3	0.6466	52.424	0.545
2	24.8	3209.5	54	0.8176	47.576	0.521

^{1}H NMR

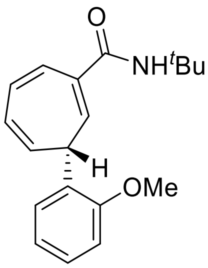

***(R)-N-(tert-butyl)-3-(2-methoxyphenyl)cyclohepta-1,4,6-triene-1-carboxamide* (4b)**

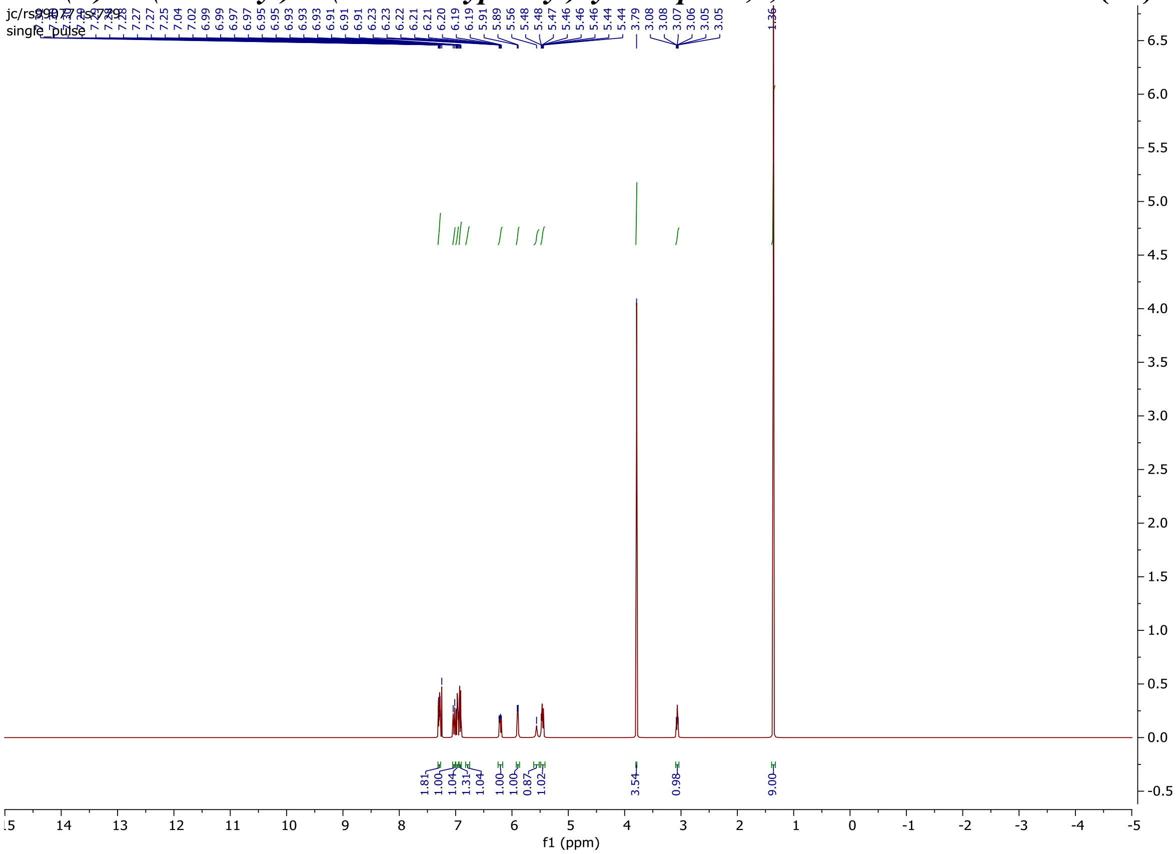

^{13}C NMR

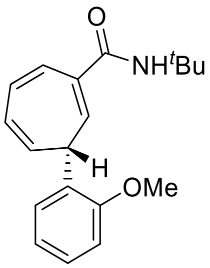

(R)-N-(tert-butyl)-3-(2-methoxyphenyl)cyclohepta-1,4,6-triene-1-carboxamide **(4b)**

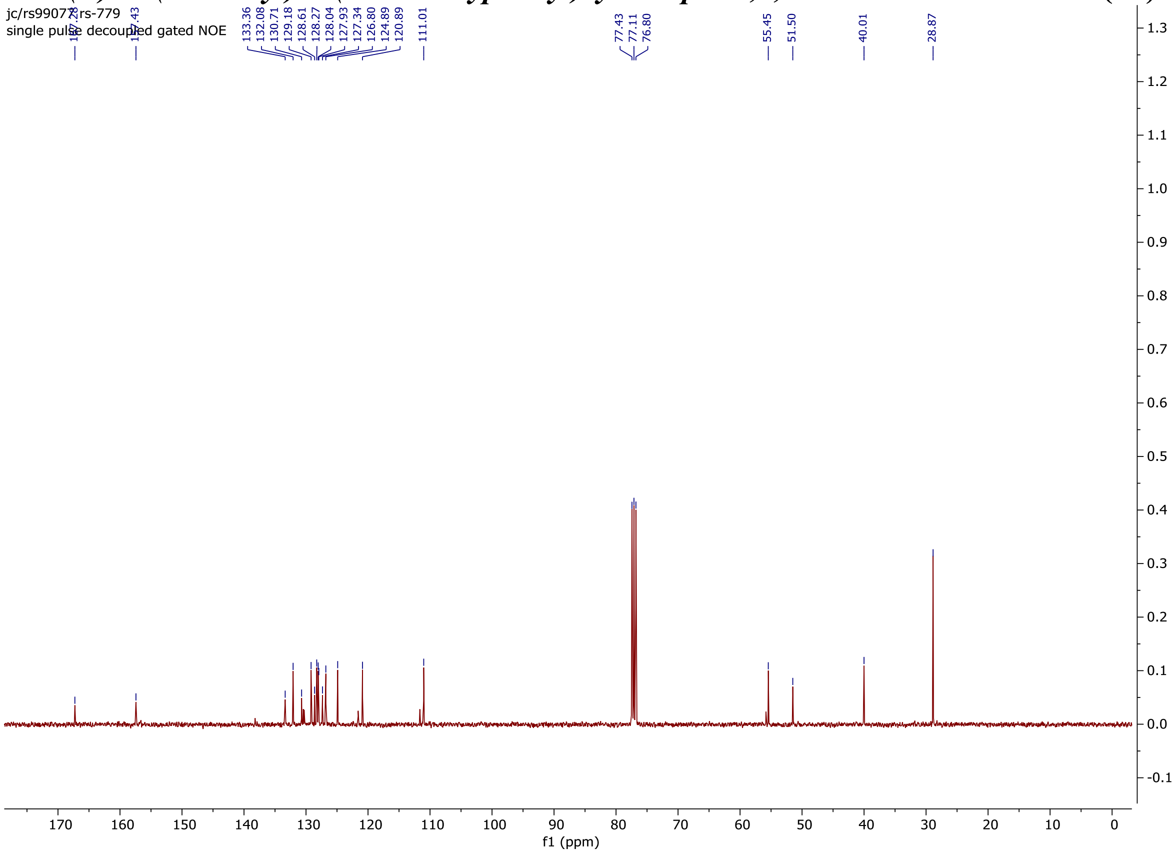

HPLC

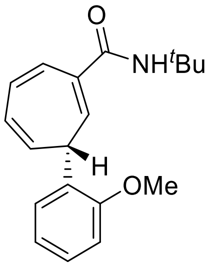

(R)-N-(tert-butyl)-3-(2-methoxyphenyl)cyclohepta-1,4,6-triene-1-carboxamide **(4b)**

Analytical HPLC (IA Column), eluting with hexane-IPA–(3:97), showed it to consist of 8:92 mixture of two enantiomers with retention times 8.93 mins (minor) and 10.11 mins (major), respectively.

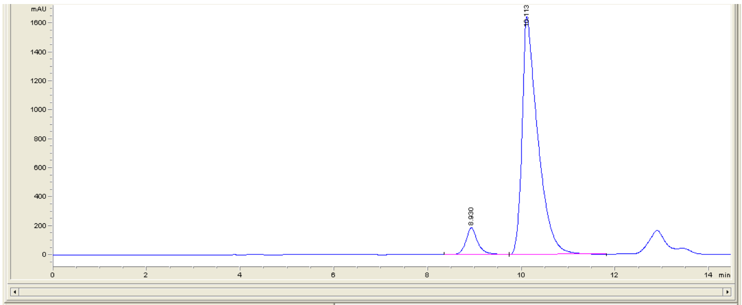

File Information

LC-File	RS-781 3%.D
File Path	E:\CHEM32\1\DATA\RAKESH\RAKESH S 2020-12-14 18-24-31\
Date	14-Dec-20, 18:25:18
Sample	RS-781 3%
Sample Info	97:3 hex:IPA IA
Barcode	
Operator	SYSTEM
Method	RAKESH 97-03 HEXANE-IPA1ML.M
Analysis Time	14.473 min
Sampling Rate	0.0067 min (0.402 sec), 2172 datapoints

#	Time	Area	Height	Width	Area%	Symmetry
1	8.93	3259.2	185.6	0.2528	7.833	0.788
2	10.113	38350.6	1643.4	0.3263	92.167	0.4

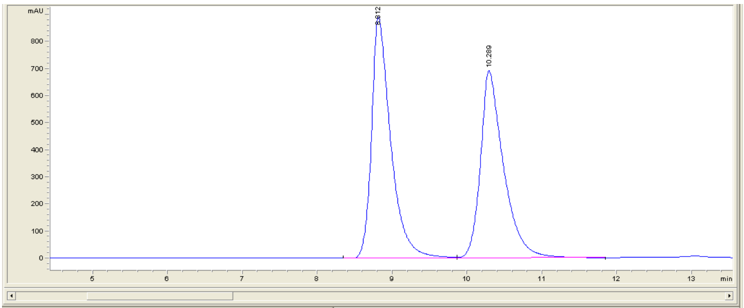

File Information

LC-File	RS-779 3%.D
File Path	E:\CHEM32\1\DATA\RAKESH\RAKESH S 2020-12-14 17-38-06\
Date	14-Dec-20, 17:38:53
Sample	RS-779 3%
Sample Info	97:3 hex:IPA IA
Barcode	
Operator	SYSTEM
Method	RAKESH 97-03 HEXANE-IPA1ML.M
Analysis Time	44.267 min
Sampling Rate	0.0067 min (0.402 sec), 6641 datapoints

#	Time	Area	Height	Width	Area%	Symmetry
1	8.812	15842.6	892.1	0.2612	51.663	0.515
2	10.289	14823	692.1	0.3059	48.337	0.547

^{1}H NMR

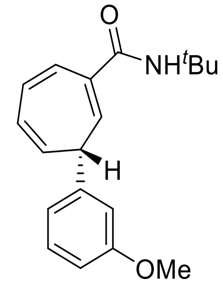

***(R)*-*N*-(tert-butyl)-3-(3-methoxyphenyl)cyclohepta-1,4,6-triene-1-carboxamide (4c)**

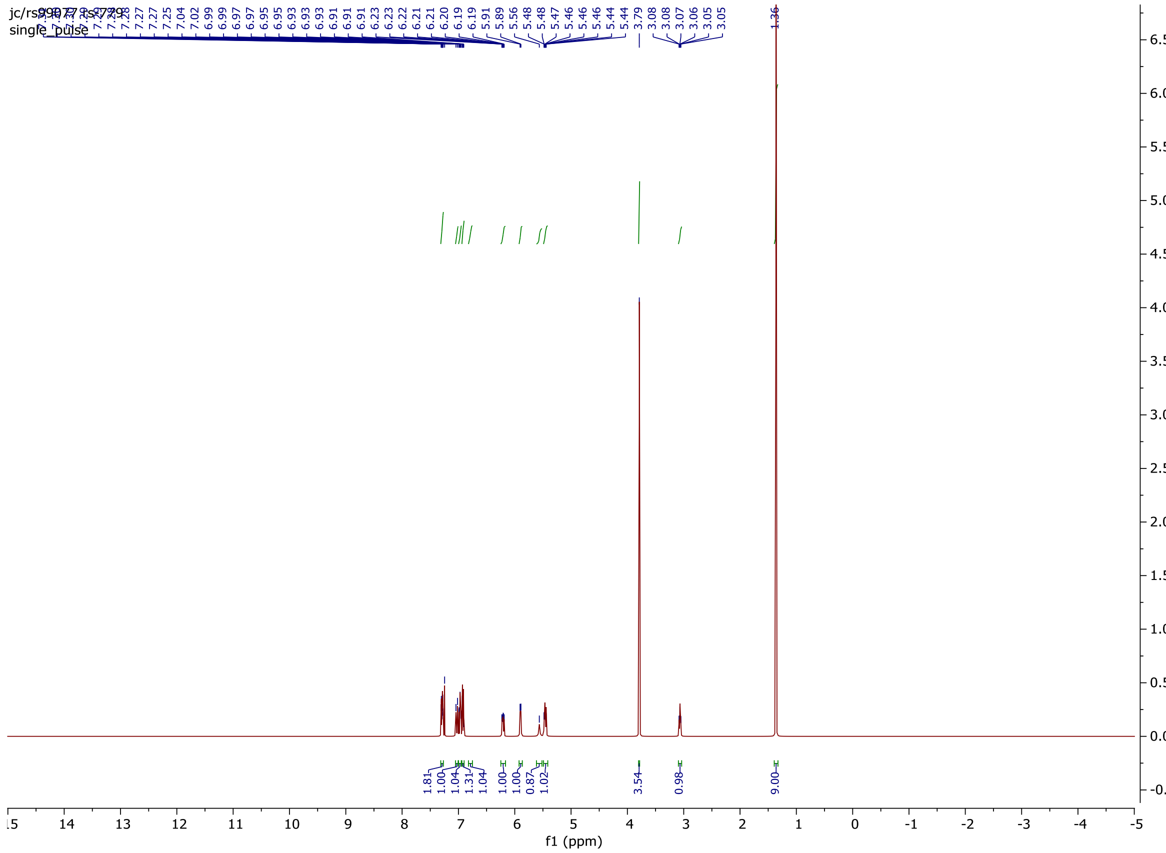

^{13}C NMR

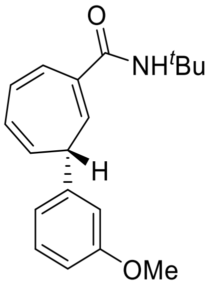

***(R)*-*N*-(tert-butyl)-3-(3-methoxyphenyl)cyclohepta-1,4,6-triene-1-carboxamide (4c)**

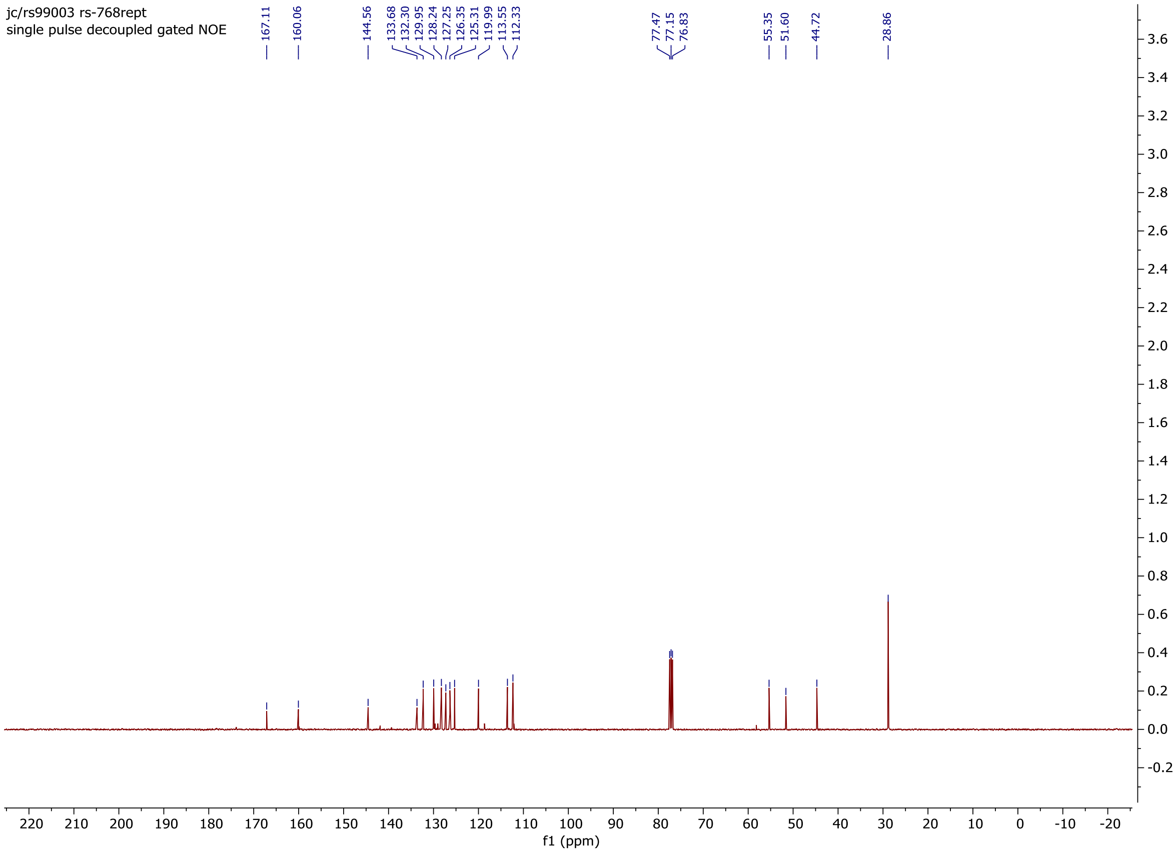

HPLC

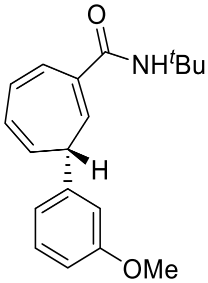

(R)-N-(tert-butyl)-3-(3-methoxyphenyl)cyclohepta-1,4,6-triene-1-carboxamide (4c)

Analytical HPLC (IA Column), eluting with hexane-IPA (99:1), showed it to consist of a 9:91 mixture of two enantiomers with retention times 14.11 mins(minor) and 19.42 mins (major), respectively

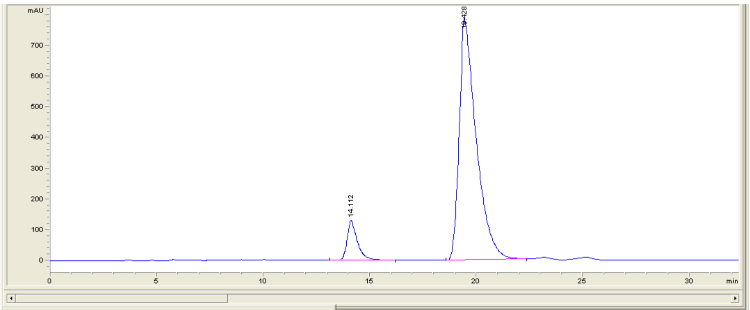

File Information

LC-File	RS-783 1%.D
File Path	E:\CHEM32\1\DATA\RAKESH\RAKESH S 2020-12-15 14-22-16\
Date	15-Dec-20, 14:23:03
Sample	RS-783 1%
Sample Info	99:1 hex:IPA IA
Barcode	
Operator	SYSTEM
Method	RAKESH 99-01 HEXANE-IPA1ML.M
Analysis Time	109.073 min
Sampling Rate	0.0067 min (0.402 sec), 16362 datapoints

#	Time	Area	Height	Width	Area%	Symmetry
1	14.112	4316.8	130.2	0.4709	9.022	0.56
2	19.428	43530.6	786.9	0.7774	90.978	0.408

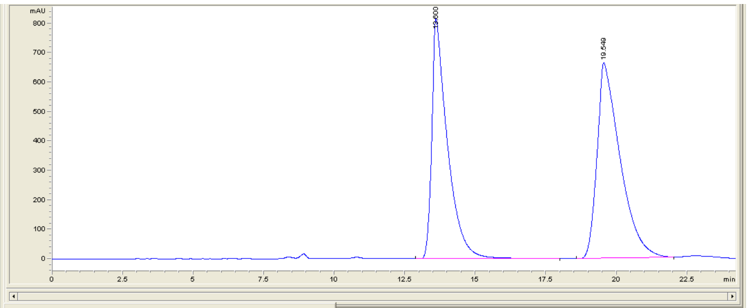

File Information

LC-File	RS-768REPT 1%.D
File Path	E:\CHEM32\1\DATA\RAKESH\RAKESH S 2020-12-15 16-58-21\
Date	15-Dec-20, 16:59:08
Sample	RS-768 rept 1%
Sample Info	99:1 hex:IPA IA
Barcode	
Operator	SYSTEM
Method	RAKESH 99-01 HEXANE-IPA1ML.M
Analysis Time	24.233 min
Sampling Rate	0.0067 min (0.402 sec), 3636 datapoints

#	Time	Area	Height	Width	Area%	Symmetry
1	13.6	31373.4	815.5	0.5269	45.503	0.336
2	19.549	37574.9	663.5	0.8147	54.497	0.399

.

^{1}H NMR

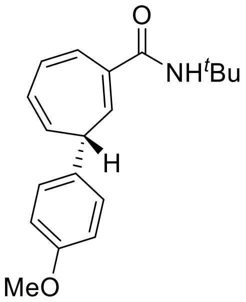

***(R)*-*N*-(tert-butyl)-3-(4-methoxyphenyl)cyclohepta-1,4,6-triene-1-carboxamide (4d)**

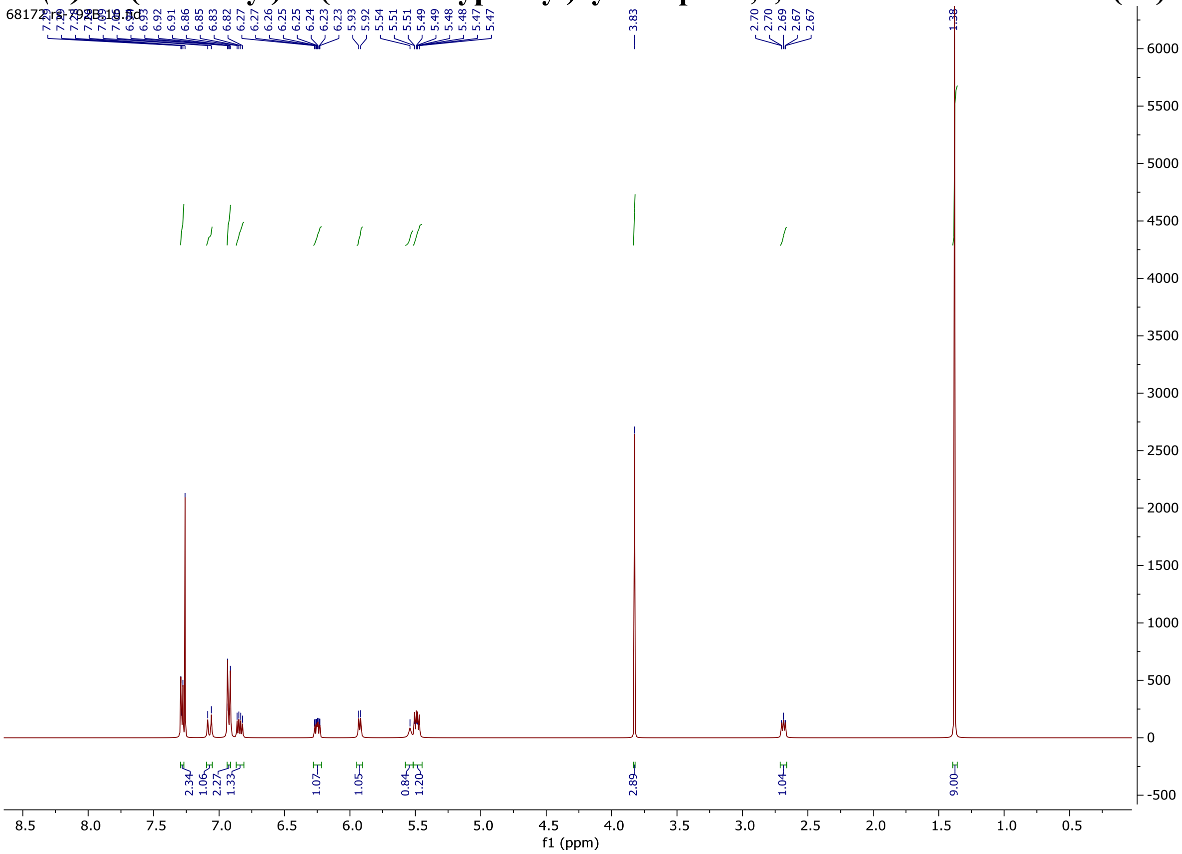

^{13}C NMR

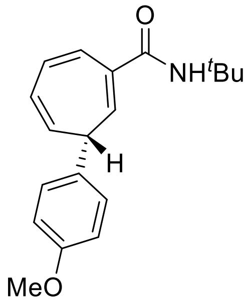

(R)-N-(tert-butyl)-3-(4-methoxyphenyl)cyclohepta-1,4,6-triene-1-carboxamide (4d)

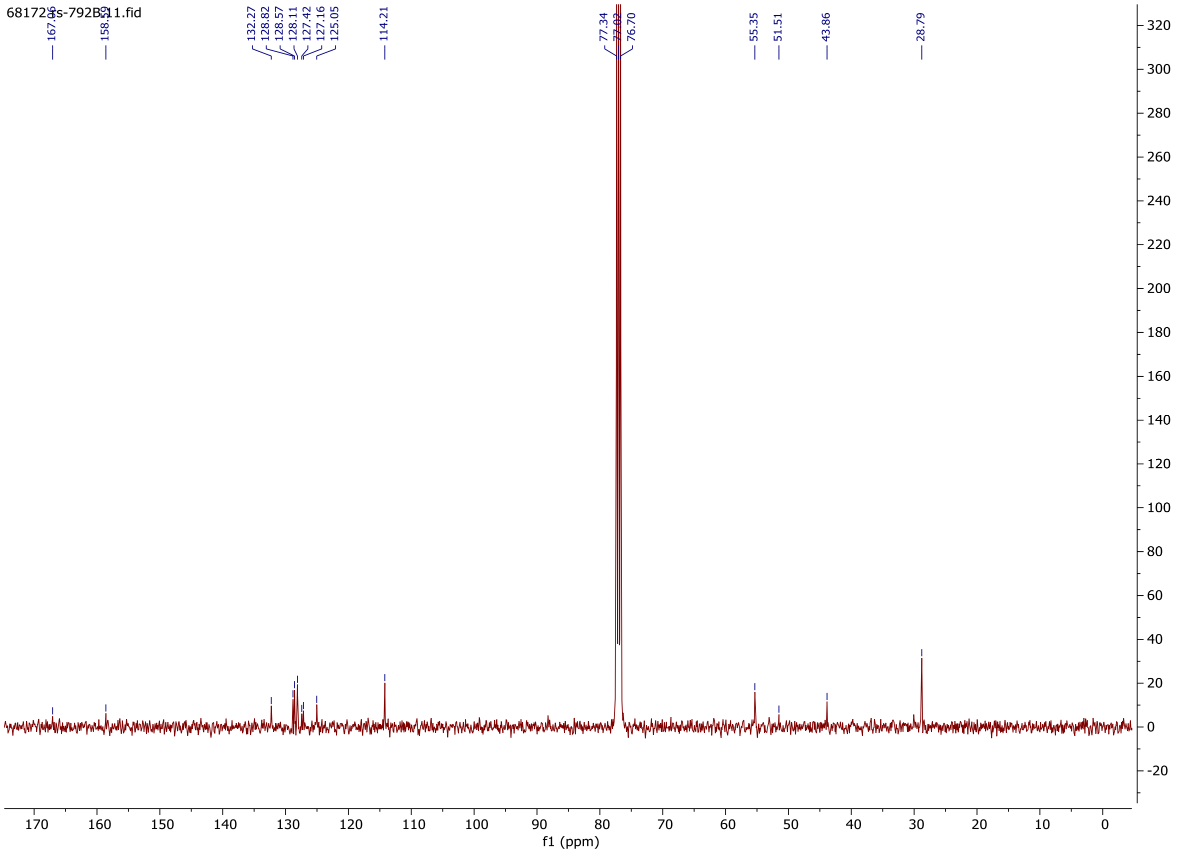

HPLC

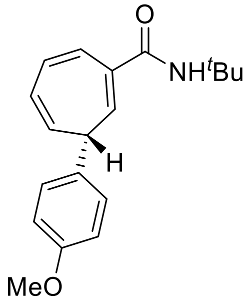

***(R)*-*N*-(tert-butyl)-3-(4-methoxyphenyl)cyclohepta-1,4,6-triene-1-carboxamide (4d)**

Analytical HPLC (Chiral Regis Whelk O1), eluting with hexane-IPA (80:20), showed it to consist of a mixture of 23:77 two enantiomers with retention times tR = 31.285 mins(minor) and 40:568 mins (major) respectively.

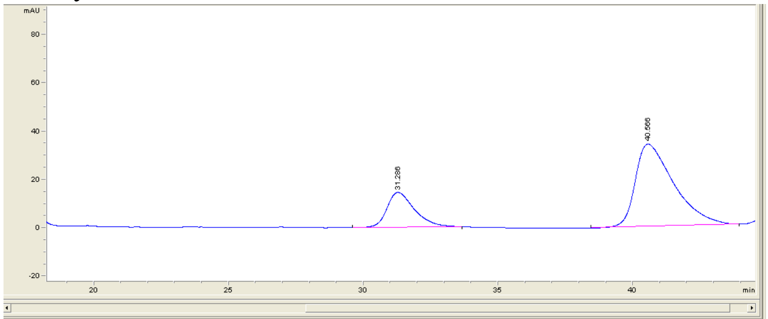

File Information

LC-File	RS-792B 20%.D
File Path	E:\CHEM32\1\DATA\RAKESH\RAKESH S 2021-01-12 15-35-02\
Date	12-Jan-21, 15:35:48
Sample	RS-792B 20%
Sample Info	80:20 hex:IPA IA
Barcode	
Operator	SYSTEM
Method	RAKESH 80-20 HEXANE-IPA1ML.M
Analysis Time	45.613 min
Sampling Rate	0.0067 min (0.402 sec), 6843 datapoints

#	Time	Area	Height	Width	Area%	Symmetry
1	31.286	1038.4	14.4	1.0165	23.384	0.629
2	40.566	3402.1	34.2	1.3802	76.616	0.475

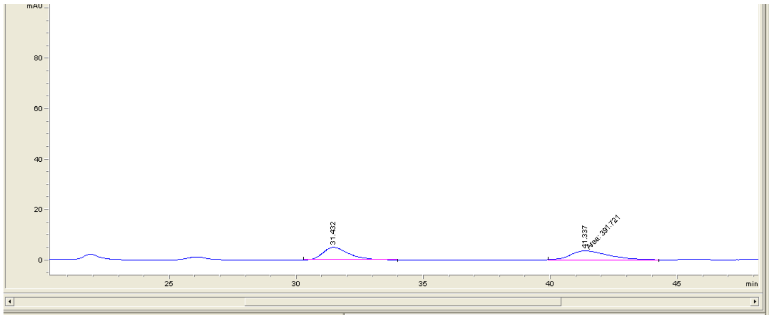

File Information

LC-File	RS-796C 20%.D
File Path	E:\CHEM32\1\DATA\RAKESH\RAKESH S 2021-01-12 16-42-39\
Date	12-Jan-21, 16:43:26
Sample	RS-796c 20%
Sample Info	80:20 hex:IPA IA
Barcode	
Operator	SYSTEM
Method	RAKESH 80-20 HEXANE-IPA1ML.M
Analysis Time	64.807 min
Sampling Rate	0.0067 min (0.402 sec), 9722 datapoints

#	Time	Area	Height	Width	Area%	Symmetry
1	31.432	358.9	5	0.8812	47.811	0.639
2	41.337	391.7	3.7	1.782	52.189	0.598

1H NMR

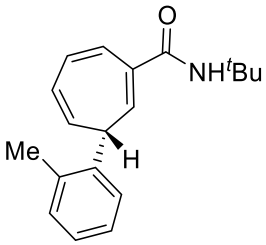

***(R)-N*-(tert-butyl)-3-(o-tolyl)cyclohepta-1,4,6-triene-1-carboxamide (4e)**

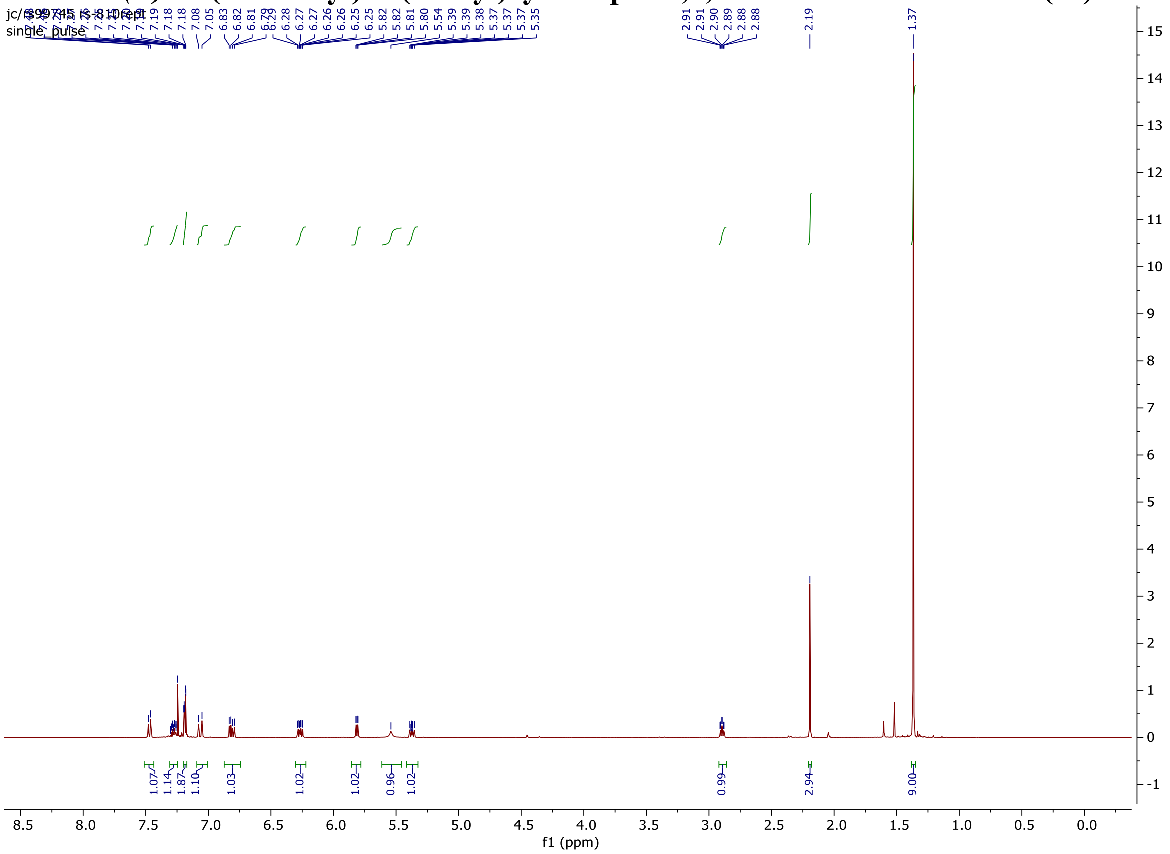

^{13}C NMR

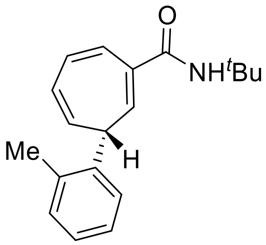

(R)-*N*-(tert-butyl)-3-(o-tolyl)cyclohepta-1,4,6-triene-1-carboxamide (4e)

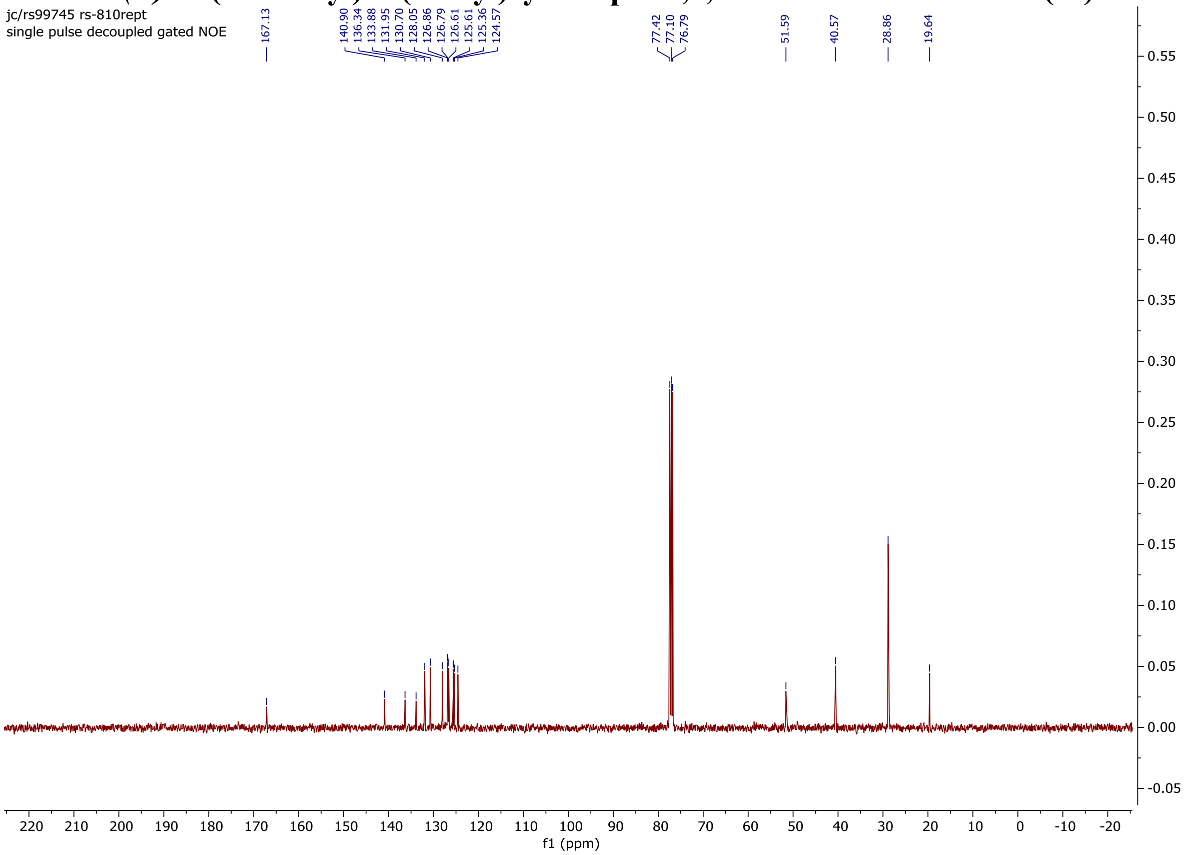

HPLC

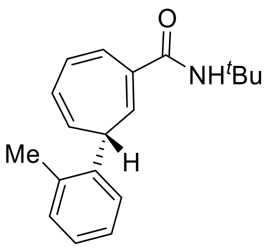

(R)-N-(tert-butyl)-3-(o-tolyl)cyclohepta-1,4,6-triene-1-carboxamide (4e)

Analytical HPLC (Chiral Regis Whelk O1), eluting with hexane- IPA (90:10), showed it to consist of a 20:80 mixture of two enantiomers with retention times 30.722 mins(minor) and 44.404 mins(major), respectively.

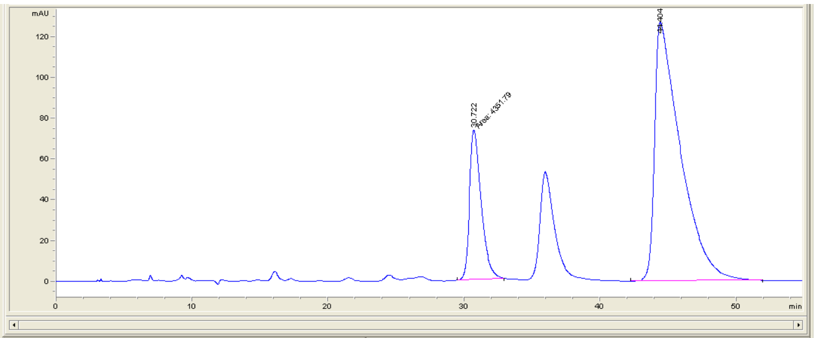

File Information

LC-File	RS-815.D
File Path	E:\CHEM32\1\DATA\RAKESH\RAKESH S 2021-01-27 17-38-14\
Date	27-Jan-21, 17:39:02
Sample	RS-815
Sample Info	90:10 hex:IPA IA
Barcode	
Operator	SYSTEM
Method	RAKESH 90-10 HEXANE-IPA1ML.M
Analysis Time	54.873 min
Sampling Rate	0.0067 min (0.402 sec), 8232 datapoints

#	Time	Area	Height	Width	Area%	Symmetry
1	30.722	4351.8	73.1	0.9916	20.453	0.615
2	44.404	16925.2	127.1	1.8077	79.547	0.262

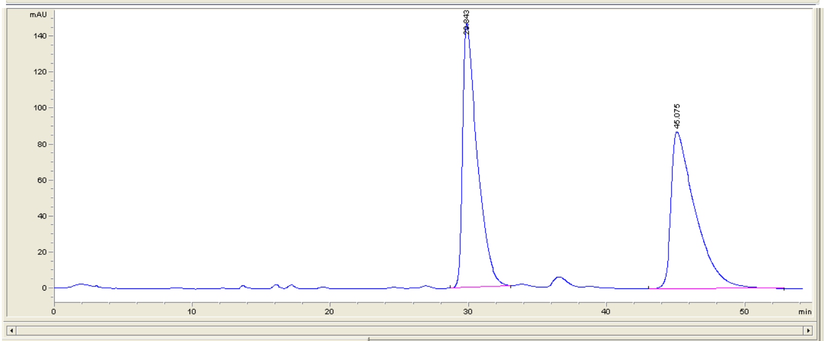

File Information

LC-File	RS-810.D
File Path	E:\CHEM32\1\DATA\RAKESH\RAKESH S 2021-01-27 18-35-46\
Date	27-Jan-21, 18:36:34
Sample	RS-810
Sample Info	90:10 hex:IPA IA
Barcode	
Operator	SYSTEM
Method	RAKESH 90-10 HEXANE-IPA1ML.M
Analysis Time	54.2 min
Sampling Rate	0.0067 min (0.402 sec), 8131 datapoints

#	Time	Area	Height	Width	Area%	Symmetry
1	29.843	10688.8	147	1.0481	50.221	0.348
2	45.075	10594.9	87.3	1.6642	49.779	0.304

1H NMR

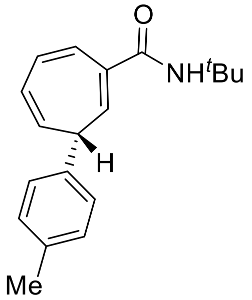

(R)-*N*-(tert-butyl)-3-(p-tolyl)cyclohepta-1,4,6-triene-1-carboxamide (4f)

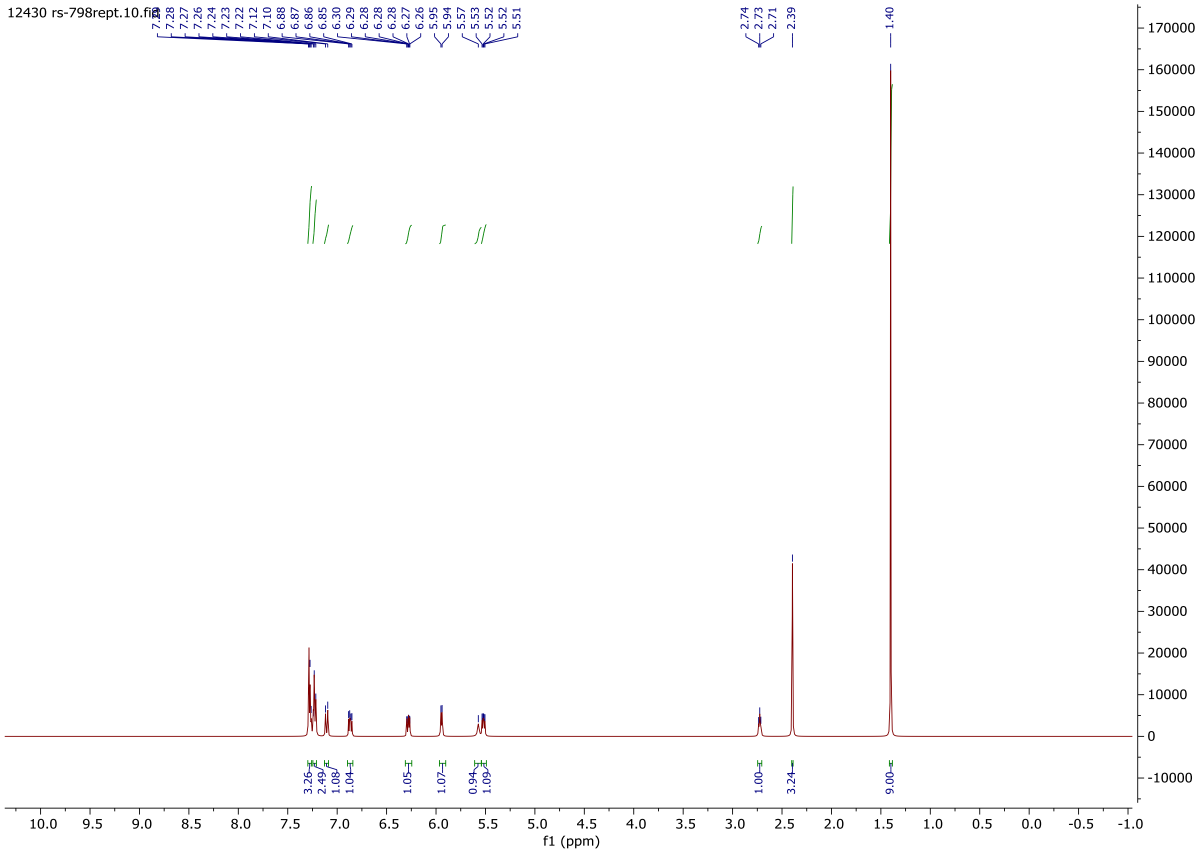

^{13}C NMR

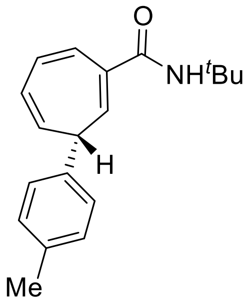

(R)-N-(tert-butyl)-3-(p-tolyl)cyclohepta-1,4,6-triene-1-carboxamide (4f)

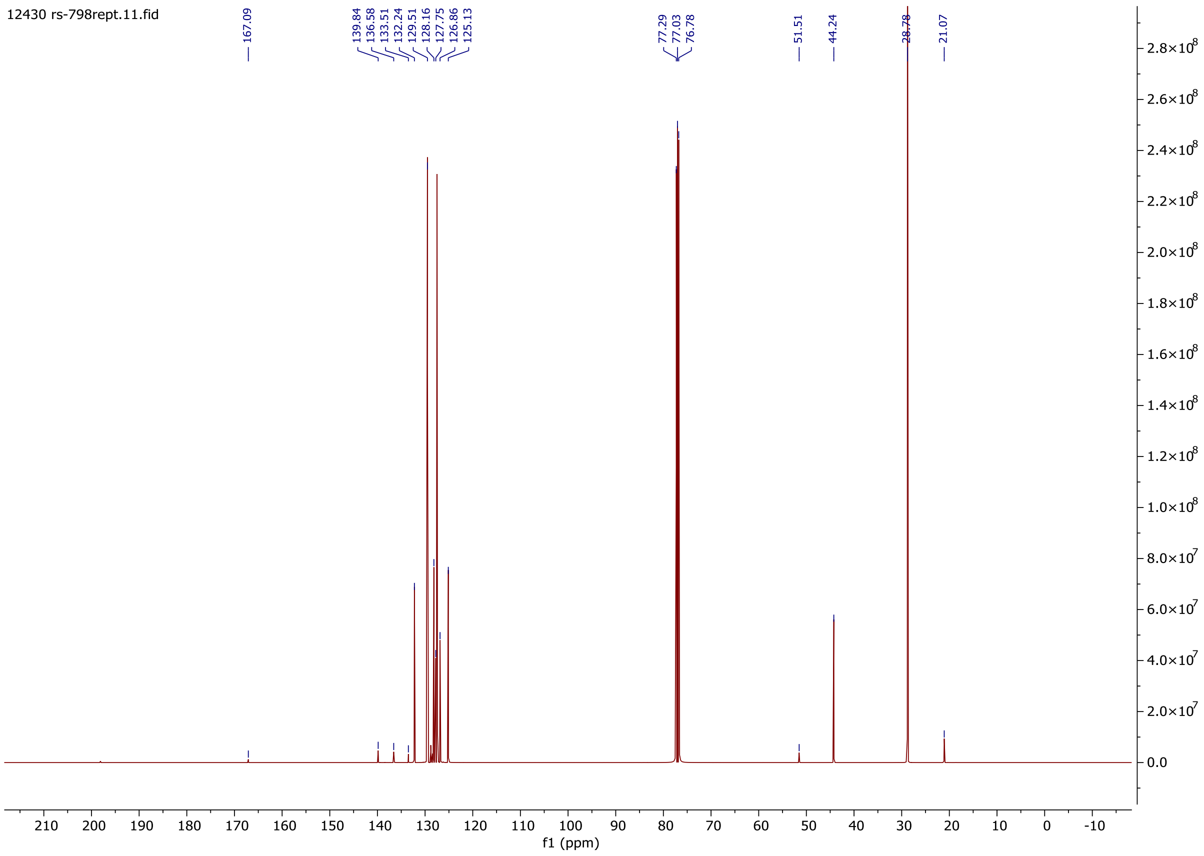

HPLC

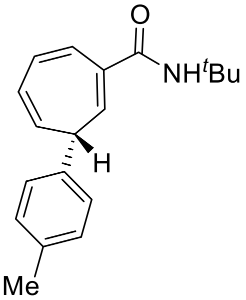

***(R)*-*N*-(tert-butyl)-3-(p-tolyl)cyclohepta-1,4,6-triene-1-carboxamide (4f)**

Analytical HPLC (IA Column), eluting with hexane- IPA (99:1), showed it to consist of a 4:96 mixture of two enantiomers with retention times 10.271 mins(minor) and 12.86 mins (major), respectively.

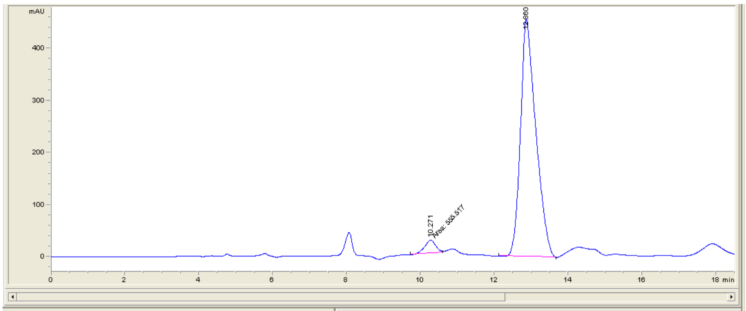

File Information

LC-File	RS-798 IA 1%.D
File Path	E:\CHEM32\1\DATA\RAKESH\RAKESH S 2021-01-21 15-10-50\
Date	21-Jan-21, 15:11:38
Sample	RS-798 IA 1%
Sample Info	99:1 hex:IPA IA
Barcode	
Operator	SYSTEM
Method	RAKESH 99-01 HEXANE-IPA1ML.M
Analysis Time	26.927 min
Sampling Rate	0.0067 min (0.402 sec), 4040 datapoints

#	Time	Area	Height	Width	Area%	Symmetry
1	10.271	555.5	25.8	0.3587	4.057	1.141
2	12.86	13137.3	456.1	0.4101	95.943	0.571

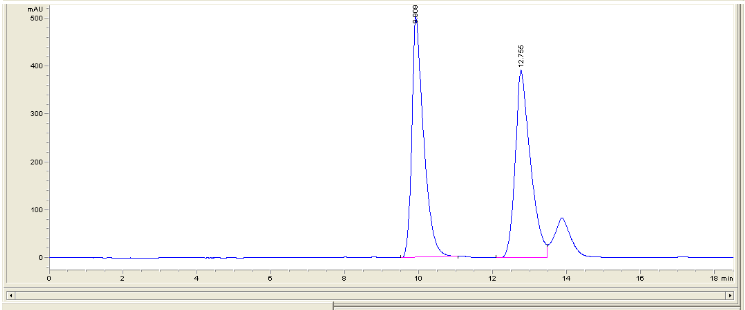

File Information

LC-File	RS-809 IA 1%.D
File Path	E:\CHEM32\1\DATA\RAKESH\RAKESH S 2021-01-21 17-29-37\
Date	21-Jan-21, 17:30:25
Sample	RS-809 IA 1%
Sample Info	99:1 hex:IPA IA
Barcode	
Operator	SYSTEM
Method	RAKESH 99-01 HEXANE-IPA1ML.M
Analysis Time	18.513 min
Sampling Rate	0.0067 min (0.402 sec), 2778 datapoints

#	Time	Area	Height	Width	Area%	Symmetry
1	9.909	11101.2	502.5	0.3138	49.745	0.474
2	12.755	11215.2	392	0.4018	50.255	0.567

^{1}H NMR

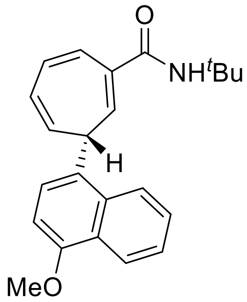

***(R)*-*N*-(tert-butyl)-3-(4-methoxynaphthalen-1-yl)cyclohepta-1,4,6-triene-1-carboxamide (4g)**

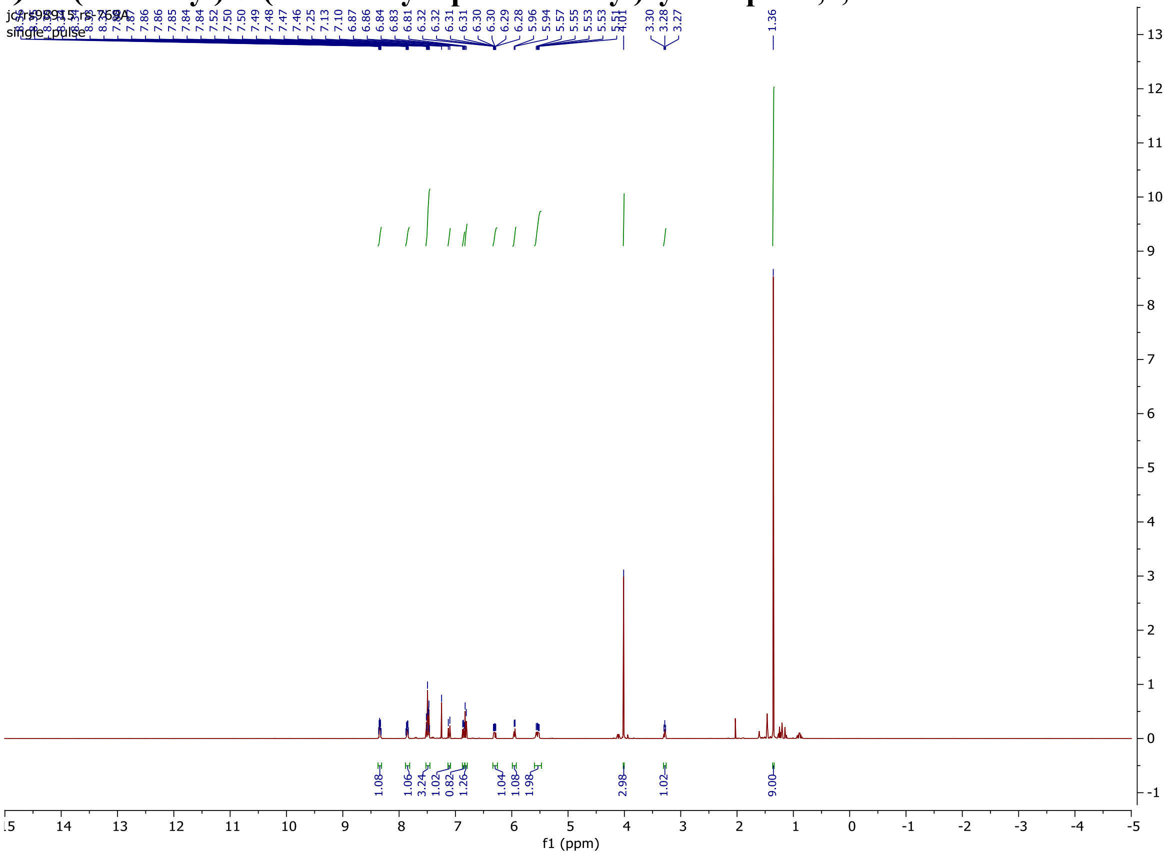

^{13}C NMR

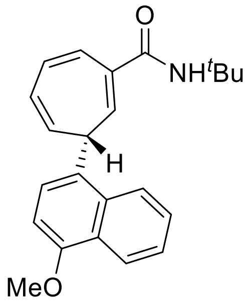

(R)-N-(tert-butyl)-3-(4-methoxynaphthalen-1-yl)cyclohepta-1,4,6-triene-1-carboxamide (4g)

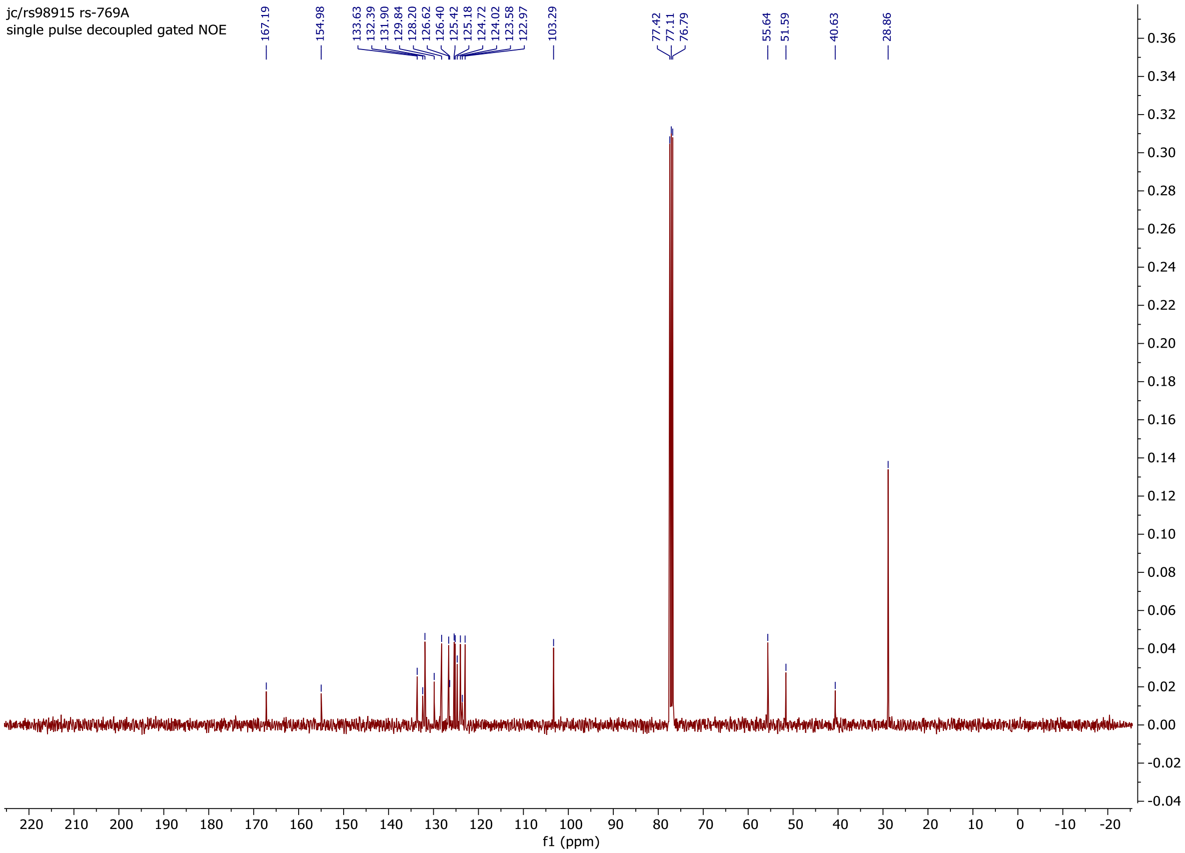

HPLC

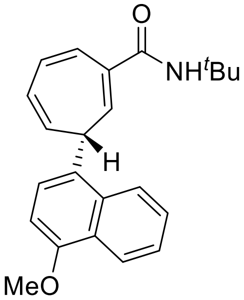

***(R)*-*N*-(tert-butyl)-3-(4-methoxynaphthalen-1-yl)cyclohepta-1,4,6-triene-1-carboxamide (4g)**

Analytical HPLC (Chiral Regis Whelk O1), eluting with hexane-IPA (15:85), showed it to consist of a 5.79:94.210 mixture of two enantiomers with retention times 32.273 mins (minor) and 40.017 mins (major).

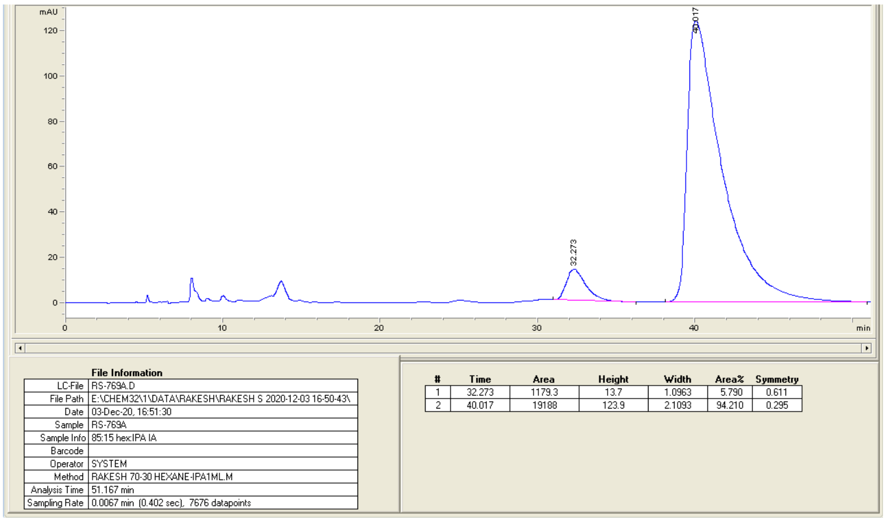

File Information

LC-File	RS-769A.D
File Path	E:\CHEM32\1\DATA\RAKESH\RAKESH S 2020-12-03 16-50-43\
Date	03-Dec-20, 16:51:30
Sample	RS-769A
Sample Info	85:15 hex:IPA IA
Barcode	
Operator	SYSTEM
Method	RAKESH 70-30 HEXANE-IPA1ML.M
Analysis Time	51.167 min
Sampling Rate	0.0067 min (0.402 sec), 7676 datapoints

#	Time	Area	Height	Width	Area%	Symmetry
1	32.273	1179.3	13.7	1.0963	5.790	0.611
2	40.017	19188	123.9	2.1093	94.210	0.295

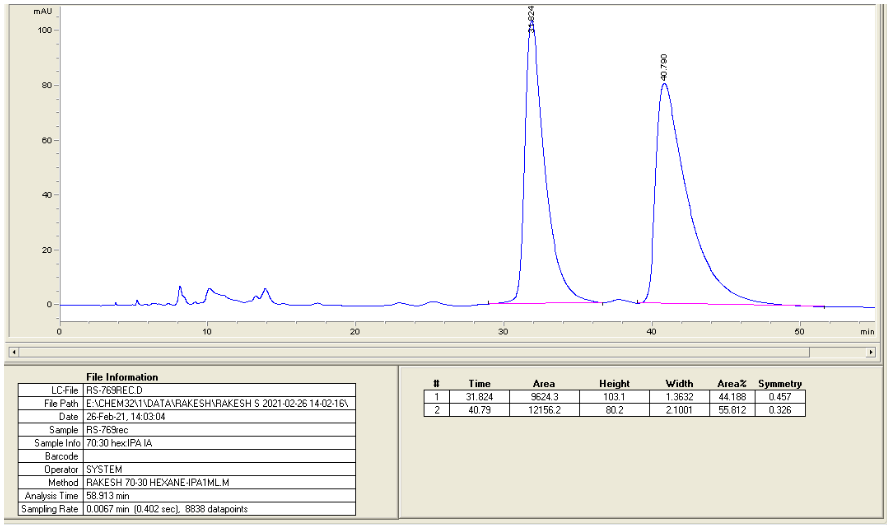

File Information

LC-File	RS-769REC.D
File Path	E:\CHEM32\1\DATA\RAKESH\RAKESH S 2021-02-26 14-02-16\
Date	26-Feb-21, 14:03:04
Sample	RS-769rec
Sample Info	70:30 hex:IPA IA
Barcode	
Operator	SYSTEM
Method	RAKESH 70-30 HEXANE-IPA1ML.M
Analysis Time	58.913 min
Sampling Rate	0.0067 min (0.402 sec), 8838 datapoints

#	Time	Area	Height	Width	Area%	Symmetry
1	31.824	9624.3	103.1	1.3632	44.188	0.457
2	40.79	12156.2	80.2	2.1001	55.812	0.326

^{1}H NMR

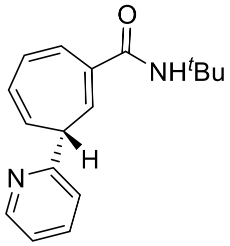

***(R)-N-(tert-butyl)-3-(pyridin-2-yl)cyclohepta-1,4,6-triene-1-carboxamide* (4h)**

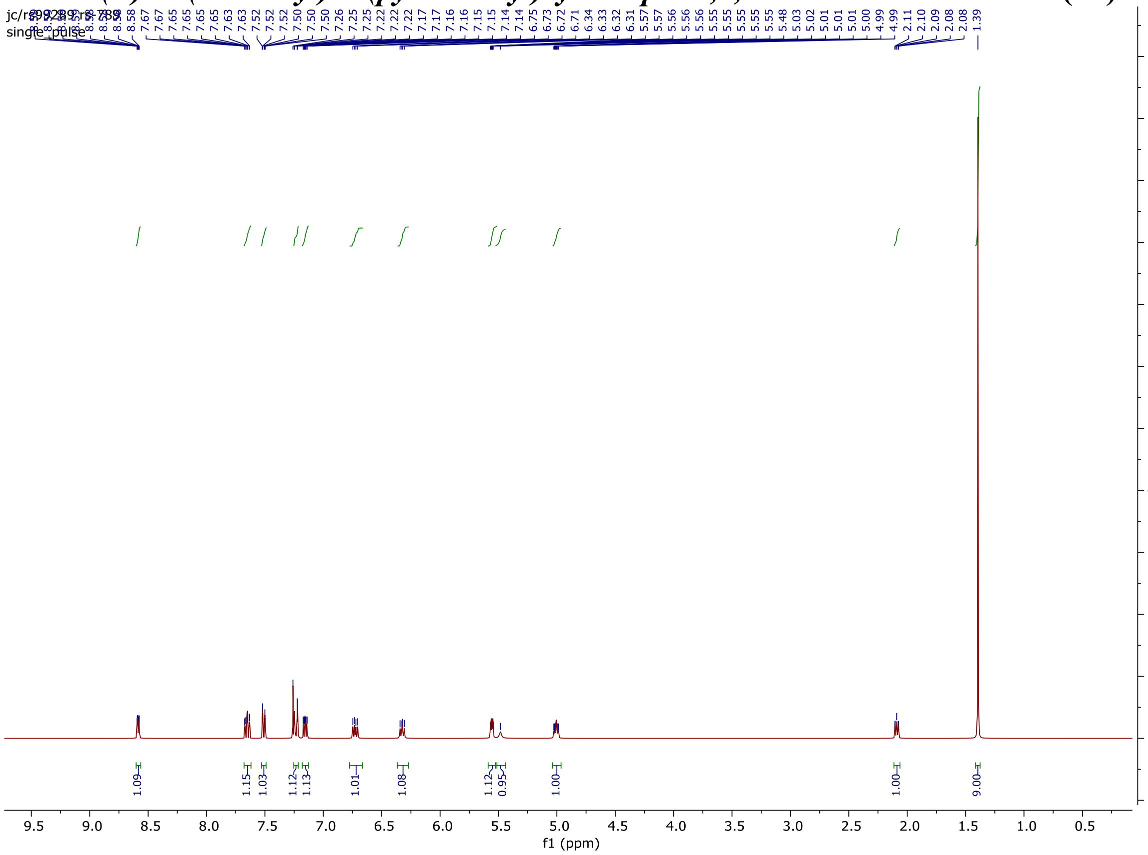

^{13}C NMR

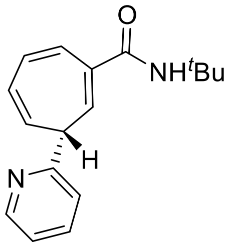

(R)-N-(tert-butyl)-3-(pyridin-2-yl)cyclohepta-1,4,6-triene-1-carboxamide **(4h)**

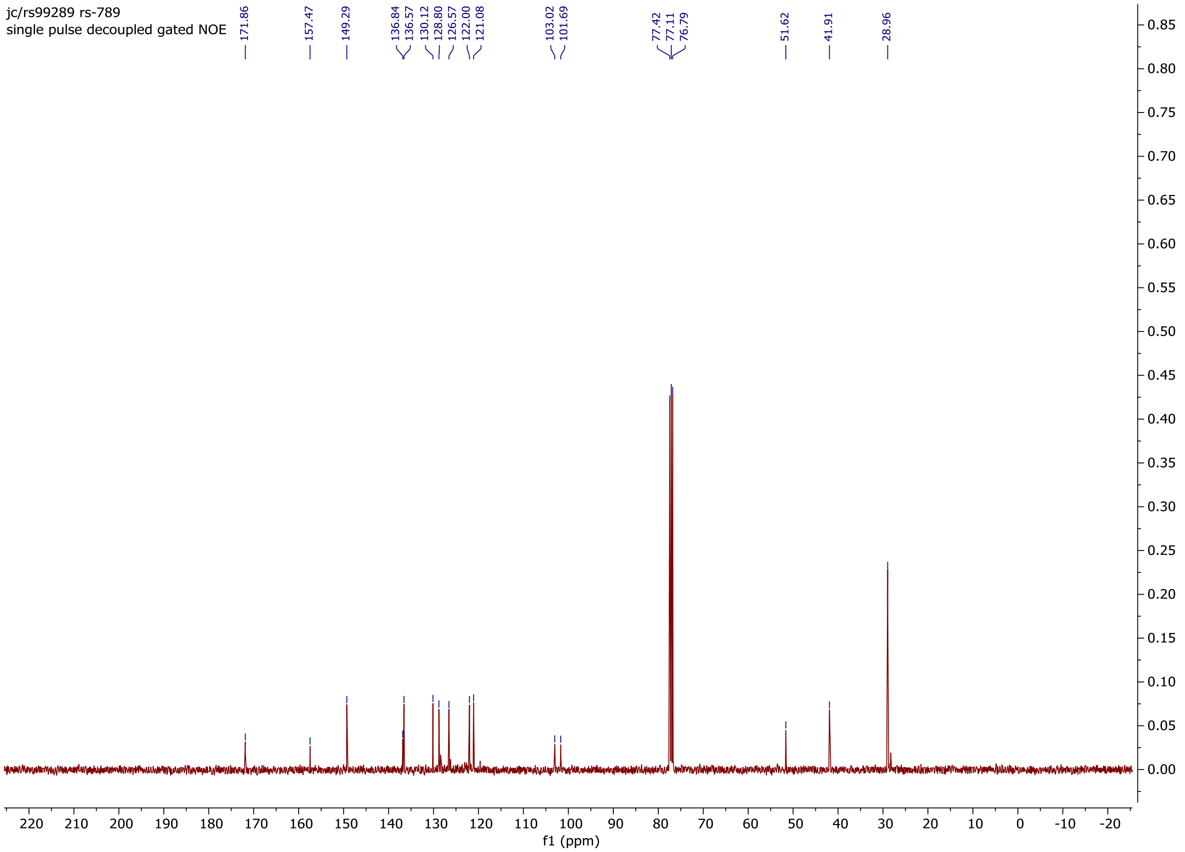

HPLC

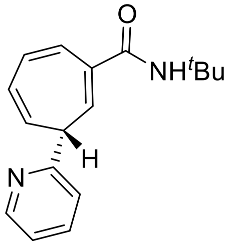

(R)-N-(tert-butyl)-3-(pyridin-2-yl)cyclohepta-1,4,6-triene-1-carboxamide **(4h)**

Analytical HPLC (Chiral Regis Whelk O1), eluting with IPA–hexane (85:15), showed it to consist of a 39:61 mixture of two enantiomers with retention times 20.987 mins(minor) and 22.505 mins (major), respectively.

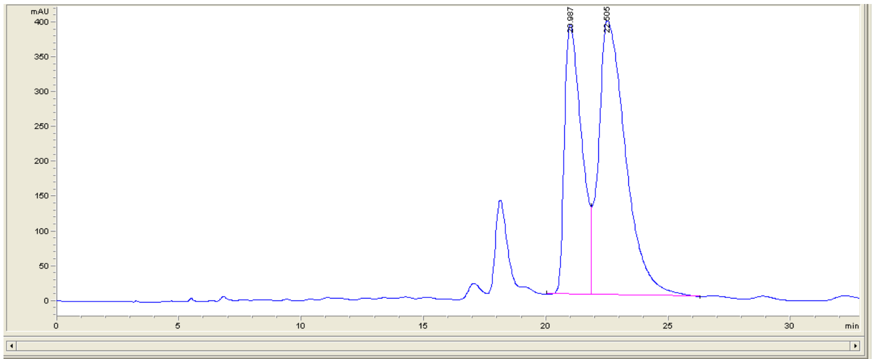

File Information

LC-File	RS-789.D
File Path	E:\CHEM32\1\DATA\RAKESH\RAKESH S 2021-01-11 17-41-22\
Date	11-Jan-21, 17:42:09
Sample	RS-789
Sample Info	85:15 hex:IPA IA
Barcode	
Operator	SYSTEM
Method	RAKESH 85-15 HEXANE-IPA1ML.M
Analysis Time	32.82 min
Sampling Rate	0.0067 min (0.402 sec), 4924 datapoints

#	Time	Area	Height	Width	Area%	Symmetry
1	20.987	19095.3	387.6	0.7407	38.597	0.48
2	22.505	30378.1	394	1.1695	61.403	0.458

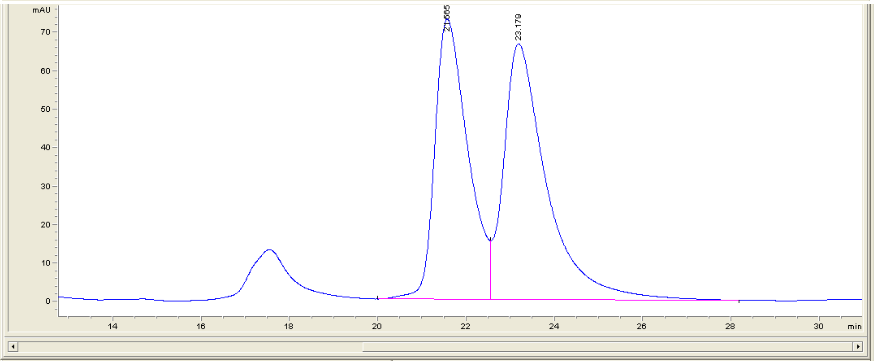

File Information

LC-File	RS-782 15%.D
File Path	E:\CHEM32\1\DATA\RAKESH\RAKESH S 2020-12-18 14-35-35\
Date	18-Dec-20, 14:36:23
Sample	RS-782 15%
Sample Info	70:30 hex:IPA IA
Barcode	
Operator	SYSTEM
Method	RAKESH 85-15 HEXANE-IPA1ML.M
Analysis Time	30.967 min
Sampling Rate	0.0067 min (0.402 sec), 4646 datapoints

#	Time	Area	Height	Width	Area%	Symmetry
1	21.565	3802.6	72.8	0.785	45.755	0.585
2	23.179	4508.1	66.5	0.9768	54.245	0.48

^{1}H NMR

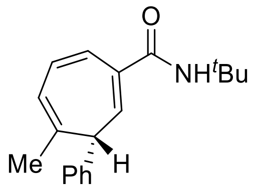

(*S*)-*N*-(tert-butyl)-4-methyl-3-phenylcyclohepta-1,4,6-triene-1-carboxamide (4i)

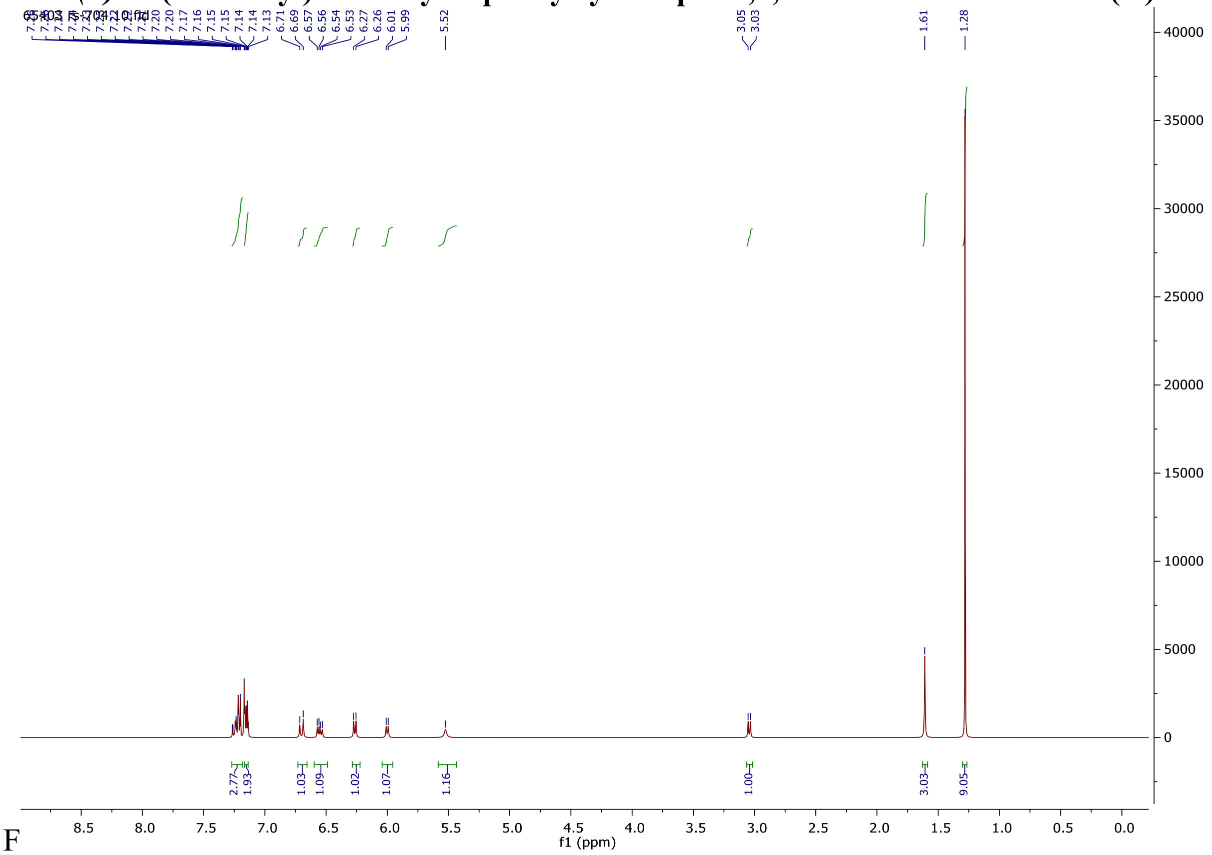

F

^{13}C NMR

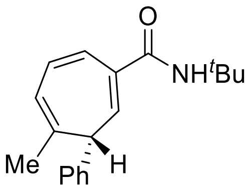

(S)-N-(tert-butyl)-4-methyl-3-phenylcyclohepta-1,4,6-triene-1-carboxamide (4i)

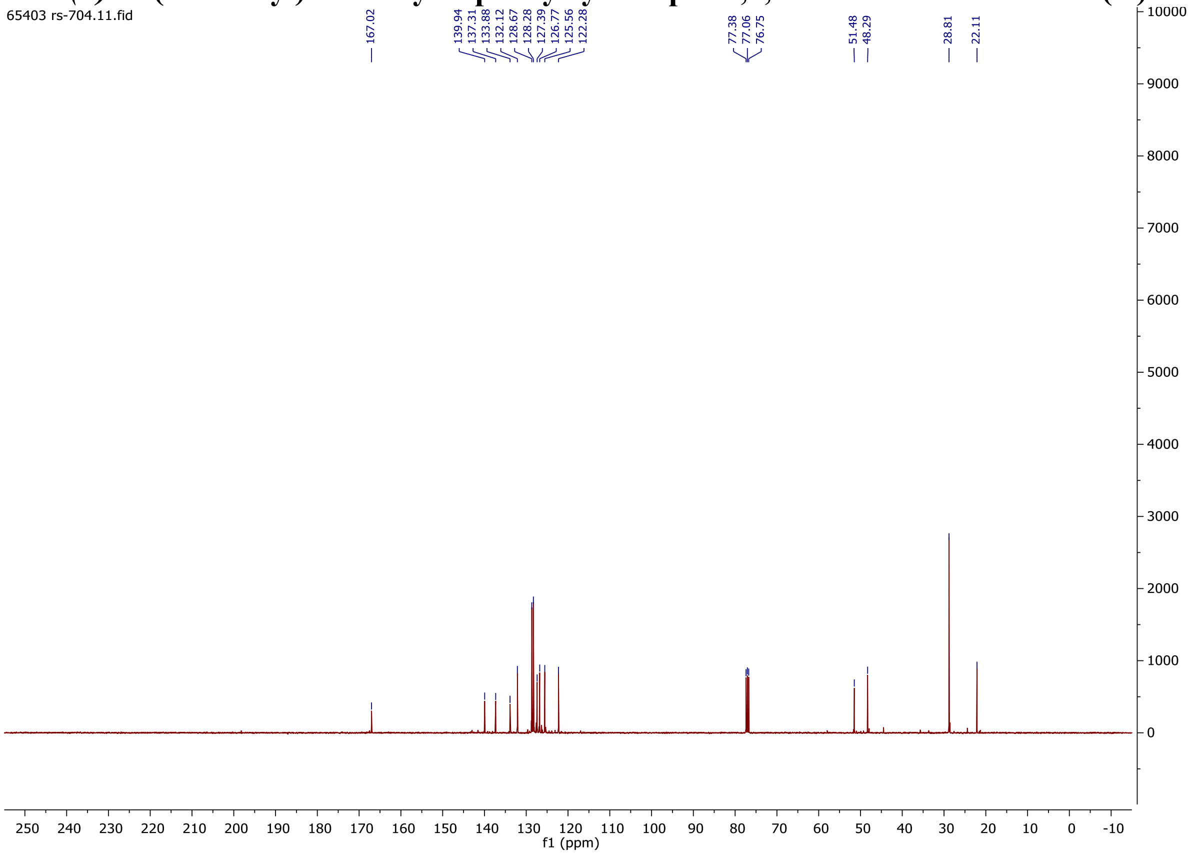

HPLC

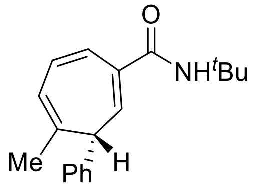

***(S)*-*N*-(tert-butyl)-4-methyl-3-phenylcyclohepta-1,4,6-triene-1-carboxamide (4i)**

Analytical HPLC (Chiral Regis Whelk O1), eluting with hexane- IPA (70:30), showed it to consist of a 5.3: 94:6 mixture of two enantiomers with retention times 21.53 mins (minor) and 29.20 mins (major) respectively.

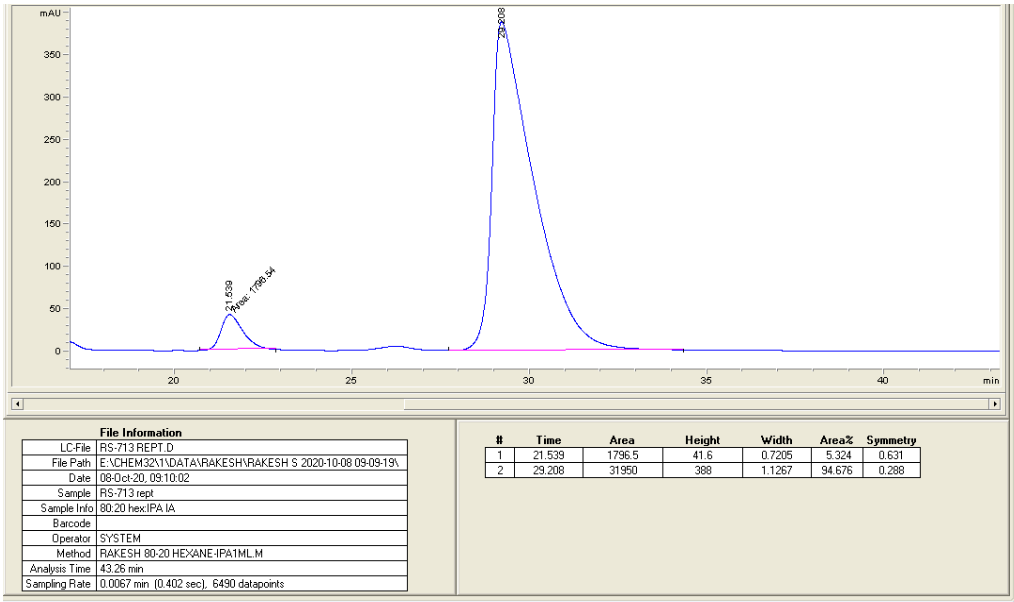

LC-File	RS-713 REPT.D
File Path	E:\CHEM32\1\DATA\RAKESH\RAKESH S 2020-10-08 09-09-19\
Date	08-Oct-20, 09:10:02
Sample	RS-713 rept
Sample Info	80:20 hex:IPA IA
Barcode	
Operator	SYSTEM
Method	RAKESH 80-20 HEXANE-IPA1ML.M
Analysis Time	43.26 min
Sampling Rate	0.0067 min (0.402 sec), 6490 datapoints

#	Time	Area	Height	Width	Area%	Symmetry
1	21.539	1796.5	41.6	0.7205	5.324	0.631
2	29.208	31950	388	1.1267	94.676	0.288

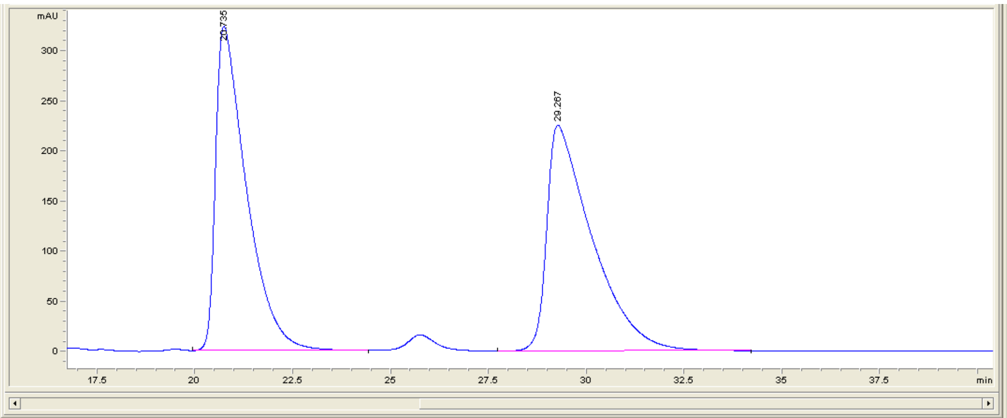

File Information

LC-File	RS-704 REPT.D
File Path	E:\CHEM32\1\DATA\RAKESH\RAKESH S 2020-10-08 09-57-44\
Date	08-Oct-20, 09:58:30
Sample	RS-704 rept
Sample Info	80:20 hex:IPA IA
Barcode	
Operator	SYSTEM
Method	RAKESH 80-20 HEXANE-IPA1ML.M
Analysis Time	40.393 min
Sampling Rate	0.0067 min (0.402 sec), 6060 datapoints

#	Time	Area	Height	Width	Area%	Symmetry
1	20.735	17724.4	323.3	0.8177	49.443	0.344
2	29.267	18124	225.1	1.1574	50.557	0.305

^{1}H NMR

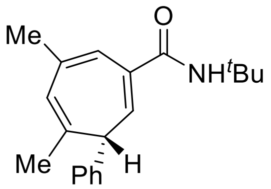

***(S)*-*N*-(tert-butyl)-4,6-dimethyl-3-phenylcyclohepta-1,4,6-triene-1-carboxamide (4j)**

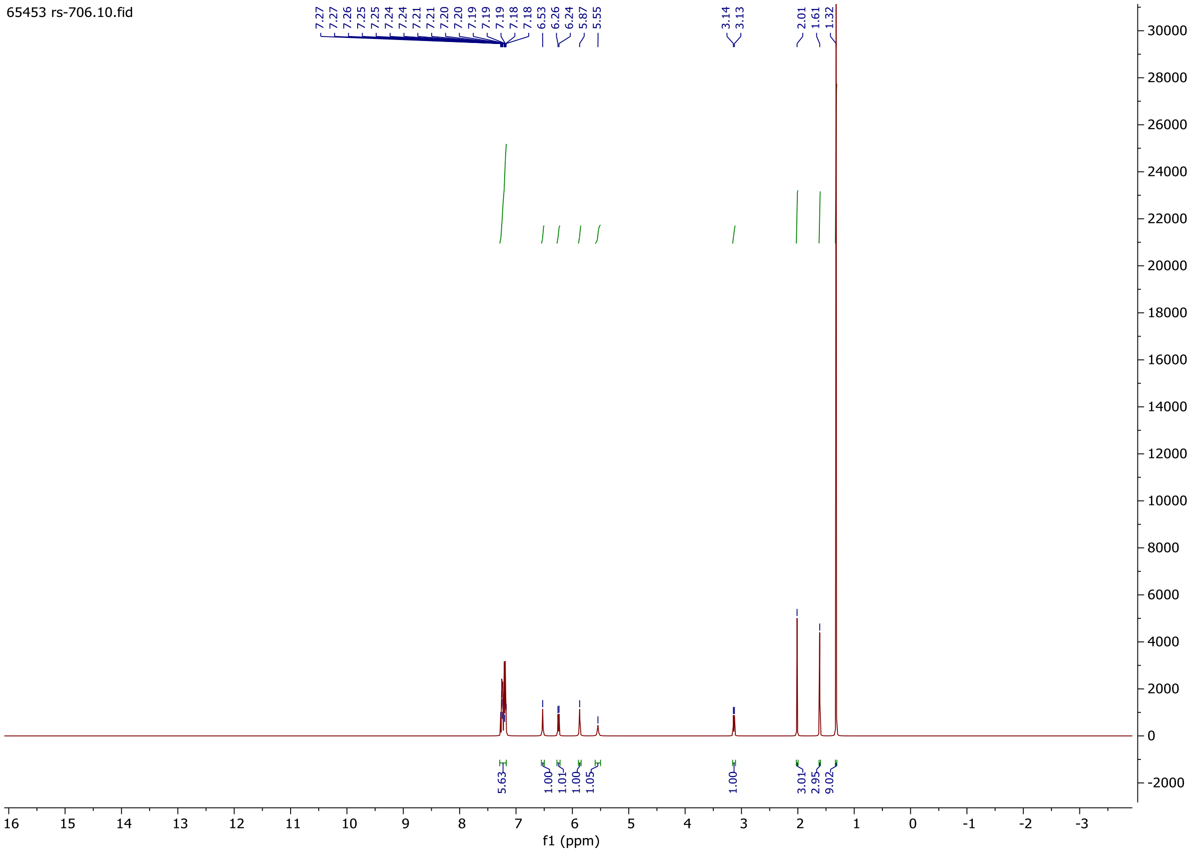
65453 rs-706.10.fid
7.27
7.27
7.26
7.25
7.25
7.24
7.24
7.21
7.21
7.20
7.20
7.19
7.19
7.19
7.18
6.53
6.26
6.24
5.87
5.55
3.14
3.13
2.01
1.61
1.32
5.63
1.00
1.01
1.00
1.05
1.00
3.01
2.95
9.02
f1 (ppm)

^{13}C NMR

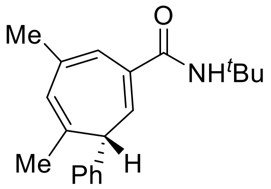

(S)-N-(tert-butyl)-4,6-dimethyl-3-phenylcyclohepta-1,4,6-triene-1-carboxamide (4j)

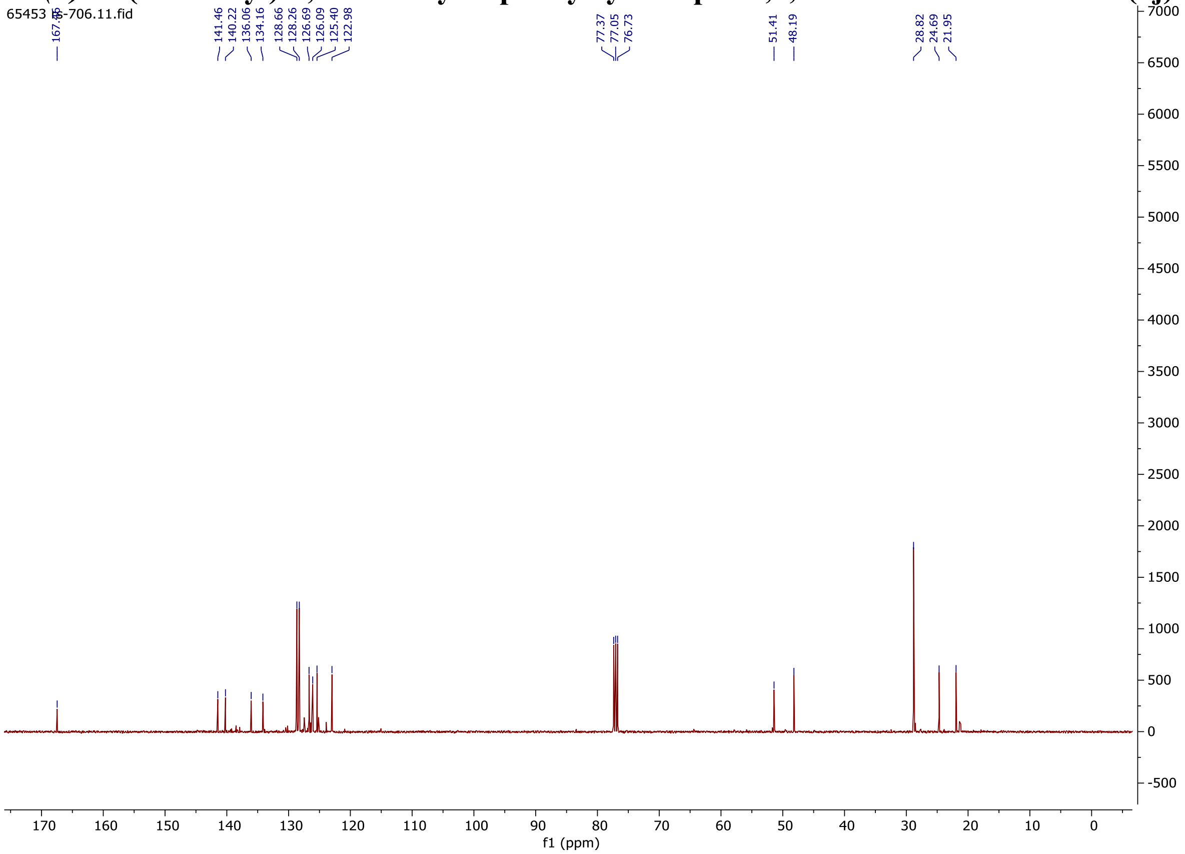

HPLC

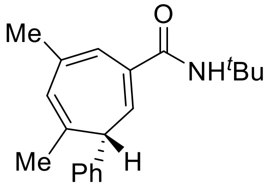

***(S)*-*N*-(tert-butyl)-4,6-dimethyl-3-phenylcyclohepta-1,4,6-triene-1-carboxamide (4j)**

Analytical HPLC (Chiral Regis Whelk O1), eluting with hexane-IPA (70:30), showed it to consist of a 29.00: 70.99 mixture of two enantiomers with retention times tR = 10.41 and 11.99 respectively.

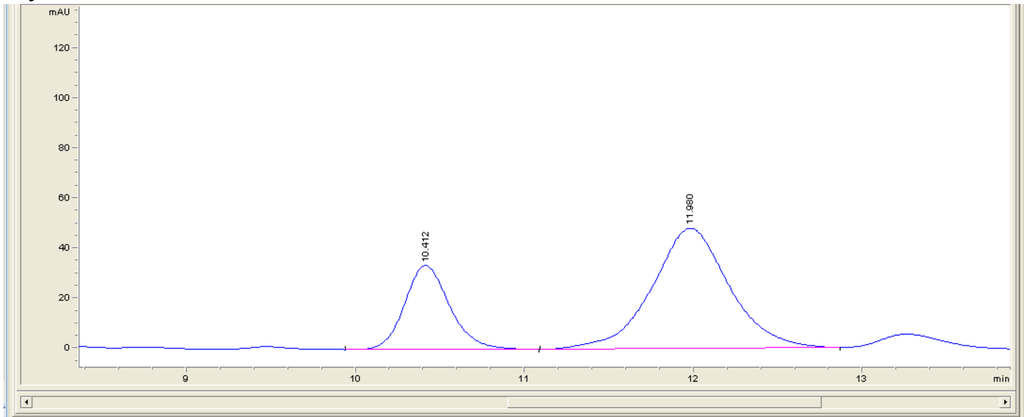

File Information

LC-File	RS-720.D
File Path	E:\CHEM32\1\DATA\RAKESH\RAKESH S 2020-11-09 17-17-11\
Date	09-Nov-20, 17:17:58
Sample	RS-720
Sample Info	70:30 hex:IPA IA
Barcode	
Operator	SYSTEM
Method	RAKESH 70-30 HEXANE-IPA1ML.M
Analysis Time	17 min
Sampling Rate	0.0067 min (0.402 sec), 2551 datapoints

#	Time	Area	Height	Width	Area%	Symmetry
1	10.412	622.1	33.6	0.2823	29.009	0.813
2	11.98	1522.5	48	0.4678	70.991	0.956

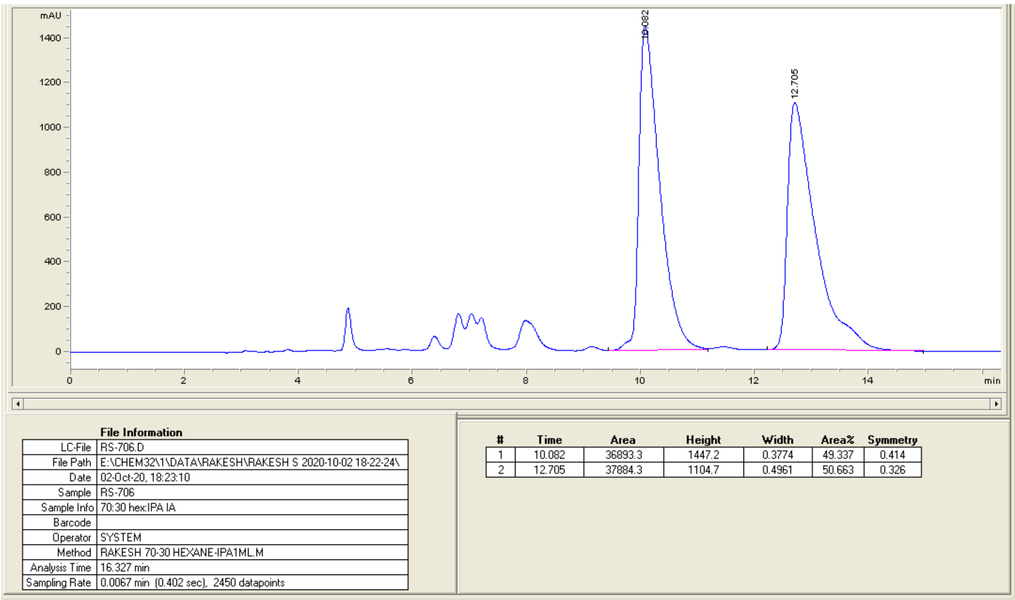

File Information

LC-File	RS-706.D
File Path	E:\CHEM32\1\DATA\RAKESH\RAKESH S 2020-10-02 18-22-24\
Date	02-Oct-20, 18:23:10
Sample	RS-706
Sample Info	70:30 hex:IPA IA
Barcode	
Operator	SYSTEM
Method	RAKESH 70-30 HEXANE-IPA1ML.M
Analysis Time	16.327 min
Sampling Rate	0.0067 min (0.402 sec), 2450 datapoints

#	Time	Area	Height	Width	Area%	Symmetry
1	10.082	36893.3	1447.2	0.3774	49.337	0.414
2	12.705	37884.3	1104.7	0.4961	50.663	0.326

^{1}H NMR

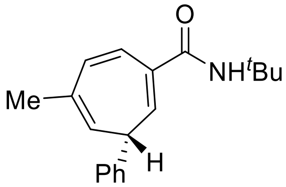

(*R*)-*N*-(tert-butyl)-5-methyl-3-phenylcyclohepta-1,4,6-triene-1-carboxamide (4k)

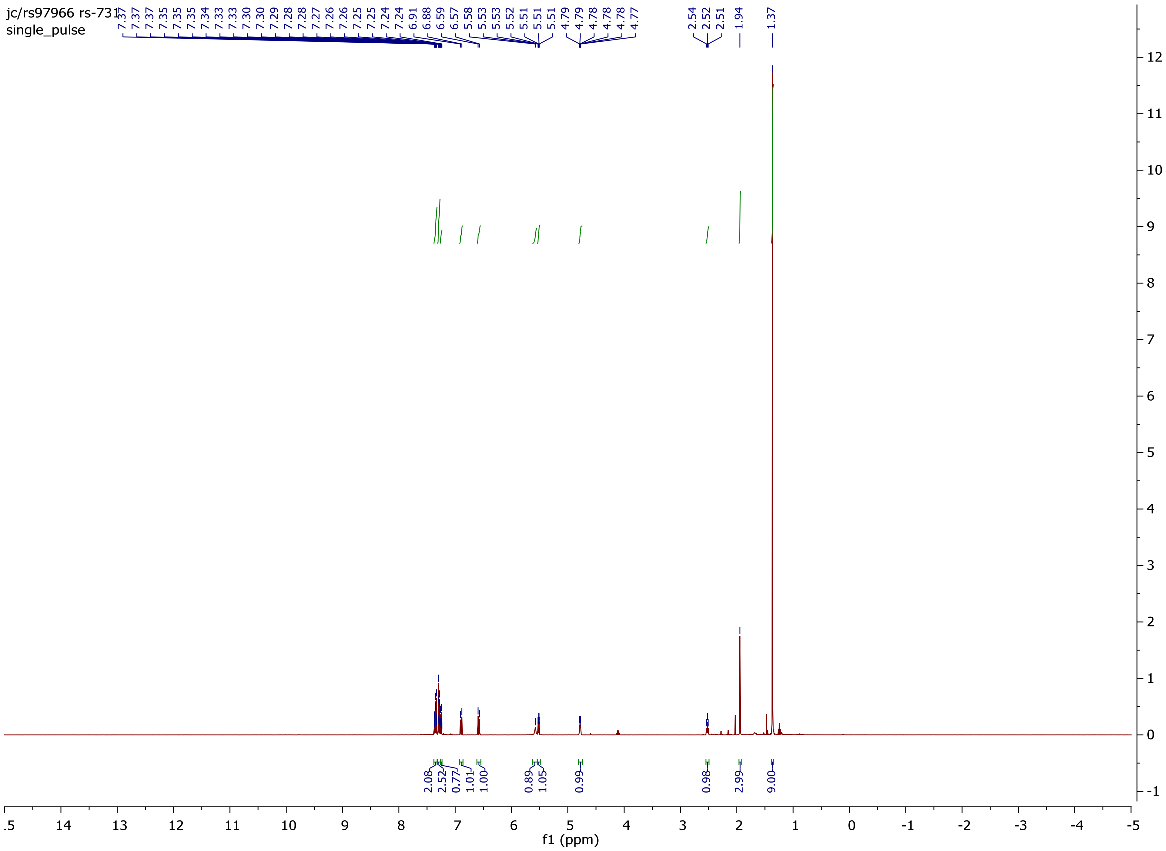

^{13}C NMR

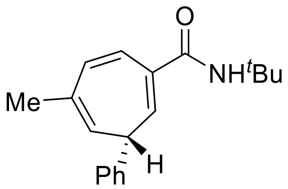

(R)-*N*-(tert-butyl)-5-methyl-3-phenylcyclohepta-1,4,6-triene-1-carboxamide (4k)

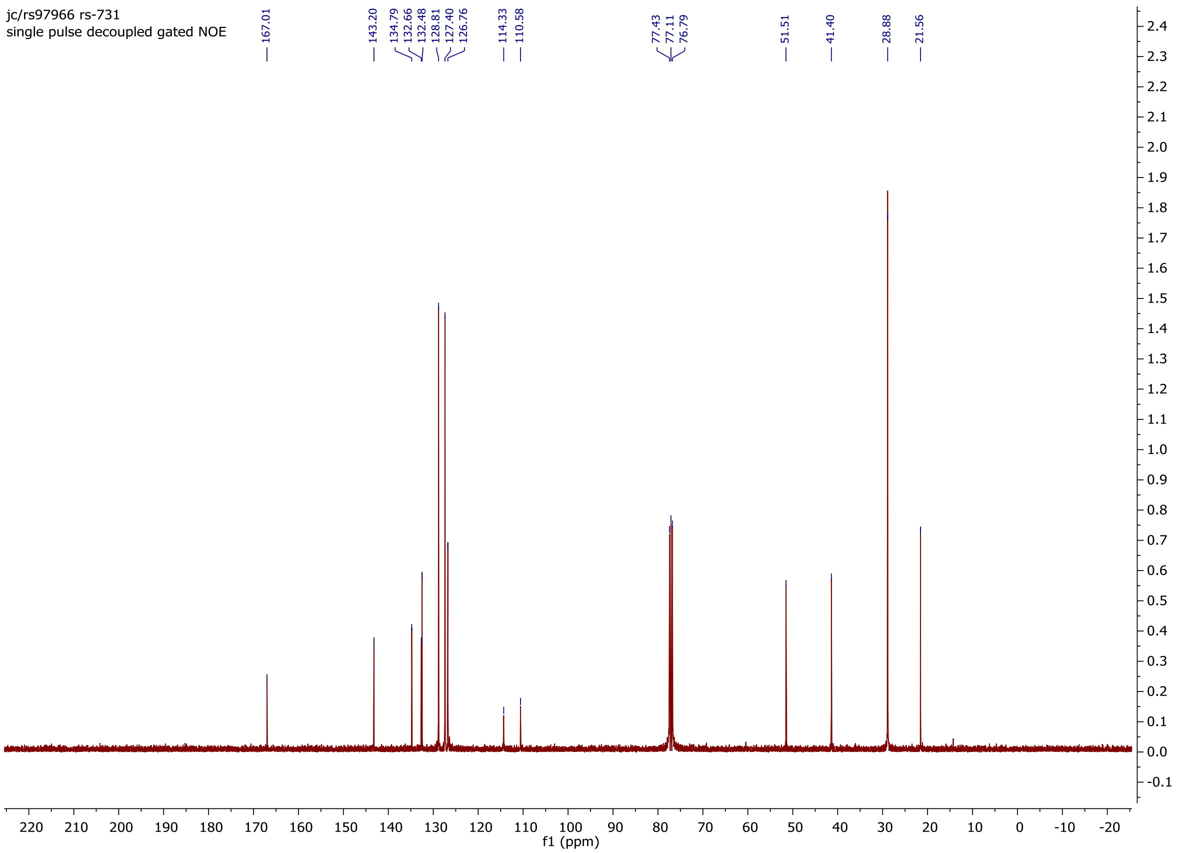

HPLC

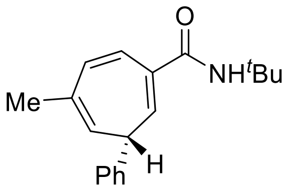

***(R)*-*N*-(tert-butyl)-5-methyl-3-phenylcyclohepta-1,4,6-triene-1-carboxamide (4k)**

Condition 1: Analytical HPLC (Chiral Regis Whelk O1), eluting with hexane-IPA (70:30), showed it to consist of a 12:88 mixture of two enantiomers with retention times 10.44 mins (minor) and 16.60 min (major) respectively.

Condition 2: Analytical HPLC (Chiral Regis Whelk O1), eluting with hexane-IPA (70:30), showed it to consist of a 20:80 mixture of two enantiomers with retention times 9.88 mins (minor) and 16.17 min (major) respectively.

Condition 1:

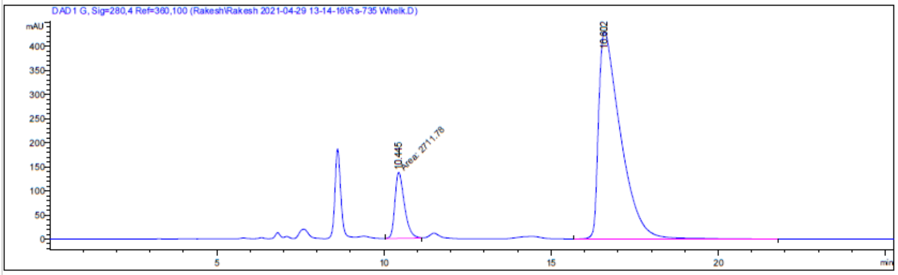

Signal 1: DAD1 G, Sig=280,4 Ref=360,100

Peak #	RetTime [min]	Type	Width [min]	Area [mAU*s]	Height [mAU]	Area %
1	10.445	MM	0.3299	2711.78003	137.00198	12.1189
2	16.602	BB	0.6740	1.96646e4	429.59396	87.8811

Totals : 2.23764e4 566.59595

Condition 2:

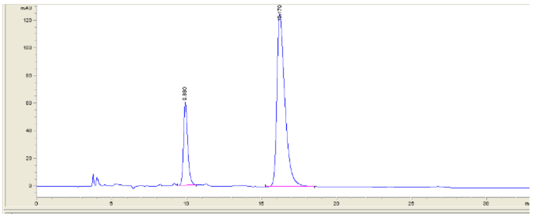

File Information

LC-File	RS-731.D
File Path	E:\CHEM32\1\DATA\RAKESH\RAKESH S 2020-11-03 10-33-47\
Date	03-Nov-20, 10:34:28
Sample	RS-731
Sample Info	70:30 hex:IPA IA
Barcode	
Operator	SYSTEM
Method	RAKESH 70-30 HEXANE-IPA1ML.M
Analysis Time	33.16 min
Sampling Rate	0.0067 min (0.402 sec), 4975 datapoints

#	Time	Area	Height	Width	Area%	Symmetry
1	9.88	1150.2	60.1	0.2914	19.979	0.714
2	16.179	4607	124.9	0.5482	80.021	0.517

rec-

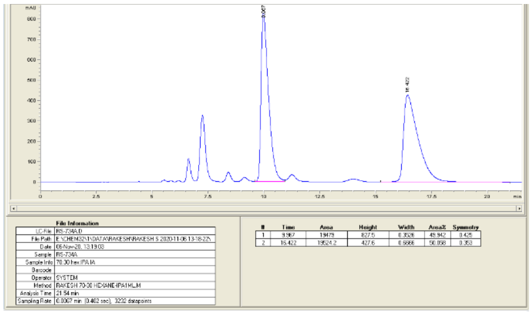

1H NMR

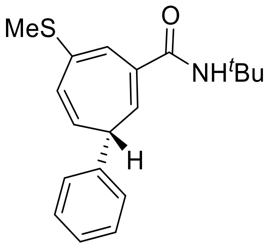

(R)-*N*-(tert-butyl)-6-(methylthio)-3-phenylcyclohepta-1,4,6-triene-1-carboxamide (4l)

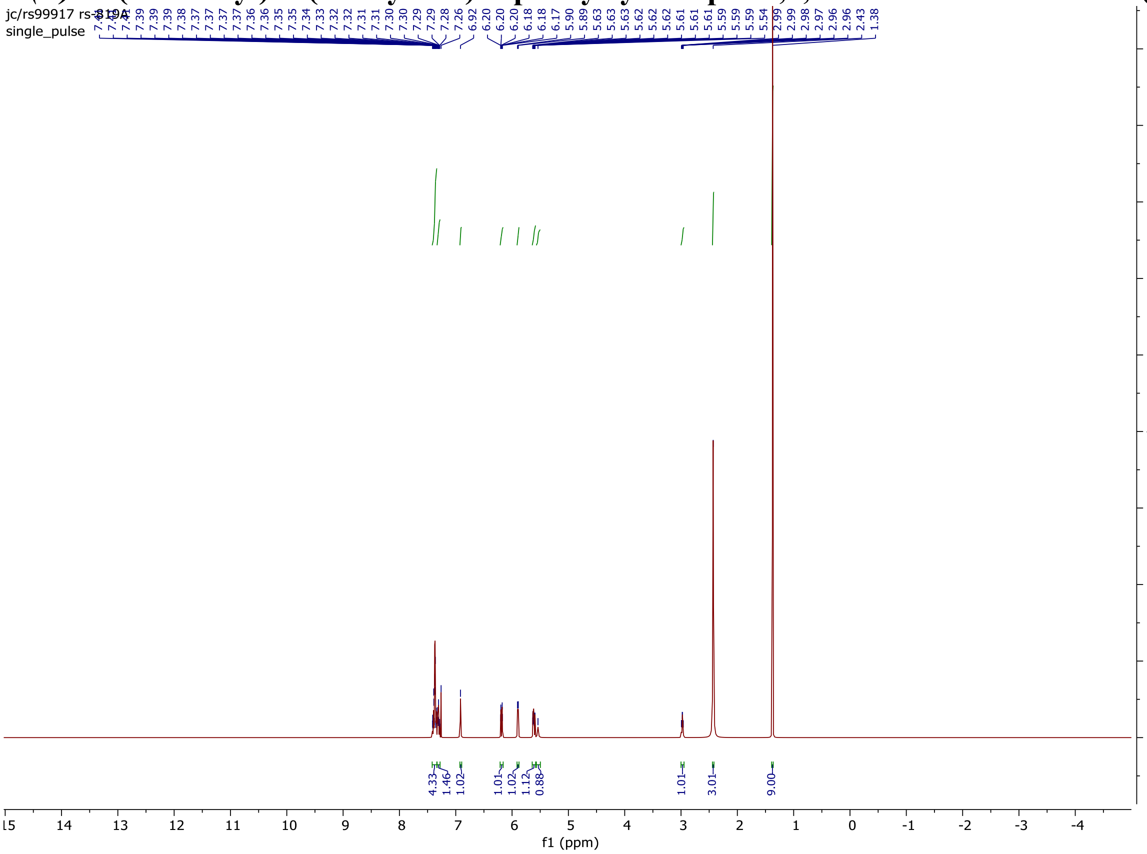

^{13}C NMR

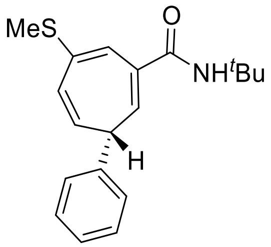

(R)-N-(tert-butyl)-6-(methylthio)-3-phenylcyclohepta-1,4,6-triene-1-carboxamide (4l)

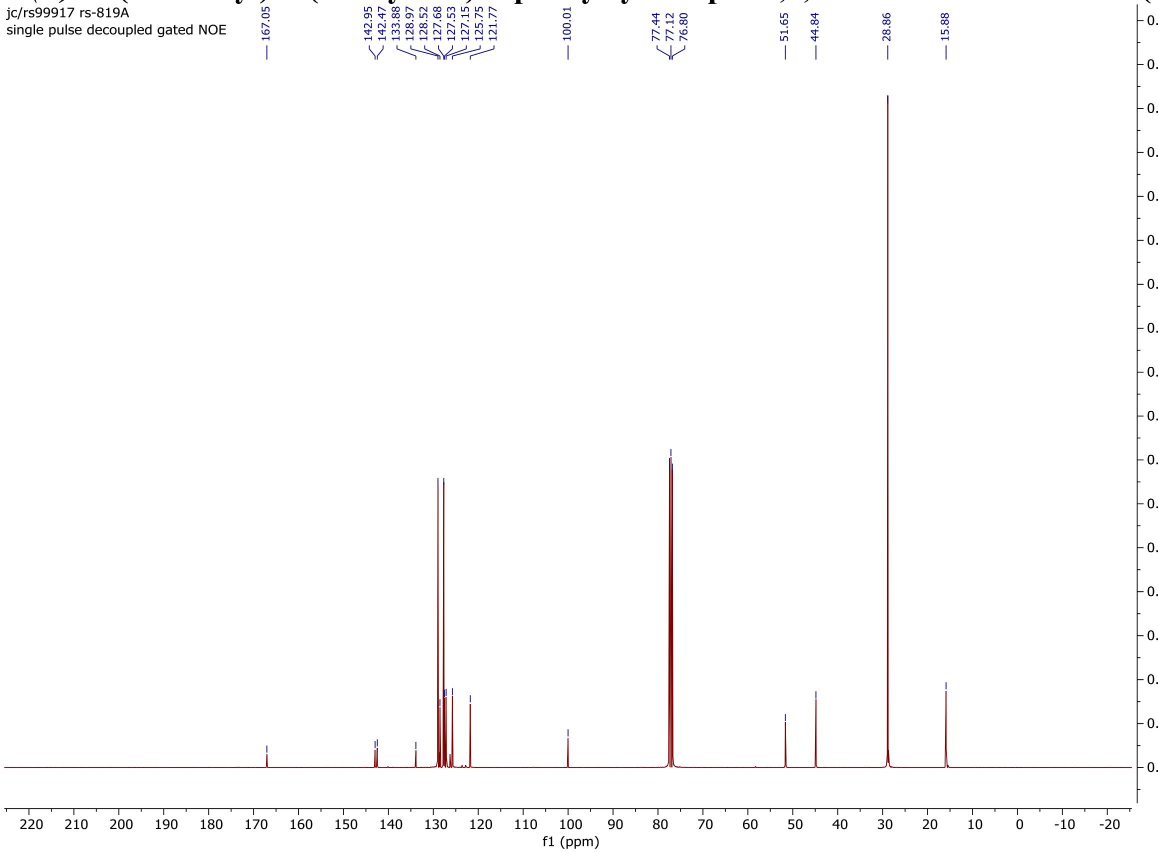

HPLC

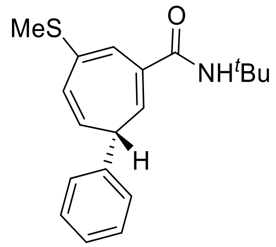

***(R)*-*N*-(tert-butyl)-6-(methylthio)-3-phenylcyclohepta-1,4,6-triene-1-carboxamide (4l)**

Analytical HPLC (IA Column), eluting with hexane- IPA (99:5), showed it to consist of a 1:99 mixture of two enantiomers with retention times 7.68 mins(minor) and 8.982 mins (major), flow rate 1mL/min.

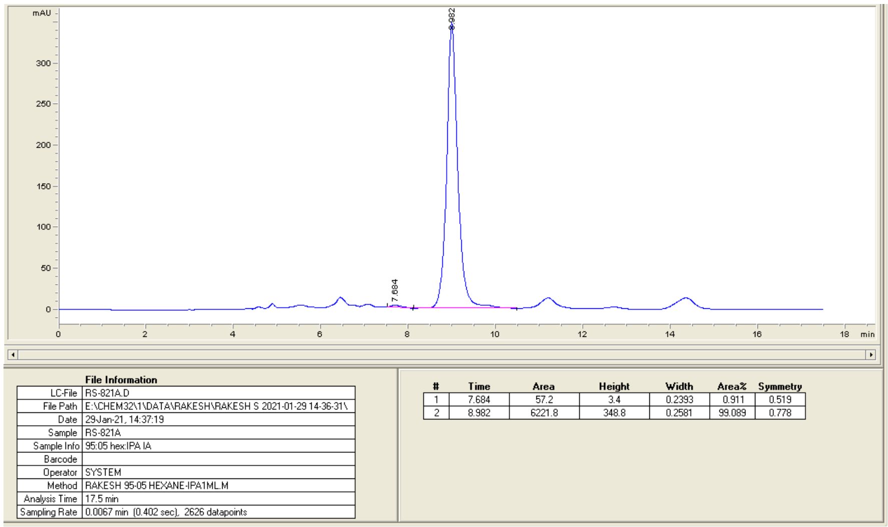

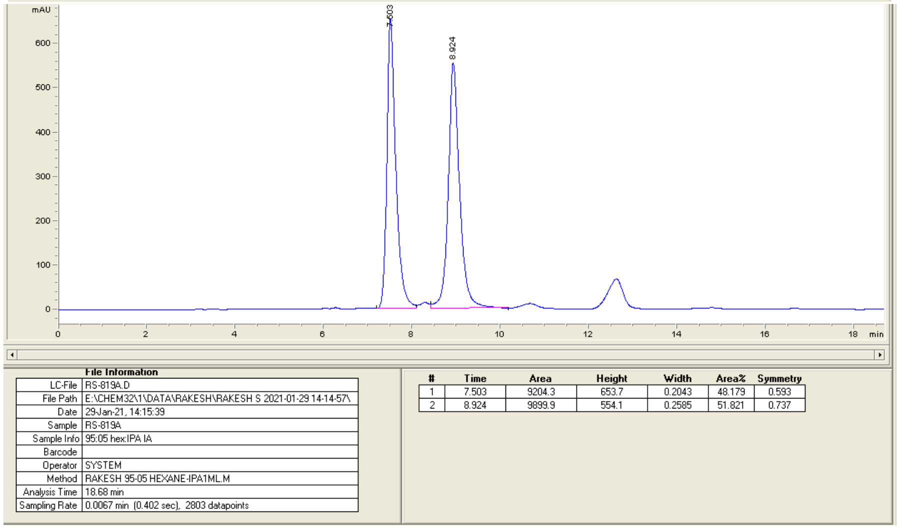

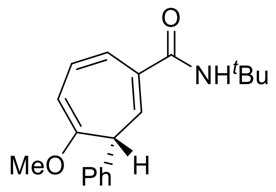

(*R*)-*N*-(tert-butyl)-4-methoxy-3-phenylcyclohepta-1,4,6-triene-1-carboxamide (4m)

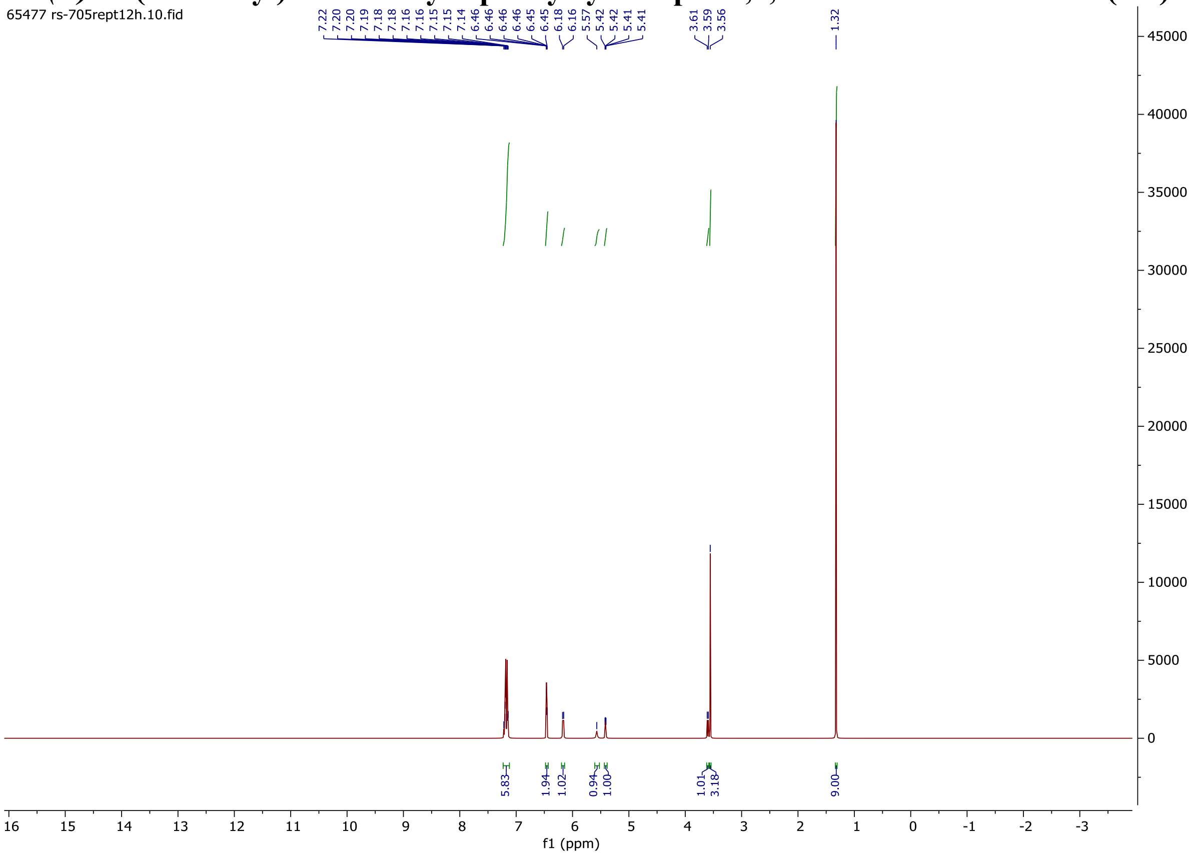

^{13}C NMR

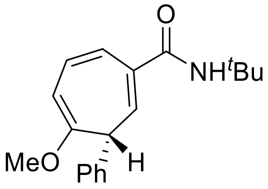

(R)-N-(tert-butyl)-4-methoxy-3-phenylcyclohepta-1,4,6-triene-1-carboxamide (4m)

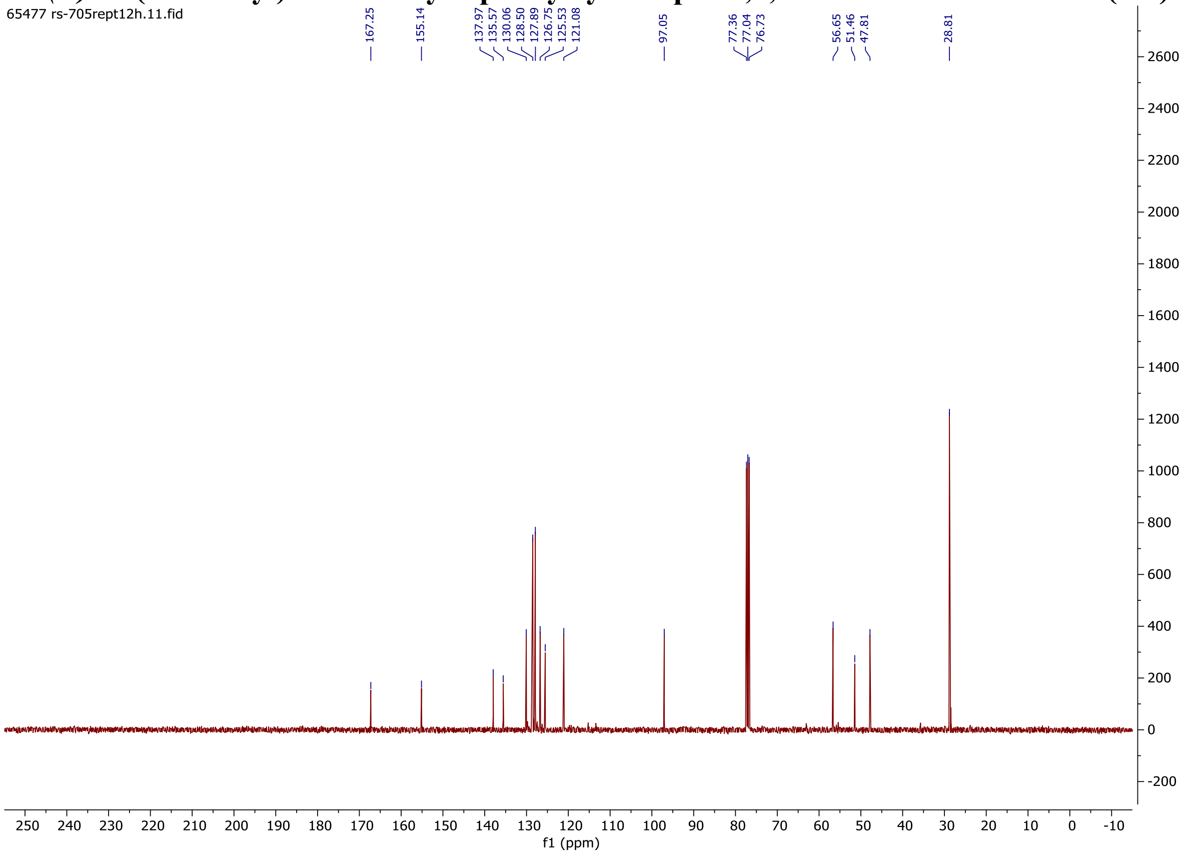

HPLC

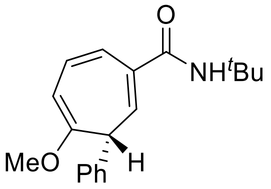

***(R)*-*N*-(tert-butyl)-4-methoxy-3-phenylcyclohepta-1,4,6-triene-1-carboxamide (4m)**

Analytical HPLC (IA Column), eluting with hexane-IPA–(10:90), showed it to consist of a mixture of 39:61 two enantiomers with retention times tR = 6.025 mins (minor) and 7.58 mins (major) respectively.

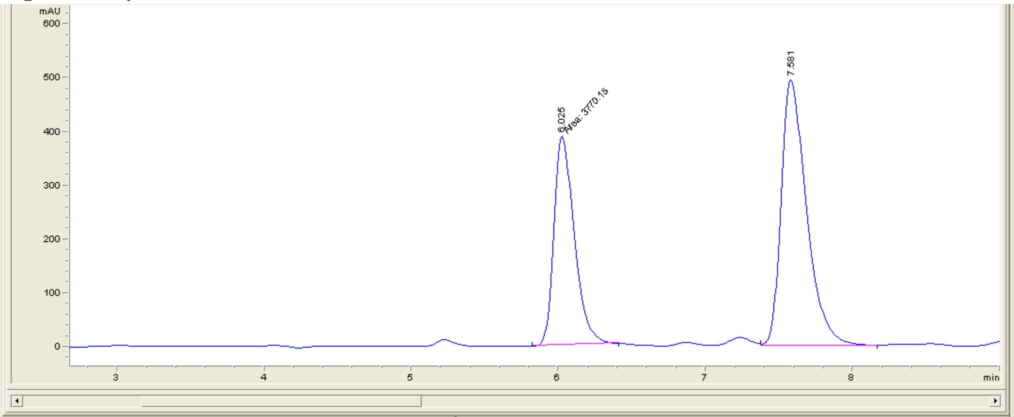

File Information

LC-File	RS-726 PREP.D
File Path	E:\CHEM32\1\DATA\RAKESH\RAKESH S 2020-11-05 09-52-08\
Date	05-Nov-20, 09:52:55
Sample	RS-726 prep
Sample Info	90:10 hex:IPA IA
Barcode	
Operator	SYSTEM
Method	RAKESH 90-10 HEXANE-IPA1ML.M
Analysis Time	21.88 min
Sampling Rate	0.0067 min (0.402 sec), 3283 datapoints

#	Time	Area	Height	Width	Area%	Symmetry
1	6.025	3770.2	388.7	0.1616	38.847	0.667
2	7.581	5935	494	0.1842	61.153	0.587

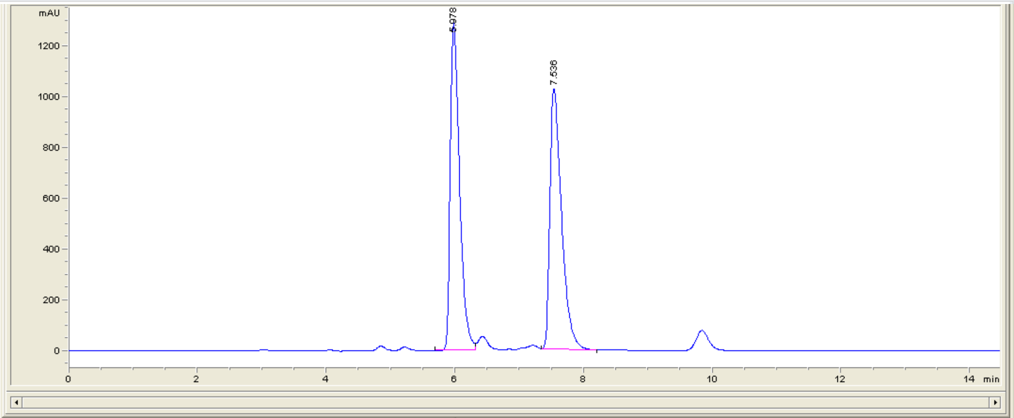

File Information

LC-File	RS-705A PREP.D
File Path	E:\CHEM32\1\DATA\RAKESH\RAKESH S 2020-11-05 09-32-20\
Date	05-Nov-20, 09:33:12
Sample	RS-705A prep
Sample Info	90:10 hex:IPA IA
Barcode	
Operator	SYSTEM
Method	RAKESH 90-10 HEXANE-IPA1ML.M
Analysis Time	14.473 min
Sampling Rate	0.0067 min (0.402 sec), 2172 datapoints

#	Time	Area	Height	Width	Area%	Symmetry
1	5.978	12965.2	1286.9	0.1535	49.941	0.533
2	7.536	12996	1027.2	0.1916	50.059	0.494

^{1}H NMR

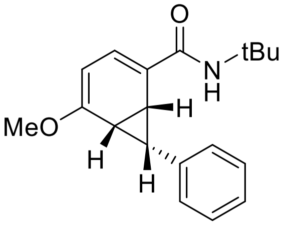

(1*R*,6*S*,7*S*)-*N*-(tert-butyl)-5-methoxy-7-phenylbicyclo[4.1.0]hepta-2,4-diene-2-carboxamide (5n)

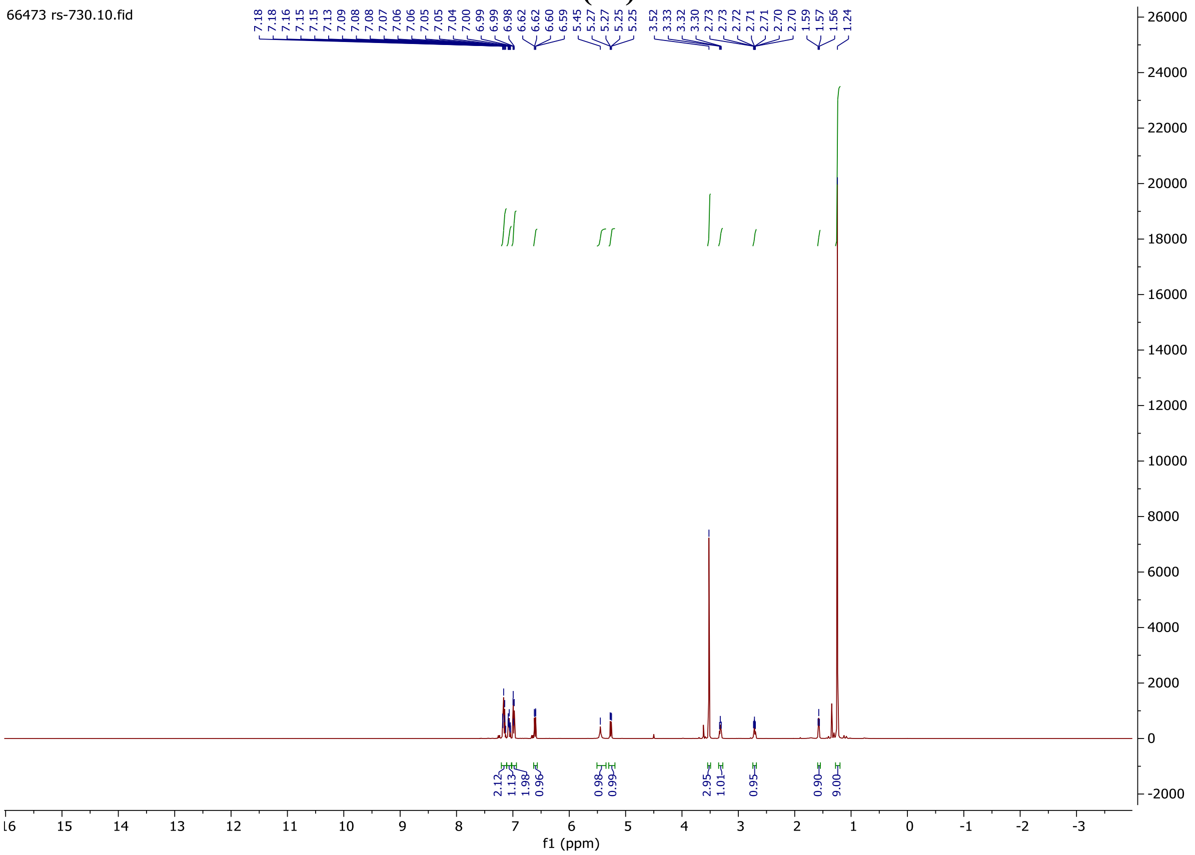

^{13}C NMR

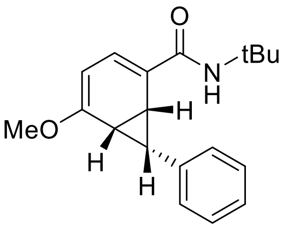

(1*R*,6*S*,7*S*)-*N*-(tert-butyl)-5-methoxy-7-phenylbicyclo[4.1.0]hepta-2,4-diene-2-carboxamide (5n)

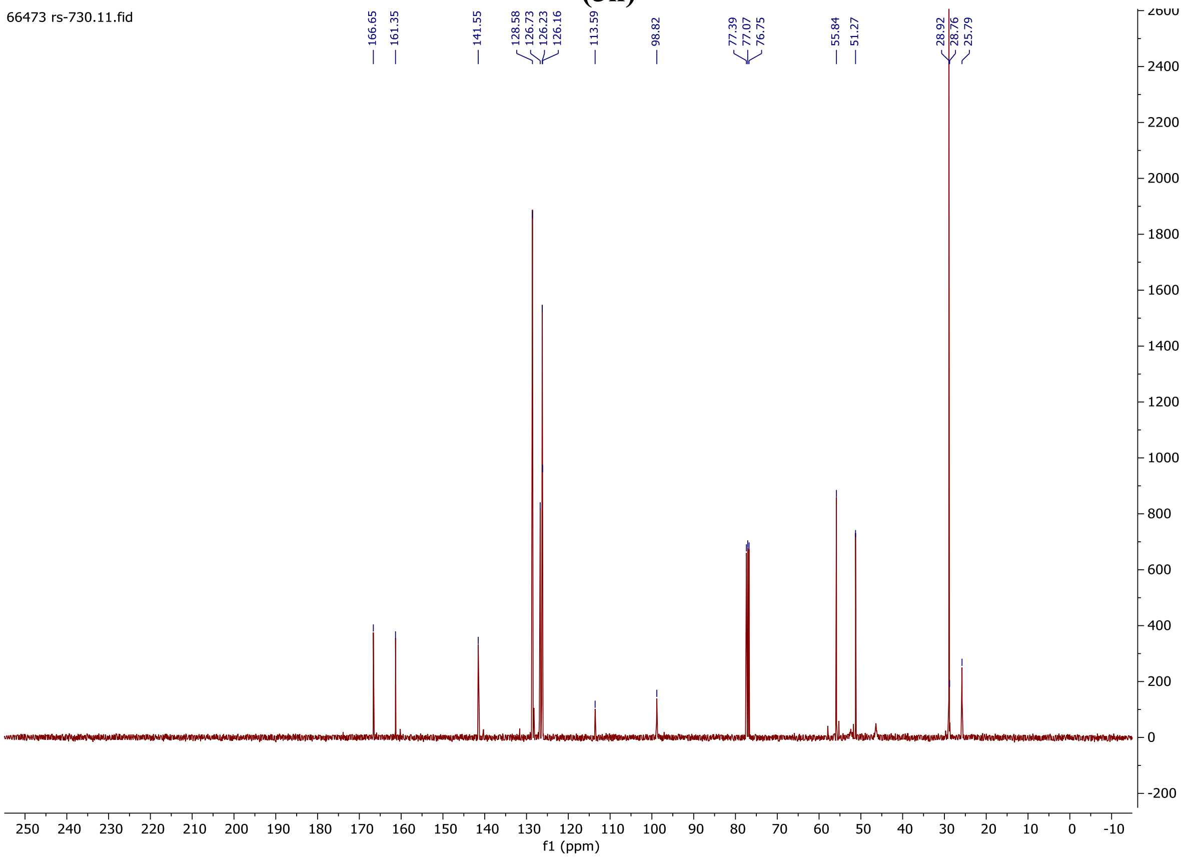

NOSY

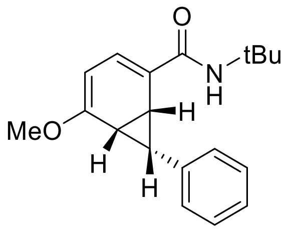

(1*R*,6*S*,7*S*)-*N*-(tert-butyl)-5-methoxy-7-phenylbicyclo[4.1.0]hepta-2,4-diene-2-carboxamide (5n)

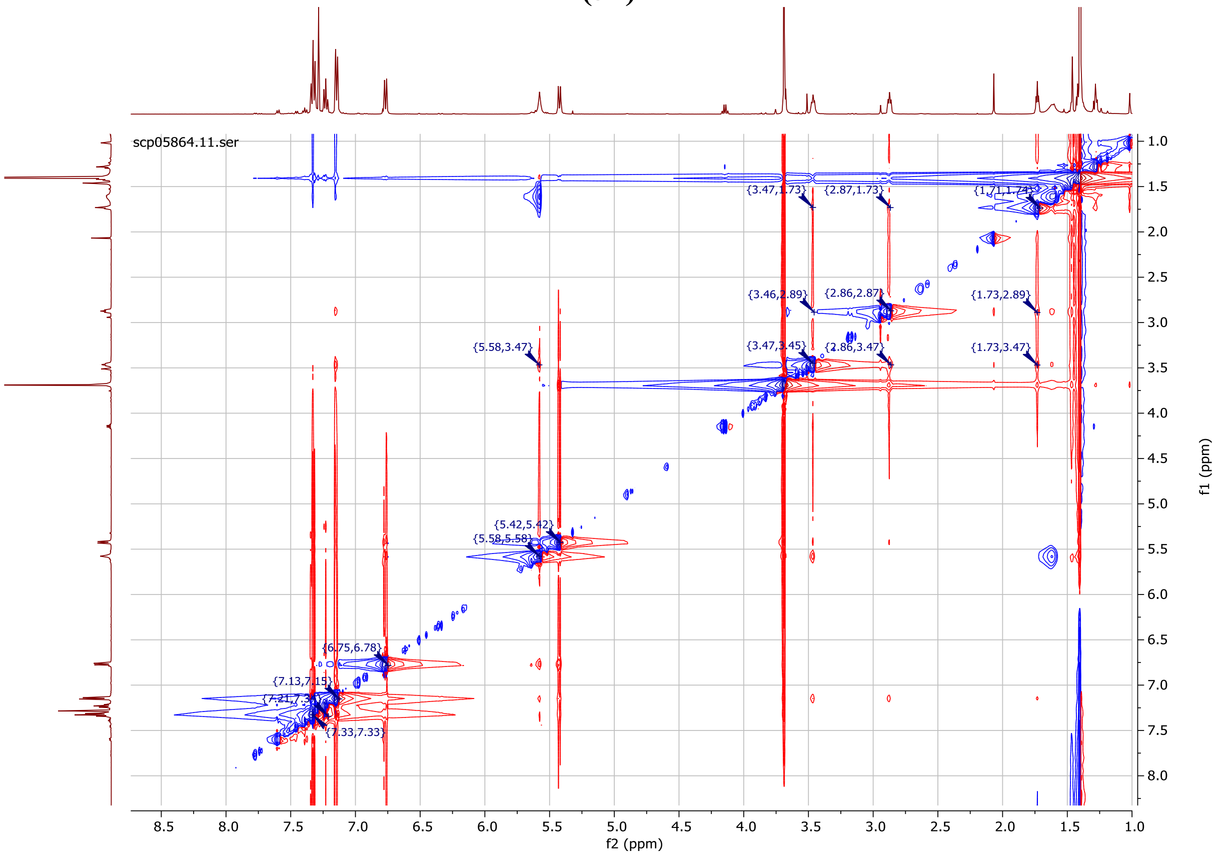

HPLC

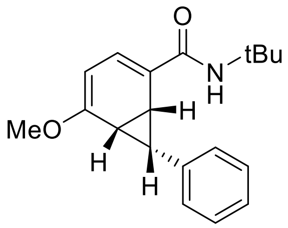

(1*R*,6*S*,7*S*)-*N*-(tert-butyl)-5-methoxy-7-phenylbicyclo[4.1.0]hepta-2,4-diene-2-carboxamide 5n)

Analytical HPLC (IA Column), eluting with hexane-IPA (95:5), showed it to consist of a 6:94 mixture of two enantiomers with retention times 18.05 mins (minor) and 23.82 mins (major) respectively.

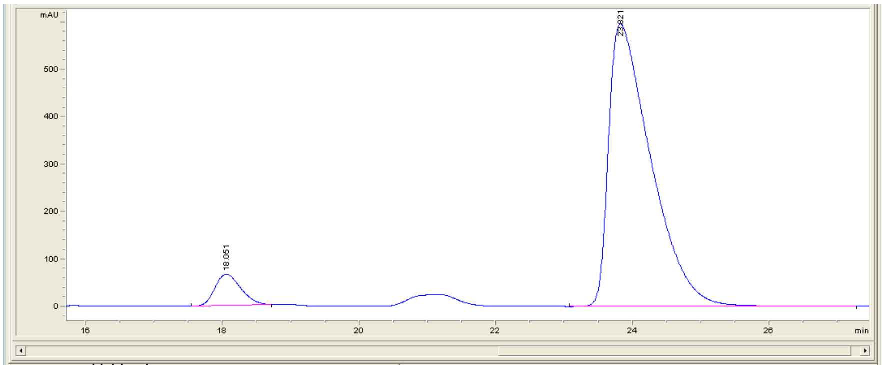

File Information

LC-File	RS-721PREP.D
File Path	E:\CHEM32\1\DATA\RAKESH\RAKESH S 2020-11-05 12-10-32\
Date	05-Nov-20, 12:11:20
Sample	RS-721prep
Sample Info	95:05 hex:IPA IA
Barcode	
Operator	SYSTEM
Method	RAKESH 95-05 HEXANE-IPA1ML.M
Analysis Time	27.773 min
Sampling Rate	0.0067 min (0.402 sec), 4167 datapoints

#	Time	Area	Height	Width	Area%	Symmetry
1	18.051	1729.5	66.2	0.4028	6.319	0.739
2	23.821	25641.2	596.4	0.6356	93.681	0.362

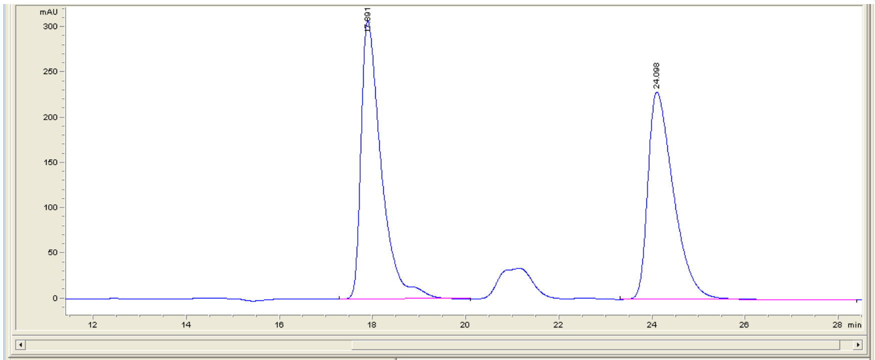

File Information

LC-File	RS-730.D
File Path	E:\CHEM32\1\DATA\RAKESH\RAKESH S 2020-11-05 12-43-32\
Date	05-Nov-20, 12:44:19
Sample	RS-730
Sample Info	95:05 hex:IPA IA
Barcode	
Operator	SYSTEM
Method	RAKESH 95-05 HEXANE-IPA1ML.M
Analysis Time	28.947 min
Sampling Rate	0.0067 min (0.402 sec), 4343 datapoints

#	Time	Area	Height	Width	Area%	Symmetry
1	17.891	9299.5	307.4	0.447	51.395	0.457
2	24.098	8794.8	229.3	0.5835	48.605	0.505

^{1}H NMR

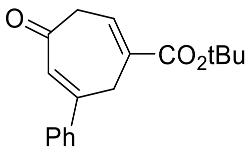

tert-butyl 4-oxo-6-phenylcyclohepta-1,5-diene-1-carboxylate (6n)

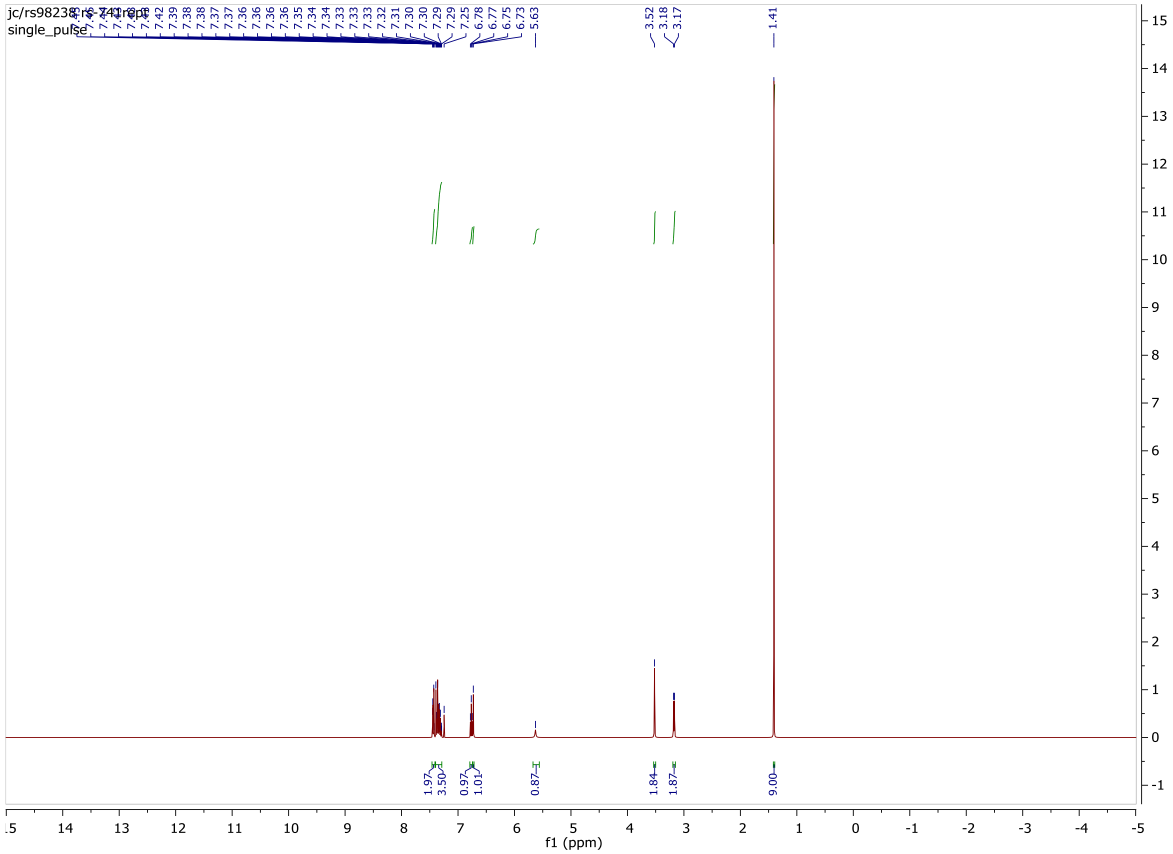

^{13}C NMR

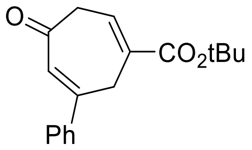

tert-butyl 4-oxo-6-phenylcyclohepta-1,5-diene-1-carboxylate (6n)

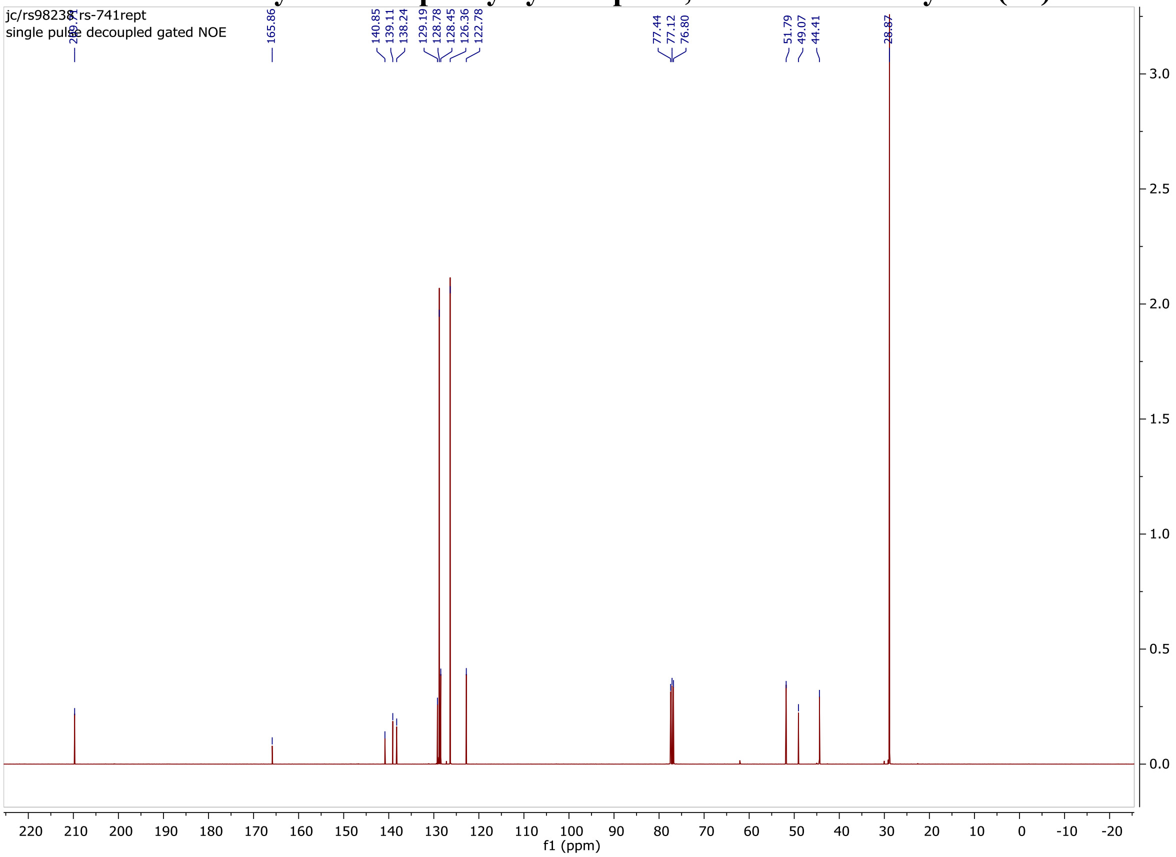

^{1}H NMR

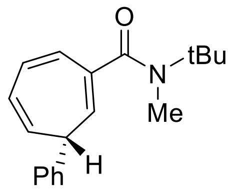

(*R*)-*N*-(*tert*-butyl)-*N*-methyl-3-phenylcyclohepta-1,4,6-triene-1-carboxamide (8)

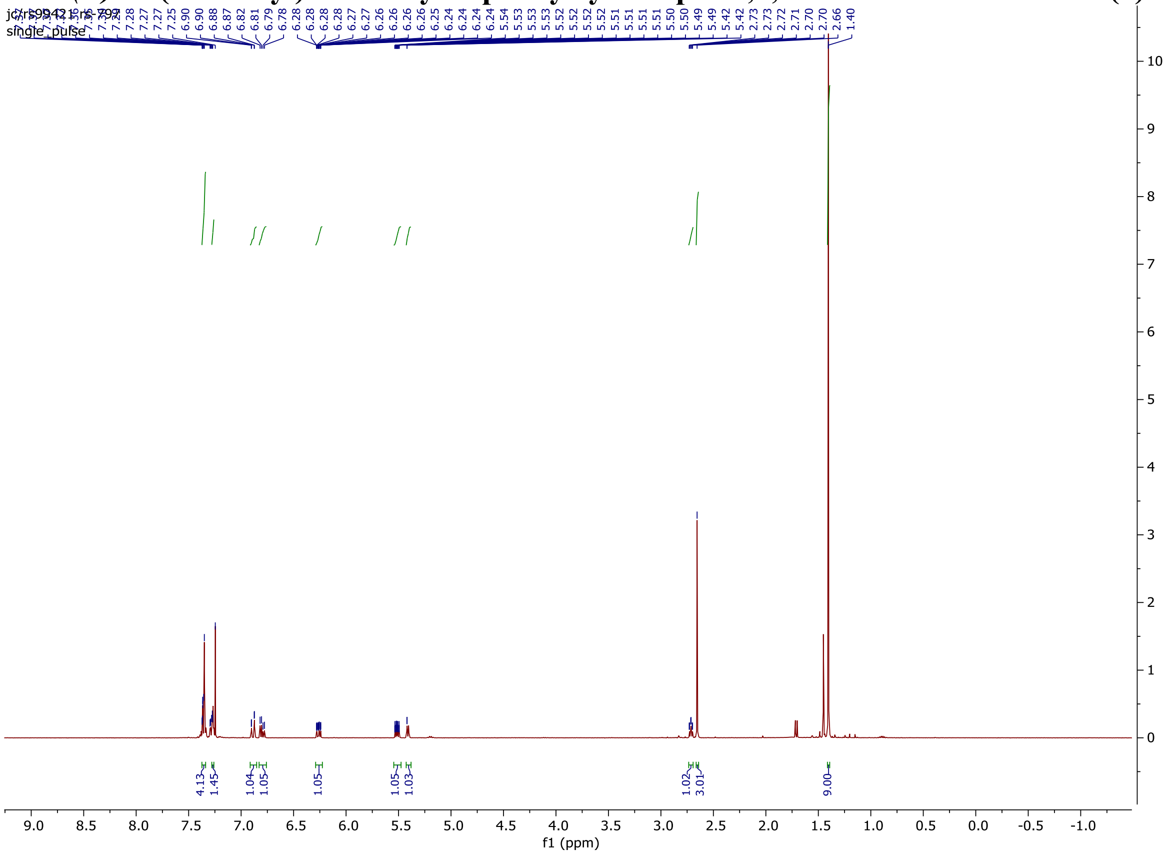

^{13}C NMR

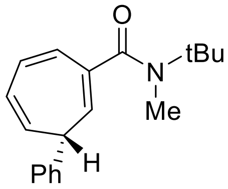

***(R)*-*N*-(*tert*-butyl)-*N*-methyl-3-phenylcyclohepta-1,4,6-triene-1-carboxamide (8)**

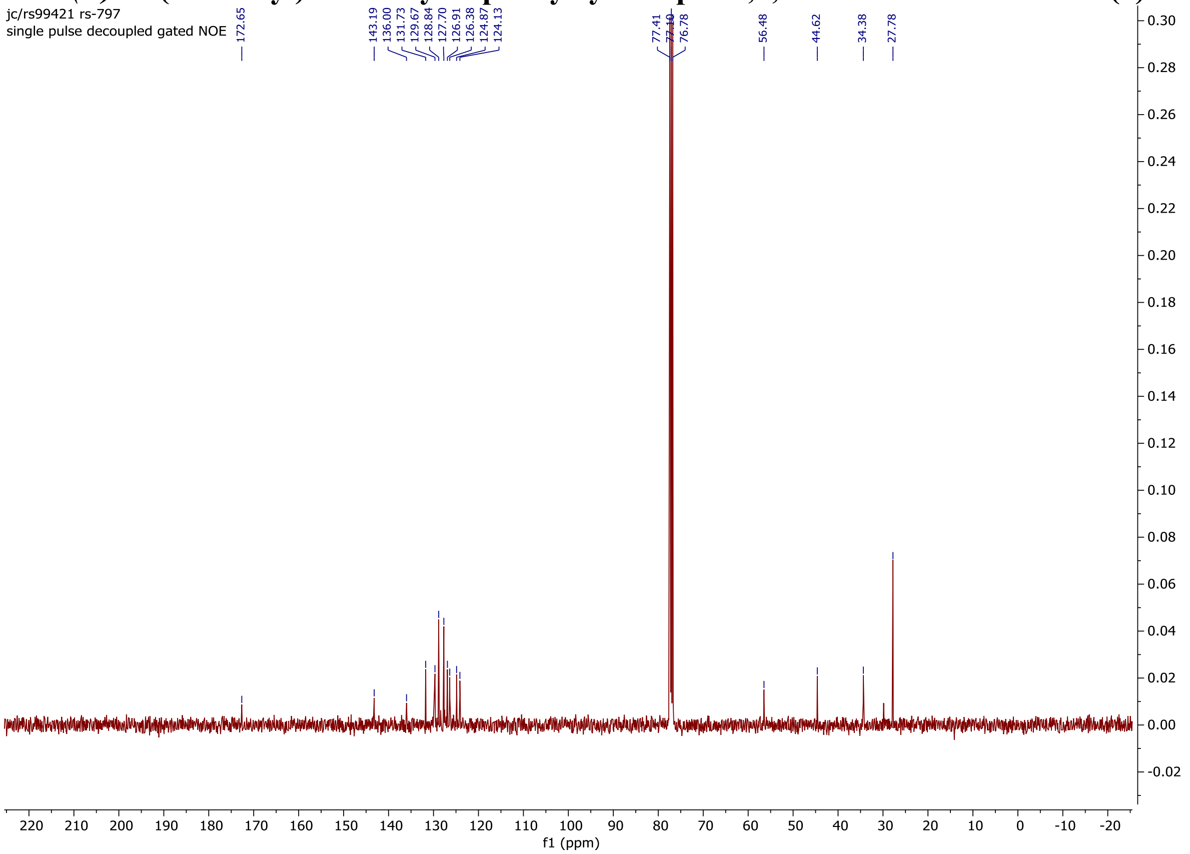

HPLC

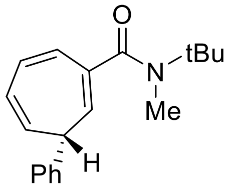

(*R*)-*N*-(*tert*-butyl)-*N*-methyl-3-phenylcyclohepta-1,4,6-triene-1-carboxamide (8)

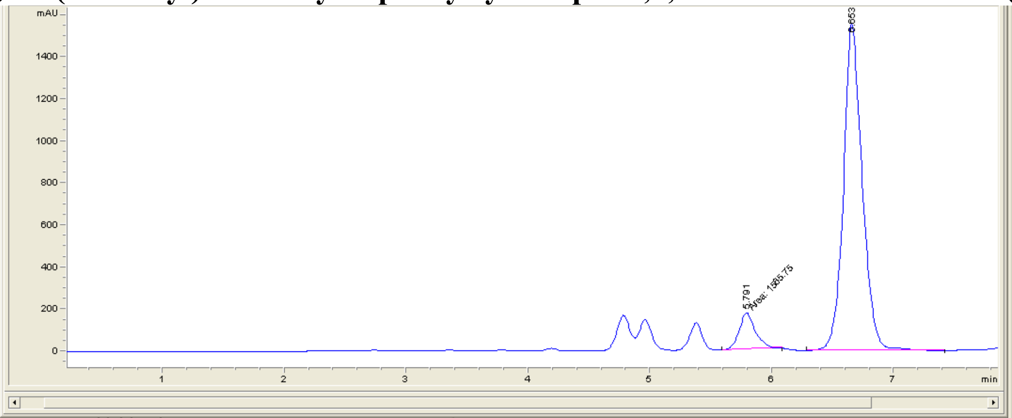

File Information

LC-File	RS-842.D
File Path	E:\CHEM32\1\DATA\RAKESH\RAKESH S 2021-02-24 16-51-33\
Date	24-Feb-21, 16:52:20
Sample	RS-842
Sample Info	95:05 hex:IPA IA
Barcode	
Operator	SYSTEM
Method	RAKESH 95-05 HEXANE-IPA1ML.M
Analysis Time	8.92 min
Sampling Rate	0.0067 min (0.402 sec), 1339 datapoints

#	Time	Area	Height	Width	Area%	Symmetry
1	5.791	1565.7	171.8	0.1519	8.590	0.807
2	6.653	16662.5	1554.9	0.157	91.410	0.759

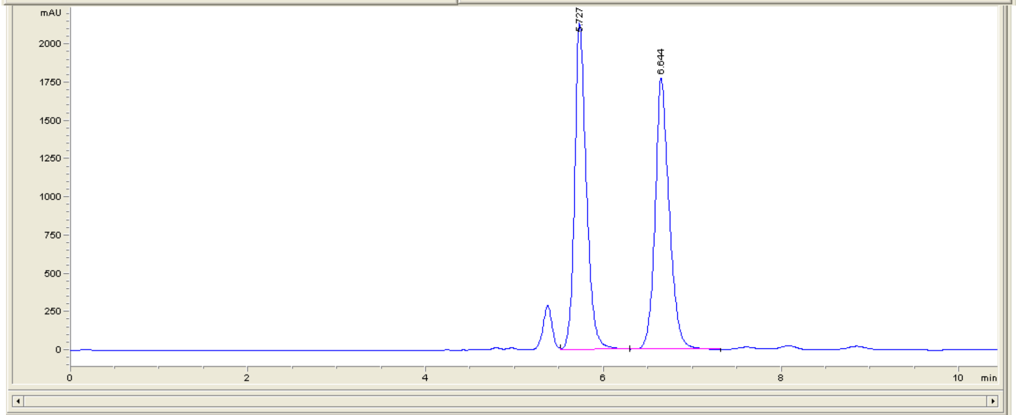

File Information

LC-File	RS-797.D
File Path	E:\CHEM32\1\DATA\RAKESH\RAKESH S 2021-02-24 16-38-23\
Date	24-Feb-21, 16:39:10
Sample	RS-797
Sample Info	95:05 hex:IPA IA
Barcode	
Operator	SYSTEM
Method	RAKESH 95-05 HEXANE-IPA1ML.M
Analysis Time	10.433 min
Sampling Rate	0.0067 min (0.402 sec), 1566 datapoints

#	Time	Area	Height	Width	Area%	Symmetry
1	5.727	19489.3	2133.8	0.1366	49.975	0.678
2	6.644	19509.2	1774.9	0.1602	50.025	0.698

^{1}H NMR

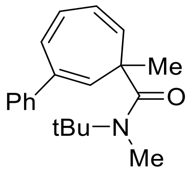

N-(tert-butyl)-N,1-dimethyl-3-phenylcyclohepta-2,4,6-triene-1-carboxamide (9)

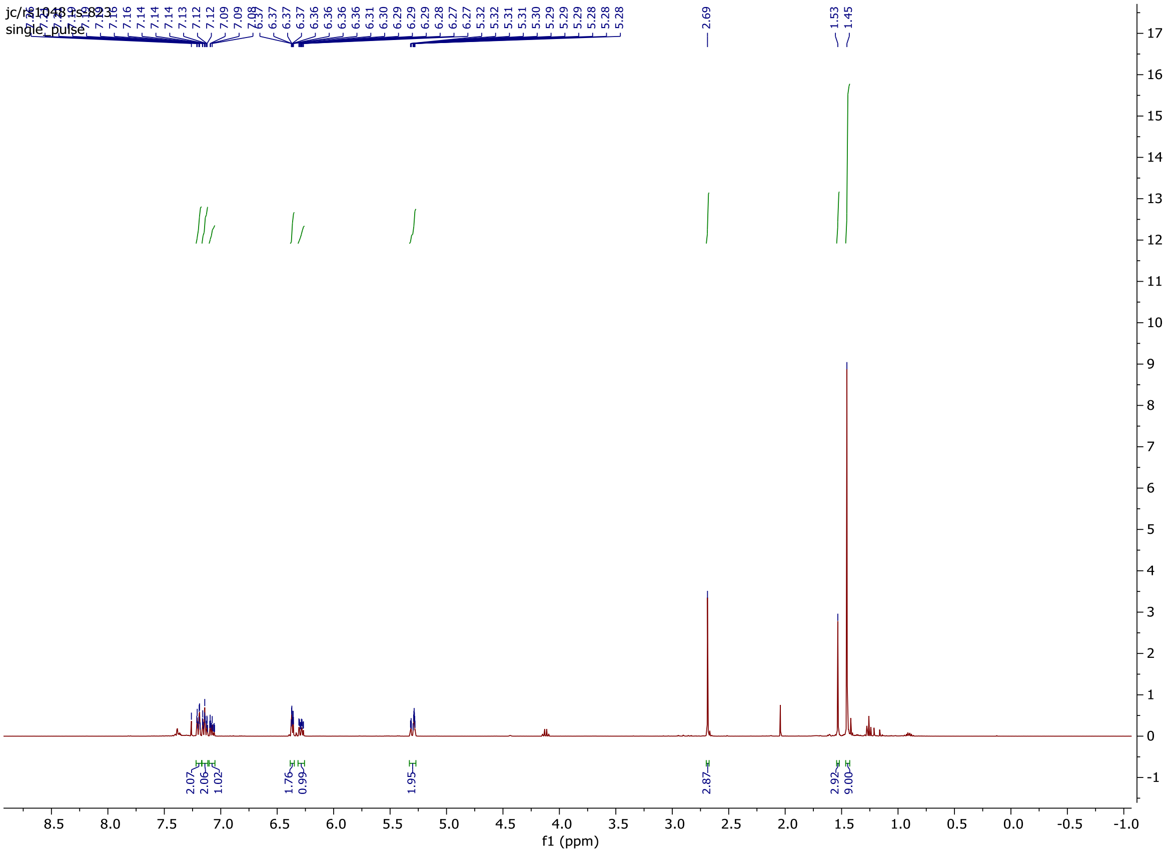

^{13}C NMR

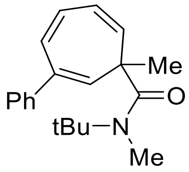

N-(tert-butyl)-N,1-dimethyl-3-phenylcyclohepta-2,4,6-triene-1-carboxamide (9)

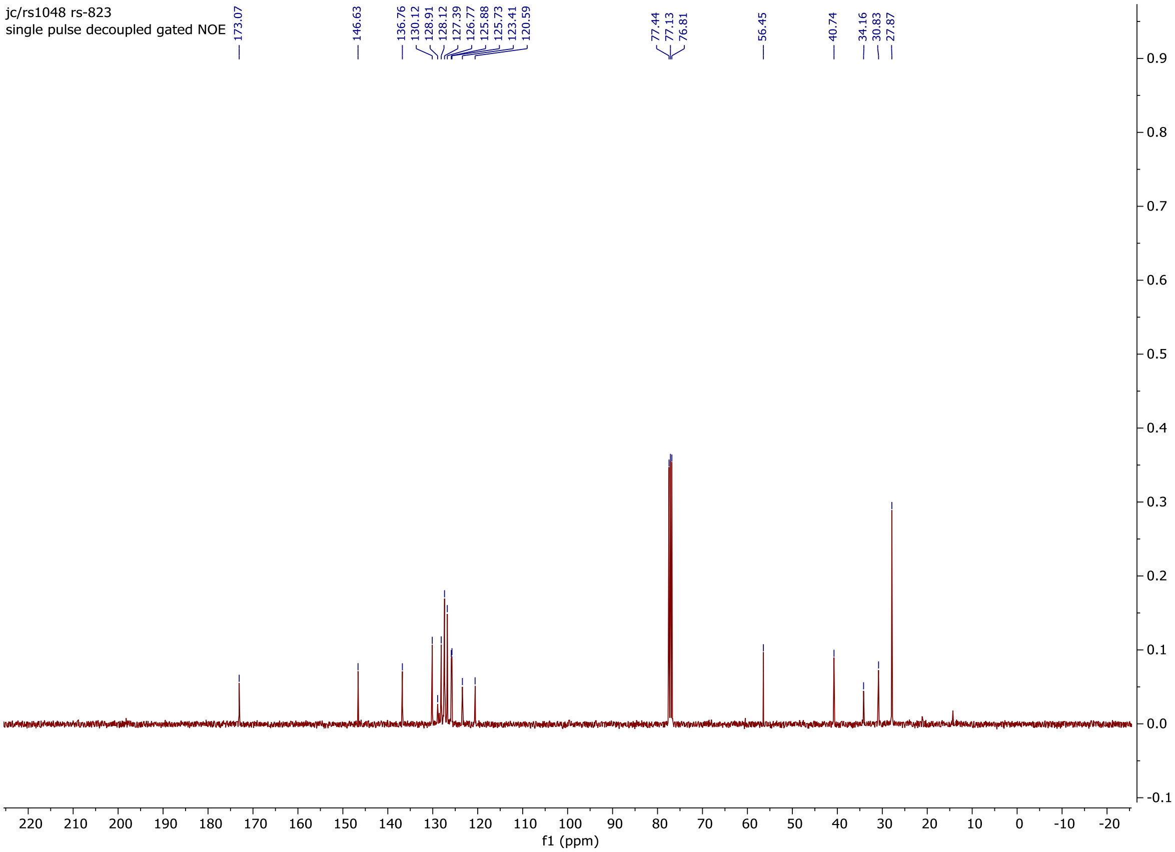

^{1}H NMR

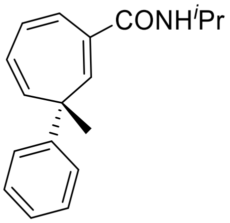

***(R)*-*N*-isopropyl-3-methyl-3-phenylcyclohepta-1,4,6-triene-1-carboxamide (11a)**

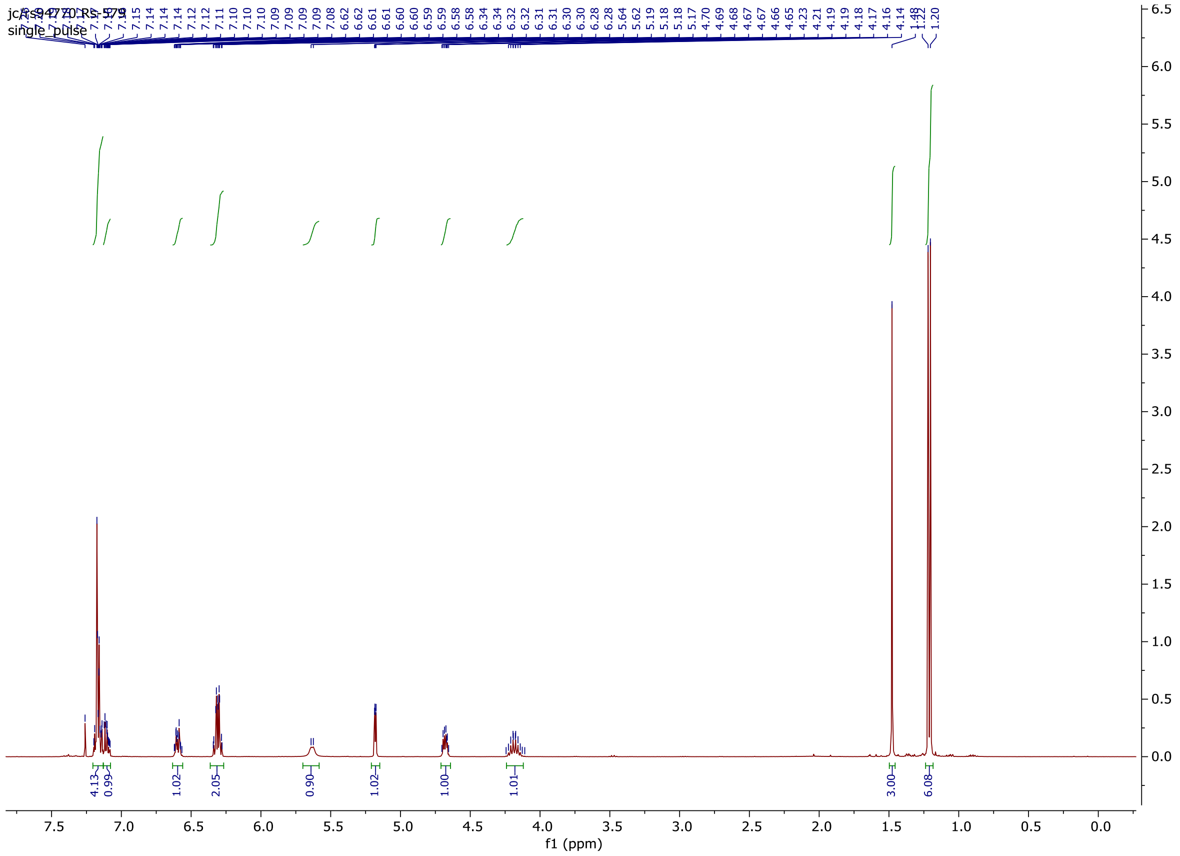

^{13}C NMR

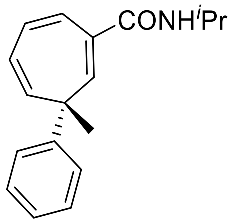

(R)-N-isopropyl-3-methyl-3-phenylcyclohepta-1,4,6-triene-1-carboxamide (11a)

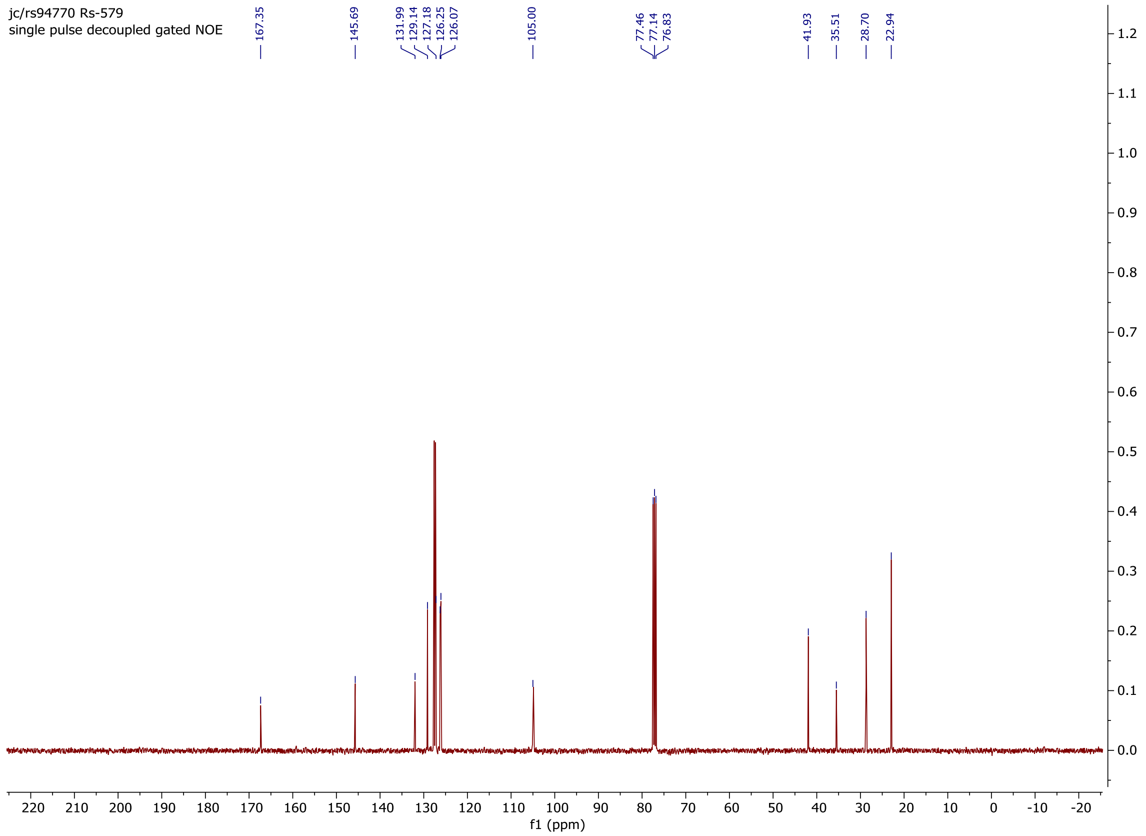

HPLC

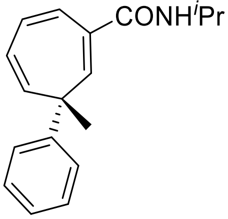

(R)-N-isopropyl-3-methyl-3-phenylcyclohepta-1,4,6-triene-1-carboxamide (11a)

Analytical HPLC (Chiral Regis Whelk O1), eluting with hexane-IPA (80:20), showed it to consist of a 0.42:99.57 mixture of two enantiomers with retention times 15.472 mins(minor) and 20.112 mins (major), respectively.

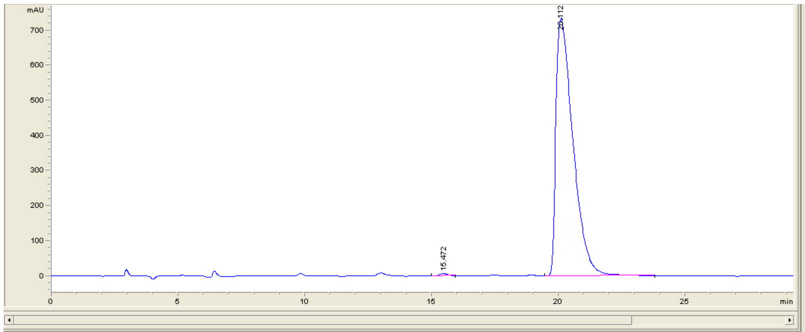

File Information

LC-File	RS-579.D
File Path	E:\CHEM32\1\DATA\RAKESH\RAKESH S 2020-03-18 11-25-48\
Date	18-Mar-20, 11:26:34
Sample	RS-579
Sample Info	80:20 hex:IPA IA
Barcode	
Operator	SYSTEM
Method	RAKESH 85-15 HEXANE-IPA1ML.M
Analysis Time	36.527 min
Sampling Rate	0.0067 min (0.402 sec), 5480 datapoints

#	Time	Area	Height	Width	Area%	Symmetry
1	15.472	148	6.6	0.3535	0.428	0.937
2	20.112	34396.5	733.7	0.7089	99.572	0.389

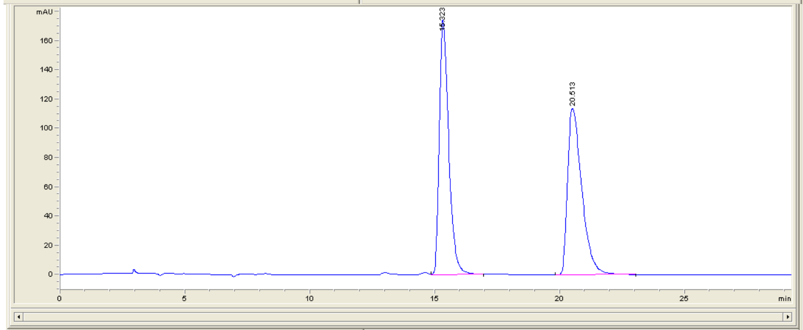

File Information

LC-File	RS-581.D
File Path	E:\CHEM32\1\DATA\RAKESH\RAKESH S 2020-03-20 17-49-30\
Date	20-Mar-20, 17:50:10
Sample	RS-581
Sample Info	80:20 hex:IPA IA
Barcode	
Operator	SYSTEM
Method	RAKESH 85-15 HEXANE-IPA1ML.M
Analysis Time	29.287 min
Sampling Rate	0.0067 min (0.402 sec), 4394 datapoints

#	Time	Area	Height	Width	Area%	Symmetry
1	15.323	4565.8	174	0.4021	50.338	0.592
2	20.513	4504.4	113.7	0.6063	49.662	0.501

^{1}H NMR

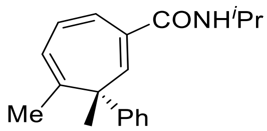

(S)-N-isopropyl-3,4-dimethyl-3-phenylcyclohepta-1,4,6-triene-1-carboxamide (11b)

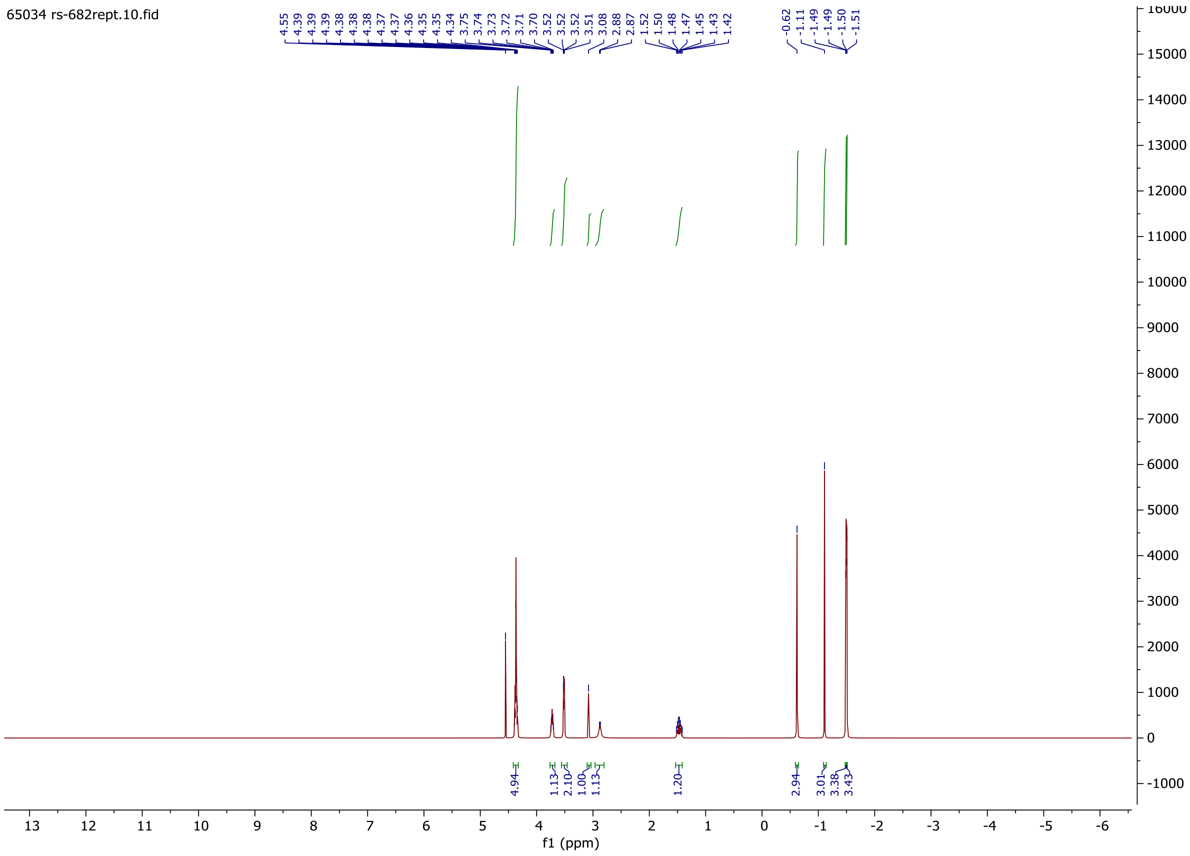

^{13}C NMR

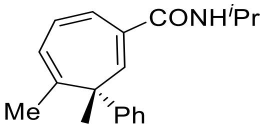

***(S)-N-isopropyl-3,4-dimethyl-3-phenylcyclohepta-1,4,6-triene-1-carboxamide* (11b)**

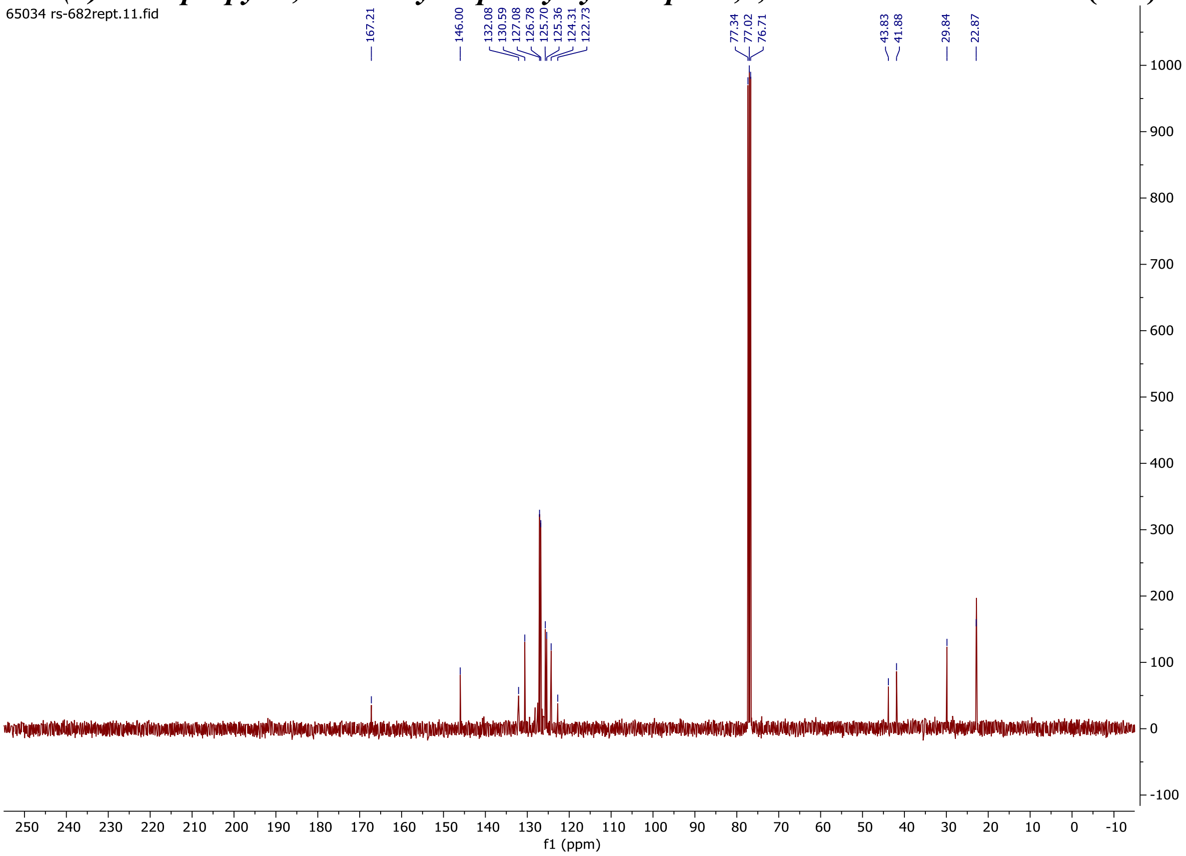

HPLC

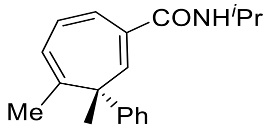

(*S*)-*N*-isopropyl-3,4-dimethyl-3-phenylcyclohepta-1,4,6-triene-1-carboxamide (11b)

Analytical HPLC (Chiral Regis Whelk O1), eluting with hexane-IPA (85:15), showed it to consist of a 2:98 mixture of two enantiomers with retention times 25.838 mins(minor) and 28.485 mins (major), respectively.

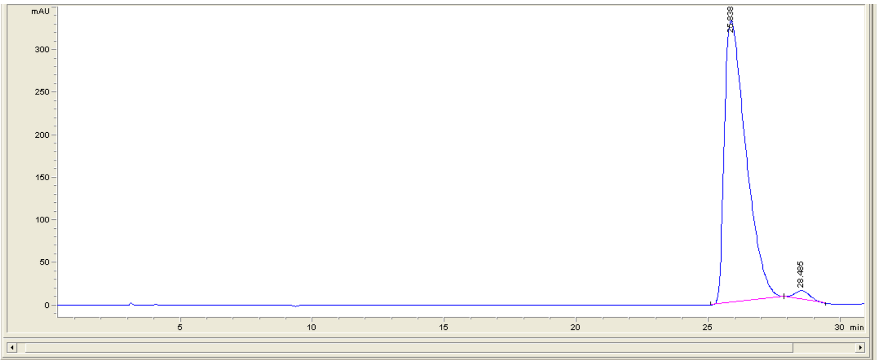

File Information

LC-File	RS-689PREP.D
File Path	E:\CHEM32\1\DATA\RAKESH\RAKESH S 2020-09-25 17-26-01\
Date	25-Sep-20, 17:26:47
Sample	RS-689 prep
Sample Info	85:15 hex:IPA IA
Barcode	
Operator	SYSTEM
Method	RAKESH 90-10 HEXANE-IPA1ML.M
Analysis Time	33.327 min
Sampling Rate	0.0067 min (0.402 sec), 5000 datapoints

#	Time	Area	Height	Width	Area%	Symmetry
1	25.838	18850	331.4	0.8655	97.870	0.411
2	28.485	410.2	10	0.6343	2.130	0.704

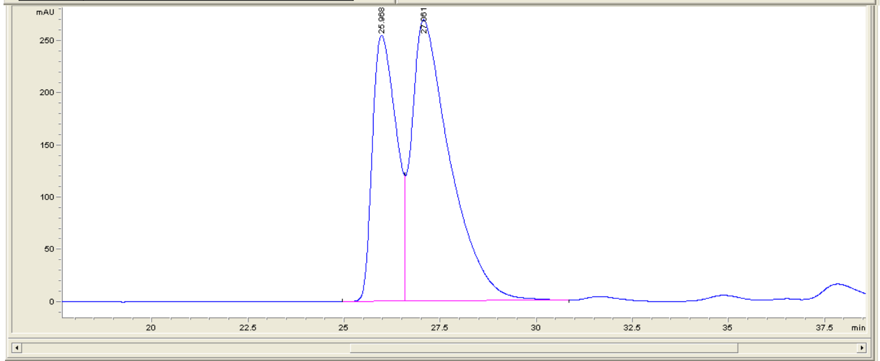

File Information

LC-File	RS-682.D
File Path	E:\CHEM32\1\DATA\RAKESH\RAKESH S 2020-09-23 12-43-28\
Date	23-Sep-20, 12:44:14
Sample	RS-682
Sample Info	90:10 hex:IPA IA
Barcode	
Operator	SYSTEM
Method	RAKESH 90-10 HEXANE-IPA1ML.M
Analysis Time	44.94 min
Sampling Rate	0.0067 min (0.402 sec), 6742 datapoints

#	Time	Area	Height	Width	Area%	Symmetry
1	25.968	11132.5	254.9	0.6755	37.206	0.601
2	27.061	18789.1	269.7	1.0028	62.794	0.408

^{1}H NMR

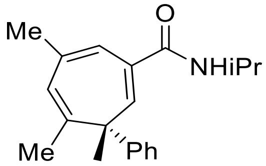

(S)-*N*-isopropyl-3,4,6-trimethyl-3-phenylcyclohepta-1,4,6-triene-1-carboxamide (11c)

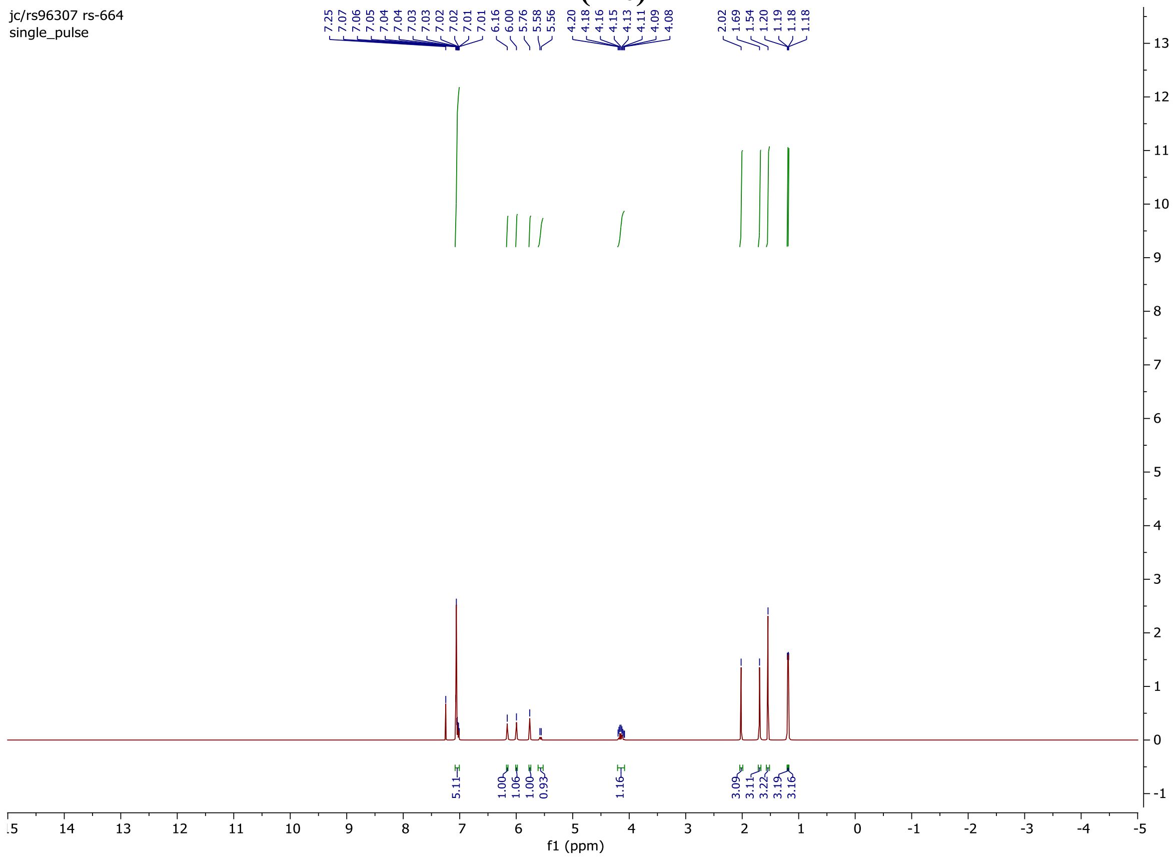

^{13}C NMR

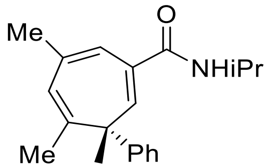

(S)-N-isopropyl-3,4,6-trimethyl-3-phenylcyclohepta-1,4,6-triene-1-carboxamide (11c)

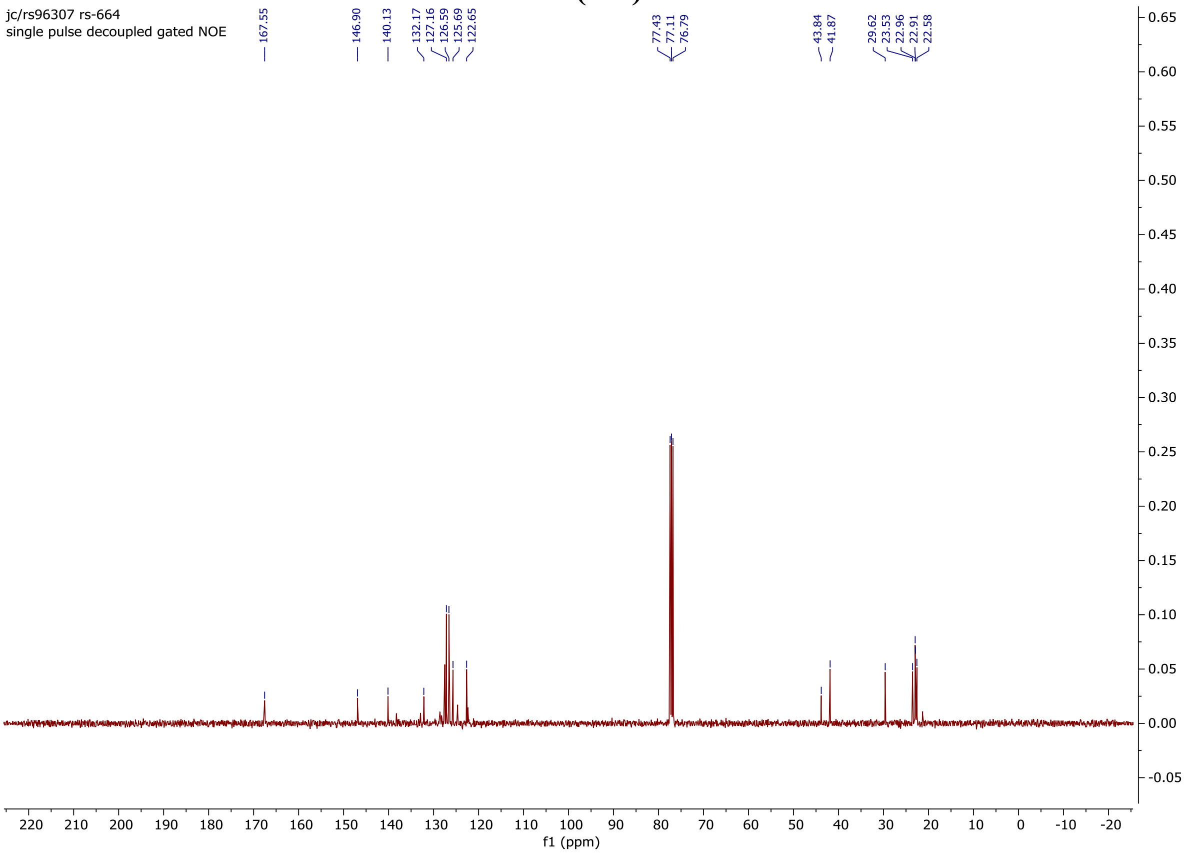

HPLC

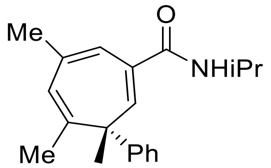

(S)-N-isopropyl-3,4,6-trimethyl-3-phenylcyclohepta-1,4,6-triene-1-carboxamide (11c)

Analytical HPLC (Chiral Regis Whelk O1), eluting with hexane-IPA (85:15), showed it to consist of a 90:10 mixture of two enantiomers with retention times 14.816 mins(major) and 16.019 mins (minor), respectively.

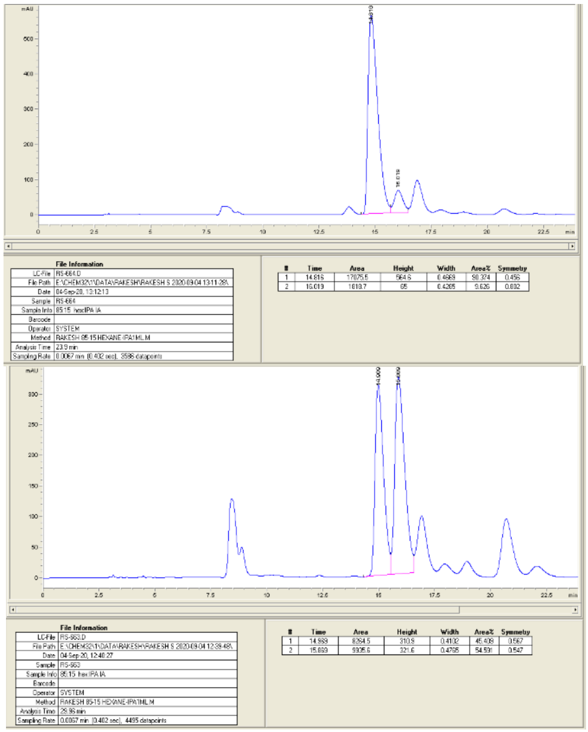

^{1}H NMR

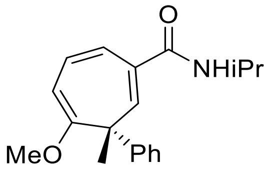

***(R)*-*N*-isopropyl-4-methoxy-3-methyl-3-phenylcyclohepta-1,4,6-triene-1-carboxamide (11d)**

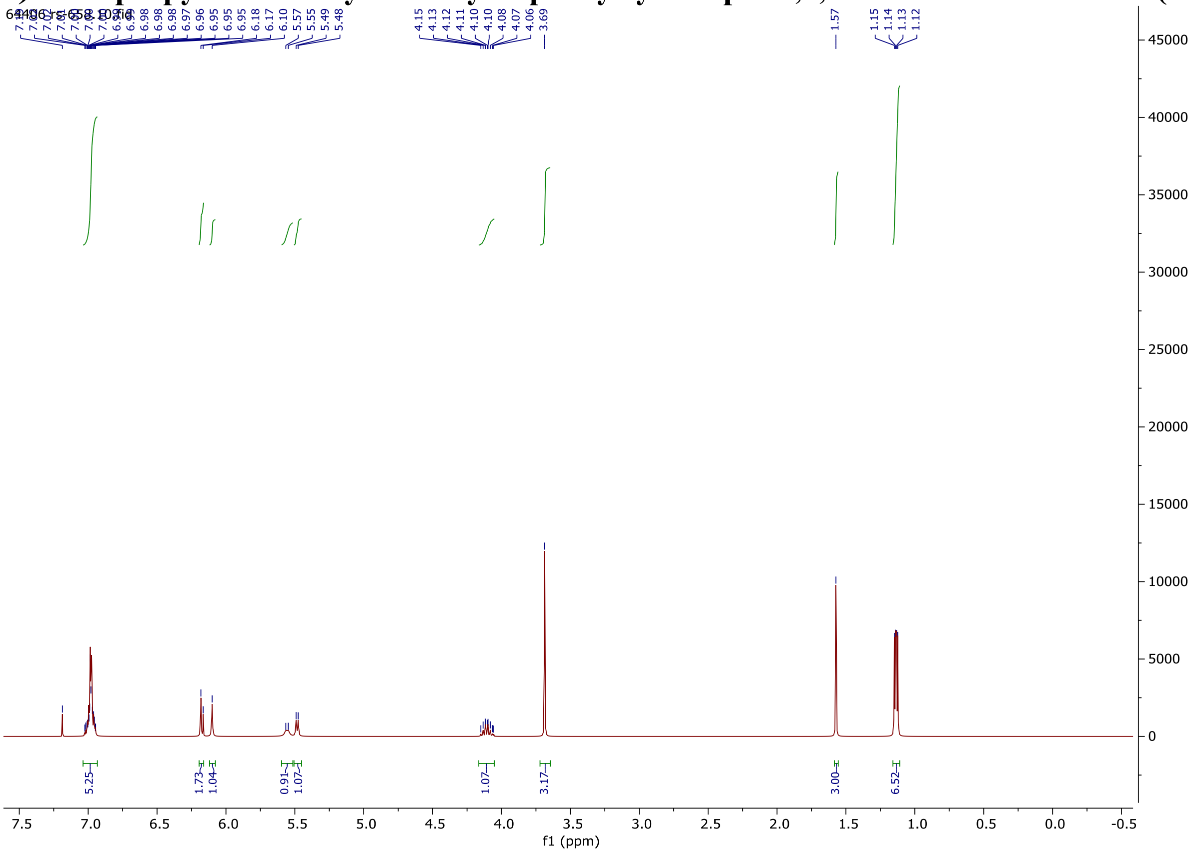

^{13}C NMR

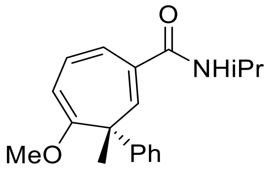

***(R)*-*N*-isopropyl-4-methoxy-3-methyl-3-phenylcyclohepta-1,4,6-triene-1-carboxamide (11d)**

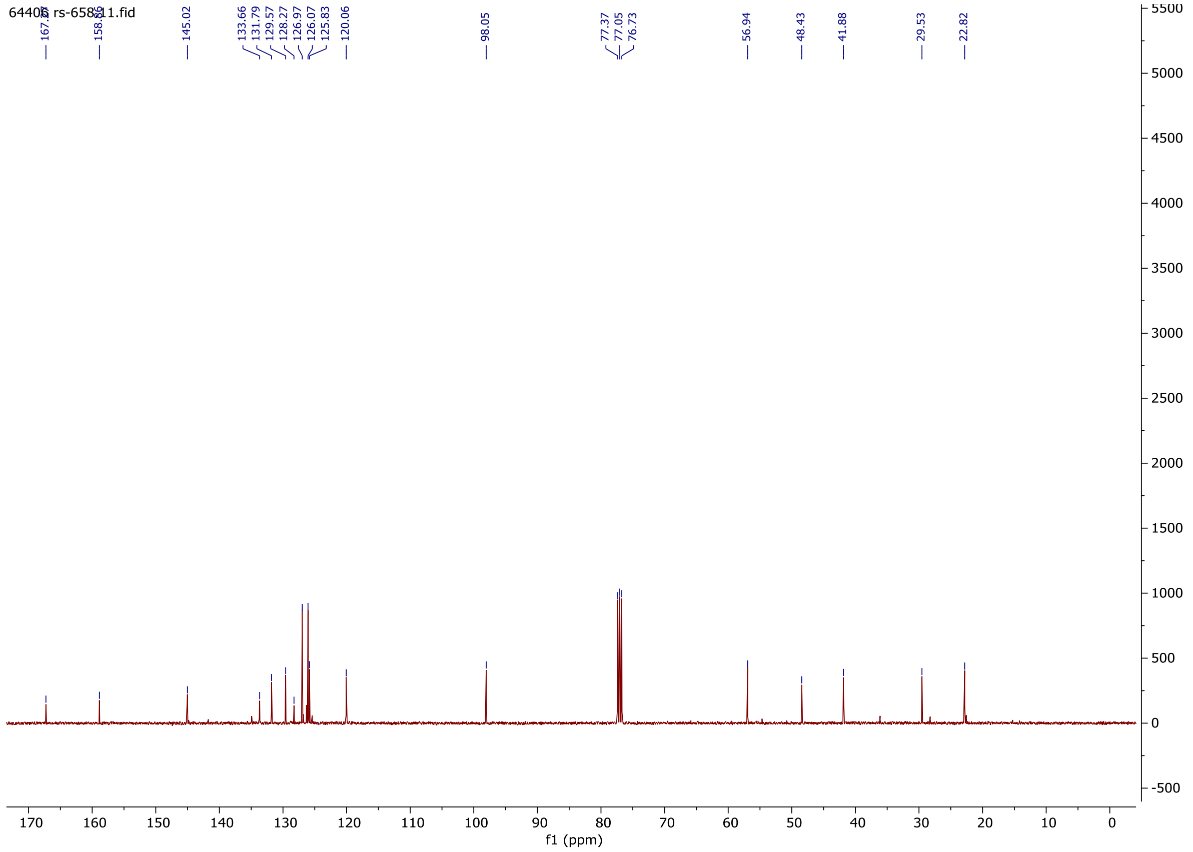

HPLC

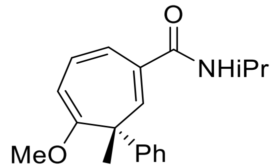

***(R)*-*N*-isopropyl-4-methoxy-3-methyl-3-phenylcyclohepta-1,4,6-triene-1-carboxamide (11d)**

Analytical HPLC (Chiral Regis Whelk O1), eluting with hexane-IPA (85:15), showed it to consist of a 100:00 mixture of one enantiomer with retention times 17.575 mins.

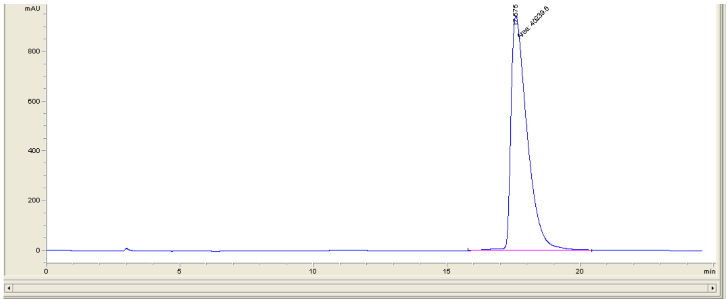

File Information

LC-File	RS-660.D
File Path	E:\CHEM32\1\DATA\RAKESH\RAKESH S 2020-08-28 17-08-54\
Date	28-Aug-20, 17:09:40
Sample	RS-660
Sample Info	85:15 hex:IPA IA
Barcode	
Operator	SYSTEM
Method	RAKESH 85-15 HEXANE-IPA1ML.M
Analysis Time	24.573 min
Sampling Rate	0.0067 min (0.402 sec), 3687 datapoints

#	Time	Area	Height	Width	Area%	Symmetry
1	17.575	40239.6	945	0.7097	100.000	0.418

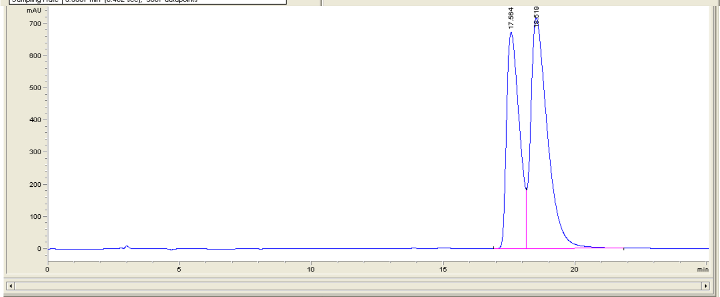

File Information

LC-File	RS-658.D
File Path	E:\CHEM32\1\DATA\RAKESH\RAKESH S 2020-08-28 16-39-40\
Date	28-Aug-20, 16:40:26
Sample	RS-658
Sample Info	85:15 hex:IPA IA
Barcode	
Operator	SYSTEM
Method	RAKESH 85-15 HEXANE-IPA1ML.M
Analysis Time	25.08 min
Sampling Rate	0.0067 min (0.402 sec), 3763 datapoints

#	Time	Area	Height	Width	Area%	Symmetry
1	17.564	22615.1	673	0.52	41.747	0.524
2	18.519	31556.2	717.1	0.6634	58.253	0.456

^{1}H NMR

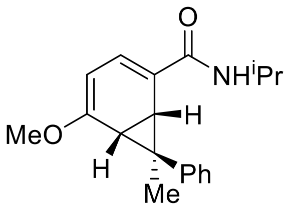

(1*S*,6*R*,7*R*)-*N*-isopropyl-5-methoxy-7-methyl-7-phenylbicyclo[4.1.0]hepta-2,4-diene-2-carboxamide (12e)

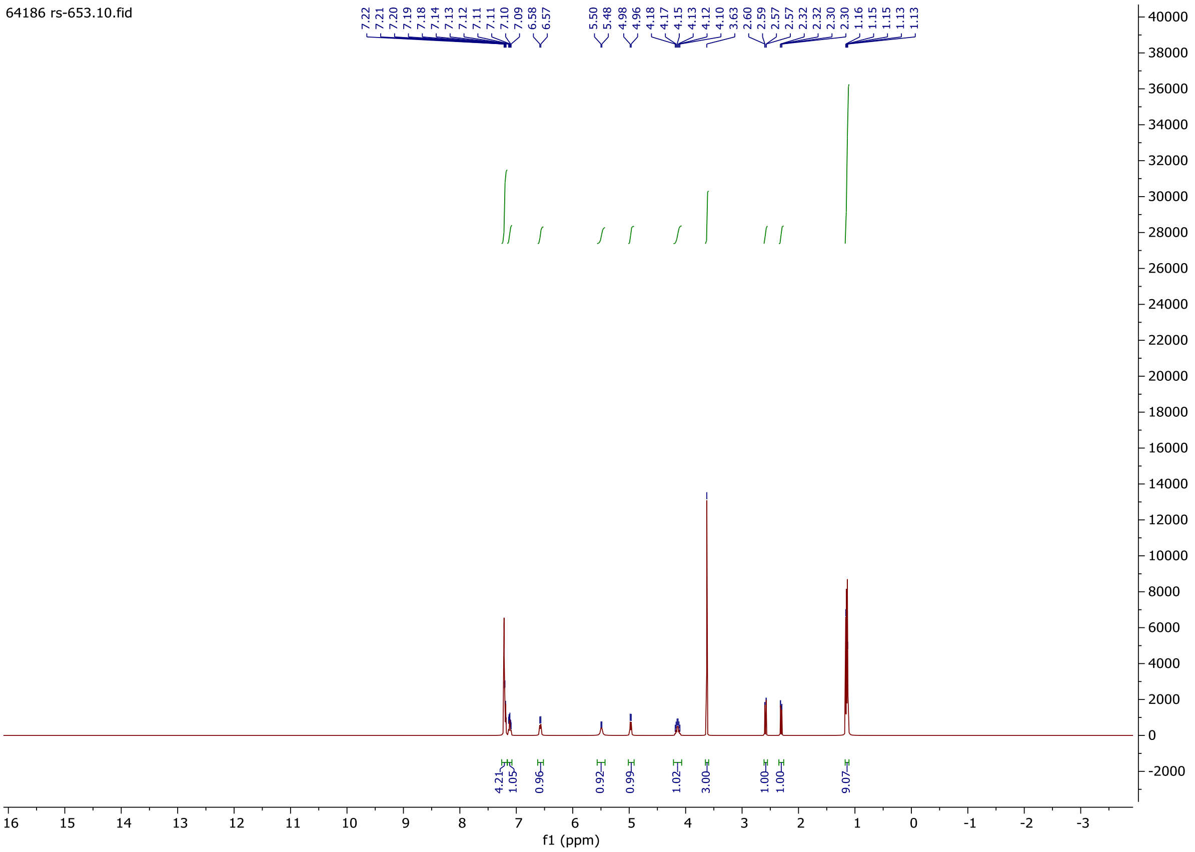

^{13}C NMR

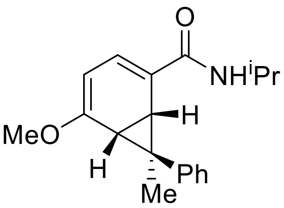

(1*S*,6*R*,7*R*)-*N*-isopropyl-5-methoxy-7-methyl-7-phenylbicyclo[4.1.0]hepta-2,4-diene-2-carboxamide (12e)

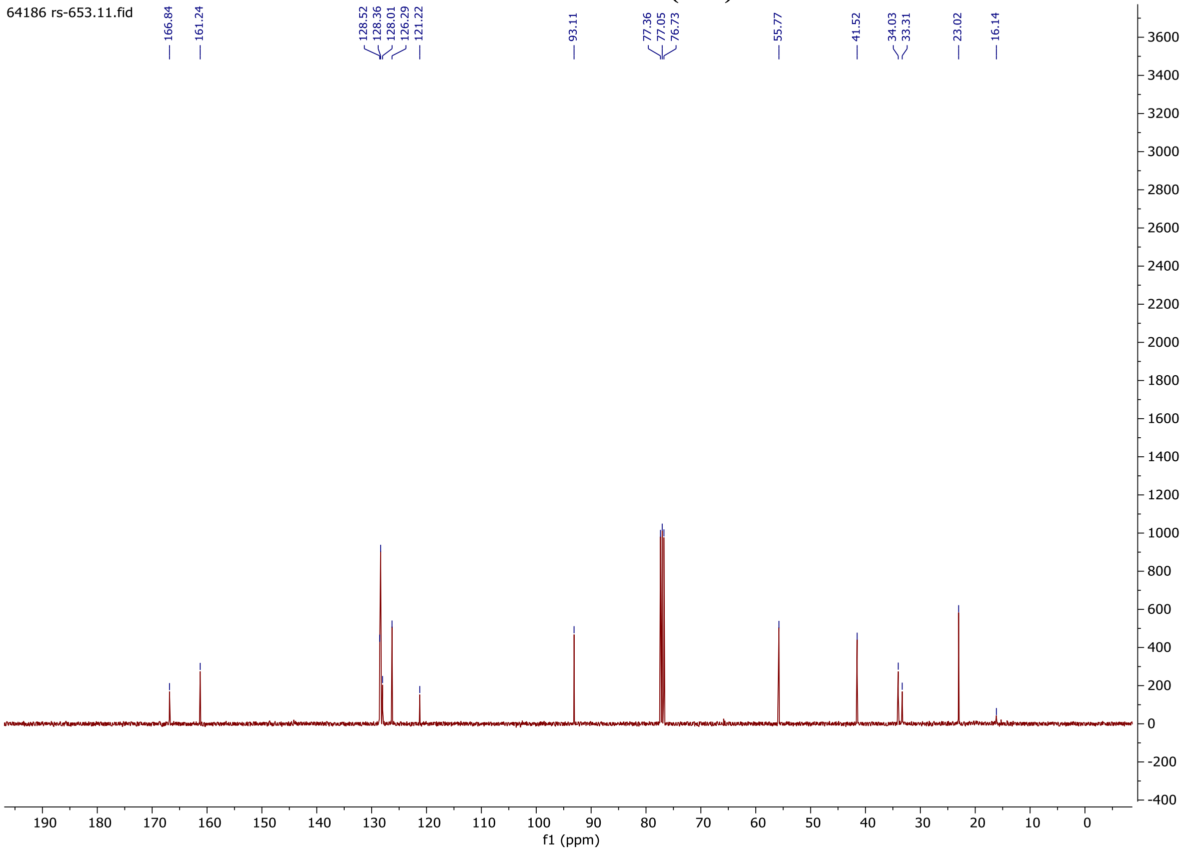

NOSY

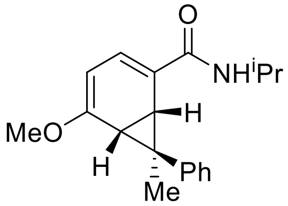

(1*S*,6*R*,7*R*)-*N*-isopropyl-5-methoxy-7-methyl-7-phenylbicyclo[4.1.0]hepta-2,4-diene-2-carboxamide (12e)

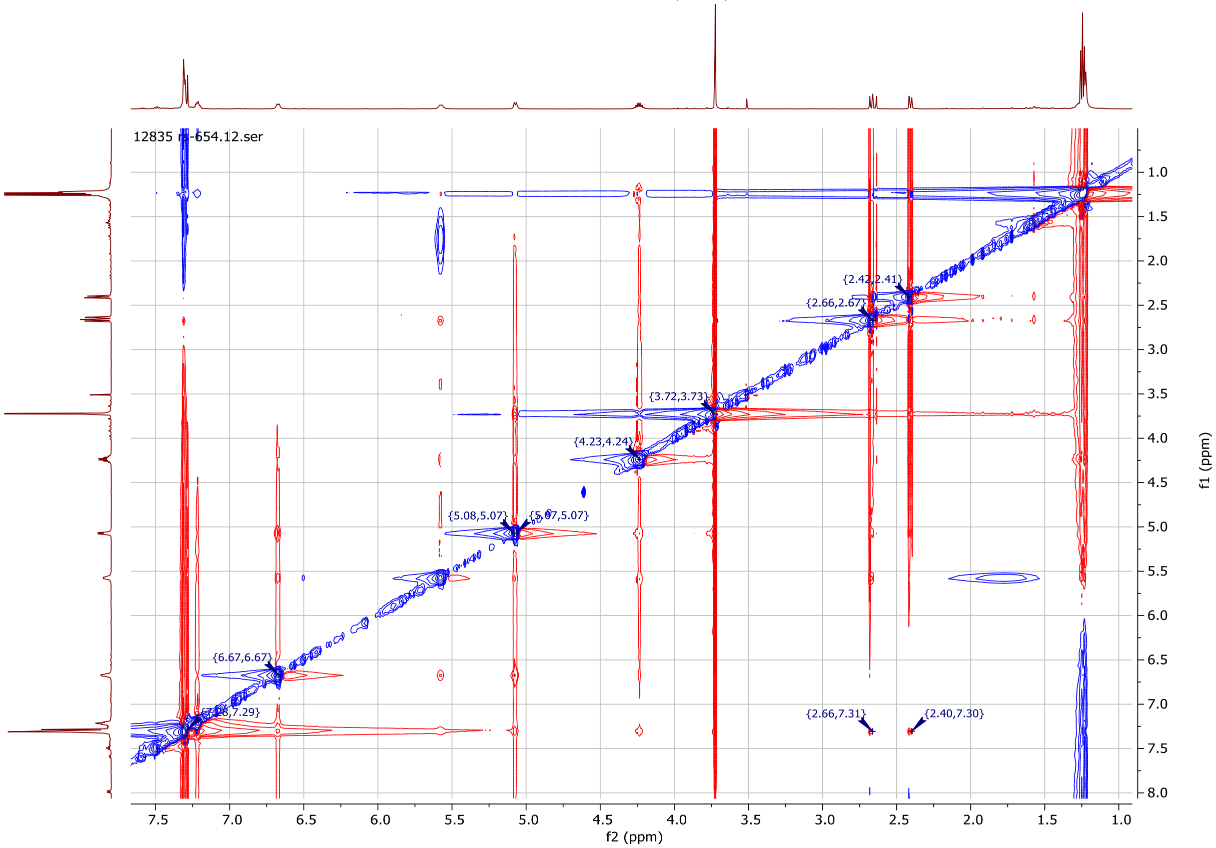

HPLC

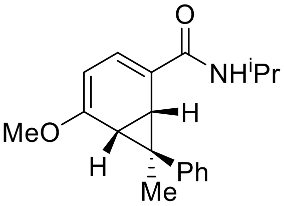

(1*S*,6*R*,7*R*)-*N*-isopropyl-5-methoxy-7-methyl-7-phenylbicyclo[4.1.0]hepta-2,4-diene-2-carboxamide (12e)

Analytical HPLC (Chiral Regis Whelk O1), eluting with hexane-IPA (70:30), showed it to consist of a 2:98 mixture of two enantiomers with retention times 14.253 mins (minor) and 22.635 mins (major), respectively.

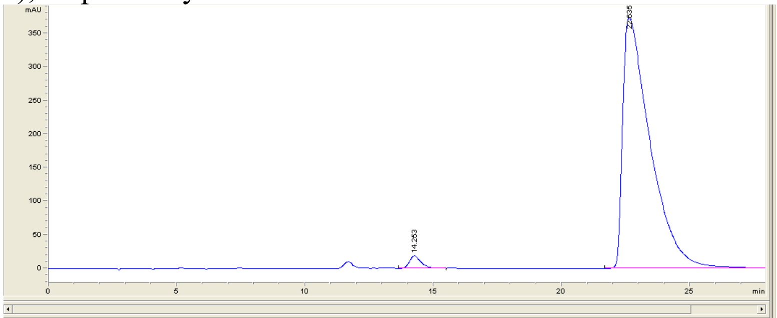

File Information

LC-File	RS-654 PREP.D
File Path	E:\CHEM32\1\DATA\RAKESH\RAKESH S 2020-08-25 13-08-59\
Date	25-Aug-20, 13:09:45
Sample	RS-654prep
Sample Info	70:30 hex:IPA IA
Barcode	
Operator	SYSTEM
Method	RAKESH 70-30 HEXANE-IPA1ML.M
Analysis Time	30.633 min
Sampling Rate	0.0067 min (0.402 sec), 4596 datapoints

#	Time	Area	Height	Width	Area%	Symmetry
1	14.253	570.4	18.8	0.4578	2.008	0.684
2	22.635	27843.8	373	1.107	97.992	0.293

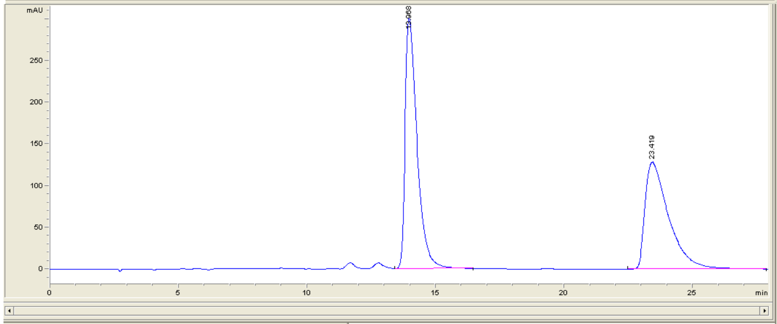

File Information

LC-File	RS-653 PREP.D
File Path	E:\CHEM32\1\DATA\RAKESH\RAKESH S 2020-08-25 12-39-27\
Date	25-Aug-20, 12:40:06
Sample	RS-653prep
Sample Info	70:30 hex:IPA IA
Barcode	
Operator	SYSTEM
Method	RAKESH 70-30 HEXANE-IPA1ML.M
Analysis Time	27.94 min
Sampling Rate	0.0067 min (0.402 sec), 4192 datapoints

#	Time	Area	Height	Width	Area%	Symmetry
1	13.968	9664.3	300	0.4839	54.040	0.475
2	23.419	8219.4	128.7	0.9413	45.960	0.398

^{1}H NMR

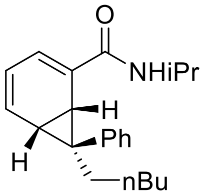

(1*R*,6*S*,7*S*)-*N*-isopropyl-7-pentyl-7-phenylbicyclo[4.1.0]hepta-2,4-diene-2-carboxamide (14)

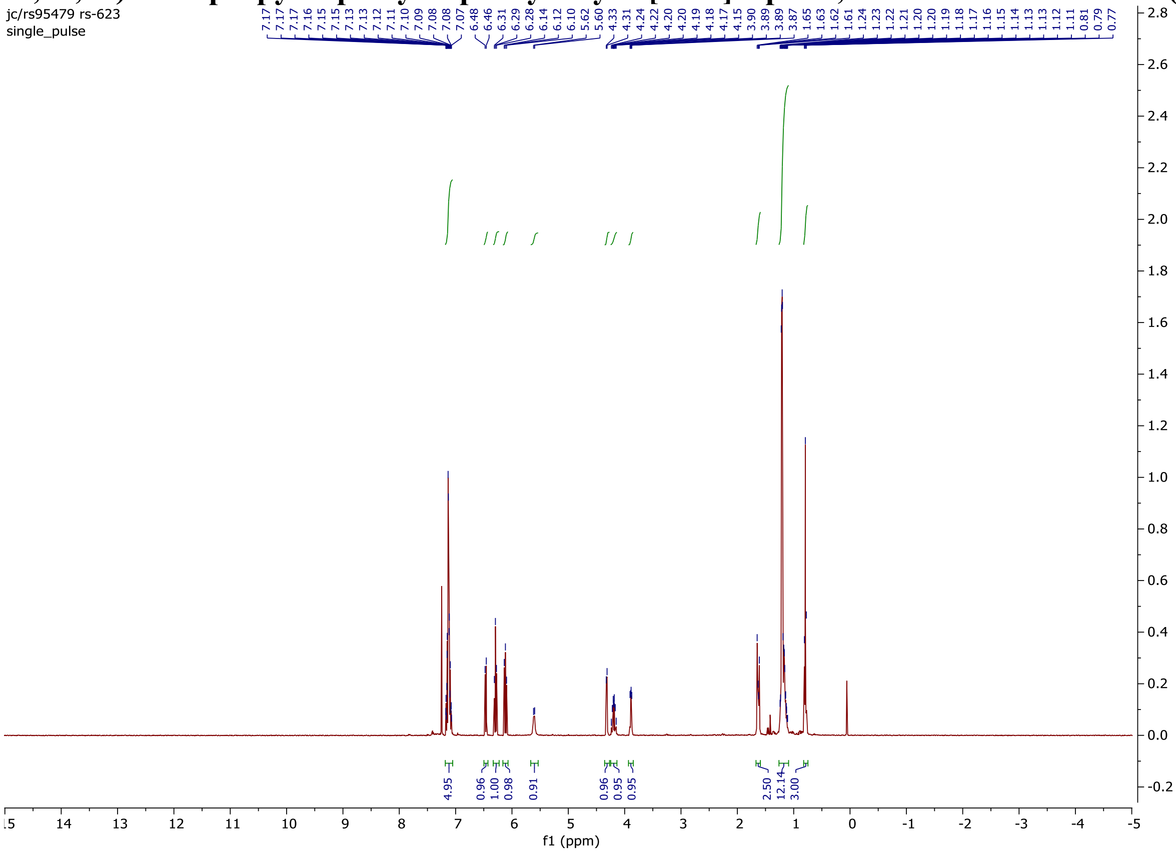

COSY

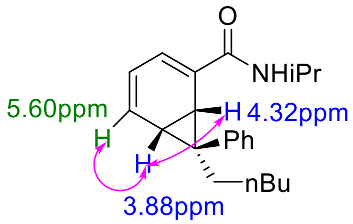

(1*R*,6*S*,7*S*)-*N*-isopropyl-7-pentyl-7-phenylbicyclo[4.1.0]hepta-2,4-diene-2-carboxamide (14)

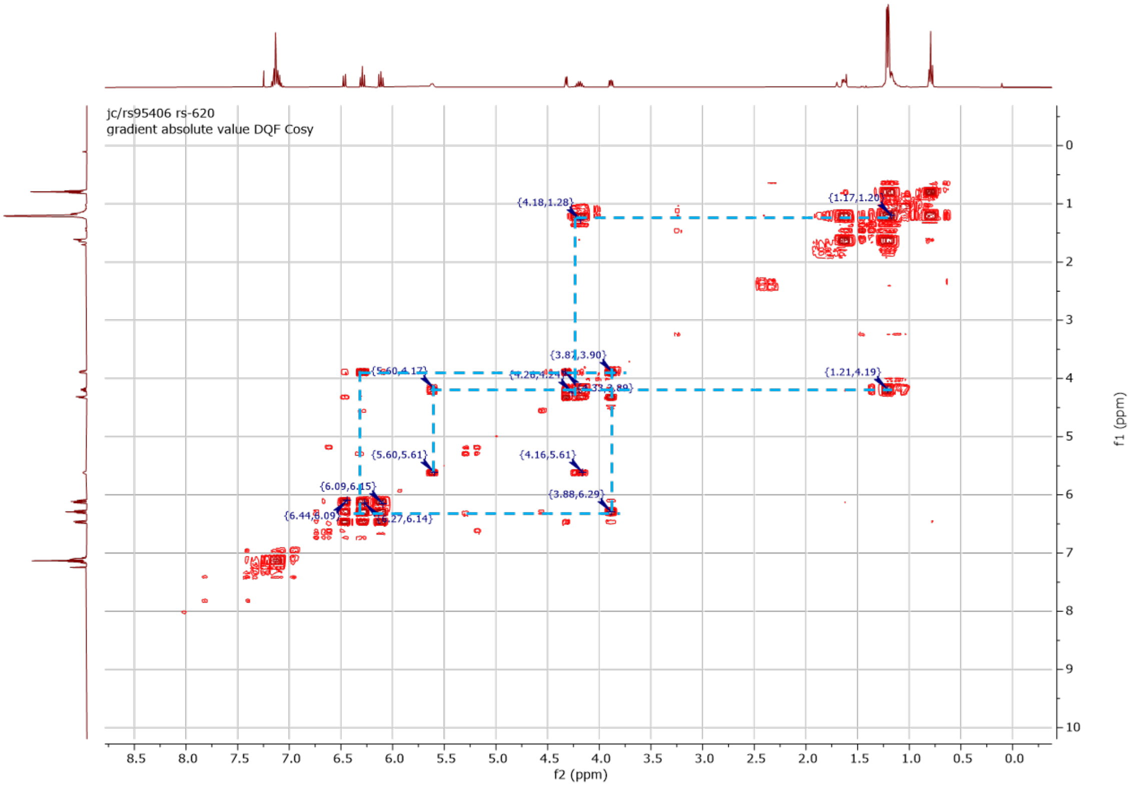

NOSY

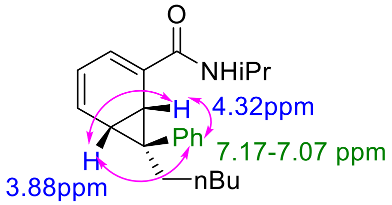

(1*R*,6*S*,7*S*)-*N*-isopropyl-7-pentyl-7-phenylbicyclo[4.1.0]hepta-2,4-diene-2-carboxamide (14)

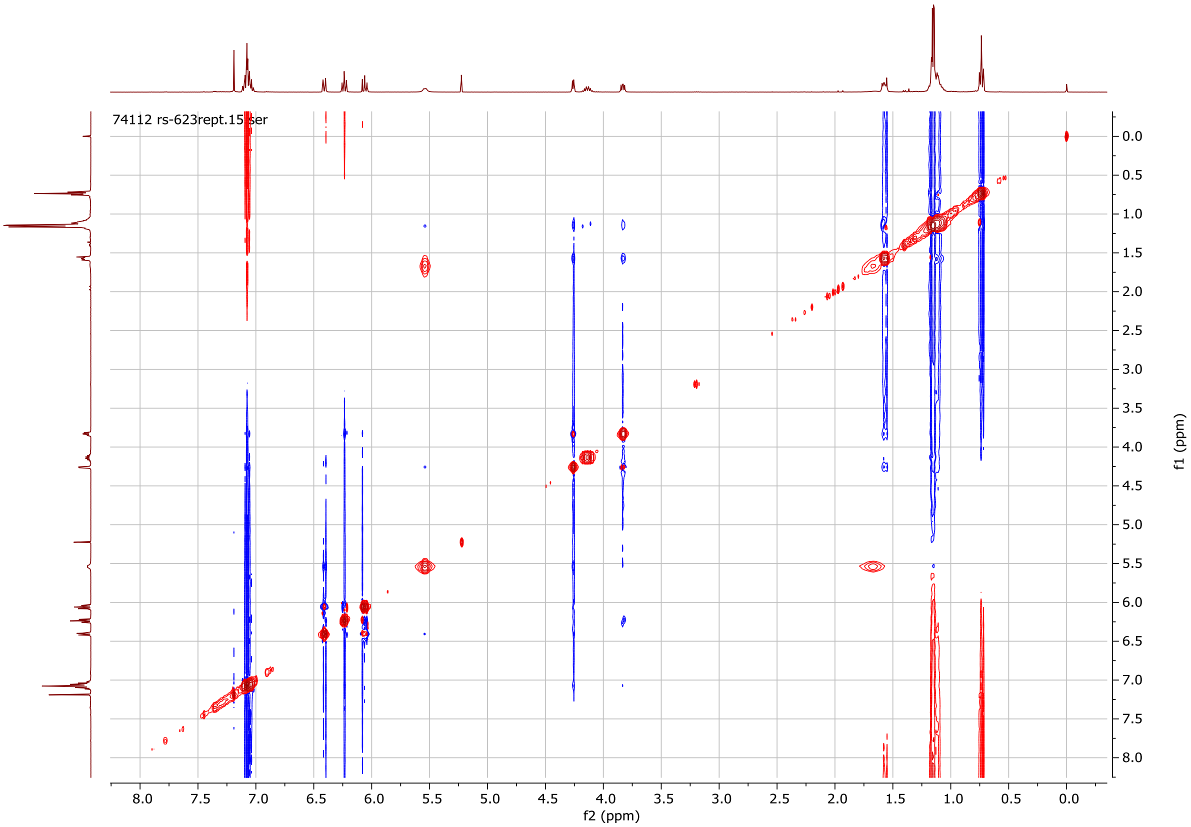

^{13}C NMR

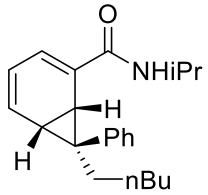

(1*R*,6*S*,7*S*)-*N*-isopropyl-7-pentyl-7-phenylbicyclo[4.1.0]hepta-2,4-diene-2-carboxamide (14)

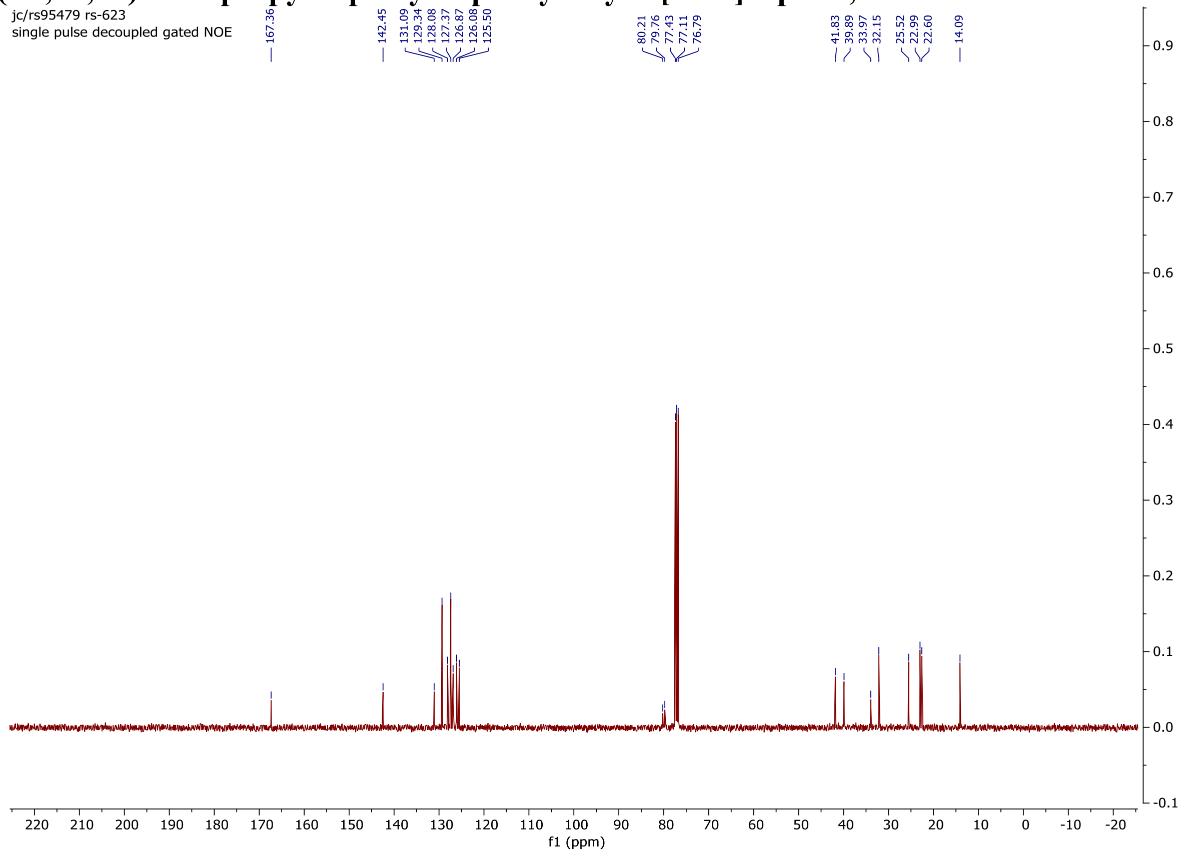

HMBC

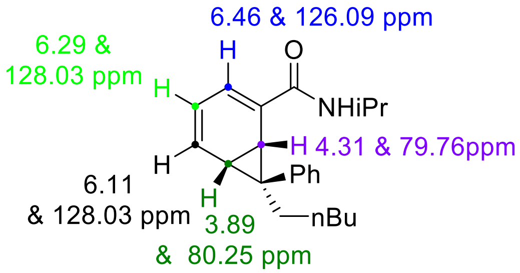

(1*R*,6*S*,7*S*)-*N*-isopropyl-7-pentyl-7-phenylbicyclo[4.1.0]hepta-2,4-diene-2-carboxamide (14)

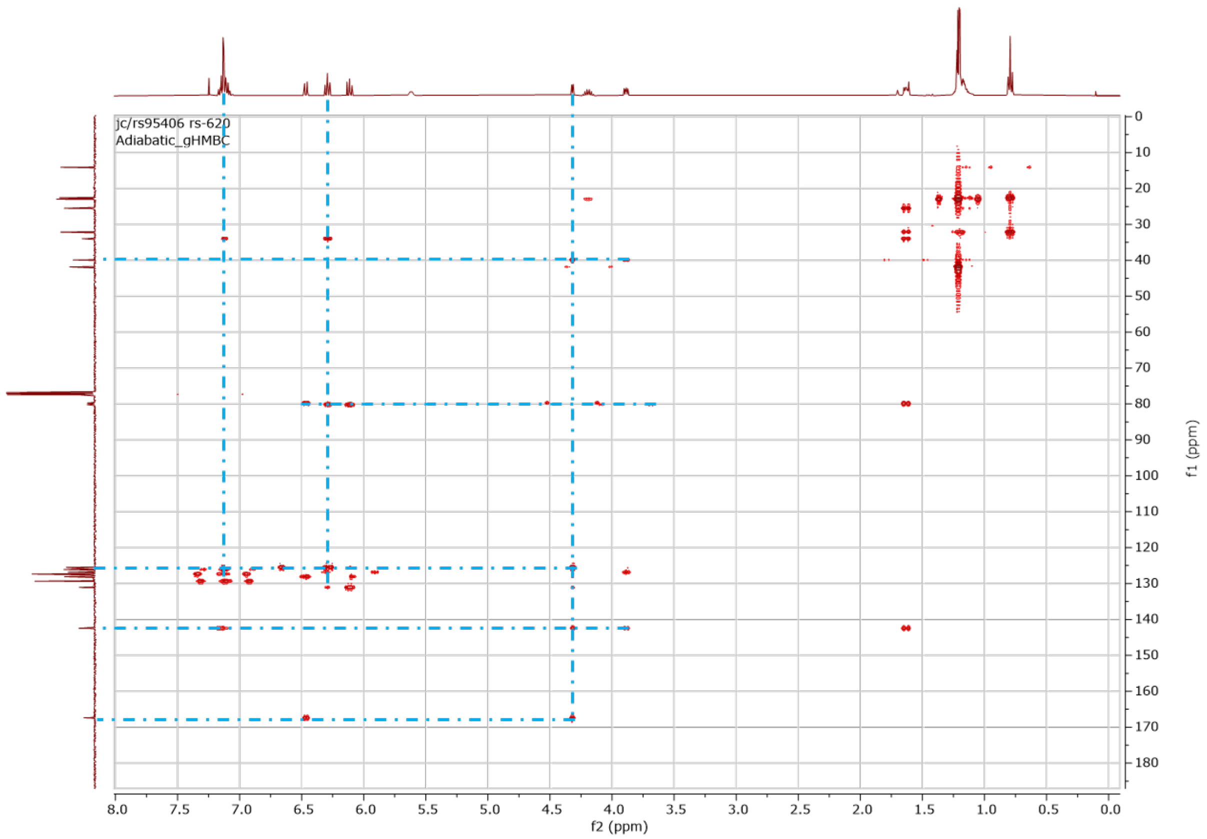

HSQC

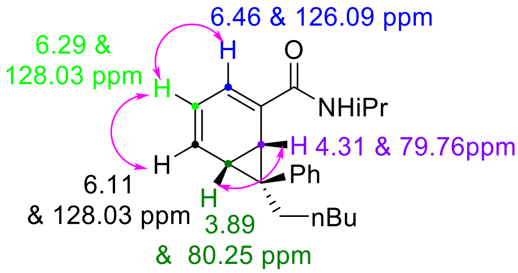

(1*R*,6*S*,7*S*)-*N*-isopropyl-7-pentyl-7-phenylbicyclo[4.1.0]hepta-2,4-diene-2-carboxamide (14)

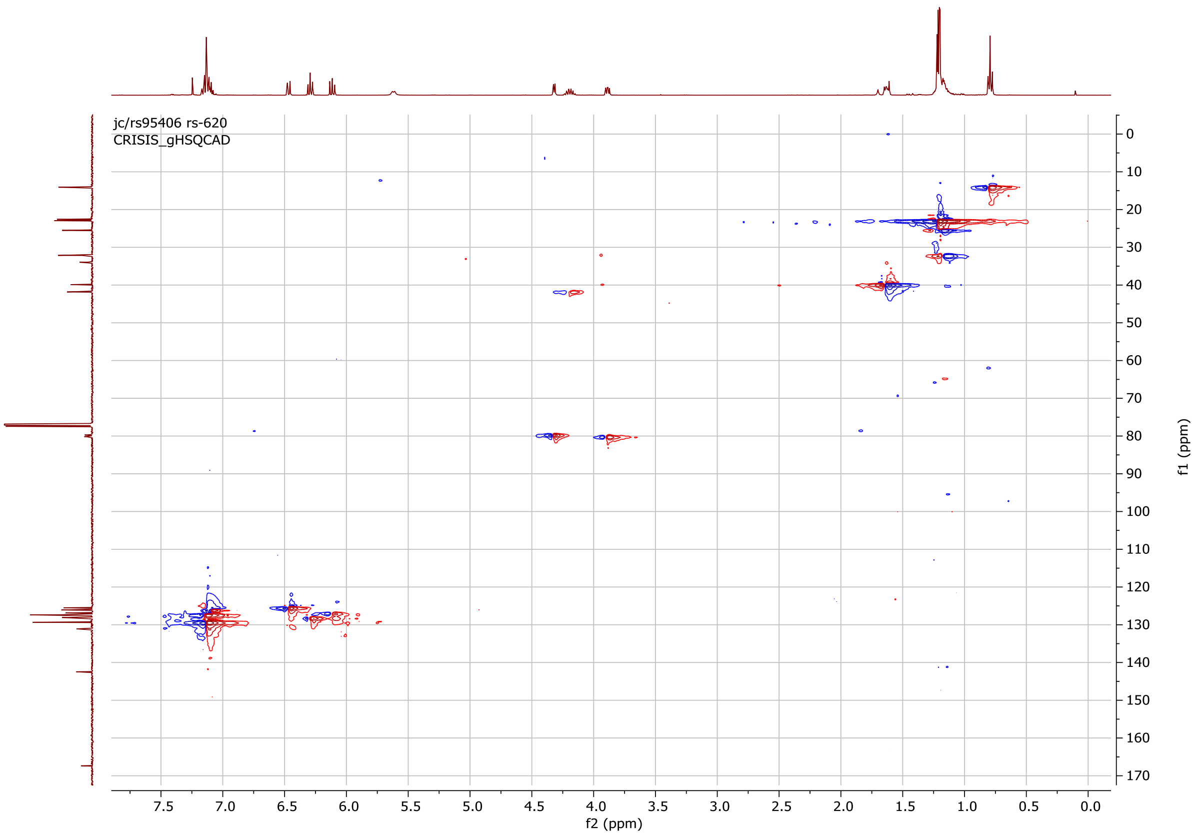

HPLC

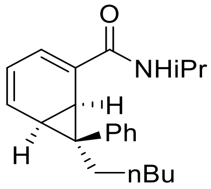

(1*R*,6*S*,7*S*)-*N*-isopropyl-7-pentyl-7-phenylbicyclo[4.1.0]hepta-2,4-diene-2-carboxamide (14)

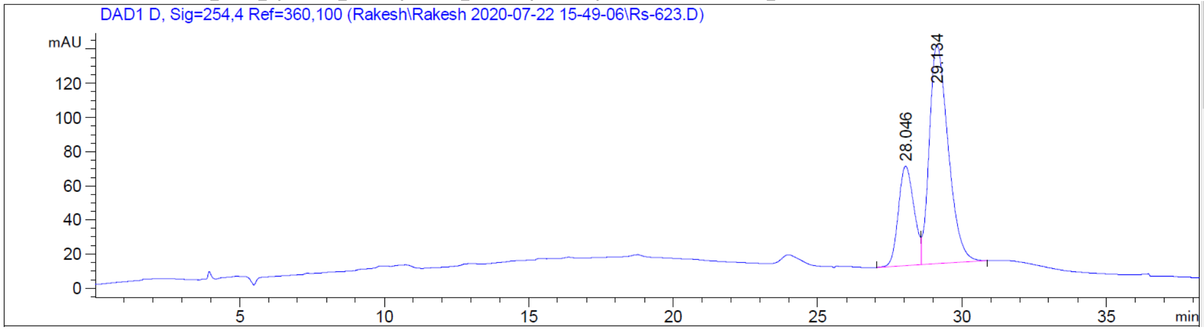

Signal 1: DAD1 D, Sig=254,4 Ref=360,100

Peak #	RetTime [min]	Type	Width [min]	Area [mAU*s]	Height [mAU]	Area %
1	28.046	BV	0.5827	2180.86279	58.26950	27.1906
2	29.134	VB	0.6485	5839.78369	127.94576	72.8094

Totals : 8020.64648 186.21527

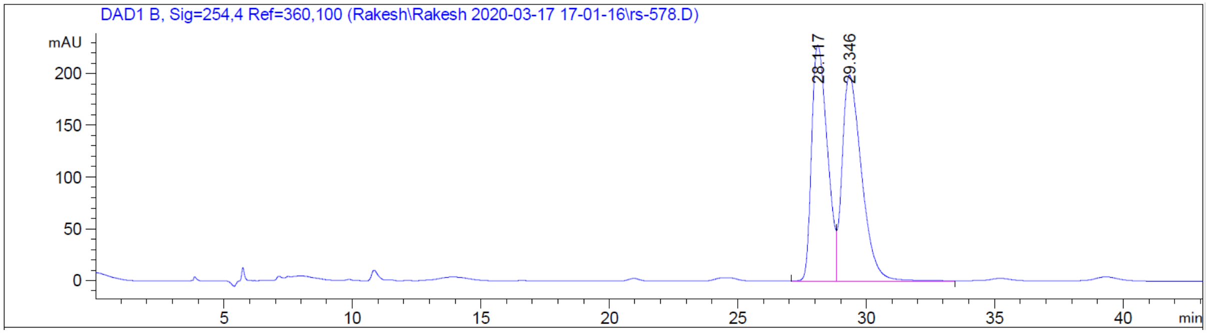

Signal 1: DAD1 B, Sig=254,4 Ref=360,100

Peak #	RetTime [min]	Type	Width [min]	Area [mAU*s]	Height [mAU]	Area %
1	28.117	BV	0.6686	9653.18652	227.72067	47.8699
2	29.346	VB	0.8067	1.05123e4	198.88542	52.1301

Totals : 2.01654e4 426.60609
